# Modular Synthesis of (Borylmethyl)silanes through Orthogonal Functionalization of a Carbon Atom

**DOI:** 10.1021/acs.orglett.3c00474

**Published:** 2023-03-10

**Authors:** Rajdip Chowdhury, Gábor Zoltán Elek, Beatriz Meana-Baamonde, Abraham Mendoza

**Affiliations:** † Department of Organic Chemistry, Arrhenius Laboratory, Stockholm University, 10691-Stockholm, Sweden; ‡ Department of Chemistry and Biotechnology, Tallinn University of Technology, 12618 Tallinn, Estonia; § Institute of Molecular Science (ICMol), University of Valencia, 46980 Paterna, Spain

## Abstract

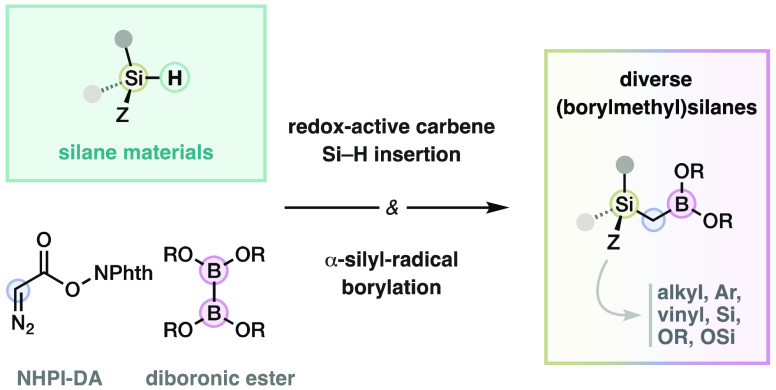

(Borylmethyl)trimethylsilanes are important building blocks in organic synthesis displaying a unique reactivity. Yet, the synthesis of more advanced derivatives is limited by the advanced silicon intermediates required for their preparation. Herein, a one-pot synthesis of (borylmethyl)silanes is developed, sourced on available alkyl-, aryl-, alkoxy-, aryloxy-, and silyl-hydrosilane materials. The privileged reactivity of *N*-hydroxyphthalimidyl diazoacetate (NHPI-DA) in Si–H insertion and α-silyl redox-active esters in different decarboxylative borylation reactions are scrutinized.

Organosilicon compounds are very important in organic synthesis and material science.^1^ These are useful not only to prepare silicon containing molecules, polymers, and surfaces, but also to address the selective synthesis of advanced organic compounds.^1^ In this context, an emerging family of reagents are (borylmethyl)trimethylsilanes (**1**),^2^ which count with orthogonal silicon and boron functionalization handles in the same carbon atom (Scheme 1). These reagents are used in the Matteson–Majumdar olefination to yield vinylboronates,^2a^ Suzuki cross-couplings to obtain allyl-^2c,2d^ or benzyltrimethylsilanes,^2f^ or epoxide opening/olefination cascades toward vinyltrimethylsilanes.^2g^ They also allow the synthesis of 1- and 2-borylalkyltrimethylsilanes, and α-trimethylsilylketones.^2b,2h^ Despite the unique reactivity of these compounds, only trimethylsilyl reagents have been explored due to the limitations in the synthesis of other organosilicon derivatives. (Borylmethyl)silanes **1** with advanced alkyl, aryl, alkoxy, or silyl substituents at silicon could (1) transfer more complex silyl groups, (2) tune the stereoelectronic properties of the reagents, and (3) facilitate the oxidation of the resulting silane products. (Borylmethyl)silanes **1** are now sourced in the more reactive chlorosilanes **2**, despite the larger number of commercial hydrosilanes and hydrosiloxane materials **3** (Scheme 1B), probably due to the lower reactivity of the Si–H bond.

**Scheme 1:**
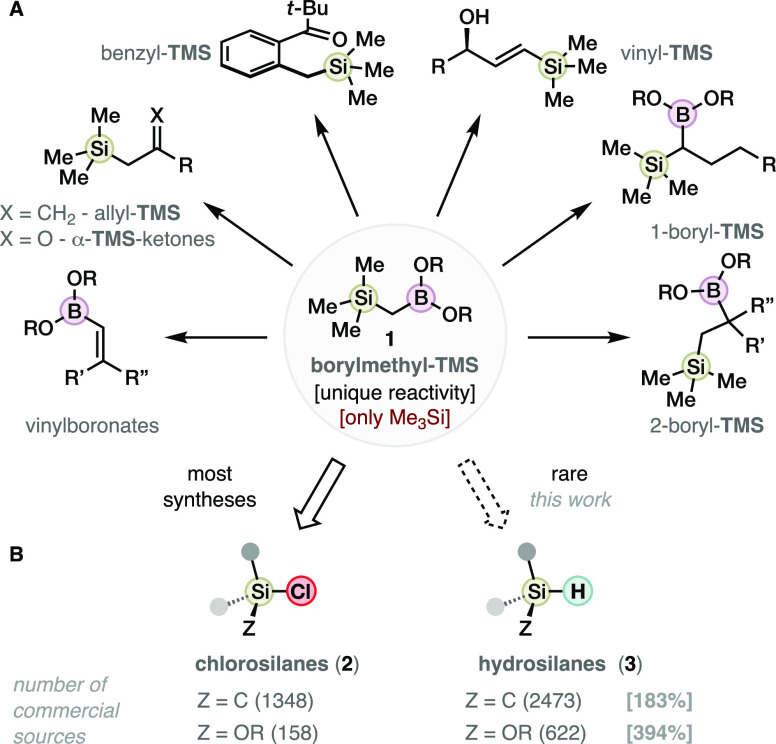
Applications of (Borylmethyl)trimethylsilanes in Organic Synthesis (A) and Availability of Organosilicon Sources (B)

At the moment, the most common route to obtain (borylmethyl)silanes **1** (Scheme 2A) requires the preparation of chloromethyltrialkylsilane **4** from the corresponding tetraalkylsilane or chlorosilane, followed by magnesiation to the α-silylmethyl Grignard **5**, and nucleophilic borylation with a borate.^2a,2b,3a,3b^ More recently, hydrosilane materials have been combined at high temperature with a designed methylboronic acid **6** to obtain a few (borylmethyl)silane derivatives as a proof of concept.^3c^ Lastly, iridium-catalyzed C–H borylation of methylchlorosilanes^3d^ and photoinduced C–H borylation of methylsilanes **7**(3e) have emerged to complement classic syntheses, but these methods require a large excess of materials and are still directly or indirectly sourced in chlorosilanes (**2**). Therefore, a general synthesis of (borylmethyl)silanes **1** using abundant alkyl-, aryl-, alkoxy-, aryloxy-, silyl-, and silyloxy-hydrosilane materials **3** (Scheme 2B) could enable the exploration of advanced geminal borosilanes.

**Scheme 2:**
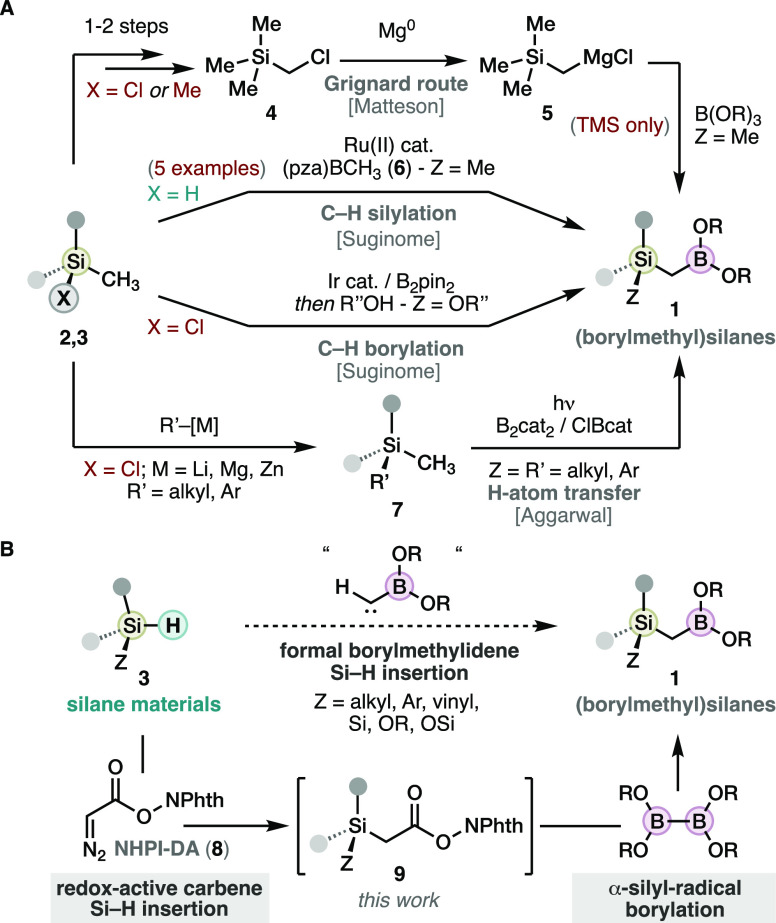
Existing Approaches toward (Borylmethyl)silanes (A), and the Borylmethylidene Si–H Insertion Pursued Herein (B)

Our group has recently demonstrated the orthogonality of the *N*-hydroxyphthalimide (NHPI) ester moiety in carbene transfer processes^4^ such as asymmetric cyclopropanation reactions^4a−4d^ and indole C–H insertion.^4e^ In this context, we hypothesized that the redox-active diazo compound *N*-hydroxyphthalimidoyl diazoacetate (NHPI-DA, **8**; Scheme 2,B) could engage in mild carbene *ipso*-hydrosilylation with hydrosilane materials **3**. Related reactions have been extensively studied with conventional diazo compounds^5,6^ but never with redox-active carbene precursors before our work. The compatibility of the redox-active ester (RAE) group to the electrophilic Si–H insertion intermediates and the reductive silane materials were reasonable concerns. The resulting redox-active α-silyl acetates (**9**) should undergo decarboxylative borylation to yield diverse (borylmethyl)silanes (**1**). However, the RAEs **9** only were known to participate in one example of decarboxylative acylation (C–C coupling),^7^ and therefore their potential in borylation reactions via α-silyl radicals was unknown.

The abundant literature in Si–H carbene insertion^6,8,9^ gave us ample possibilities to explore suitable transition metal catalysts to engage NHPI-DA (**8**). However, those most successful with conventional diazo compounds such as rhodium(II) carboxylates, copper bipyridine or bisoxazolines, iron porphyrin or phthalocyanines all proved to be ineffective (≤21% yield; see SI).^8,9^ In line with our previous studies on NHPI-DA (**8**),^4e^ it was found that the achiral ruthenium metallacycle [Ru(Me_2_-Pheox)]PF_6_ (**10**) was unique at catalyzing the insertion of **8** into the model tris(isopropyl)silane (**3a**), producing the desired compound **9a** in 92% yield (Scheme 3). Remarkably, this performance is achieved with only 1 mol % catalyst loading, in 30 min reaction time, and without excess nor slow addition of the diazo compound. This contrasts with the usual efficiencies obtained with conventional diazo compounds.^6,8,9^ It is important to mention that **10**, originally developed by Iwasa and co-workers,^10a^ was only known before to catalyze the Si–H insertion with α-functionalized diazoacetates.^10b^

**Scheme 3:**
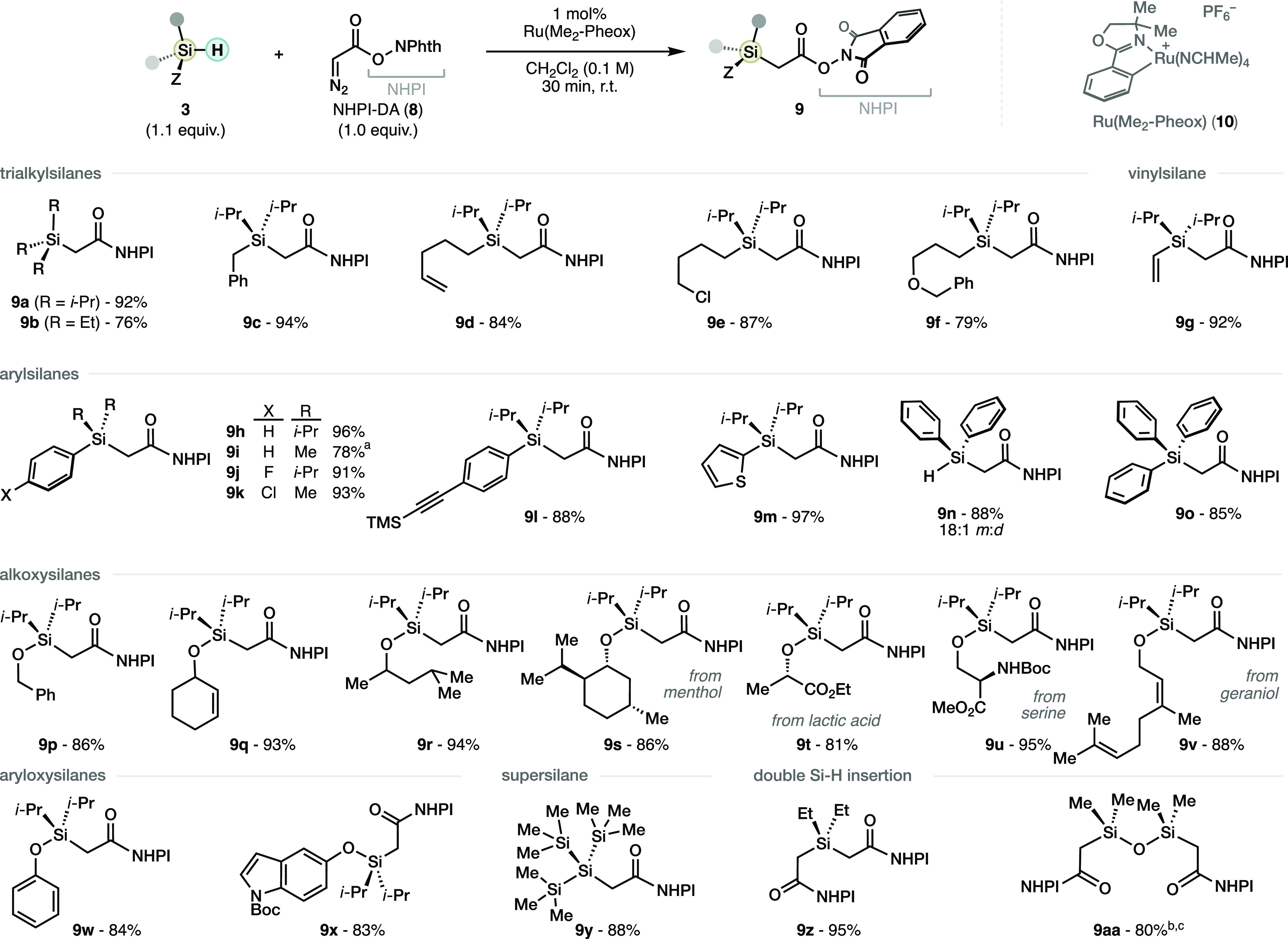
Scope of the Si–H Insertion Reaction Using NHPI-DA (**8**) Crude yield 90%. ^1^H NMR yield with internal standard due to instability. **10** (2 mol %), **3** (0.5 equiv) used. Isolated yields (0.3 mmol scale).

The performance of this catalytic system was elaborated in a wide range of diversely substituted silanes (Scheme 3). Initially, different trialkylsilanes were evaluated, which include the most common primary and secondary alkyl (**9a**,**b**), and benzyl substituents (**9c**). Aliphatic substrates with alkene, halide, and protected alcohol functions (**9d**–**f**) were also tolerated. The more challenging vinylsilane also seamlessly engaged in the reaction (**9g**). Importantly, olefin cyclopropanation was not observed to compete in substrates with alkenes (**9d**,**g**), indicating that silanes are more reactive toward NHPI-functionalized ruthenium carbenes. Arylsilanes (**9h**–**m**) also participated selectively in the *ipso*-hydrosilylation in the same conditions, including fluoro- (**9j**), chloro- (**9k**), and alkynyl- (**9l**) functionalities. Interestingly, the electron-rich thiophenyl heterocycle could also be included (**9m**). The more reactive polyarylsilanes (**9n**,**o**) swiftly produced the expected RAEs, with almost exclusive *mono*-insertion selectivity in the case of diphenylsilane (**9n**). Alkoxysilanes are more sensitive and rarely explored in carbene transfer reactions,^11^ but these engaged in the standard conditions (**9p**–**x**). Derivatives were obtained from allylic alcohols (**9q**,**v**) and chiral natural products including a terpene (**9s**), an α-hydroxy acid (**9t**), and an α-amino acid (**9u**). In this sense, we questioned the system with more labile aryloxysilanes that were never reported to engage in Si–H insertion with diazo esters. Pleasingly, efficient insertion with aryloxysilanes bearing aryl (**9w**) and indole (**9x**) substituents was found. Furthermore, the supersilane featuring Si–Si bonds efficiently delivered the extremely bulky derivative **9y**, and silanes with two Si–H bonds produced the double insertion products either at the same (**9z**) or distinct (**9aa**) silicon atoms. Overall, the ruthenium-catalyzed Si–H insertion using NHPI-DA (**8**) displays an unprecedented generality in a wide range of hydrosilane compound families, and allows swift access to diverse α-silyl RAEs.

While the decarboxylative borylation of conventional RAEs has been intensively developed recently,^12^ the viability of α-silyl radical intermediates was unknown to the best of our knowledge. Thus, we set out to explore the borylation methods by Baran^12a,12b^ and Aggarwal^12c^ in the representative substrates **9a**,**b**,**i**,**o**,**r** (Table 1), evidencing the strong stereoelectronic influence of the silyl substituents in the efficiency of the borylation reactions. The photoinduced method developed by Aggarwal^12c^ was efficient with the less hindered alkylsilanes (entries 2, 3). In stark contrast, the nickel- and copper-catalyzed protocols developed by Baran^12a,12c^ demonstrated to be relatively insensitive to steric bulk (entry 1), and performed in the more challenging alkoxysilane class (entry 5), albeit in low yield. Among these two methods, the copper-catalyzed conditions were superior in the triarylsilane case (entry 4), and allowed telescoping in one-pot from hydrosilanes **3** (entry 2; yields in parentheses). Thus, the copper-catalyzed method was included in the standard protocol for the desired insertion–borylation process.

**Table 1:**
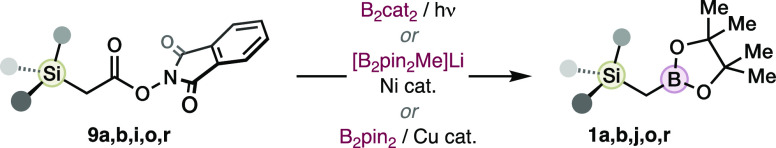
Decarboxyative Borylation of α-Silyl RAEs

		yield **1** (%)^a^		
entry	[Si]	B_2_cat_2_ hν^b^^,^^e^	[B_2_pin_2_Me]Li Ni cat.^c^^,^^e^	B_2_pin_2_ Cu cat.^d^^,^^e^
1	(*i*-Pr)_3_Si (**9a**)	0	77	76
2	Et_3_Si (**9b**)	59 (53)	65 (9)	77 (63)
3	PhMe_2_Si (**9i**)	75	17	27
4	Ph_3_Si (**9o**)	21	38	53
5	(*i*-Pr)_2_(OR)Si (**9r**)	0	21	32

a ^1^H NMR yields using 1,1,2,2-tetrachloroethane as internal standard. In parentheses, yields of the one-pot Si–H insertion/decarboxylative borylation from silane **2b**.

b Ref (12c).

c Ref (12a).

d Ref (12b).

e See SI for details.

With this knowledge in hand, we examined the scope of the one-pot insertion–borylation protocol of hydrosilane materials **3** (Scheme 4). In general, functionalized trialkylsilanes (**1a**–**g**) provided the corresponding (borylmethyl)silanes in good yields, including an alkyl chloride (**1f**) that would be challenging to obtain through the established Grignard route. The (borylmethyl)silane derived from vinylsilane (**1h**) could also be easily obtained under the standard conditions in moderate yield. Arylsilanes (**1i**–**o**) can also furnish the corresponding (borylmethyl)silane products decorated with halogens (**1k**,**l**), alkyne (**1m**), and (hetero)aromatic (**1n**) groups. Nevertheless, the derivative of the bulkier triphenylsilane was obtained albeit in lower yield. Remarkably, the (borylmethyl)silane derived from the extremely bulky supersilane (**1p**) could be seamlessly obtained using this protocol. Several alkoxysilanes were evaluated despite their unexplored potential in decarboxylative coupling reactions. Alkoxysilane derivatives engaged in the methylborylation in general with moderate yields (**1q**–**s**), despite the relatively complex alkoxy group. The more sensitive aryloxysilanes (**1t**,**u**) resulted understandably in lower yields. Finally we found that the doubly methylborylated product **1v** derived from tetramethyldisiloxane could be obtained under the standard conditions, albeit only in low yield. The main byproduct in these reactions are methylsilanes derived from hydrogen atom transfer (HAT) to the intermediate α-silyl radical. Thus, lower yields are observed in substrates where either intramolecular cyclization^13a,13b^ (**1e**) or HAT^13c^ (**1r**) is possible. To put this results in perspective, it is important to highlight that the moderate yields obtained are still higher than those obtained with previous methods, as illustrated by compounds **1d**,**j**.^3e^ Also, silanes containing vinyl (**1h**), alkynyl (**1m**), and electron-rich (hetero)aryl (**1n**,**t**,**u**) moieties would all be expected to be problematic in the electrophilic reactions previously available (Scheme 2A).^3c−3e^ The (borylmethyl)silanes derived from dialkoxy- and trialkoxysilanes are yet beyond reach. Future upgrades in decarboxylative borylation reactions^12^ may expand the potential of α-silyl RAEs obtained by the NHPI-DA Si–H insertion presented herein (Scheme 3).

**Scheme 4:**
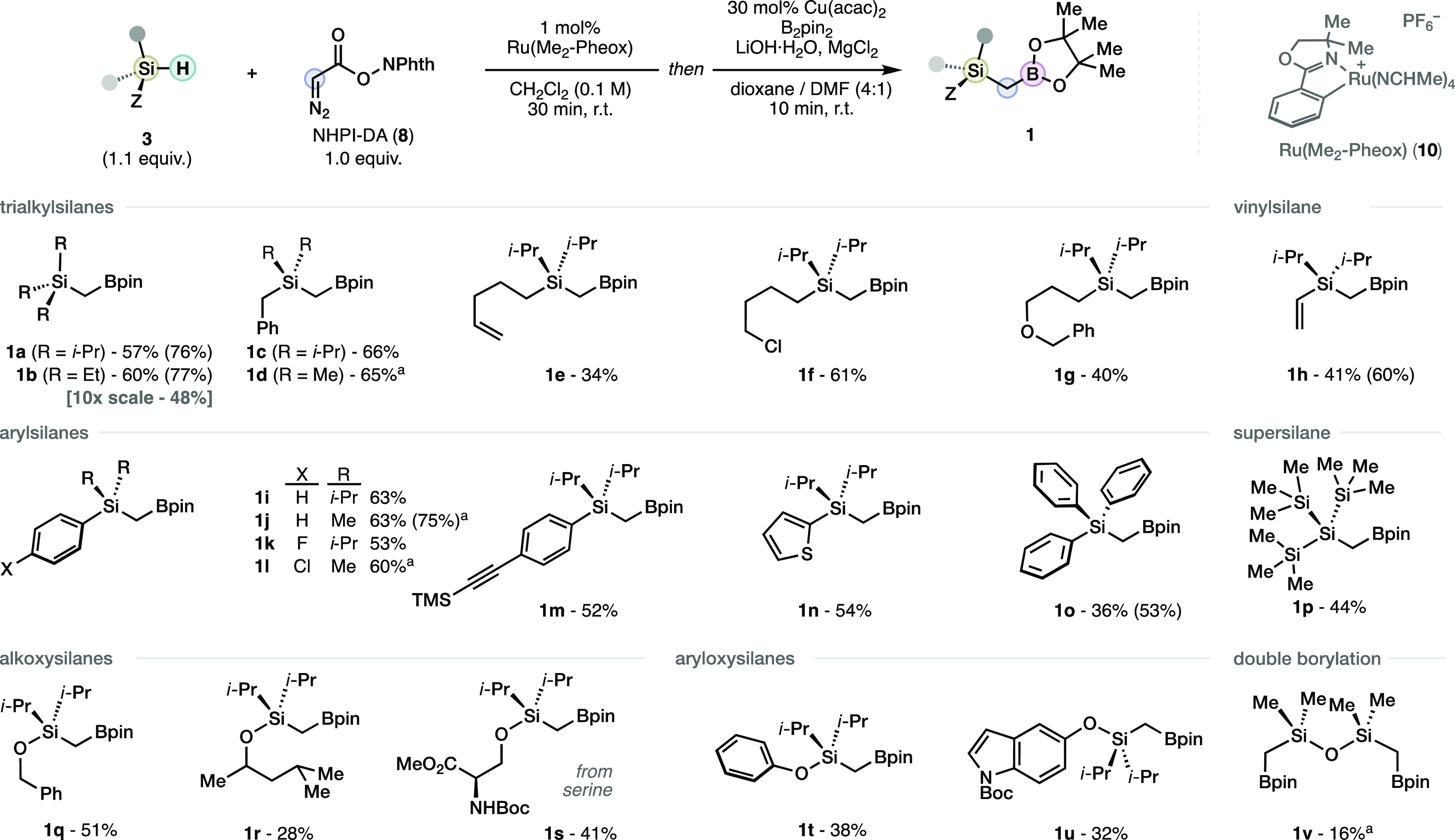
Scope of the One-Pot Methylborylation of Hydrosilanes Using NHPI-DA (**8**) Photoinduced borylation was used instead (see SI).^12c^ Isolated yields. In parentheses, yields from the isolated RAE.

In summary, we have developed a one-pot protocol for the synthesis of (borylmethyl)silanes from various hydrosilane materials using the redox-active diazo compound NHPI-DA as a borylmethylidene surrogate. A ruthenium metallacyclic catalyst enables Si–H insertion in an unprecedented wide range of organosilanes. The stereoelectronic properties of the resulting α-silyl redox-active esters have been demonstrated to have a critical impact in their decarboxylative borylation. These advances allow the synthesis of (borylmethyl)silanes from available alkyl-, aryl-, alkoxy-, aryloxy-, silyloxy-, and tris(trialkylsilyl)-hydrosilanes. Considering the current versatility displayed by (borylmethyl)silanes,^2^ it is expected that the newly developed protocol will expedite further applications of these privileged reagents.

## Data Availability

The data underlying this study are available in the published article and its Supporting Information. Raw data is available for download from Zenodo at http://dx.doi.org/10.5281/zenodo.7673449.

## Supporting Information Available

Experimental procedures, optimization details, and characterization data (PDF)
