# Supplementary material for: Modular Synthesis of (Borylmethyl)silanes through Orthogonal Functionalization of a Carbon Atom
Source: Org Lett. 2023 Mar 10;25(11):1935–40. doi: 10.1021/acs.orglett.3c00474 (PMC10043938; doi:10.1021/acs.orglett.3c00474)

Supporting Information

Modular synthesis of (borylmethyl)silanes through orthogonal functionalization of a carbon atom

Rajdip Chowdhury,[a] Gábor Zoltán Elek,[a,b] Beatriz Meana-Baamonde[a,c] and Abraham Mendoza*[a,c]

[a] Dept. of Organic Chemistry, Arrhenius laboratory,
Stockholm University, 106 91 Stockholm (Sweden)

[b] Department of Chemistry and Biotechnology,
Tallinn University of Technology, 12618 Tallinn (Estonia)

[c] Institute of Molecular Science (ICMol)
University of Valencia, 46980 Paterna (Spain)

Raw data for this manuscript is available for download from Zenodo
(http://dx.doi.org/10.5281/zenodo.7673449)

Corresponding author email: abraham.mendoza@uv.es

Table of Contents

1. General Information 1

2. Preparation of Starting Materials 2

2.1 General procedure A: Preparation of Grignard reagents 2

2.2 General procedure B: Reaction of the freshly prepared Grignard reagents (using general procedure A) with chlorodiisopropylsilane 2

2.3 General procedure C: Synthesis of alkoxysilanes using DMAP and triethylamine 3

2.4 General procedure D: Synthesis of alkoxysilanes using imidazole 3

2.5 Synthesis of silane materials 3

Synthesis of diisopropyl(pent-4-en-1-yl)silane (**3d**) 3

Synthesis of (4-chlorobutyl)diisopropylsilane (**3e**) 4

Synthesis of (3-(benzyloxy)propyl)diisopropylsilane (**3f**) 4

Synthesis of (4-fluorophenyl)diisopropylsilane (**3j**) 5

Synthesis of ((4-(diisopropylsilyl)phenyl)ethynyl)trimethylsilane (**3l**) 5

Synthesis of diisopropyl(thiophen-2-yl)silane (**3m**) 6

Synthesis of (cyclohex-2-en-1-yloxy)diisopropylsilane (**3q**) 6

Synthesis of diisopropyl((4-methylpentan-2-yl)oxy)silane (**3r**) 7

Synthesis of diisopropyl(((1S,2R,5S)-2-isopropyl-5-methylcyclohexyl)oxy)silane (**3s**) 8

Synthesis of ethyl (R)-2-((diisopropylsilyl)oxy)propanoate (**3t**) 8

Synthesis of methyl N-(tert-butoxycarbonyl)-O-(diisopropylsilyl)-D-serinate (**3u**) 9

Synthesis of (E)-((3,7-dimethylocta-2,6-dien-1-yl)oxy)diisopropylsilane (**3v**) 9

Synthesis of tert-butyl 5-((diisopropylsilyl)oxy)-1H-indole-1-carboxylate (**3x**) 10

3. Results and Discussion 11

3.1 Optimization of the Si−H insertion reaction using NHPI-DA (8). 11

3.2 General procedure E: Ru(II)-Pheox catalyzed Si–H insertion of silanes 11

Synthesis of 1,3-dioxoisoindolin-2-yl 2-(triisopropylsilyl)acetate (**9a**) 12

Synthesis of 1,3-dioxoisoindolin-2-yl 2-(triethylsilyl)acetate (**9b**) 12

Synthesis of 1,3-dioxoisoindolin-2-yl 2-(benzyldiisopropylsilyl)acetate (**9c**) 13

Synthesis of 1,3-dioxoisoindolin-2-yl 2-(diisopropyl(pent-4-en-1-yl)silyl)acetate (**9d**) 13

Synthesis of 1,3-dioxoisoindolin-2-yl 2-((4-chlorobutyl)diisopropylsilyl)acetate (**9e**) 14

Synthesis of 1,3-dioxoisoindolin-2-yl 2-((3-(benzyloxy)propyl)diisopropylsilyl)acetate (**9f**) 14

Synthesis of 1,3-dioxoisoindolin-2-yl 2-(diisopropyl(vinyl)silyl)acetate (**9g**) 15

Synthesis of 1,3-dioxoisoindolin-2-yl 2-(diisopropyl(phenyl)silyl)acetate (**9h**) 15

Synthesis of 1,3-dioxoisoindolin-2-yl 2-(dimethyl(phenyl)silyl)acetate (**9i**) 16

Synthesis of 1,3-dioxoisoindolin-2-yl 2-((4-fluorophenyl)diisopropylsilyl)acetate (**9j**) 16

Synthesis of 1,3-dioxoisoindolin-2-yl 2-((4-chlorophenyl)dimethylsilyl)acetate (**9k**) 17

Synthesis of 1,3-dioxoisoindolin-2-yl 2-(diisopropyl(4-((trimethylsilyl)ethynyl)phenyl)silyl)acetate (**9l**) 17

Synthesis of 1,3-dioxoisoindolin-2-yl 2-(diisopropyl(thiophen-2-yl)silyl)acetate (**9m**) 18

Synthesis of 1,3-dioxoisoindolin-2-yl 2-(diphenylsilyl)acetate (**9n**) 18

Synthesis of 1,3-dioxoisoindolin-2-yl 2-(triphenylsilyl)acetate (**9o**) 19

Synthesis of 1,3-dioxoisoindolin-2-yl 2-((benzyloxy)diisopropylsilyl)acetate (**9p**) 19

Synthesis of 1,3-dioxoisoindolin-2-yl 2-((cyclohex-2-en-1-yloxy)diisopropylsilyl)acetate (**9q**) 20

Synthesis of 1,3-dioxoisoindolin-2-yl 2-(diisopropyl((4-methylpentan-2-yl)oxy)silyl)acetate (**9r**) 20

Synthesis of 1,3-dioxoisoindolin-2-yl 2-(diisopropyl(((1S,2R,5S)-2-isopropyl-5-methylcyclohexyl)oxy)silyl)acetate (**9s**) 21

Synthesis of ethyl (R)-2-(((2-((1,3-dioxoisoindolin-2-yl)oxy)-2-oxoethyl)diisopropylsilyl)oxy)propanoate (**9t**) 22

Synthesis of 1,3-dioxoisoindolin-2-yl (R)-3,3-diisopropyl-6-(methoxycarbonyl)-10,10-dimethyl-8-oxo-4,9-dioxa-7-aza-3-silaundecanoate (**9u**) 22

Synthesis of 1,3-dioxoisoindolin-2-yl (E)-2-(((2,7-dimethylocta-2,6-dien-1-yl)oxy)diisopropylsilyl)acetate (**9v**) 23

Synthesis of 1,3-dioxoisoindolin-2-yl 2-(diisopropyl(phenoxy)silyl)acetate (**9w**) 23

Synthesis of tert-butyl 5-(((2-((1,3-dioxoisoindolin-2-yl)oxy)-2-oxoethyl)diisopropylsilyl)oxy)-1H-indole-1-carboxylate (**9x**) 24

Synthesis of redox-active ester derived from 1,1,1,3,3,3-hexamethyl-2-(trimethylsilyl)trisilane (**9y**) 24

Synthesis of redox-active ester derived from diethylsilane (**9z**) 25

Synthesis of redox-active ester derived from 1,1,1,3,3,3-hexamethyl-2-(trimethylsilyl)trisilane (**9aa**) 25

4. Diversification of the α-Silyl Redox-Active Ester Products 26

4.1 General procedure F: One-pot methylborylation of silanes using NHPI-DA (8) 26

4.2 General procedure G: One-pot methylborylation of dimethylsilanes using a photo-induced approach 26

Synthesis of triisopropyl((4,4,5,5-tetramethyl-1,3,2-dioxaborolan-2-yl)methyl)silane (**1a**) 28

Synthesis of triethyl((4,4,5,5-tetramethyl-1,3,2-dioxaborolan-2-yl)methyl)silane (**1b**) 28

Synthesis of benzyldiisopropyl((4,4,5,5-tetramethyl-1,3,2-dioxaborolan-2-yl)methyl)silane (**1c**) 29

Synthesis of benzyldimethyl((4,4,5,5-tetramethyl-1,3,2-dioxaborolan-2-yl)methyl)silane (**1d**) ..29

Synthesis of (4-chlorobutyl)diisopropyl((4,4,5,5-tetramethyl-1,3,2-dioxaborolan-2-yl)methyl)silane (**1f**) 30

Synthesis of (3-(benzyloxy)propyl)diisopropyl((4,4,5,5-tetramethyl-1,3,2-dioxaborolan-2-yl)methyl)silane (**1g**) 31

Synthesis of diisopropyl((4,4,5,5-tetramethyl-1,3,2-dioxaborolan-2-yl)methyl)(vinyl)silane (**1h**) 32

Synthesis of diisopropyl(phenyl)((4,4,5,5-tetramethyl-1,3,2-dioxaborolan-2-yl)methyl)silane (**1i**) 32

Synthesis of dimethyl(phenyl)((4,4,5,5-tetramethyl-1,3,2-dioxaborolan-2-yl)methyl)silane (**1j**) 33

Synthesis of (4-fluorophenyl)diisopropyl((4,4,5,5-tetramethyl-1,3,2-dioxaborolan-2-yl)methyl)silane (**1k**) 33

Synthesis of (4-chlorophenyl)dimethyl((4,4,5,5-tetramethyl-1,3,2-dioxaborolan-2-yl)methyl)silane (**1l**) 34

Synthesis of ((4-(diisopropyl((4,4,5,5-tetramethyl-1,3,2-dioxaborolan-2-yl)methyl)silyl)phenyl)ethynyl)trimethylsilane (**1m**) 34

Synthesis of diisopropyl((4,4,5,5-tetramethyl-1,3,2-dioxaborolan-2-yl)methyl)(thiophen-2-yl)silane (**1n**) 35

Synthesis of triphenyl((4,4,5,5-tetramethyl-1,3,2-dioxaborolan-2-yl)methyl)silane (**1o**) 36

Synthesis of 1,1,1,3,3,3-hexamethyl-2-((4,4,5,5-tetramethyl-1,3,2-dioxaborolan-2-yl)methyl)-2-(trimethylsilyl)trisilane (**1p**) 36

Synthesis of (benzyloxy)diisopropyl((4,4,5,5-tetramethyl-1,3,2-dioxaborolan-2-yl)methyl)silane (**1q**) 37

Synthesis of diisopropyl((4-methylpentan-2-yl)oxy)((4,4,5,5-tetramethyl-1,3,2-dioxaborolan-2-yl)methyl)silane (**1r**) 37

Synthesis of methyl N-(tert-butoxycarbonyl)-O-(diisopropyl((4,4,5,5-tetramethyl-1,3,2-dioxaborolan-2-yl)methyl)silyl)-D-serinate (**1s**) 38

Synthesis of diisopropyl(phenoxy)((4,4,5,5-tetramethyl-1,3,2-dioxaborolan-2-yl)methyl)silane (**1t**) 39

Synthesis of tert-butyl 5-((diisopropyl((4,4,5,5-tetramethyl-1,3,2-dioxaborolan-2-yl)methyl)silyl)oxy)-1H-indole-1-carboxylate (**1u**) 39

Synthesis of 1,1,3,3-tetramethyl-1,3-bis((4,4,5,5-tetramethyl-1,3,2-dioxaborolan-2-yl)methyl)disiloxane (**1v**) 40

5. Scaled-up synthesis of triethyl((4,4,5,5-tetramethyl-1,3,2-dioxaborolan-2-yl)methyl)silane (**1b**) 40

6. References 42

7. NMR Spectra of Synthesized Compounds 43

^{1}H-NMR (400 MHz, $CDCl_3$) for compound **3d** 43

^{13}C-NMR (101 MHz, $CDCl_3$) for compound **3d** 44

^{1}H-NMR (400 MHz, $CDCl_3$) for compound **3e** 45

^{13}C-NMR (101 MHz, $CDCl_3$) for compound **3e** 46

^{1}H-NMR (400 MHz, $CDCl_3$) for compound **3f** 47

^{13}C-NMR (101 MHz, $CDCl_3$) for compound **3f** 48
^{1}H-NMR (400 MHz, $CDCl_3$) for compound **3j** 49
^{13}C-NMR (101 MHz, $CDCl_3$) for compound **3j** 50
^{19}F-NMR (377 MHz, $CDCl_3$) for compound **3j** 51
^{1}H-NMR (400 MHz, $CDCl_3$) for compound **3l** 52
^{13}C-NMR (101 MHz, $CDCl_3$) for compound **3l** 53
^{1}H-NMR (400 MHz, $CDCl_3$) for compound **3m** 54
^{13}C-NMR (101 MHz, $CDCl_3$) for compound **3m** 55
^{1}H-NMR (400 MHz, $CDCl_3$) for compound **3q** 56
^{13}C-NMR (101 MHz, $CDCl_3$) for compound **3q** 57
^{1}H-NMR (400 MHz, $CDCl_3$) for compound **3r** 58
^{13}C-NMR (101 MHz, $CDCl_3$) for compound **3r** 59
^{1}H-NMR (400 MHz, $CDCl_3$) for compound **3s** 60
^{13}C-NMR (101 MHz, $CDCl_3$) for compound **3s** 61
^{1}H-NMR (400 MHz, $CDCl_3$) for compound **3t** 62
^{13}C-NMR (101 MHz, $CDCl_3$) for compound **3t** 63
^{1}H-NMR (400 MHz, $CDCl_3$) for compound **3u** 64
^{13}C-NMR (101 MHz, $CDCl_3$) for compound **3u** 65
^{1}H-NMR (400 MHz, $CDCl_3$) for compound **3v** 66
^{13}C-NMR (101 MHz, $CDCl_3$) for compound **3v** 67
^{1}H-NMR (400 MHz, $CDCl_3$) for compound **3x’** 68
^{13}C-NMR (101 MHz, $CDCl_3$) for compound **3x’** 69
^{1}H-NMR (400 MHz, $CDCl_3$) for compound **3x** 70
^{13}C-NMR (101 MHz, $CDCl_3$) for compound **3x** 71
^{1}H-NMR (400 MHz, $CDCl_3$) for compound **9a** 72
^{13}C-NMR (101 MHz, $CDCl_3$) for compound **9a** 73
^{1}H-NMR (400 MHz, $CDCl_3$) for compound **9b** 74
^{13}C-NMR (101 MHz, $CDCl_3$) for compound **9b** 75
^{1}H-NMR (400 MHz, $CDCl_3$) for compound **9c** 76
^{13}C-NMR (101 MHz, $CDCl_3$) for compound **9c** 77
^{1}H-NMR (400 MHz, $CDCl_3$) for compound **9d** 78
^{13}C-NMR (101 MHz, $CDCl_3$) for compound **9d** 79
^{1}H-NMR (400 MHz, $CDCl_3$) for compound **9e** 80
^{13}C-NMR (101 MHz, $CDCl_3$) for compound **9e** 81
^{1}H-NMR (400 MHz, $CDCl_3$) for compound **9f** 82
^{13}C-NMR (101 MHz, $CDCl_3$) for compound **9f** 83

^{1}H-NMR (400 MHz, $CDCl_3$) for compound **9g** 84
^{13}C-NMR (101 MHz, $CDCl_3$) for compound **9g** 85
^{1}H-NMR (400 MHz, $CDCl_3$) for compound **9h** 86
^{13}C-NMR (101 MHz, $CDCl_3$) for compound **9h** 87
^{1}H-NMR (400 MHz, $CDCl_3$) for compound **9i** 88
^{13}C-NMR (101 MHz, $CDCl_3$) for compound **9i** 89
^{1}H-NMR (400 MHz, $CDCl_3$) for compound **9j** 90
^{13}C-NMR (101 MHz, $CDCl_3$) for compound **9j** 91
^{19}F-NMR (377 MHz, $CDCl_3$) for compound **9j** 92
^{1}H-NMR (400 MHz, $CDCl_3$) for compound **9k** 93
^{13}C-NMR (101 MHz, $CDCl_3$) for compound **9k** 94
^{1}H-NMR (400 MHz, $CDCl_3$) for compound **9l** 95
^{13}C-NMR (101 MHz, $CDCl_3$) for compound **9l** 96
^{1}H-NMR (400 MHz, $CDCl_3$) for compound **9m** 97
^{13}C-NMR (101 MHz, $CDCl_3$) for compound **9m** 98
^{1}H-NMR (400 MHz, $CDCl_3$) for compound **9n** 99
^{13}C-NMR (101 MHz, $CDCl_3$) for compound **9n** 100
^{1}H-NMR (400 MHz, $CDCl_3$) for compound **9o** 101
^{13}C-NMR (101 MHz, $CDCl_3$) for compound **9o** 102
^{1}H-NMR (400 MHz, $CDCl_3$) for compound **9p** 103
^{13}C-NMR (101 MHz, $CDCl_3$) for compound **9p** 104
^{1}H-NMR (400 MHz, $CDCl_3$) for compound **9q** 105
^{13}C-NMR (101 MHz, $CDCl_3$) for compound **9q** 106
^{1}H-NMR (400 MHz, $CDCl_3$) for compound **9r** 107
^{13}C-NMR (101 MHz, $CDCl_3$) for compound **9r** 108
^{1}H-NMR (400 MHz, $CDCl_3$) for compound **9s** 109
^{13}C-NMR (101 MHz, $CDCl_3$) for compound **9s** 110
^{1}H-NMR (400 MHz, $CDCl_3$) for compound **9t** 111
^{13}C-NMR (101 MHz, $CDCl_3$) for compound **9t** 112
^{1}H-NMR (400 MHz, $CDCl_3$) for compound **9u** 113
^{13}C-NMR (101 MHz, $CDCl_3$) for compound **9u** 114
^{1}H-NMR (400 MHz, $CDCl_3$) for compound **9v** 115
^{13}C-NMR (101 MHz, $CDCl_3$) for compound **9v** 116
^{1}H-NMR (400 MHz, $CDCl_3$) for compound **9w** 117
^{13}C-NMR (101 MHz, $CDCl_3$) for compound **9w** 118
^{1}H-NMR (400 MHz, $CDCl_3$) for compound **9x** 119

^{13}C-NMR (101 MHz, $CDCl_3$) for compound **9x** 120
^{1}H-NMR (400 MHz, $CDCl_3$) for compound **9y** 121
^{13}C-NMR (101 MHz, $CDCl_3$) for compound **9y** 122
^{1}H-NMR (400 MHz, $CDCl_3$) for compound **9z** 123
^{13}C-NMR (101 MHz, $CDCl_3$) for compound **9z** 124
^{1}H-NMR (400 MHz, $CDCl_3$) for compound **9aa** 125
^{13}C-NMR (401 MHz, $CDCl_3$) for compound **9aa** 126
^{1}H-NMR (400 MHz, $CDCl_3$) for compound **1a** 127
^{13}C-NMR (101 MHz, $CDCl_3$) for compound **1a** 128
^{1}H-NMR (400 MHz, $CDCl_3$) for compound **1b** 129
^{13}C-NMR (101 MHz, $CDCl_3$) for compound **1b** 130
^{1}H-NMR (400 MHz, $CDCl_3$) for compound **1c** 131
^{13}C-NMR (101 MHz, $CDCl_3$) for compound **1c** 132
^{1}H-NMR (400 MHz, $CDCl_3$) for compound **1d** 133
^{13}C-NMR (101 MHz, $CDCl_3$) for compound **1d** 134
^{1}H-NMR (400 MHz, $CDCl_3$) for compound **1e** 135
^{13}C-NMR (101 MHz, $CDCl_3$) for compound **1e** 136
^{1}H-NMR (400 MHz, $CDCl_3$) for compound **1f** 137
^{13}C-NMR (101 MHz, $CDCl_3$) for compound **1f** 138
^{1}H-NMR (400 MHz, $CDCl_3$) for compound **1g** 139
^{13}C-NMR (101 MHz, $CDCl_3$) for compound **1g** 140
^{1}H-NMR (400 MHz, $CDCl_3$) for compound **1h** 141
^{13}C-NMR (101 MHz, $CDCl_3$) for compound **1h** 142
^{1}H-NMR (400 MHz, $CDCl_3$) for compound **1i** 143
^{13}C-NMR (101 MHz, $CDCl_3$) for compound **1i** 144
^{1}H-NMR (400 MHz, $CDCl_3$) for compound **1j** 145
^{13}C-NMR (101 MHz, $CDCl_3$) for compound **1j** 146
^{1}H-NMR (400 MHz, $CDCl_3$) for compound **1k** 147
^{13}C-NMR (101 MHz, $CDCl_3$) for compound **1k** 148
^{19}F-NMR (377 MHz, $CDCl_3$) for compound **1k** 149
^{1}H-NMR (400 MHz, $CDCl_3$) for compound **1l** 150
^{13}C-NMR (101 MHz, $CDCl_3$) for compound **1l** 151
^{1}H-NMR (400 MHz, $CDCl_3$) for compound **1m** 152
^{13}C-NMR (101 MHz, $CDCl_3$) for compound **1m** 153
^{1}H-NMR (400 MHz, $CDCl_3$) for compound **1n** 154
^{13}C-NMR (101 MHz, $CDCl_3$) for compound **1n** 155

^{1}H-NMR (400 MHz, $CDCl_3$) for compound **1o** 156
^{13}C-NMR (101 MHz, $CDCl_3$) for compound **1o** 157
^{1}H-NMR (400 MHz, $CDCl_3$) for compound **1p** 158
^{13}C-NMR (101 MHz, $CDCl_3$) for compound **1p** 159
^{1}H-NMR (400 MHz, $CDCl_3$) for compound **1q** 160
^{13}C-NMR (101 MHz, $CDCl_3$) for compound **1q** 161
^{1}H-NMR (400 MHz, $CDCl_3$) for compound **1r** 162
^{13}C-NMR (101 MHz, $CDCl_3$) for compound **1r** 163
HSQC-NMR ($CDCl_3$) for compound **1r** 164
^{1}H-NMR (400 MHz, $CDCl_3$) for compound **1s** 165
^{13}C-NMR (101 MHz, $CDCl_3$) for compound **1s** 166
^{1}H-NMR (400 MHz, $CDCl_3$) for compound **1t** 167
^{13}C-NMR (101 MHz, $CDCl_3$) for compound **1t** 168
^{1}H-NMR (400 MHz, $CDCl_3$) for compound **1u** 169
^{13}C-NMR (101 MHz, $CDCl_3$) for compound **1u** 170
^{1}H-NMR (400 MHz, $CDCl_3$) for compound **1v** 171
^{13}C-NMR (101 MHz, $CDCl_3$) for compound **1v** 172

1. General Information

Materials: Silanes were either synthesised using the procedures described in the literature[1-6] or purchased from commercial sources, e.g.; Sigma Aldrich, Fluorochem and TCI Chemicals etc. NHPI-DA (**8**) was prepared according to the procedure developed by our group.[12] Alternatively, it can be purchased from Key Organics (CAS: 816437-80-6; Product Number: SO-3001). Catalysts, Ru(Me_2-Pheox) and Fe[TPP]Cl were synthesized using the procedures described in the literature,[13] Other catalysts and ligands used in this work, were purchased from aforementioned commercial sources. Dry solvents were obtained by passing them through activated alumina columns. When appropriate, degassing of anhydrous solvent was performed by bubbling argon for 30 minutes under sonication. The solvents used in column chromatography, petroleum ether, pentane, dichloromethane, methanol and ethyl acetate, were purchased from commercial suppliers in HPLC grade and used without further purification.

Chromatography: Thin layer chromatography (TLC) was carried out on 0.25 mm E. Merck silica plates (60F – 254) using UV light (λ = 254 nm) as visualizing agent and vanillin, phosphomolybdic acid (PMA) or $KMnO_4$ solution and heat as developing agents, as specified. Flash column chromatography on SiO_2 was performed using E. Merck silica oil (60 Å, particle size 0.043–0.063mm).

Characterization: NMR spectra for characterization of compounds were recorded at room temperature on a Bruker instrument 400 MHz (^{1}H) and at 101 MHz (^{13}C) and 377 MHz (^{19}F), or 500 MHz (^{1}H) and at 126 MHz (^{13}C). Chemical shifts (δ) are reported in ppm, using the residual solvent peak in $CDCl_3$ (δ_H = 7.26 and δ_C = 77.16 ppm), or the trifluorotoluene signal as external reference (δ_F = –62.74 ppm). Coupling constants (*J*) are given in hertz (Hz). Data are reported as follows: chemical shift, multiplicity (s: singlet, d: doublet, t: triplet, q: quartet, p: pentet, dp: double pentet, ddp: doublet of double pentets, h: hextet, hept: heptet, br: broad, m: multiplet), coupling constants and integration.

High-resolution mass spectra (HRMS) were determined with a Bruker Daltonics microTOF Mass Spectrometer using an ESI ion source and acetonitrile as mobile phase. 1 µM *i*-PrOH solution of KOH as additive was helpful for the ionization of compounds (**9n**,**z**). For compounds **3d,e,f,j,l,m,q,r,t** neither ESI-TOF, APCI-TOF ion source proved suitable to provide high-precision data; low-resolution mass spectra (LRMS) recorded on a Shimadzu GC-MS (EI) are reported instead. Melting points (m.p.) of solid samples were measured by a melting-point apparatus (Stuart SMP 50).

Experimental details: Reactions were performed in common pyrex round bottom flasks, microwave vials 2 - 8 mL (VWR or Biotage®), or 5 - 20 ml flat bottom vials (Cronus, SMILabHut Ltd. or VWR®) crimped on top with 20 mm Sil/PTFE Septa. Reaction temperatures were maintained using Thermowatch-controlled silicone oil baths, dry ice-acetone, water-ice baths or an Immersion Cooler (Julabo-FT902) equipped with temperature controller.

2. Preparation of Starting Materials

The following silanes were synthesized according to the procedures reported in the literature. All the data are in accordance with the literature.[1-7]

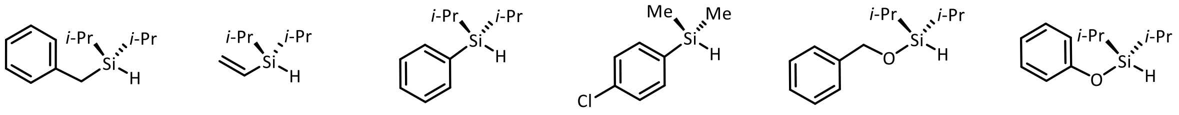

3c **3g** **3h** **3k** **3p** **3w**

Figure 2.1: Silanes prepared according to the procedures reported in the literature.

2.1 General procedure A: Preparation of Grignard reagents

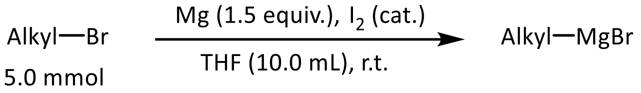

A flame-dried microwave vial was charged with Mg turnings (0.18 g, 7.5 mmol, 1.5 equiv.) and I_2 (13 mg, 0.05 mmol, 0.01 equiv.). In a separate flame-dried vial, alkyl bromide (5.0 mmol, 1.0 equiv.) was dissolved in anhydrous THF (10.0 mL) to make a 0.5 M solution. A small portion of the alkyl bromide solution (*ca.* 2.0 mL) was added to the mixture of Mg and I_2. The vial was stirred while heated gently with a heat gun until the dark brown colour disappeared. The rest of the alkyl bromide solution (8.0 mL) was added dropwise while the vial was heated with a heat gun. After 1 h, the resulting Grignard reagent was titrated using the protocol reported by Knochel and co-workers.[1]

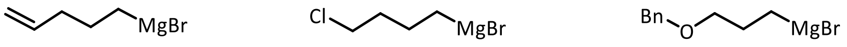

S2 (0.44 M) **S3** (0.49 M) **S4** (0.31 M)

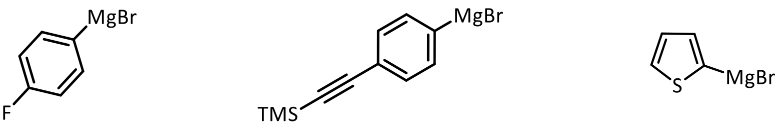

S5 (0.81 M) **S6** (0.36M) **S7** (0.40 M)

Scheme 2.1: Freshly prepared Grignard reagents.

2.2 General procedure B: Reaction of the freshly prepared Grignard reagents (using general procedure A) with chlorodiisopropylsilane

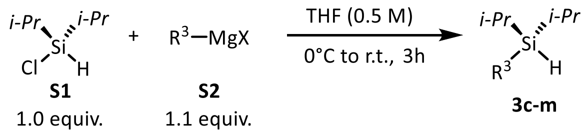

A freshly prepared (using general procedure A) or a commercially available Grignard reagent (1.1 equiv.) was added dropwise to the ice-bath cooled THF solution of

chlorodiisopropylsilane (**S1**; 1 equiv.; 0.5 M). After 3 hours of stirring the mixture was quenched with water and the aqueous layer was extracted with diethyl ether (3 x 10 mL). The combined organic layers were dried over Na_2SO_4 and the solvent was removed under reduced pressure. The crude was purified by flash chromatography on SiO_2 using pentane or pentane/EtOAc as eluent to afford silanes **3c-m**.

2.3 General procedure C: Synthesis of alkoxysilanes using DMAP and triethylamine

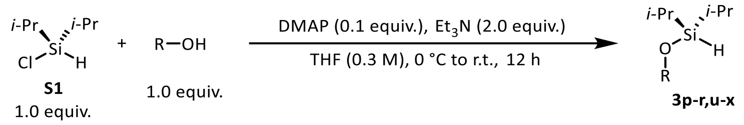

In a 50 mL round-bottomed flask chlorodiisopropylsilane (**S1**; 1.0 equiv.) was added to a mixture of the corresponding alcohol (1.0 equiv.) and DMAP (0.1 equiv.) in tetrahydrofuran (0.3 M) at 0 °C under argon atmosphere. Triethylamine (2.0 equiv.) was added dropwise and the reaction mixture was stirred for 12 h at room temperature. Upon completion, the white solid was filtered off and the filtrate was concentrated under reduced pressure. The crude was purified by flash chromatography on SiO_2 (pentane/EtOAc = 98:2) to afford alkoxysilanes **3p-r,u-x**.

2.4 General procedure D: Synthesis of alkoxysilanes using imidazole

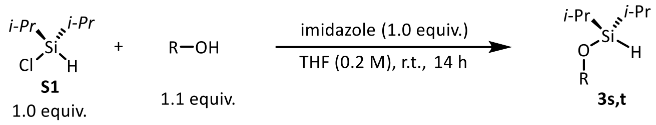

In a 50 mL round-bottomed flask, chlorodiisopropylsilane (**S1**; 1.0 equiv.) was added to a mixture of an alcohol (1.1 equiv.) and imidazole (1.0 equiv.) in tetrahydrofuran (0.2 M) at room temperature under argon atmosphere. The resulting reaction mixture was stirred for 14 h. Upon completion, the white solid was filtered off and the filtrate was concentrated under reduced pressure. The crude was purified by flash chromatography on SiO_2 (pentane/EtOAc = 98:2) to afford alkoxysilanes **3s,t.**

2.5 Synthesis of silane materials

*Synthesis of diisopropyl(pent-4-en-1-yl)silane (**3d**)*

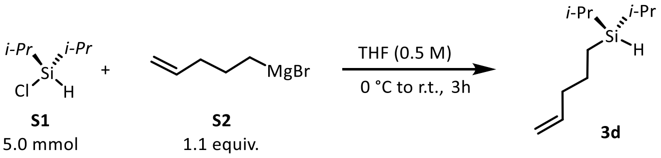

General procedure B was applied using chlorodiisopropylsilane (**S1**; 0.9 mL, 5 mmol, 1 equiv.), pent-4-en-1-ylmagnesium bromide (**S2**; 12.5 mL, 0.44 M in THF, 3.3 mmol, 1.1 equiv.), at 0 ˚C to room temperature for 3 h under argon. The crude was purified by flash chromatography on SiO_2 (pentane/EtOAc = 99:1 to 98:2) to afford compound **2d** (0.46 g, 2.5 mmol, 50%).

Appearance: colourless oil.

TLC: R_f = 0.6 (pentane/EtOAc = 98:2, stains in $KMnO_4$).

^{1}H-NMR (400 MHz, $CDCl_3$) δ (ppm) = 5.80 (ddt, *J* = 17.0, 10.2, 6.7 Hz, 1H), 5.05 – 4.93 (m, 2H), 3.44 – 3.40 (m, 1H), 2.13 – 2.05 (m, 2H), 1.53 – 1.44 (m, 2H), 1.04 – 1.01 (m, 14H), 0.62 (ddd, *J* = 11.3, 5.8, 3.2 Hz, 2H).

^{13}C-NMR (101 MHz, $CDCl_3$) δ (ppm) = 138.8, 114.5, 37.6, 24.8, 19.1, 18.7, 10.6, 8.0.

GC-MS [$C_{11}H_{24}Si$] (m/*z*) = 141 ($C_8H_{17}Si$), 113 ($C_6H_{13}Si$), 99 ($C_5H_{11}Si$), 85 (C_4H_9Si), 71 (C_3H_7Si).
***Due to poor ionization either by ESI-TOF or APCI-TOF HRMS for this compound could not be acquired.*

*Synthesis of (4-chlorobutyl)diisopropylsilane (**3e**)*

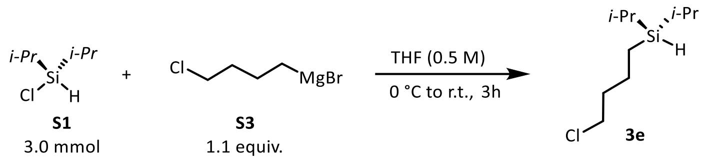

General procedure B was applied using chlorodiisopropylsilane (**S1**; 0.5 mL, 3 mmol, 1 equiv.), (4-chlorobutyl)magnesium bromide (**S3**; 6.7 mL, 0.49 M in THF, 3.3 mmol, 1.1 equiv.), at 0 ˚C to room temperature for 3 h under argon. The crude was purified by flash chromatography on SiO_2 (pentane/EtOAc = 99:1 to 98:2) to afford compound **3e** (0.18 g, 0.87 mmol, 29%).

Appearance: colourless oil.

TLC: R_f = 0.6 (pentane/EtOAc = 98:2, stains in $KMnO_4$).

^{1}H-NMR (400 MHz, $CDCl_3$) δ (ppm) = 3.55 (t, *J* = 6.6 Hz, 2H), 3.46 – 3.41 (m, 1H), 1.82 (dt, *J* = 14.2, 6.8 Hz, 2H), 1.60 – 1.49 (m, 2H), 1.04 – 1.02 (m, 12H), 0.90 – 0.82 (m, 2H), 0.68 – 0.58 (m, 2H).

^{13}C-NMR (101 MHz, $CDCl_3$) δ (ppm) = 44.7, 36.1, 22.6, 19.1, 18.7, 10.5, 7.6.

GC-MS [$C_{10}H_{23}ClSi$] (m/*z*) = 163 ($C_7H_{16}ClSi$), 135 ($C_5H_{12}ClSi$), 121 ($C_4H_{10}ClSi$).
***Due to poor ionization either by ESI-TOF or APCI-TOF HRMS for this compound could not be acquired.*

*Synthesis of (3-(benzyloxy)propyl)diisopropylsilane (**3f**)*

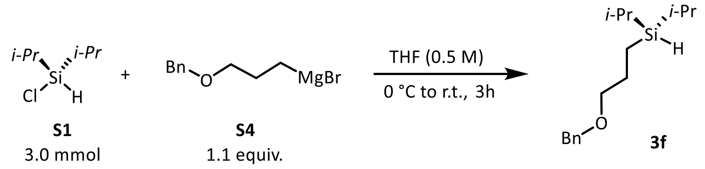

General procedure B was applied using chlorodiisopropylsilane (**S1**; 0.5 mL, 3 mmol, 1 equiv.), (3-(benzyloxy)propyl)magnesium bromide (**S4**; 10.6 mL, 0.31 M in THF, 3.3 mmol, 1.1 equiv.), at 0 ˚C to room temperature for 3 h under argon. The crude was purified by flash chromatography on SiO_2 (pentane/EtOAc = 99:1 to 98:2) to afford compound **3f** (0.75 g, 2.5 mmol, 84%).

Appearance: colourless oil.

TLC: R_f = 0.6 (pentane/EtOAc = 98:2, UV-active and stains in $KMnO_4$).

^{1}H-NMR (400 MHz, $CDCl_3$) δ (ppm) = 7.35 (d, *J* = 4.3 Hz, 4H), 7.32 – 7.26 (m, 1H), 4.52 (s, 2H), 3.46 (t, *J* = 6.8 Hz, 3H), 1.72 (dt, *J* = 16.1, 6.9 Hz, 2H), 1.03 (d, *J* = 4.1 Hz, 14H), 0.71 – 0.57 (m, 2H).

^{13}C-NMR (101 MHz, $CDCl_3$) δ (ppm) = 138.7, 128.3, 127.6, 127.5, 73.2, 72.8, 25.4, 19.1, 18.7, 10.6, 4.5.

GC-MS [$C_{16}H_{28}OSi$] (m/*z*) = 264 ($C_6H_{28}OSi$), 91 (C_7H_7).
***Due to poor ionization either by ESI-TOF or APCI-TOF HRMS for this compound could not be acquired*

*Synthesis of (4-fluorophenyl)diisopropylsilane (**3j**)*

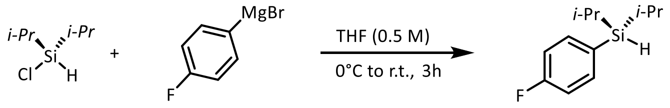

S1 3.0 mmol; **S5** 1.1 equiv.; **3j**

General procedure B was applied using chlorodiisopropylsilane (**S1**; 0.5 mL, 3 mmol, 1 equiv.), (4-fluorophenyl)magnesium bromide (**S5**; 4.1 mL, 0.81 M in THF, 3.3 mmol, 1.1 equiv.), at 0 ˚C to room temperature for 3 h under argon. The crude was purified by flash chromatography on SiO_2 (pentane/EtOAc = 99:1 to 98:2) to afford compound **3j** (0.50 g, 2.3 mmol, 79%).

Appearance: colourless oil.

TLC: R_f = 0.4 (pentane; UV-active and stains in iodine).

^{1}H-NMR (400 MHz, $CDCl_3$) δ (ppm) = 7.52 – 7.45 (m, 2H), 7.09 – 7.02 (m, 2H), 3.94 (t, *J* = 3.1 Hz, 1H), 1.25 – 1.15 (m, 2H), 1.06 (d, *J* = 7.2 Hz, 6H), 0.98 (d, *J* = 7.3 Hz, 6H).

^{13}C-NMR (101 MHz, $CDCl_3$) δ (ppm) = 165.1 (d, $J_{C\text{-}F}$ = 241.9 Hz), 137.3 (d, $J_{C\text{-}F}$ = 7.5 Hz), 129.5 (d, $J_{C\text{-}F}$ = 4.0 Hz), 115.0 (d, $J_{C\text{-}F}$ = 19.8 Hz), 18.6, 18.4, 10.7.

^{19}F-NMR (377 MHz) δ (ppm) = –111.95.

GC-MS [$C_{12}H_{19}FSi$] (m/*z*) = 210 ($C_{12}H_{19}FSi$), 167 ($C_9H_{12}FSi$), 125 (C_6H_6FSi), 139 (C_7H_8FSi).
***Due to poor ionization either by ESI-TOF or APCI-TOF HRMS for this compound could not be acquired.*

*Synthesis of ((4-(diisopropylsilyl)phenyl)ethynyl)trimethylsilane (**3l**)*

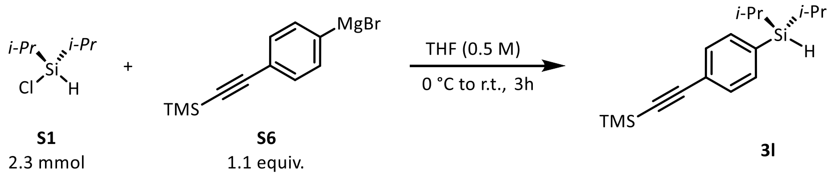

S1 2.3 mmol; **S6** 1.1 equiv.; **3l**

General procedure B was applied using chlorodiisopropylsilane (**S1**; 0.39 mL, 2.3 mmol, 1.0 equiv.), (4-((trimethylsilyl)ethynyl)phenyl)magnesium bromide (**S6**; 7.1 mL, 0.36 M in THF,

2.5 mmol, 1.1 equiv.), at 0 ˚C to room temperature for 3 h under argon. The crude was purified by flash chromatography on SiO_2 (pentane/EtOAc = 99:1 to 98:2) to afford compound **3l** (0.52 g, 1.8 mmol, 78%).

Appearance: Colourless oil.

TLC: R_f = 0.75 (pentane; UV-active and stains green in vanillin).

^{1}H-NMR (400 MHz, $CDCl_3$) δ (ppm) = 7.44 (s, 4H), 3.92 (t, *J* = 3.2 Hz, 1H), 1.22 (ddp, *J* = 10.5, 7.4, 3.2 Hz, 2H), 1.05 (d, *J* = 7.3 Hz, 6H), 0.97 (d, *J* = 7.3 Hz, 6H), 0.25 (s, 9H).

^{13}C-NMR (101 MHz, $CDCl_3$) δ (ppm) = 135.2, 135.1, 130.9, 123.7, 105.1, 94.9, 18.6, 18.4, 10.6, -0.0.

GC-MS [$C_{17}H_{28}Si_2$] (m/*z*) = 288 ($C_{17}H_{28}Si_2$), 273 ($C_{16}H_{25}Si_2$), 245 ($C_{14}H_{21}Si_2$), 217 ($C_{12}H_{17}Si_2$).
***Due to poor ionization either by ESI-TOF or APCI-TOF HRMS for this compound could not be acquired.*

*Synthesis of diisopropyl(thiophen-2-yl)silane (**3m**)*

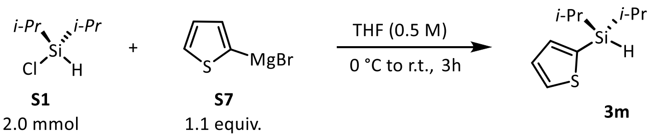

General procedure B was applied using chlorodiisopropylsilane (**S1**; 0.33 mL, 2.0 mmol, 1.0 equiv.), thiophen-2-ylmagnesium bromide (**S7**; 5.5 mL, 0.4 M in THF, 2.2 mmol, 1.1 equiv.), at 0 ˚C to room temperature for 3 h under argon. The crude was purified by flash chromatography on SiO_2 (pentane) to afford compound **3m** (0.34 g, 1.7 mmol, 87%).

Appearance: Colorless oil.

TLC: R_f = 0.81 (pentane, UV-active and stains in iodine).

^{1}H-NMR (400 MHz, $CDCl_3$) δ (ppm) = 7.64 (d, *J* = 4.6 Hz, 1H), 7.33 (d, *J* = 3.4 Hz, 1H), 7.22 (dd, *J* = 4.6, 3.4 Hz, 1H), 4.12 (t, *J* = 2.9 Hz, 1H), 1.28 – 1.17 (m, 2H), 1.06 (dd, *J* = 15.0, 7.2 Hz, 12H).

^{13}C-NMR (101 MHz, $CDCl_3$) δ (ppm) = 136.6, 131.7, 131.2, 128.2, 18.7, 18.5, 11.5.

GC-MS [$C_{10}H_{18}SSi$] (m/*z*) = 198 ($C_{10}H_{18}SSi$), 155 ($C_7H_{11}SSi$), 127 (C_5H_7SSi), 112 (C_4H_4SSi), 101 ($C_5H_{13}Si$).
***Due to poor ionization either by ESI-TOF or APCI-TOF HRMS for this compound could not be acquired.*

*Synthesis of (cyclohex-2-en-1-yloxy)diisopropylsilane (**3q**)*

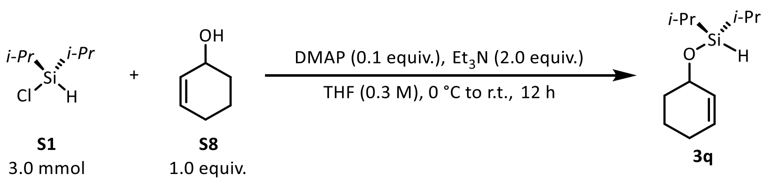

General procedure C was applied using chlorodiisopropylsilane (**S1**; 0.51 mL, 3.0 mmol, 1.0 equiv.), cyclohex-2-en-1-ol (**S8**; 0.29 mL, 3.0 mmol, 1.0 equiv.), DMAP (37 mg, 0.30 mmol, 0.10 equiv.) and triethylamine (0.83 mL, 6.0 mmol, 2.0 equiv.) in THF (0.3 M) at room temperature for 12 h under argon. The crude was purified by flash chromatography on SiO_2 (pentane/EtOAc = 99:1 to 98:2) to afford compound **3q** (0.48 g, 2.3 mmol, 75%).

Appearance: Colourless oil.

TLC: R_f = 0.55 (pentane/EtOAc = 98:2; stains in $KMnO_4$).

^{1}H-NMR (400 MHz, $CDCl_3$) δ (ppm) = 5.78 (dtd, *J* = 6.8, 3.4, 1.0 Hz, 1H), 5.75 – 5.68 (m, 1H), 4.27 – 4.21 (m, 1H), 4.19 (t, *J* = 1.7 Hz, 1H), 2.09 – 1.98 (m, 1H), 1.98 – 1.82 (m, 2H), 1.82 – 1.73 (m, 1H), 1.67 – 1.49 (m, 2H), 1.08 – 0.94 (m, 14H).

^{13}C-NMR (101 MHz, $CDCl_3$) δ (ppm) = 130.4, 129.5, 68.9, 32.0, 25.0, 19.4, 17.5, 17.44, 17.43, 17.37, 12.7, 12.5.

GC-MS [$C_{12}H_{24}OSi$] (m/*z*) = 212 ($C_{12}H_{24}OSi$), 169 ($C_9H_{17}OSi$), 89 (C_3H_9OSi), 81 (C_6H_9).
***Due to poor ionization either by ESI-TOF or APCI-TOF HRMS for this compound could not be acquired.*

*Synthesis of diisopropyl((4-methylpentan-2-yl)oxy)silane (**3r**)*

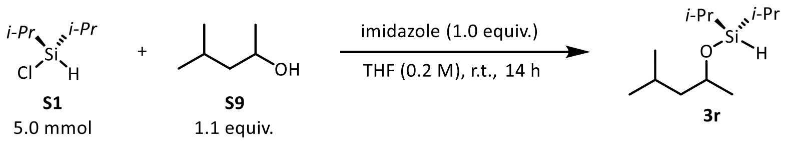

General procedure D was applied using chlorodiisopropylsilane (**S1**; 0.85 mL, 5.0 mmol, 1.0 equiv.), 4-methylpentan-2-ol (**S9**; 0.70 mL, 5.5 mmol, 1.1 equiv.) and imidazole (0.34 g, 5.0 mmol, 1.0 equiv.) in THF (0.2 M) at room temperature for 14 h under argon. The crude was purified by flash chromatography on SiO_2 (pentane/EtOAc = 99:1 to 98:2) to afford compound **3r** (0.49 g, 2.2 mmol, 45%).

Appearance: Colourless oil.

TLC: R_f = 0.75 (pentane; stains in iodine).

^{1}H-NMR (400 MHz, $CDCl_3$) δ (ppm) = 4.18 (t, *J* = 1.6 Hz, 1H), 3.95 – 3.84 (m, 1H), 1.78 – 1.65 (m, 1H), 1.45 (ddd, *J* = 13.7, 7.4, 6.4 Hz, 1H), 1.24 – 1.18 (m, 1H), 1.16 (d, *J* = 6.1 Hz, 3H), 1.08 – 1.00 (m, 12H), 1.00 – 0.94 (m, 2H), 0.89 (dd, *J* = 6.6, 5.4 Hz, 6H).

^{13}C-NMR (101 MHz, $CDCl_3$) δ (ppm) = 69.1, 49.0, 24.6, 23.6, 23.0, 22.6, 17.57, 17.51, 17.46, 17.40, 12.7, 12.6.

GC-MS [$C_{12}H_{28}OSi$] (m/*z*) = 216 ($C_{12}H_{28}OSi$), 201 ($C_{11}H_{25}OSi$), 173 ($C_9H_{21}OSi$), 159 ($C_8H_{19}OSi$), 131 ($C_6H_{15}OSi$).
***Due to poor ionization either by ESI-TOF or APCI-TOF HRMS for this compound could not be acquired.*

*Synthesis of diisopropyl(((1S,2R,5S)-2-isopropyl-5-methylcyclohexyl)oxy)silane (**3s**)*

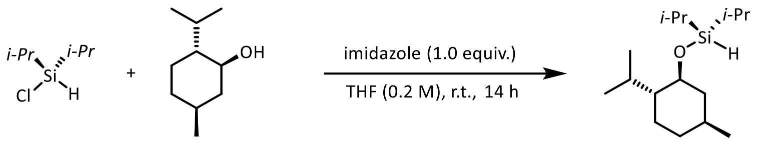

S1
2.0 mmol

S10
1.1 equiv.

3s

General procedure D was applied using chlorodiisopropylsilane (**S1**; 0.34 mL, 2.0 mmol, 1.0 equiv.), (1*S*,2*R*,5*S*)-2-isopropyl-5-methylcyclohexan-1-ol (**S10**; 0.65 g, 4.2 mmol, 1.1 equiv.) and imidazole (0.28 g, 4.2 mmol, 1.0 equiv.) in THF (0.2 M) at room temperature for 14 h under argon. The crude was purified by flash chromatography on SiO_2 (pentane/EtOAc = 99:1 to 98:2) to afford compound **3s** (0.31 g, 1.2 mmol, 59%).

Appearance: Colourless oil.

TLC: R_f = 0.75 (pentane; UV-active and stains green in vanillin).

^{1}H-NMR (400 MHz, $CDCl_3$) δ (ppm) = 4.22 (t, *J* = 1.5 Hz, 1H), 3.44 (td, *J* = 10.3, 4.3 Hz, 1H), 2.31 – 2.20 (m, 1H), 2.01 – 1.94 (m, 1H), 1.65 – 1.56 (m, 2H), 1.42 – 1.31 (m, 1H), 1.20 – 1.12 (m, 1H), 1.06 – 1.02 (m, 12H), 1.01 – 0.92 (m, 4H), 0.90 (dd, *J* = 6.8, 1.6 Hz, 6H), 0.88 – 0.80 (m, 1H), 0.75 (d, *J* = 7.0 Hz, 3H).

^{13}C-NMR (101 MHz, $CDCl_3$) δ (ppm) = 74.5, 50.5, 44.9, 34.7, 31.8, 25.2, 23.0, 22.5, 21.4, 17.81, 17.76, 17.69, 17.67, 16.0, 13.1, 12.9.

HRMS (ESI-TOF) m/*z*: $[M+Na]^+$ Calcd for $C_{16}H_{34}OSiNa$ 293.2271; Found 293.2273.

*Synthesis of ethyl (R)-2-((diisopropylsilyl)oxy)propanoate (**3t**)*

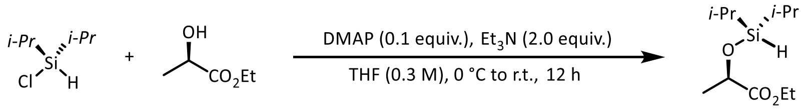

S1
3.0 mmol

S11
1.0 equiv.

3t

General procedure C was applied using chlorodiisopropylsilane (**S1**: 0.68 mL, 4.0 mmol, 1.0 equiv.), ethyl (*R*)-2-hydroxypropanoate (**S11**; 0.46 mL, 4.2 mmol, 1.0 equiv.), DMAP (49 mg, 40 µmol, 0.1 equiv.) and triethylamine (1.1 mL, 8.0 mmol, 2.0 equiv.) in THF (0.3 M) at room temperature for 12 h under argon. The crude was purified by flash chromatography on SiO_2 (pentane/EtOAc = 99:1 to 98:2) to afford compound **3t** (0.40 g, 1.7 mmol, 43%).

Appearance: Colourless oil.

TLC: R_f = 0.55 (pentane/EtOAc = 98:2; stains in $KMnO_4$).

^{1}H-NMR (400 MHz, $CDCl_3$) δ (ppm) = 4.34 (q, *J* = 6.8 Hz, 1H), 4.26 – 4.13 (m, 3H), 1.43 (d, *J* = 6.8 Hz, 3H), 1.28 (t, *J* = 7.1 Hz, 3H), 1.08 – 0.99 (m, 14H).

^{13}C-NMR (101 MHz, $CDCl_3$) δ (ppm) = 173.7, 70.5, 60.8, 21.0, 17.32, 17.27, 17.15, 14.2, 12.5, 12.4.

GC-MS [$C_{11}H_{24}O_3Si$] (m/*z*) = 189 ($C_8H_{17}O_3Si$), 161 ($C_6H_{13}O_3Si$), 133 ($C_6H_{17}OSi$), 117 ($C_5H_9O_3$), 108 (C_7H_8O).

***Due to poor ionization either by ESI-TOF or APCI-TOF HRMS for this compound could not be acquired.*

*Synthesis of methyl N-(tert-butoxycarbonyl)-O-(diisopropylsilyl)-D-serinate (**3u**)*

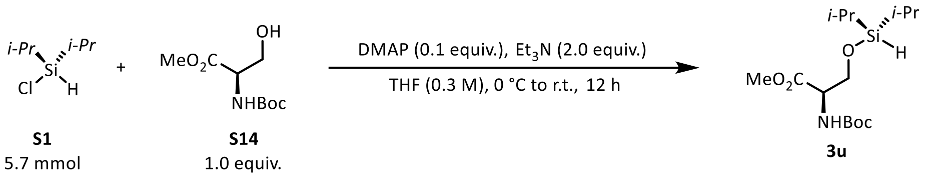

General procedure C was applied using chlorodiisopropylsilane (**S1**; 0.97 mL, 5.7 mmol, 1.0 equiv.), methyl (*tert*-butoxycarbonyl)-*D*-serinate (**S14**; 1.2 g, 5.7 mmol, 1.0 equiv.), DMAP (70 mg, 0.57 mmol, 0.10 equiv.) and triethylamine (1.6 mL, 11.4 mmol, 2.0 equiv.) in THF (0.3 M) at room temperature for 12 h under argon. The crude was purified by flash chromatography on SiO_2 (pentane/EtOAc = 99:1 to 98:2) to afford compound **3u** (0.85 g, 2.5 mmol, 45%).

Appearance: Colourless oil.

TLC: R*f* = 0.6 (pentane/EtOAc = 95:5; stains in $KMnO_4$).

^{1}H-NMR (400 MHz, $CDCl_3$) δ (ppm) = 5.35 (d, *J* = 8.2 Hz, 1H), 4.43 – 4.32 (m, 1H), 4.15 – 4.08 (m, 2H), 3.93 (dd, *J* = 10.1, 3.0 Hz, 1H), 3.75 (s, 3H), 1.45 (s, 9H), 1.03 – 0.97 (m, 14H).

^{13}C-NMR (101 MHz, $CDCl_3$) δ (ppm) = 171.1, 155.4, 79.9, 66.0, 55.6, 52.3, 28.3, 17.3, 17.2, 17.12, 17.08, 12.4, 12.2.

HRMS (ESI-TOF) m/*z*: $[M+Na]^+$ Calcd for $C_{15}H_{31}NO_5SiNa$ 356.1864; Found 356.1864.

*Synthesis of (E)-((3,7-dimethylocta-2,6-dien-1-yl)oxy)diisopropylsilane (**3v**)*

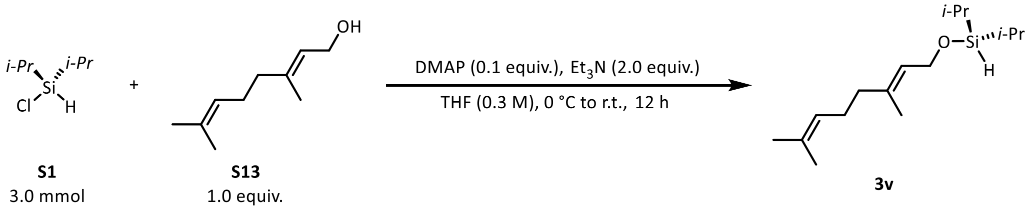

General procedure C was applied using chlorodiisopropylsilane (**S1**; 0.51 mL, 3.0 mmol, 1.0 equiv.), geraniol (**S13**; 0.52 mL, 3.0 mmol, 1.0 equiv.), DMAP (37 mg, 0.30 mmol, 0.10 equiv.) and triethylamine (0.84 mL, 6.0 mmol, 2.0 equiv.) in THF (0.3 M) at room temperature for 12 h under argon. The crude was purified by flash chromatography on SiO_2 (pentane/EtOAc = 99:1 to 98:2) to afford compound **3v** (0.54 g, 2.0 mmol, 67%).

Appearance: Colourless oil.

TLC: R*f* = 0.55 (pentane/EtOAc = 98:2; stains in $KMnO_4$).

^{1}**H-NMR** (400 MHz, $CDCl_3$) δ (ppm) = 5.38 – 5.33(m, 1H), 5.10 (ddt, *J* = 6.9, 5.6, 1.4 Hz, 1H), 4.26 (d, *J* = 6.5 Hz, 2H), 4.15 (t, *J* = 1.5 Hz, 1H), 2.13 – 2.07 (m, 2H), 2.06 – 1.98 (m, 2H), 1.68 (s, 3H), 1.64 (s, 3H), 1.60 (s, 3H), 1.08 – 0.96 (m, 14H).

^{13}**C-NMR** (101 MHz, $CDCl_3$) δ (ppm) = 137.8, 131.6, 124.1, 123.6, 62.5, 39.5, 26.4, 25.7, 17.7, 17.42, 17.36, 16.4, 12.4.

HRMS (ESI-TOF) m/*z*: $[M+Na]^+$ Calcd for $C_{16}H_{32}OSiNa$ 291.2115 ; Found 291.2118.

*Synthesis of tert-butyl 5-((diisopropylsilyl)oxy)-1H-indole-1-carboxylate (**3x**)*

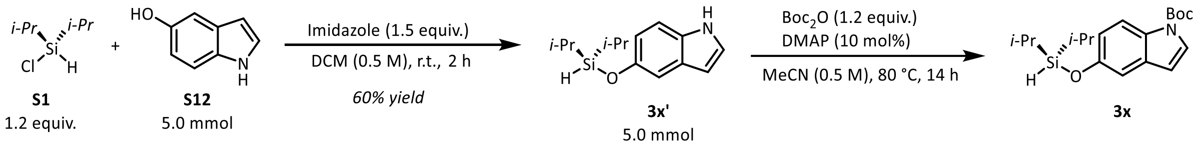

Compound **3x'** was synthesized following the procedure reported by Huang and co-workers for a similar compound.[8] A 50 mL round-bottoed flask was charged with 5-hydroxyindole (**S12**; 0.66 g, 5.0 mmol, 1.0 equiv.) and imidazole (0.50 g, 7.5 mmol, 1.5 equiv.). The mixture was evacuated and backfilled with argon (three times). Dry DCM (0.5 M) was added followed by the dropwise addition of chlorodiisopropylsilane (**S1**; 1.0 mL, 6.0 mmol, 1.2 equiv.). The resulting mixture was stirred at room temperature for 2 h. Upon completion, the reaction mixture was poured in water (50 mL) and extracted with DCM (3 × 50 mL). The combined organic layers was dried over Na_2SO_4, filtered and concentrated under reduced pressure. The crude product was purified by flash chromatography on SiO_2 (pentane/EtOAc = 99:1 to 98:2) to afford compound **3x'**.

Characterization data for compound 3x'

Appearance: Colourless oil.

TLC: R_{f} = 0.55 (pentane/EtOAc = 98:2; UV-active and stains red in vanillin).

^{1}**H-NMR** (400 MHz, $CDCl_3$) δ (ppm) = 8.00 (s, 1H), 7.24 (d, *J* = 8.7 Hz, 1H), 7.18 – 7.14 (m, 2H), 6.85 (dd, *J* = 8.7, 2.3 Hz, 1H), 6.48 – 6.43 (m, 1H), 4.56 (d, *J* = 1.5 Hz, 1H), 1.22 – 1.07 (m, 14H).

^{13}**C-NMR** (101 MHz, $CDCl_3$) δ (ppm) = 150.3, 131.4, 128.5, 124.9, 115.4, 111.3, 109.1, 102.3, 17.4, 17.3, 12.5.

A flame-dried microwave vial was charged with 5-((diisopropylsilyl)oxy)-1*H*-indole (**3x'**; 1.2 g, 5.0 mmol, 1.0 equiv.), *tert*-butyl dicarbonate (1.3 g, 6.0 mmol, 1.2 equiv.) and DMAP (61 mg, 0.50 mmol, 10 mol%). The vial was evacuated and backfilled with argon (three times), followed by addition of dry acetonitrile (10 mL). The resulting mixture was heated at 80 ˚C overnight. Upon completion, the solvent was evaporated under reduced pressure and the crude was purified by flash chromatography on SiO_2 (pentane/EtOAc = 99:1 to 98:2) to afford compound **3x** (0.84 g, 2.5 mmol, 50%).

Appearance: Colourless oil.

TLC: R_f = 0.55 (pentane/EtOAc = 98:2; UV-active and stains red in vanillin).

^{1}H-NMR (400 MHz, $CDCl_3$) δ (ppm) = 7.97 (d, *J* = 7.7 Hz, 1H), 7.55 (d, *J* = 3.4 Hz, 1H), 7.06 (d, *J* = 2.4 Hz, 1H), 6.91 (dd, *J* = 8.9, 2.4 Hz, 1H), 6.46 (d, *J* = 3.7 Hz, 1H), 4.55 (t, *J* = 1.7 Hz, 1H), 1.66 (s, 9H), 1.16 – 1.03 (m, 14H).

^{13}C-NMR (101 MHz, $CDCl_3$) δ (ppm) = 152.2, 149.7, 131.5, 130.3, 126.5, 116.6, 115.6, 110.0, 107.0, 83.4, 28.2, 17.3, 17.2, 12.4.

HRMS (ESI-TOF) m/*z*: $[M+Na]^+$ Calcd for $C_{19}H_{29}NO_3SiNa$ 370.1809; Found 370.1800.

3. Results and Discussion

3.1 Optimization of the Si–H insertion reaction using NHPI-DA (8).

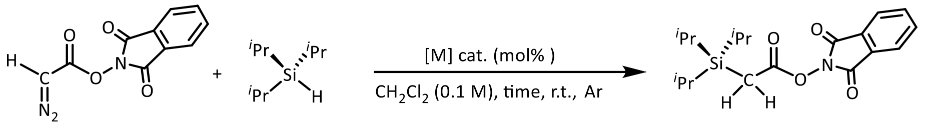

NHPI-DA (**8**) 1.0 equiv. — **3a** (2.0 equiv.) — **9a**

entry	[M] Cat. (mol%)	time (h)	conv. **8** (%)	yield **9a** (%)[a]
1	$Rh_2(OAc)_4$ (1)	14	> 99	21
2	$Rh_2(piv)_4$ (1)	14	> 99	9
3	$Cu(OTf)_2$ (10)	14	32	13
4	$[Cu(OTf)]_2{\cdot}C_6H_6$ (10)	14	> 99	15
5	$[Cu(MeCN)_4]PF_6$ (10) + **L1** (10)	14	> 99	15
6	$[Cu(MeCN)_4]PF_6$ (10) + **L2** (10)	14	> 99	18
7	Fe**L3** (10)	14	10	5
8	Fe**L4** (10)	14	5	n.d.
9	[Ru(*p*-cymene)Cl_2]$_2$ (2.5)	14	> 99	39
10	[Ru**L5**$(MeCN)_4$]PF_6 (5)	14	> 99	97
11[b]	[Ru**L5**$(MeCN)_4$]PF_6 (1)	0.5	> 99	95

[a] Yields were determined by ^{1}H-NMR using 1,1,2,2-tetrachloroethane as internal standard; [b] 1.1 equiv. of **3a** was used; n.d. is not detected; **L1** = 2,2'-bibyridine; **L2** = (S)-*i*-Pr-BOX; **L3** = tetraphenyl porphyrin; **L4 =** phthalocyanine; **L5** = 4,4-dimethyl-2-phenyloxazolin-2'-yl; n.d. not detected.

3.2 General procedure E: Ru(II)-Pheox catalyzed Si–H insertion of silanes

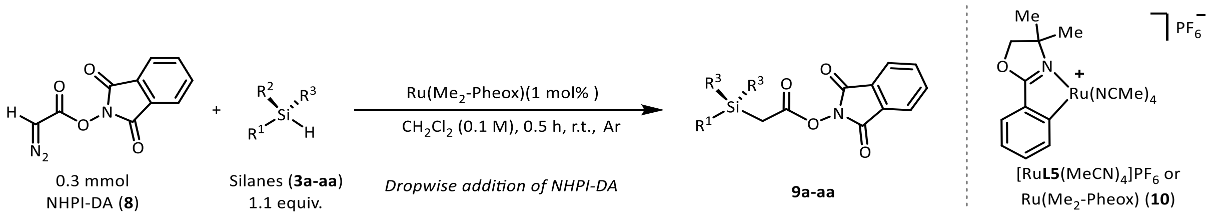

0.3 mmol NHPI-DA (**8**) — Silanes (**3a-aa**) 1.1 equiv. — *Dropwise addition of NHPI-DA* — **9a-aa** — [Ru**L5**$(MeCN)_4$]PF_6 or Ru(Me_2-Pheox) (**10**)

A flame-dried vial was charged with a stirring bar and silane (**3a-aa**; 0.33 mmol, 1.1 equiv.). The vial was evacuated and refilled with argon (three cycles), followed by the addition of a solution of Ru(Me_2-Pheox) (**10**; 3.0 µmol, 0.01 equiv.) in dry DCM (1.0 mL). The mixture was stirred for 5 min at room temperature, followed by dropwise addition (over a period of 1 to 2 min) of a solution of *N*-hydroxyphthalimidyl diazoacetate (NHPI-DA **8**; 0.30 mmol, 1.0 equiv.) in dry DCM (2.0 mL). The resulting mixture was stirred for 30 min at room temperature. Upon completion, the solvent was evaporated under reduced pressure and the crude was purified by flash chromatography on SiO_2 (unless otherwise specified) using pentane/EtOAc to afford α-silyl NHPI esters **9a-aa**.

*Synthesis of 1,3-dioxoisoindolin-2-yl 2-(triisopropylsilyl)acetate (**9a**)*

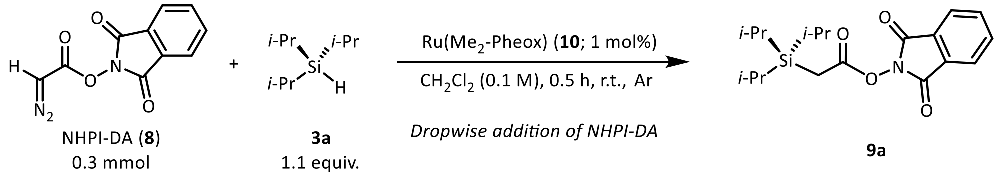

General procedure E was applied using NHPI-DA (**8**; 69 mg, 0.30 mmol, 1.0 equiv.), triisopropylsilane (**3a**; 52 mg, 0.33 mmol, 1.1 equiv.) and Ru(Me_2-Pheox) (**10**; 1.8 mg, 3.0 µmol, 0.01 equiv.) in DCM (0.1 M) for 0.5 h at room temperature. The crude was purified by flash chromatography on SiO_2 (pentane/EtOAc = 10:1) to afford compound **9a** (0.10 g, 0.27 mmol, 92%).

Appearance: white solid.

TLC: R_f = 0.57 (Pentane/EtOAc = 10:1; UV-active).

m.p.: 101.2˚C – 102.8˚C

^{1}H-NMR (400 MHz, $CDCl_3$) δ (ppm) = 7.87 – 7.82 (m, 2H), 7.78 – 7.73 (m, 2H), 2.22 (s, 2H), 1.34 – 1.25 (m, 3H), 1.14 (d, *J* = 7.5 Hz, 18H).

^{13}C-NMR (101 MHz, $CDCl_3$) δ (ppm) = 169.7, 162.4, 134.7, 129.2, 123.9, 18.5, 15.7, 11.4.

HRMS (ESI-TOF) m/*z* : $[M+Na]^+$ Calcd for $C_{19}H_{27}NO_4SiNa$ 384.1602; Found 384.1602.

*Synthesis of 1,3-dioxoisoindolin-2-yl 2-(triethylsilyl)acetate (**9b**)*

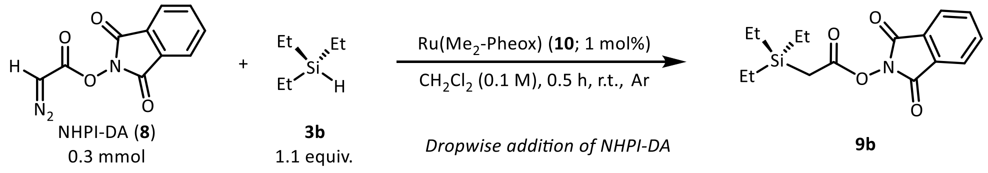

General procedure E was applied using NHPI-DA (**8**; 69 mg, 0.30 mmol, 1.0 equiv.), diethylsilane (**3b**; 39 mg, 0.33 mmol, 1.1 equiv.) and Ru(Me_2-Pheox) (**10**; 1.8 mg, 3.0 µmol, 0.01 equiv.) in DCM (0.1 M) for 0.5 h at room temperature. The crude was purified by flash chromatography on SiO_2 (pentane/EtOAc = 10:1) to afford compound **9b** (73 mg, 0.23 mmol, 76%).

Appearance: white solid.

TLC: R_f = 0.43 (Pentane/EtOAc = 10:1; UV-active).

m.p.: 56.6°C – 58.5°C

^{1}H-NMR (400 MHz, $CDCl_3$) δ (ppm) = 7.87 – 7.85 (m, 2H), 7.77 – 7.75 (m, 2H), 2.18 (s, 2H), 1.02 (t, *J* = 7.9 Hz, 9H), 0.79 (q, *J* = 8.3 Hz, 6H).

^{13}C-NMR (101 MHz, $CDCl_3$) δ (ppm) = 169.0, 162.4, 134.7, 129.2, 123.9, 18.6, 7.1, 3.4.

HRMS: (ESI-TOF) m/*z*: $[M+Na]^+$ Calcd for $C_{16}H_{21}NO_4SiNa$ 342.1132; Found 342.1133.

*Synthesis of 1,3-dioxoisoindolin-2-yl 2-(benzyldiisopropylsilyl)acetate (**9c**)*

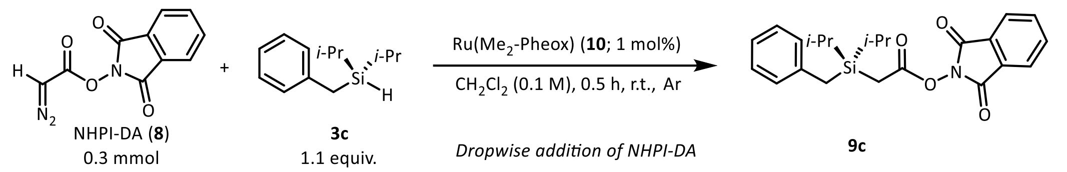

General procedure E was applied using NHPI-DA (**8**; 69 mg, 0.30 mmol, 1.0 equiv.), benzyldiisopropylsilane (**3c**; 68 mg, 0.33 mmol, 1.1 equiv.) and Ru(Me_2-Pheox) (**10**; 1.8 mg, 3.0 µmol, 0.01 equiv.) in DCM (0.1 M) for 0.5 h at room temperature. The crude was purified by flash chromatography on SiO_2 (pentane/EtOAc = 10:1) to afford compound **9c** (0.11 g, 0.28 mmol, 94%).

Appearance: colourless oil.

TLC: R_f = 0.5 (Pentane/EtOAc = 9:1; UV-active and stains in $KMnO_4$).

^{1}H-NMR (400 MHz, $CDCl_3$) δ (ppm) = 7.89 – 7.86 (m, 2H), 7.80 – 7.75 (m, 2H), 7.26 – 7.21 (m, 2H), 7.20 – 7.15 (m, 2H), 7.12 – 7.07 (m, 1H), 2.44 (s, 2H), 2.20 (s, 2H), 1.30 – 1.19 (m, 2H), 1.07 (dd, *J* = 7.4, 1.0 Hz, 12H).

^{13}C-NMR (101 MHz, $CDCl_3$) δ (ppm) = 169.2, 162.2, 138.9, 134.7, 129.0, 128.6, 128.5, 124.5, 123.9, 19.4, 17.83, 17.79, 16.4, 11.5.

HRMS: (ESI-TOF) m/*z* : $[M+Na]^+$ Calcd for $C_{23}H_{27}NO_4SiNa$ 432.1602; Found 432.1601.

*Synthesis of 1,3-dioxoisoindolin-2-yl 2-(diisopropyl(pent-4-en-1-yl)silyl)acetate (**9d**)*

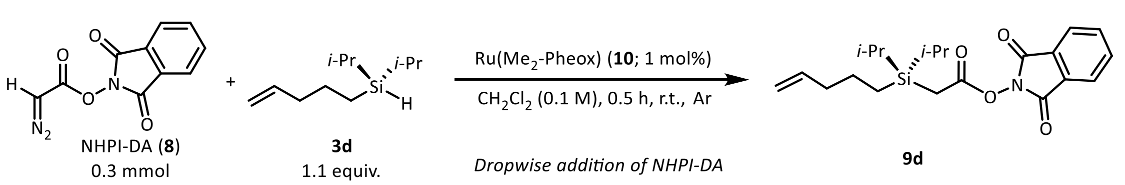

General procedure E was applied using NHPI-DA (**8**; 69 mg, 0.30 mmol, 1.0 equiv.), diisopropyl(pent-4-en-1-yl)silane (**3d**; 61 mg, 0.33 mmol, 1.1 equiv.) and Ru(Me_2-Pheox) (**10**; 1.8 mg, 3.0 µmol, 0.01 equiv.) in DCM (0.1 M) for 0.5 h at room temperature. The crude was purified by flash chromatography on SiO_2 (pentane/EtOAc = 10:1) to afford compound **9d** (98 mg, 0.25 mmol, 84%).

Appearance: colourless oil.

TLC: R_f = 0.5 (Pentane/EtOAc = 10:1; UV-active and stains in $KMnO_4$).

^{1}H-NMR (400 MHz, $CDCl_3$) δ (ppm) = 7.89 – 7.83 (m, 2H), 7.79 – 7.74 (m, 2H), 5.81 (ddt, *J* = 17.0, 10.2, 6.7 Hz, 1H), 5.07 – 4.93 (m, 2H), 2.19 (s, 2H), 2.11 (q, *J* = 7.1 Hz, 2H), 1.57 – 1.47 (m, 2H), 1.26 – 1.15 (m, 2H), 1.10 (d, *J* = 6.3 Hz, 12H), 0.89 – 0.82 (m, 2H).

^{13}C-NMR (101 MHz, $CDCl_3$) δ (ppm) = 169.3, 162.2, 138.5, 134.6, 129.0, 123.8, 114.8, 37.8, 23.2, 18.0, 16.6, 11.5, 9.5.

HRMS: (ESI-TOF) m/*z* : $[M+Na]^+$ Calcd for $C_{21}H_{29}NO_4SiNa$ 410.1758; Found 410.1755.

*Synthesis of 1,3-dioxoisoindolin-2-yl 2-((4-chlorobutyl)diisopropylsilyl)acetate (**9e**)*

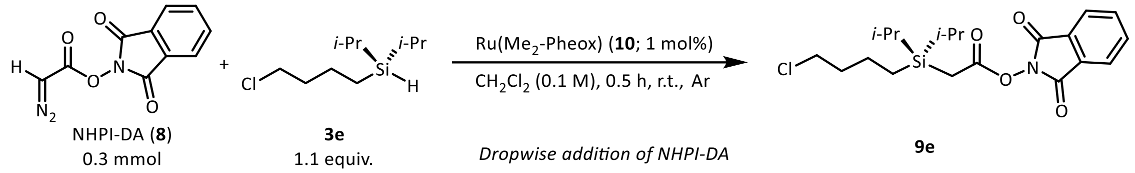

General procedure E was applied using NHPI-DA (**8**; 69 mg, 0.30 mmol, 1.0 equiv.), (4-chlorobutyl)diisopropylsilane (**3e**; 68 mg, 0.33 mmol, 1.1 equiv.) and Ru(Me_2-Pheox) (**10**; 1.8 mg, 3.0 µmol, 0.01 equiv.) in DCM (0.1 M) for 0.5 h at room temperature. The crude was purified by flash chromatography on SiO_2 (pentane/EtOAc = 10:1) to afford compound **9e** (0.11 g, 0.26 mmol, 87%).

Appearance: colourless oil.

TLC: R_f = 0.58 (Pentane/EtOAc = 8:2; UV-active and stains in $KMnO_4$).

^{1}H-NMR (400 MHz, $CDCl_3$) δ (ppm) = 7.89 – 7.84 (m, 2H), 7.79 – 7.74 (m, 2H), 3.57 (t, *J* = 6.6 Hz, 2H), 2.20 (s, 2H), 1.84 (p, *J* = 6.8 Hz, 2H), 1.63 – 1.55 (m, 2H), 1.26 – 1.14 (m, 2H), 1.10 (d, *J* = 6.5 Hz, 12H), 0.91 – 0.82 (m, 2H).

^{13}C-NMR (101 MHz, $CDCl_3$) δ (ppm) = 169.2, 162.2, 134.6, 129.0, 123.8, 44.5, 36.2, 20.8, 18.0, 16.5, 11.6, 9.0.

HRMS: (ESI-TOF) m/*z* $[M+Na]^+$ Calcd for $C_{20}H_{28}ClNO_4SiNa$ 432.1368; Found 432.1365.

*Synthesis of 1,3-dioxoisoindolin-2-yl 2-((3-(benzyloxy)propyl)diisopropylsilyl)acetate (**9f**)*

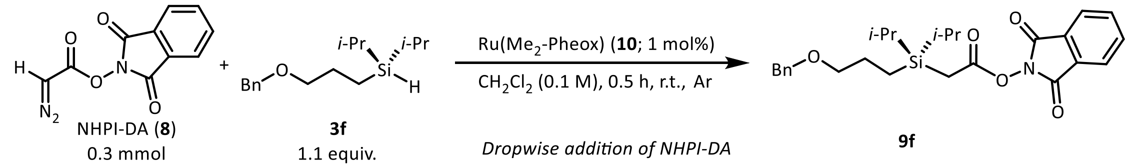

General procedure E was applied using NHPI-DA (**8**; 69 mg, 0.30 mmol, 1.0 equiv.), (3-(benzyloxy)propyl)diisopropylsilane (**3f**; 87 mg, 0.33 mmol, 1.1 equiv.) and Ru(Me_2-Pheox) (**10**; 1.8 mg, 3.0 µmol, 0.01 equiv.) in DCM (0.1 M) for 0.5 h at room temperature. The crude was purified by flash chromatography on SiO_2 (pentane/EtOAc = 10:1) to afford compound **9f** (0.11 g, 0.23 mmol, 79%).

Appearance: colourless oil.

TLC: R_f = 0.46 (Pentane/EtOAc = 8:2; UV-active and stains in $KMnO_4$).

^{1}H-NMR (400 MHz, $CDCl_3$) δ (ppm) = 7.88 – 7.84 (m, 2H), 7.80 – 7.74 (m, 2H), 7.36 – 7.28 (m, 4H), 7.26 – 7.21 (m, 1H), 4.51 (s, 2H), 3.48 (t, *J* = 6.9 Hz, 2H), 2.21 (s, 2H), 1.82 – 1.71 (m, 2H), 1.29 – 1.16 (m, 2H), 1.11 (d, *J* = 6.4 Hz, 12H), 0.90 – 0.83 (m, 2H).

^{13}C-NMR (101 MHz, $CDCl_3$) δ (ppm) = 169.2, 162.2, 138.7, 134.6, 129.0, 128.3, 127.6, 127.4, 123.8, 73.3, 72.8, 23.9, 18.0, 16.6, 11.6, 6.0.

HRMS: (ESI-TOF) m/*z* : $[M+Na]^+$ Calcd for $C_{26}H_{33}NO_5SiNa$ 490.2020; Found 490.2025.

*Synthesis of 1,3-dioxoisoindolin-2-yl 2-(diisopropyl(vinyl)silyl)acetate (**9g**)*

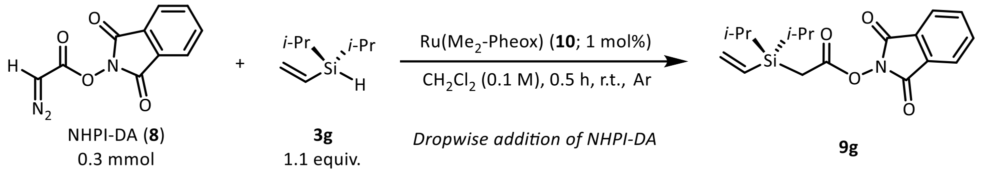

General procedure E was applied using NHPI-DA (**8**; 69 mg, 0.30 mmol, 1.0 equiv.), diisopropyl(vinyl)silane (**3g**; 47 mg, 0.33 mmol, 1.1 equiv.) and Ru(Me_2-Pheox) (**10**; 1.8 mg, 3.0 µmol, 0.01 equiv.) in DCM (0.1 M) for 0.5 h at room temperature. The crude was purified by flash chromatography on SiO_2 (pentane/EtOAc = 10:1) to afford compound **9g** (95 mg, 0.27 mmol, 92%).

Appearance: colourless oil.

TLC: R_f = 0.45 (Pentane/EtOAc = 10:1; UV-active and stains in $KMnO_4$).

^{1}H NMR (400 MHz, $CDCl_3$) δ (ppm) = 7.91 – 7.85 (m, 2H), 7.81 – 7.76 (m, 2H), 6.25 – 6.08 (m, 2H), 5.88 (dd, *J* = 18.2, 5.7 Hz, 1H), 2.30 (s, 2H), 1.36 – 1.27 (m, 2H), 1.13 (d, *J* = 8.5 Hz, 12H).

^{13}C NMR (101 MHz, $CDCl_3$) δ (ppm) = 169.1, 162.3, 135.7, 134.8, 131.5, 129.5, 124.0, 17.8, 17.7, 16.4, 11.2.

HRMS: (ESI-TOF) m/*z* : $[M+Na]^+$ Calcd for $C_{18}H_{23}NO_4SiNa$ 368.1289; Found 368.1295.

*Synthesis of 1,3-dioxoisoindolin-2-yl 2-(diisopropyl(phenyl)silyl)acetate (**9h**)*

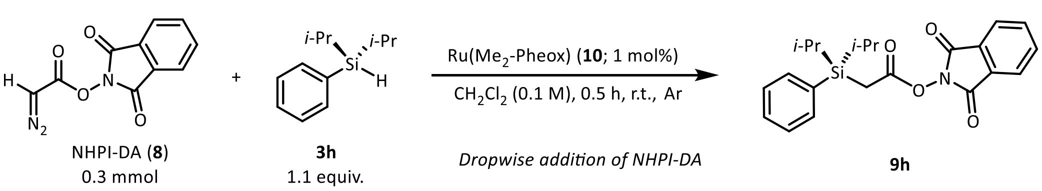

General procedure E was applied using NHPI-DA (**8**; 69 mg, 0.30 mmol, 1.0 equiv.), diisopropyl(phenyl)silane (**3h**; 64 mg, 0.33 mmol, 1.1 equiv.) and Ru(Me_2-Pheox) (**10**; 1.8 mg, 3.0 µmol, 0.01 equiv.) in DCM (0.1 M) for 0.5 h at room temperature. The crude was purified by flash chromatography on SiO_2 (pentane/EtOAc = 10:1) to afford compound **9h** (0.11 g, 0.29 mmol, 96%).

Appearance: colourless oil.

TLC: R_f = 0.45 (Pentane/EtOAc = 9:1; UV-active and stains in $KMnO_4$).

^{1}H-NMR (400 MHz, $CDCl_3$) δ (ppm) = 7.91 – 7.85 (m, 2H), 7.80 – 7.74 (m, 2H), 7.59 – 7.53 (m, 2H), 7.44 – 7.36 (m, 3H), 2.53 (s, 2H), 1.58 (hept, *J* = 7.4 Hz, 2H), 1.13 (dd, *J* = 12.5, 7.4 Hz, 12H).

^{13}C-NMR (101 MHz, $CDCl_3$) δ (ppm) = 169.1, 162.2, 134.7, 134.6, 132.3, 129.6, 129.0, 127.9, 123.9, 17.6, 17.5, 16.1, 11.0.

HRMS: (ESI-TOF) m/*z* : $[M+Na]^+$ Calcd for $C_{22}H_{25}NO_4SiNa$ 418.1445; Found 418.1446.

*Synthesis of 1,3-dioxoisoindolin-2-yl 2-(dimethyl(phenyl)silyl)acetate (**9i**)*

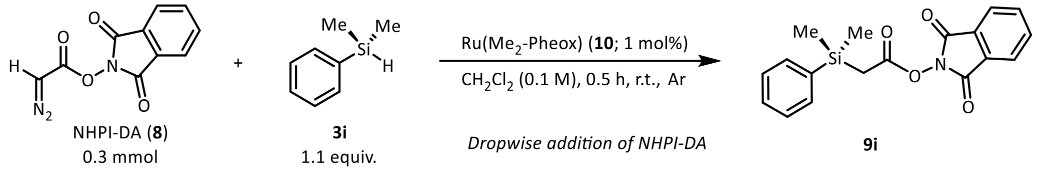

General procedure E was applied using NHPI-DA (**8**; 69 mg, 0.30 mmol, 1.0 equiv.), dimethyl(phenyl)silane (**3j**; 45 mg, 0.33 mmol, 1.1 equiv.) and Ru(Me_2-Pheox) (**10**; 1.8 mg, 3.0 µmol, 0.01 equiv.) in DCM (0.1 M) for 0.5 h at room temperature. The crude was purified by flash chromatography on SiO_2 (pentane/EtOAc = 10:1) to afford compound **9i** (79 mg, 0.23 mmol, 78%).

Appearance: white solid.

m.p.: 98.5 – 102.1 °C

TLC: R_f = 0.52 (Pentane/EtOAc = 8:2; UV-active and stains in $KMnO_4$).

^{1}H-NMR (400 MHz, $CDCl_3$) δ (ppm) = 7.90 – 7.86 (m, 2H), 7.80 – 7.77 (m, 2H), 7.62 – 7.60 (m, 2H), 7.42 – 7.38 (m, 3H), 2.40 (s, 2H), 0.59 (s, 6H).

^{13}C-NMR (101 MHz, $CDCl_3$) δ (ppm) = 168.3, 162.2, 135.9, 134.6, 133.6, 129.9, 129.0, 128.1, 123.9, 23.2, -3.2.

HRMS: (ESI-TOF) m/*z* : $[M+NH_4]^+$ Calcd for $C_{18}H_{17}NO_4SiNH_4$: 357.1265; Found 357.1256.

*Synthesis of 1,3-dioxoisoindolin-2-yl 2-((4-fluorophenyl)diisopropylsilyl)acetate (**9j**)*

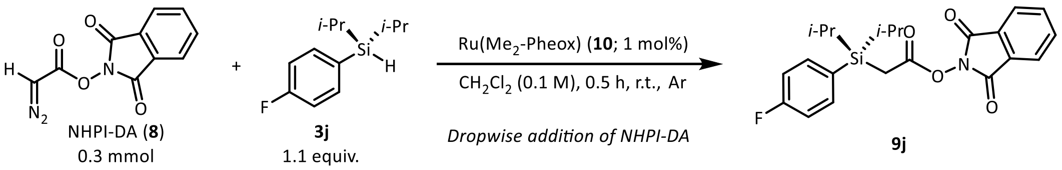

General procedure E was applied using NHPI-DA (**8**; 69 mg, 0.30 mmol, 1.0 equiv.), (4-fluorophenyl)diisopropylsilane (**3j**; 69 mg, 0.33 mmol, 1.1 equiv.) and Ru(Me_2-Pheox) (**10**; 1.8 mg, 3.0 µmol, 0.01 equiv.) in DCM (0.1 M) for 0.5 h at room temperature. The crude was purified by flash chromatography on SiO_2 (pentane/EtOAc = 10:1) to afford compound **9j** (0.11 g, 0.27 mmol, 91%).

Appearance: white solid.

m.p.: 70.3 – 73.5 °C

TLC: R$_f$ = 0.55 (Pentane/EtOAc = 9:1; UV-active and stains in $KMnO_4$).

^{1}H-NMR (400 MHz, $CDCl_3$) δ (ppm) = 7.88 – 7.84 (m, 2H), 7.79 – 7.76 (m, 2H), 7.59 – 7.53 (m, 2H), 7.14 – 7.07 (m, 2H), 2.50 (s, 2H), 1.55 (dt, *J* = 14.8, 7.4 Hz, 2H), 1.12 (dd, *J* = 11.2, 7.4 Hz, 12H).

^{13}C-NMR (101 MHz, $CDCl_3$) δ (ppm) = 168.9, 165.3 (d, $J_{C\text{-}F}$ = 249.2 Hz), 162.1, 136.7 (d, $J_{C\text{-}F}$ = 7.6 Hz), 134.7, 129.0, 127.8 (d, $J_{C\text{-}F}$ = 3.7 Hz), 123.9, 115.3 (d, $J_{C\text{-}F}$ = 19.7 Hz), 17.63, 17.55, 16.3, 11.1.

^{19}F-NMR (377 MHz, $CDCl_3$) δ (ppm) = –110.82.

HRMS: (ESI-TOF) m/*z* : [M+Na]$^+$ Calcd for $C_{22}H_{24}FNO_4SiNa$ 436.1351; Found 436.1350.

*Synthesis of 1,3-dioxoisoindolin-2-yl 2-((4-chlorophenyl)dimethylsilyl)acetate (**9k**)*

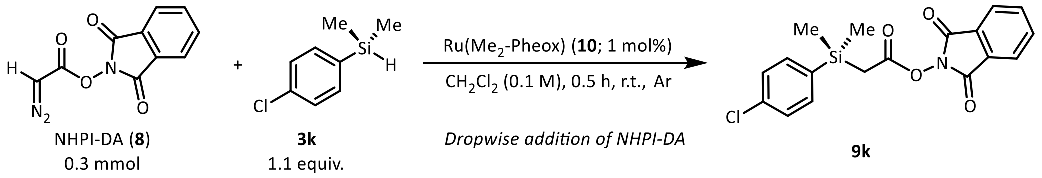

General procedure E was applied using NHPI-DA (**8**; 69 mg, 0.30 mmol, 1.0 equiv.), (4-chlorophenyl)dimethylsilane (**3k**; 56 mg, 0.33 mmol, 1.1 equiv.) and Ru(Me_2-Pheox) (**10**; 1.8 mg, 3.0 µmol, 0.01 equiv.) in DCM (0.1 M) for 0.5 h at room temperature. The crude was purified by flash chromatography on SiO_2 (pentane/EtOAc = 10:1) to afford compound **9k** (0.10 g, 0.28 mmol, 93%).

Appearance: colourless oil.

TLC: R$_f$ = 0.55 (Pentane/EtOAc = 9:1; UV-active and stains in $KMnO_4$).

^{1}H-NMR (400 MHz, $CDCl_3$) δ (ppm) = 7.90 – 7.84 (m, 2H), 7.79 – 7.75 (m, 2H), 7.54 (d, *J* = 8.4 Hz, 2H), 7.38 (d, *J* = 8.3 Hz, 2H), 2.37 (s, 2H), 0.58 (s, 6H).

^{13}C-NMR (101 MHz, $CDCl_3$) δ (ppm) = 168.1, 162.2, 135.0, 134.7, 134.1, 129.0, 128.4, 123.9, 23.2, -3.1.

HRMS: (ESI-TOF) m/*z* : [M+Na]$^+$ Calcd for $C_{18}H_{16}ClNO_4SiNa$ 396.0429; Found 396.0439.

*Synthesis of 1,3-dioxoisoindolin-2-yl 2-(diisopropyl(4-((trimethylsilyl)ethynyl)phenyl)silyl)acetate (**9l**)*

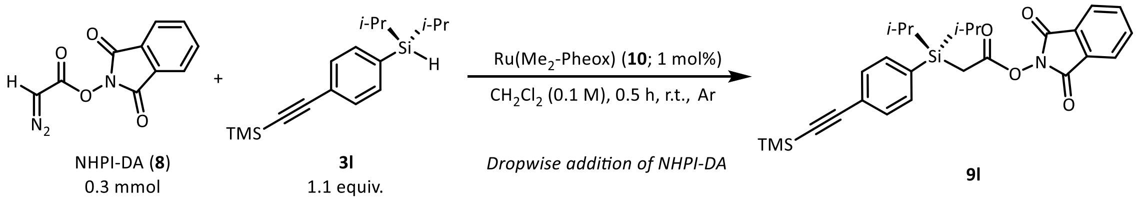

General procedure E was applied using NHPI-DA (**8**; 69 mg, 0.30 mmol, 1.0 equiv.), ((4-(diisopropylsilyl)phenyl)ethynyl)trimethylsilane (**3l**; 95 mg, 0.33 mmol, 1.1 equiv.) and Ru(Me$_2$-Pheox) (**10**; 1.8 mg, 3.0 µmol, 0.01 equiv.) in DCM (0.1 M) for 0.5 h at room temperature. The crude was purified by flash chromatography on SiO_2 (pentane/EtOAc = 10:1) to afford compound **9l** (0.13 g, 0.26 mmol, 88%).

Appearance: colourless oil.

TLC: R_f = 0.5 (Pentane/EtOAc = 9:1; UV-active and stains green in vanillin).

^{1}H-NMR (400 MHz, $CDCl_3$) δ (ppm) = 7.88 – 7.84 (m, 2H), 7.79 – 7.75 (m, 2H), 7.53 – 7.46 (m, 4H), 2.50 (s, 2H), 1.60 – 1.52 (m, 2H), 1.11 (dd, *J* = 12.7, 7.4 Hz, 12H), 0.25 (s, 9H).

^{13}C-NMR (101 MHz, $CDCl_3$) δ (ppm) = 168.9, 162.1, 134.6, 134.4, 133.2, 131.2, 129.0, 124.3, 123.9, 104.9, 95.4, 17.6, 17.5, 16.0, 11.0, -0.1.

HRMS: (ESI-TOF) m/*z* : $[M+Na]^+$ Calcd for $C_{27}H_{33}NO_4Si_2Na$ 514.1845; Found 514.1840.

*Synthesis of 1,3-dioxoisoindolin-2-yl 2-(diisopropyl(thiophen-2-yl)silyl)acetate (**9m**)*

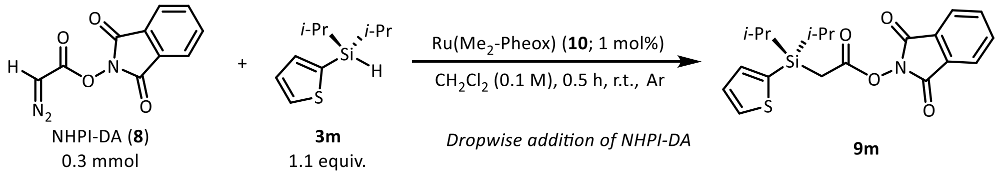

General procedure E was applied using NHPI-DA (**8**; 69 mg, 0.30 mmol, 1.0 equiv.), diisopropyl(thiophen-2-yl)silane (**3m**; 65 mg, 0.33 mmol, 1.1 equiv.) and Ru(Me$_2$-Pheox) (**10**; 1.8 mg, 3.0 µmol, 0.01 equiv.) in DCM (0.1 M) for 0.5 h at room temperature. The crude was purified by flash chromatography on SiO_2 (pentane/EtOAc = 10:1) to afford compound **9m** (0.12 g, 0.29 mmol, 97%).

Appearance: colourless oil.

TLC: R_f = 0.5 (Pentane/EtOAc = 9:1; UV-active and stains in $KMnO_4$).

^{1}H-NMR (400 MHz, $CDCl_3$) δ (ppm) = 7.91 – 7.83 (m, 2H), 7.80 – 7.75 (m, 2H), 7.68 (dd, *J* = 4.6, 0.8 Hz, 1H), 7.42 (dd, *J* = 3.4, 0.9 Hz, 1H), 7.26 (dd, *J* = 4.7, 3.4 Hz, 1H), 2.55 (s, 2H), 1.61 – 1.49 (m, 2H), 1.16 (dd, *J* = 7.5, 4.9 Hz, 12H).

^{13}C-NMR (101 MHz, $CDCl_3$) δ (ppm) = 169.0, 162.2, 136.5, 134.6, 131.4, 130.7, 129.0, 128.3, 123.8, 17.7, 17.6, 16.3, 11.0.

HRMS: (ESI-TOF) m/*z* : $[M+Na]^+$ Calcd for $C_{20}H_{23}NO_4SSiNa$ 424.1009; Found 424.1010.

*Synthesis of 1,3-dioxoisoindolin-2-yl 2-(diphenylsilyl)acetate (**9n**)*

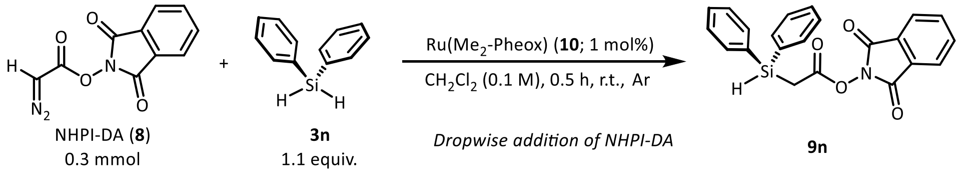

General procedure E was applied using NHPI-DA (**8**; 69 mg, 0.30 mmol, 1.0 equiv.), diphenylsilane (**3n**; 61 mg, 0.33 mmol, 1.1 equiv.) and Ru(Me_2-Pheox) (**10**; 1.8 mg, 3.0 µmol, 0.01 equiv.) in DCM (0.1 M) for 0.5 h at room temperature. The crude was purified by recrystallization from $CHCl_3$/pentane to afford compound **9n** (0.12 g, 0.26 mmol, 88%; 18:1 *mono*/*di*).

Appearance: white solid.

TLC: R_f = 0.5 (Pentane/EtOAc = 8:2; UV-active).

m.p.: 107.3 – 110.6 °C

^{1}H-NMR (400 MHz, $CDCl_3$) δ (ppm) = 7.89 – 7.83 (m, 2H), 7.78 – 7.74 (m, 2H), 7.71 – 7.67 (m, 4H), 7.50 – 7.40 (m, 6H), 5.27 (t, *J* = 3.3 Hz, 1H), 2.76 (d, *J* = 3.4 Hz, 2H).

^{13}C-NMR (101 MHz, $CDCl_3$) δ (ppm) = 167.9, 161.9, 135.3, 134.6, 130.9, 130.6, 129.0, 128.3, 123.9, 19.9.

HRMS: (ESI-TOF) m/*z* : $[M+Na]^+$ Calcd for $C_{22}H_{17}NO_4SiNa$ 410.0819; Found 410.0805.

*Synthesis of 1,3-dioxoisoindolin-2-yl 2-(triphenylsilyl)acetate (**9o**)*

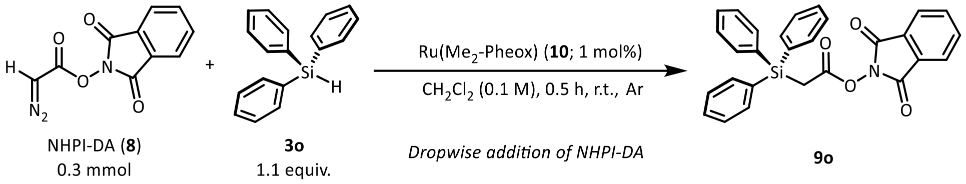

General procedure E was applied using NHPI-DA (**8**; 69 mg, 0.30 mmol, 1.0 equiv.), triphenylsilane (**3o**; 86 mg, 0.33 mmol, 1.1 equiv.) and Ru(Me_2-Pheox) (**10**; 1.8 mg, 3.0 µmol, 0.01 equiv.) in DCM (0.1 M) for 0.5 h at room temperature. The crude was purified by filtration through Florisil (Pentane/EtOAc = 10:1) to afford compound **9o** (0.12 g, 0.25 mmol, 85%).

Appearance: Off-white solid.

TLC: Decomposes on silica gel, not detectable.

m.p.: 210.3˚C – 213.2˚C

^{1}H-NMR (400 MHz, $CDCl_3$) δ (ppm) = 7.85 – 7.79 (m, 2H), 7.76 – 7.71 (m, 2H), 7.66 – 7.61 (m, 6H), 7.50 – 7.38 (m, 9H), 3.01 (s, 2H).

^{13}C-NMR (101 MHz, $CDCl_3$) δ (ppm) = 168.0, 162.0, 136.0, 134.7, 132.1, 130.5, 129.1, 128.3, 123.9, 21.1.

HRMS: (ESI-TOF) m/*z* : $[M+Na]^+$ Calcd for $C_{28}H_{21}NO_4SiNa$ 486.1132; Found 486.1130.

*Synthesis of 1,3-dioxoisoindolin-2-yl 2-((benzyloxy)diisopropylsilyl)acetate (**9p**)*

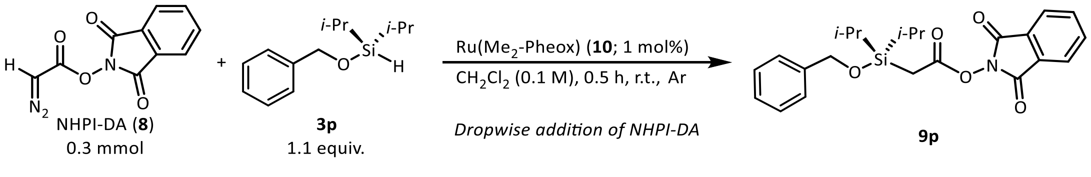

General procedure E was applied using NHPI-DA (**8**; 69 mg, 0.30 mmol, 1.0 equiv.), (benzyloxy)diisopropylsilane (**3p**; 73 mg, 0.33 mmol, 1.1 equiv.) and Ru(Me$_2$-Pheox) (**10**; 1.8 mg, 3.0 µmol, 0.01 equiv.) in DCM (0.1 M) for 0.5 h at room temperature. The crude was purified by flash chromatography on SiO_2 (pentane/EtOAc = 10:1) to afford compound **9p** (0.11 g, 0.26 mmol, 86%).

Appearance: colourless oil

TLC: R_f = 0.4 (Pentane/EtOAc = 9:1; UV-active and stains in $KMnO_4$).

^{1}H-NMR (400 MHz, $CDCl_3$) δ (ppm) = 7.89 – 7.85 (m, 2H), 7.79 – 7.75 (m, 2H), 7.40 – 7.29 (m, 4H), 7.28 – 7.22 (m, 1H), 4.96 (s, 2H), 2.38 (s, 2H), 1.38 (hept, *J* = 7.4 Hz, 2H), 1.16 (t, *J* = 7.2 Hz, 12H).

^{13}C-NMR (101 MHz, $CDCl_3$) δ (ppm) = 168.5, 162.2, 140.6, 134.7, 129.0, 128.2, 127.1, 126.1, 123.9, 65.5, 18.2, 17.3, 17.2, 12.7.

HRMS: (ESI-TOF) m/*z* : $[M+Na]^+$ Calcd for $C_{23}H_{27}NO_5SiNa$ 448.1551; Found 448.1551.

*Synthesis of 1,3-dioxoisoindolin-2-yl 2-((cyclohex-2-en-1-yloxy)diisopropylsilyl)acetate (**9q**)*

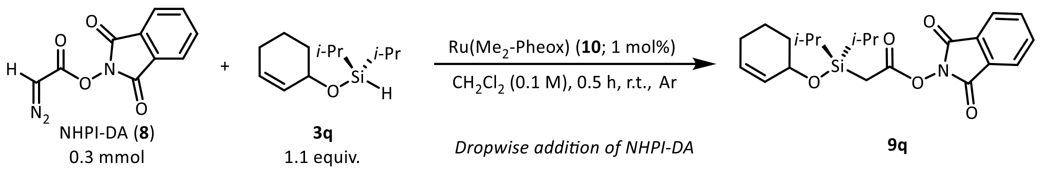

General procedure E was applied using NHPI-DA (**8**; 69 mg, 0.30 mmol, 1.0 equiv.), (cyclohex-2-en-1-yloxy)diisopropylsilane (**3q**; 70 mg, 0.33 mmol, 1.1 equiv.) and Ru(Me$_2$-Pheox) (**10**; 1.8 mg, 3.0 µmol, 0.01 equiv.) in DCM (0.1 M) for 0.5 h at room temperature. The crude was purified by flash chromatography on SiO_2 (pentane/EtOAc = 9:1) to afford compound **9q** (0.12 g, 0.28 mmol, 93%).

Appearance: colourless oil.

TLC: R_f = 0.58 (Pentane/EtOAc = 9:1; UV-active and stains in $KMnO_4$).

^{1}H-NMR (400 MHz, $CDCl_3$) δ (ppm) = 7.89 – 7.83 (m, 2H), 7.78 – 7.74 (m, 2H), 5.78 (ddt, *J* = 10.2, 3.5, 1.8 Hz, 1H), 5.74 – 5.69 (m, 1H), 4.48 – 4.45 (m, 1H), 2.34 (s, 2H), 2.07 – 1.98 (m, 1H), 1.97 – 1.83 (m, 2H), 1.81 – 1.73 (m, 1H), 1.70 – 1.62 (m, 1H), 1.60 – 1.49 (m, 1H), 1.35 – 1.22 (m, 2H), 1.13 (dd, *J* = 7.2, 2.5 Hz, 12H).

^{13}C-NMR (101 MHz, $CDCl_3$) δ (ppm) = 168.5, 162.2, 134.6, 130.3, 129.7, 129.0, 123.8, 67.2, 32.3, 25.0, 19.3, 18.4, 17.28, 17.26, 13.1, 13.0.

HRMS: (ESI-TOF) m/*z* : $[M+Na]^+$ Calcd for $C_{22}H_{29}NO_5SiNa$ 438.1707; Found 438.1706.

*Synthesis of 1,3-dioxoisoindolin-2-yl 2-(diisopropyl((4-methylpentan-2-yl)oxy)silyl)acetate (**9r**)*

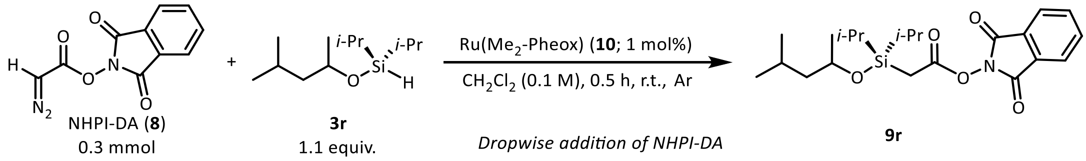

General procedure E was applied using NHPI-DA (**8**; 69 mg, 0.30 mmol, 1.0 equiv.), diisopropyl((4-methylpentan-2-yl)oxy)silane (**3r**; 71 mg, 0.33 mmol, 1.1 equiv.) and Ru(Me$_2$-Pheox) (**10**; 1.8 mg, 3.0 µmol, 0.01 equiv.) in DCM (0.1 M) for 0.5 h at room temperature. The crude was purified by flash chromatography on SiO_2 (pentane/EtOAc = 9:1) to afford compound **9r** (0.12 g, 0.28 mmol, 94%).

Appearance: colourless oil.

TLC: R_f = 0.6 (Pentane/EtOAc = 9:1; UV-active and stains in $KMnO_4$).

^{1}H-NMR (400 MHz, $CDCl_3$) δ (ppm) = 7.89 – 7.85 (m, 2H), 7.79 – 7.75 (m, 2H), 4.10 (h, *J* = 6.2 Hz, 1H), 2.33 (s, 2H), 1.67 (dh, *J* = 13.5, 6.8 Hz, 1H), 1.45 (dt, *J* = 13.6, 6.8 Hz, 1H), 1.31 – 1.22 (m, 3H), 1.20 (d, *J* = 6.0 Hz, 3H), 1.13 (dd, *J* = 7.1, 2.7 Hz, 12H), 0.89 (dd, *J* = 10.9, 6.6 Hz, 6H).

^{13}C-NMR (101 MHz, $CDCl_3$) δ (ppm) = 168.6, 162.2, 134.6, 129.0, 123.8, 68.1, 49.0, 24.7, 23.8, 23.1, 22.7, 18.5, 17.36, 17.32, 17.3, 17.29, 13.27, 13.2.

HRMS: (ESI-TOF) m/*z* : [M+Na]$^+$ Calcd for $C_{22}H_{33}NO_5SiNa$ 442.2020; Found: 442.2025.

*Synthesis of 1,3-dioxoisoindolin-2-yl 2-(diisopropyl(((1S,2R,5S)-2-isopropyl-5-methylcyclohexyl)oxy)silyl)acetate (**9s**)*

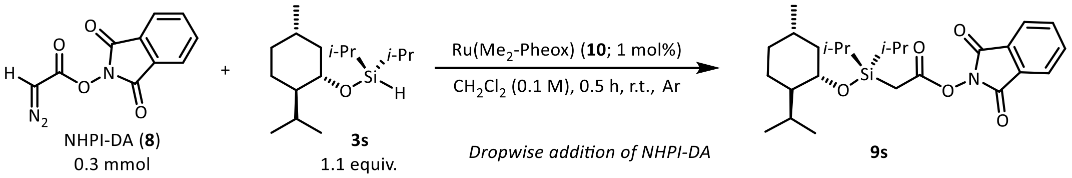

General procedure E was applied using NHPI-DA (**8**; 69 mg, 0.30 mmol, 1.0 equiv.), diisopropyl((4-methylpentan-2-yl)oxy)silane (**3s**; 89 mg, 0.33 mmol, 1.1 equiv.) and Ru(Me$_2$-Pheox) (**10**; 1.8 mg, 3.0 µmol, 0.01 equiv.) in DCM (0.1 M) for 0.5 h at room temperature. The crude was purified by flash chromatography on SiO_2 (pentane/EtOAc = 9:1) to afford compound **9s** (0.12 g, 0.25 mmol, 86%).

Appearance: colourless oil.

TLC: R_f = 0.6 (Pentane/EtOAc = 9:1; UV-active and stains in $KMnO_4$).

Appearance: colourless oil.

TLC: R_f = 0.7 (Pentane/EtOAc = 4:1, UV-active).

^{1}H-NMR (400 MHz, $CDCl_3$) δ (ppm) = 7.91 – 7.86 (m, 2H), 7.81 – 7.76 (m, 2H), 3.66 (td, *J* = 10.3, 4.3 Hz, 1H), 2.36 (s, 2H), 2.30 – 2.22 (m, 1H), 2.01 – 1.95 (m, 1H), 1.67 – 1.58 (m, 2H), 1.46 – 1.34 (m, 1H), 1.33 – 1.21 (m, 2H), 1.20 – 1.12 (m, 13H), 1.10 – 0.98 (m, 2H), 0.94 – 0.90 (m, 6H), 0.88 – 0.82 (m, 1H), 0.78 (d, *J* = 7.0 Hz, 3H).

^{13}C-NMR (101 MHz, $CDCl_3$) δ (ppm) = 168.7, 162.3, 134.7, 129.2, 124.0, 73.7, 50.5, 45.5, 34.6, 31.8, 25.3, 22.8, 22.4, 21.5, 19.1, 17.61, 17.58, 17.53, 17.50, 16.0, 13.8, 13.6.

HRMS: (ESI-TOF) m/*z* : [M+Na]$^+$ Calcd for $C_{26}H_{39}NO_5SiNa$ 496.2490; Found 496.2509

*Synthesis of ethyl (R)-2-(((2-((1,3-dioxoisoindolin-2-yl)oxy)-2-oxoethyl)diisopropylsilyl)oxy)propanoate (**9t**)*

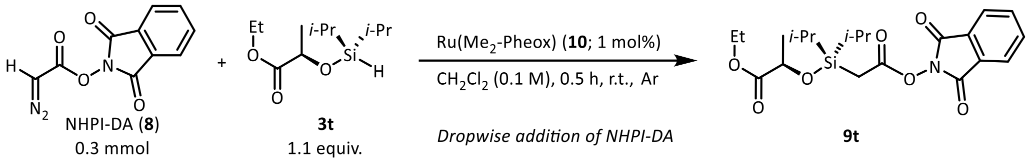

General procedure E was applied using NHPI-DA (**8**; 69 mg, 0.30 mmol, 1.0 equiv.), ethyl (*R*)-2-((diisopropylsilyl)oxy)propanoate (**3t**; 77 mg, 0.33 mmol, 1.1 equiv.) and Ru(Me$_2$-Pheox) (**10**; 1.8 mg, 3.0 µmol, 0.01 equiv.) in DCM (0.1 M) for 0.5 h at room temperature. The crude was purified by flash chromatography on SiO_2 (pentane/EtOAc = 9:1) to afford compound **9t** (0.10 g, 0.24 mmol, 81%).

Appearance: colourless oil.

TLC: R$_f$ = 0.7 (Pentane/EtOAc = 85:15; UV-active and stains in $KMnO_4$).

^{1}H-NMR (400 MHz, $CDCl_3$) δ (ppm) = 7.89 – 7.84 (m, 2H), 7.79 – 7.75 (m, 2H), 4.61 (q, *J* = 6.7 Hz, 1H), 4.23 – 4.12 (m, 2H), 2.41 (d, *J* = 12.3 Hz, 1H), 2.32 (d, *J* = 12.3 Hz, 1H), 1.45 (d, *J* = 6.7 Hz, 3H), 1.37 – 1.23 (m, 5H), 1.12 (t, *J* = 7.4 Hz, 12H).

^{13}C-NMR (101 MHz, $CDCl_3$) δ (ppm) = 173.7, 168.5, 162.1, 134.6, 129.0, 123.9, 68.8, 60.9, 21.3, 18.2, 17.16, 17.15, 17.1, 17.0, 14.2, 12.9, 12.8.

HRMS: (ESI-TOF) m/*z* : $[M+Na]^+$ Calcd for $C_{21}H_{29}NO_7SiNa$ 458.1605; Found 458.1608.

*Synthesis of 1,3-dioxoisoindolin-2-yl (R)-3,3-diisopropyl-6-(methoxycarbonyl)-10,10-dimethyl-8-oxo-4,9-dioxa-7-aza-3-silaundecanoate (**9u**)*

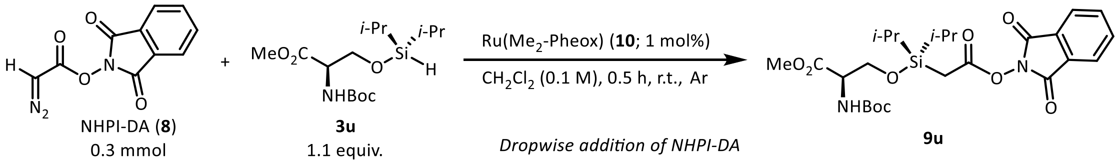

General procedure E was applied using NHPI-DA (**8**; 69 mg, 0.30 mmol, 1.0 equiv.), methyl *N*-(*tert*-butoxycarbonyl)-*O*-(diisopropylsilyl)-*D*-serinate (**3u**; 0.11 g, 0.33 mmol, 1.1 equiv.) and Ru(Me$_2$-Pheox) (**10**; 1.8 mg, 3.0 µmol, 0.01 equiv.) in DCM (0.1 M) for 0.5 h at room temperature. The crude was purified by flash chromatography on SiO_2 (pentane/EtOAc = 9:1) to afford compound **9x** (0.15 g, 0.28 mmol, 95%).

Appearance: colourless oil.

TLC: R$_f$ = 0.5 (Pentane/EtOAc = 7:3; UV-active and stains in $KMnO_4$).

^{1}H-NMR (400 MHz, $CDCl_3$) δ (ppm) = 7.88 – 7.83 (m, 2H), 7.79 – 7.74 (m, 2H), 5.57 (d, *J* = 8.6 Hz, 1H), 4.43 – 4.37 (m, 1H), 4.25 (dd, *J* = 10.0, 2.9 Hz, 1H), 4.05 (dd, *J* = 10.0, 3.0 Hz, 1H), 3.73 (s, 3H), 2.27 (d, *J* = 1.0 Hz, 2H), 1.38 (s, 9H), 1.31 – 1.20 (m, 2H), 1.08 (dd, *J* = 7.3, 3.1 Hz, 12H).

^{13}C-NMR (101 MHz, $CDCl_3$) δ (ppm) = 171.0, 168.2, 162.1, 155.5, 134.7, 129.0, 123.9, 79.7, 64.6, 55.5, 52.3, 28.3, 17.8, 17.04, 17.03, 17.02, 16.96, 12.6, 12.5.

HRMS: (ESI-TOF) m/*z* : [M+Na]$^+$ Calcd for $C_{25}H_{36}N_2O_9SiNa$ 559.2080; Found 559.2083.

*Synthesis of 1,3-dioxoisoindolin-2-yl (E)-2-(((2,7-dimethylocta-2,6-dien-1-yl)oxy)diisopropylsilyl)acetate (**9v**)*

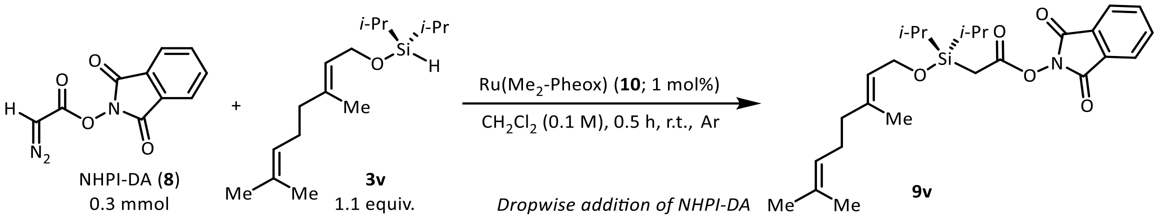

General procedure E was applied using NHPI-DA (**8**; 69 mg, 0.30 mmol, 1.0 equiv.), (*E*)-((2,7-dimethylocta-2,6-dien-1-yl)oxy)diisopropylsilane (**3v**; 89 mg, 0.33 mmol, 1.1 equiv.) and Ru(Me$_2$-Pheox) (**10**; 1.8 mg, 3.0 µmol, 0.01 equiv.) in DCM (0.1 M) for 0.5 h at room temperature. The crude was purified by flash chromatography on SiO_2 (pentane/EtOAc = 9:1) to afford compound **9v** (0.12 g, 0.26 mmol, 88%).

Appearance: colourless oil.

TLC: R$_f$ = 0.65 (Pentane/EtOAc = 9:1; UV-active and stains in $KMnO_4$).

^{1}H-NMR (400 MHz, $CDCl_3$) δ (ppm) = 7.89 – 7.83 (m, 2H), 7.79 – 7.74 (m, 2H), 5.37 – 5.33 (m, 1H), 5.11 – 5.06 (m, 1H), 4.39 – 4.34 (m, 2H), 2.33 (s, 2H), 2.13 – 2.05 (m, 2H), 2.05 – 1.98 (m, 2H), 1.67 (d, *J* = 1.0 Hz, 3H), 1.63 (d, *J* = 1.0 Hz, 3H), 1.59 (d, *J* = 0.7 Hz, 3H), 1.36 – 1.23 (m, 2H), 1.13 (dd, *J* = 7.4, 2.2 Hz, 12H).

^{13}C-NMR (101 MHz, $CDCl_3$) δ (ppm) = 168.6, 162.1, 137.6, 134.6, 131.6, 129.0, 124.0, 123.8, 123.7, 60.9, 39.5, 26.3, 25.7, 18.1, 17.7, 17.19, 17.17, 16.4, 12.8.

HRMS: (ESI-TOF) m/*z* : [M+Na]$^+$ Calcd for $C_{26}H_{37}NO_5SiNa$ 494.2333; Found 494.2333.

*Synthesis of 1,3-dioxoisoindolin-2-yl 2-(diisopropyl(phenoxy)silyl)acetate (**9w**)*

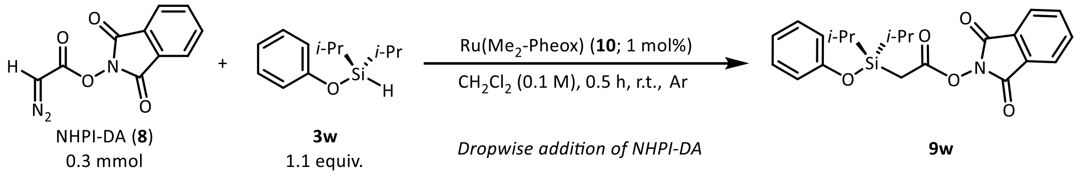

General procedure E was applied using NHPI-DA (**8**; 69 mg, 0.30 mmol, 1.0 equiv.), diisopropyl(phenoxy)silane (**3w**; 68 mg, 0.33 mmol, 1.1 equiv.) and Ru(Me$_2$-Pheox) (**10**; 1.8 mg, 3.0 µmol, 0.01 equiv.) in DCM (0.1 M) for 0.5 h at room temperature. The crude was purified by flash chromatography on SiO_2 (pentane/EtOAc = 10:1) to afford compound **9w** (0.10 g, 0.25 mmol, 84%).

Appearance: colourless oil.

TLC: R$_f$ = 0.4 (Pentane/EtOAc = 9:1; UV-active and stains in $KMnO_4$).

^{1}H-NMR (400 MHz, $CDCl_3$) δ (ppm) = 7.90 – 7.85 (m, 2H), 7.80 – 7.75 (m, 2H), 7.28 – 7.21 (m, 2H), 7.02 – 6.92 (m, 3H), 2.45 (s, 2H), 1.45 (hept, *J* = 7.4 Hz, 2H), 1.18 (dd, *J* = 7.4, 3.6 Hz, 12H).

^{13}C-NMR (101 MHz, $CDCl_3$) δ (ppm) = 168.0, 162.1, 154.6, 134.7, 129.6, 129.0, 123.9, 122.1, 120.0, 18.2, 17.0, 13.2.

HRMS: (ESI-TOF) m/*z* : $[M+Na]^+$ Calcd for $C_{22}H_{25}NO_5SiNa$ 434.1394; Found 434.1398.

*Synthesis of tert-butyl 5-(((2-((1,3-dioxoisoindolin-2-yl)oxy)-2-oxoethyl)diisopropylsilyl)oxy)-1H-indole-1-carboxylate (**9x**)*

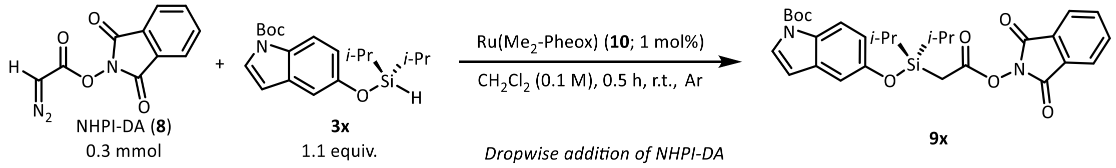

General procedure E was applied using NHPI-DA (**8**; 69 mg, 0.30 mmol, 1.0 equiv.), *tert*-butyl 5-((diisopropylsilyl)oxy)-1*H*-indole-1-carboxylate (**3x**; 0.11 g, 0.33 mmol, 1.1 equiv.) and Ru(Me_2-Pheox) (**10**; 1.8 mg, 3.0 µmol, 0.01 equiv.) in DCM (0.1 M) for 0.5 h at room temperature. The crude was purified by flash chromatography on SiO_2 (pentane/EtOAc = 9:1) to afford compound **9x** (0.14 g, 0.25 mmol, 83%).

Appearance: colourless oil

TLC: R_f = 0.58 (Pentane/EtOAc = 8:2; UV-active and stains red in vanillin).

^{1}H-NMR (400 MHz, $CDCl_3$) δ (ppm) = 7.99 (d, *J* = 7.6 Hz, 1H), 7.89 – 7.84 (m, 2H), 7.79 – 7.74 (m, 2H), 7.56 (d, *J* = 3.4 Hz, 1H), 7.11 (d, *J* = 2.3 Hz, 1H), 6.94 (dd, *J* = 8.9, 2.4 Hz, 1H), 6.48 (d, *J* = 3.3 Hz, 1H), 2.47 (s, 2H), 1.66 (s, 9H), 1.48 (hept, *J* = 7.4 Hz, 2H), 1.19 (dd, *J* = 7.5, 4.2 Hz, 12H).

^{13}C-NMR (101 MHz, $CDCl_3$) δ (ppm) = 168.1, 162.1, 150.3, 149.7, 134.6, 131.6, 130.7, 129.0, 126.7, 123.9, 117.3, 115.8, 110.9, 107.1, 83.5, 28.2, 18.2, 17.1, 13.2.

HRMS: (ESI-TOF) m/*z* : $[M+Na]^+$ Calcd for $C_{29}H_{34}N_2O_7SiNa$ 573.2027; Found 573.2029.

*Synthesis of redox-active ester derived from 1,1,1,3,3,3-hexamethyl-2-(trimethylsilyl)trisilane (**9y**)*

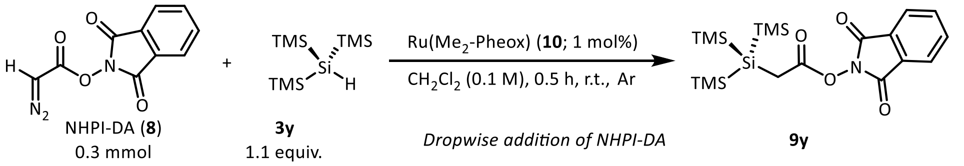

General procedure E was applied using NHPI-DA (**8**; 69 mg, 0.30 mmol, 1.0 equiv.), 1,1,1,3,3,3-hexamethyl-2-(trimethylsilyl)trisilane (**3y**; 82 mg, 0.33 mmol, 1.1 equiv.) and Ru(Me_2-Pheox) (**10**; 1.8 mg, 3.0 µmol, 0.01 equiv.) in DCM (0.1 M) for 0.5 h at room temperature. The crude was purified by flash chromatography on SiO_2 (pentane/EtOAc = 9:1) to afford compound **9y** (0.12 g, 0.28 mmol, 88%).

Appearance: white solid.

m.p.: 110.8 – 113.5 °C

TLC: R_f = 0.45 (Pentane/EtOAc = 9:1; UV-active and stains in $KMnO_4$).

^{1}H-NMR (400 MHz, $CDCl_3$) δ (ppm) = 7.89 – 7.84 (m, 2H), 7.78 – 7.75 (m, 2H), 2.25 (s, 2H), 0.26 (s, 27H).

^{13}C-NMR (101 MHz, $CDCl_3$) δ (ppm) = 170.7, 162.0, 134.6, 129.0, 123.8, 12.2, 0.6.

HRMS: (ESI-TOF) m/*z* : $[M+Na]^+$ Calcd for $C_{19}H_{33}NO_4Si_4Na$ 474.1379; Found 474.1376.

*Synthesis of redox-active ester derived from diethylsilane (**9z**)*

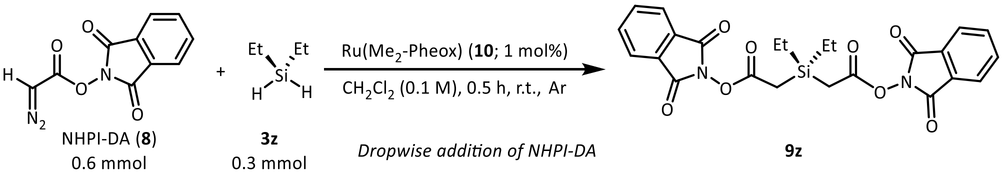

General procedure E was applied using NHPI-DA (**8**; 0.14 g, 0.60 mmol, 2.0 equiv.), diethylsilane (**3z**; 26 mg, 0.30 mmol, 1.0 equiv.) and Ru(Me_2-Pheox) (**10**; 1.8 mg, 3.0 µmol, 0.01 equiv.) in DCM (0.1 M) for 0.5 h at room temperature. The crude was purified by flash chromatography on SiO_2 (pentane/EtOAc = 9:1) to afford compound **9z** (0.14 g, 0.28 mmol, 95%).

Appearance: colourless oil.

TLC: R_f = 0.4 (Pentane/EtOAc = 8:2; UV-active and stains in $KMnO_4$).

^{1}H-NMR (400 MHz, $CDCl_3$) δ (ppm) = 7.91 – 7.85 (m, 4H), 7.81 – 7.76 (m, 4H), 2.52 (s, 4H), 1.16 –1.11 (m, 6H), 1.09 – 1.01 (m, 4H).

^{13}C-NMR (101 MHz, $CDCl_3$) δ (ppm) = 168.0, 162.1, 134.7, 129.0, 123.9, 18.6, 6.7, 3.6.

HRMS: (ESI-TOF) m/*z* : $[M+Na]^+$ Calcd for $C_{24}H_{22}N_2O_8SiNa$ 517.1038; Found 517.1048.

*Synthesis of redox-active ester derived from 1,1,1,3,3,3-hexamethyl-2-(trimethylsilyl)trisilane (**9aa**)*

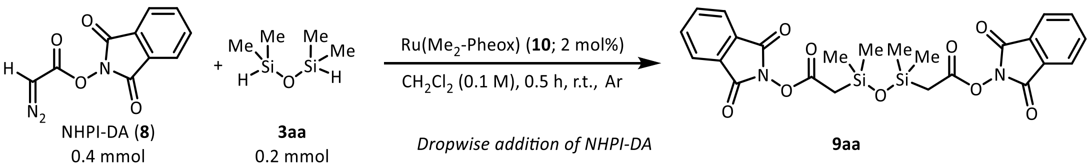

General procedure E was applied using NHPI-DA (**8**; 92 mg, 0.40 mmol, 2.0 equiv.), tetramethyldisiloxane (**3aa**; 27 mg, 02 mmol, 1.0 equiv.) and Ru(Me_2-Pheox) (**10**; 2.3 mg, 4.0 µmol, 0.02 equiv.) in DCM (0.1 M) for 0.5 h at room temperature. The crude was purified by washing with MeOH (3 × 2 mL) to afford compound **9aa** (86 mg, 0.16 mmol, 80%).

Appearance: white solid.

TLC: Decomposes on silica gel, not detectable.

m.p: 161.8 – 163.8 °C

^{1}H-NMR (400 MHz, $CDCl_3$) δ (ppm) = 7.87 – 7.85 (m, 4H), 7.80 – 7.76 (m, 4H), 2.32 (s, 4H), 0.41 (s, 12H).

^{13}C-NMR (101 MHz, $CDCl_3$) δ (ppm) = 167.8, 162.2, 134.6, 129.0, 123.9, 25.3, 0.3.

HRMS: (ESI-TOF) m/*z* : $[M+Na]^+$ Calcd for $C_{24}H_{24}N_2O_9Si_2Na$ 563.0913; Found 563.0914.

4. Diversification of the α-Silyl Redox-Active Ester Products

4.1 General procedure F: One-pot methylborylation of silanes using NHPI-DA (8)

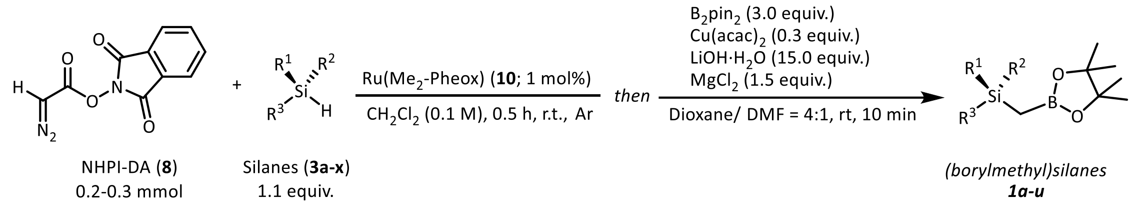

The procedure has been adapted from the one reported by Baran and co-workers.[9]

A flame-dried vial was charged with a stirring bar and silane (**3a-x**; 1.1 equiv.). The vial was evacuated and refilled with argon (three cycles), followed by the addition of a solution of Ru(Me_2-Pheox) (**10**; 0.01 equiv.) in dry DCM. The mixture was stirred for 5 min at room temperature, followed by dropwise addition (over a period of 1 to 2 min) of a solution of *N*-hydroxyphthalimidoyl diazoacetate (**8**; NHPI-DA; 0.2-0.3 mmol, 1.0 equiv.) in dry DCM. The resulting mixture was stirred for 30 min at room temperature. Upon completion, DCM was evaporated under reduced pressure and the remaining solvent was dried using high vacuum and the crude was used for the next step without further purification.

To the vial containing the crude redox-active ester of silane (1.0 equiv.) were added B_2pin_2 (3.0 equiv.), $Cu(acac)_2$ (30 mol%), $LiOH{\cdot}H_2O$ (15 equiv.) and $MgCl_2$ (1.5 equiv.). The vial was evacuated and backfilled with argon (3 times), followed by addition of degassed dioxane/DMF (4:1, 0.14 M). The resulting mixture was stirred vigorously at room temperature for 10 min until a dark brown color was observed. Upon completion, the reaction mixture was diluted with Et_2O (10 mL) and transferred to a 20 mL vial containing saturated aqueous NH_4Cl solution (5 mL), and the resulting mixture was shaken vigorously until a clear biphasic solution was observed. The organic phase was collected and the aqueous phase was extracted with Et_2O (2 x 10 mL). The combined organic layer was dried over anhydrous Na_2SO_4 and concentrated under reduced pressure. The crude was purified by flash chromatography on SiO_2 (pentane to pentane/EtOAc = 98:2) to afford the (borylmethyl)silanes **1a-u**.

4.2 General procedure G: One-pot methylborylation of dimethylsilanes using a photo-induced approach

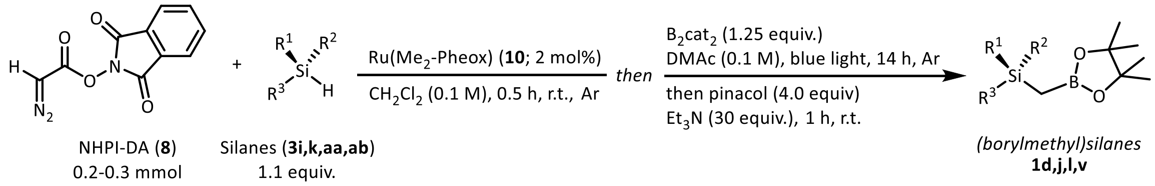

The procedure has been adapted from the one reported by Aggarwal and co-workers.[10]

A flame-dried vial was charged with a stirring bar and silanes (**3i,k,aa,ab**; 1.1 equiv.). The vial was evacuated and refilled with argon (three cycles), followed by addition of a solution of Ru(Me_2-Pheox) (**10**; 0.01 equiv.) in dry DCM. The mixture was stirred for 5 min at room temperature and then a solution of *N*-hydroxyphthalimidyl diazoacetate (**8**; NHPI-DA; 0.2-0.3 mmol; 1.0 equiv.) in dry DCM was added dropwise (over a period of 1 to 2 min). The resulting mixture was stirred for 30 min at room temperature. Upon completion, DCM was evaporated under reduced pressure and the remaining solvent was dried using high vacuum and the crude was used for the next step without further purification.

To the vial containing the crude redox-active ester of silane (1 equiv.) were added B_2cat_2 (1.25 equiv.) and anhydrous DMAc (0.1 M). Next, the headspace of the vial was purged with argon for 15-20 sec. Then the vial was closed with a cap fitted with a rubber septum. The resulting mixture was irradiated with 450 nm blue light for 14 h. Upon completion, pinacol (4.0 equiv.) dissolved in triethylamine (30.0 equiv.) was added and the resulting mixture was stirred for 1 h at room temperature. The reaction mixture was diluted with diethyl ether (10 mL) and transferred to a 20 mL vial containing saturated NH_4Cl (5 mL) and water (5 mL). The resulting mixture was stirred vigorously until a clear biphasic solution was obtained. The organic layer was separated and the aqueous layer was extracted with diethylether (2 x 10 mL). The combined organic layer was dried over anhydrous Na_2SO_4 and concentrated under reduced pressure. The crude was purified by flash chromatography on SiO_2 (pentane to pentane/EtOAc = 98:2) to afford the (borylmethyl)silanes **1d,j,l,v**.

*Synthesis of triisopropyl((4,4,5,5-tetramethyl-1,3,2-dioxaborolan-2-yl)methyl)silane (**1a**)*

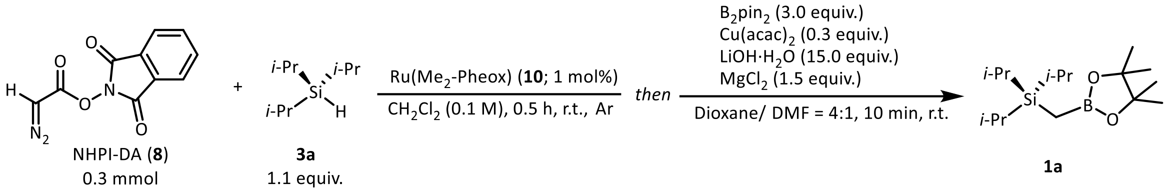

General procedure F was applied using NHPI-DA (**8**; 69 mg, 0.30 mmol, 1.0 equiv.), triisopropylsilane (**3a**; 52 mg, 0.33 mmol, 1.1 equiv.) and Ru(Me_2-Pheox) (**10**; 1.8 mg, 3.0 µmol, 0.01 equiv.) in DCM (0.1 M) for 30 min at room temperature to obtain the crude redox-active ester. Then, B_2pin_2 (0.23 g, 0.90 mmol, 3.0 equiv.), $Cu(acac)_2$ (24 mg, 90 µmol, 30 mol%), $LiOH\cdot H_2O$ (0.19 g, 4.5 mmol, 15 equiv.) and $MgCl_2$ (43 mg, 0.45 mmol, 1.5 equiv.) were added and stirred in dioxane/DMF (4:1, 2.1 mL, 0.14 M) for 10 min at room temperature. The crude was purified by flash chromatography on SiO_2 (pentane to pentane/EtOAc = 98:2) to afford compound **1a** (51 mg, 0.17 mmol, 57%).

Appearance: colourless oil.

TLC: R_f = 0.75 (Pentane/EtOAc = 98:2; stains blue in vanillin).

^{1}H-NMR (400 MHz, $CDCl_3$) δ (ppm) = 1.22 (s, 12H), 1.06 – 0.98 (m, 21H), 0.00 (s, 2H).

^{13}C-NMR (101 MHz, $CDCl_3$) δ (ppm) = 82.6, 25.0, 18.7, 12.1.

HRMS: (ESI-TOF) m/*z* : $[M+Na]^+$ Calcd for $C_{16}H_{35}BO_2SiNa$ 321.2395; Found 321.2397.

*Synthesis of triethyl((4,4,5,5-tetramethyl-1,3,2-dioxaborolan-2-yl)methyl)silane (**1b**)*

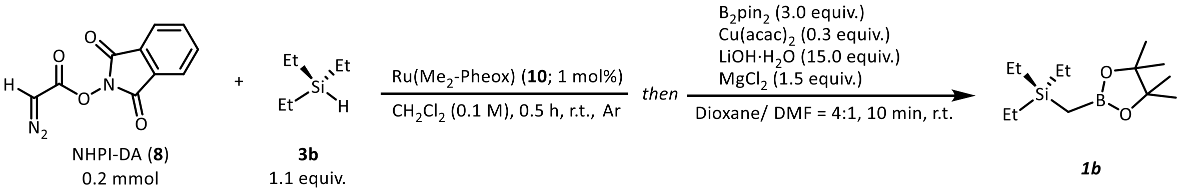

General procedure F was applied using NHPI-DA (**8**; 46 mg, 0.20 mmol, 1.0 equiv.), triethylsilane (**3b**; 26 mg, 0.22 mmol, 1.1 equiv.) and Ru(Me_2-Pheox) (**10**; 1.2 mg, 2.0 µmol, 0.01 equiv.) in DCM (0.1 M) for 30 min at room temperature to obtain the crude redox-active ester. Then, B_2pin_2 (0.15 g, 0.60 mmol, 3.0 equiv.), $Cu(acac)_2$ (16 mg, 60 µmol, 30 mol%), $LiOH\cdot H_2O$ (0.13 g, 3.0 mmol, 15 equiv.) and $MgCl_2$ (29 mg, 0.30 mmol, 1.5 equiv.) were added and stirred in dioxane/DMF (4:1, 1.4 mL, 0.14 M) for 10 min at room temperature. The crude was purified by flash chromatography on SiO_2 (pentane to pentane/EtOAc = 98:2) to afford compound **1b** (31 mg, 0.12 mmol, 60%).

Appearance: colourless oil.

TLC: R_f = 0.6 (Pentane/EtOAc = 98:2; stains blue in vanillin).

^{1}H-NMR (400 MHz, $CDCl_3$) δ (ppm) = 1.23 (s, 12H), 0.94 (t, *J* = 7.9 Hz, 9H), 0.54 (q, *J* = 7.9 Hz, 6H), 0.04 (s, 2H).

^{13}C-NMR (101 MHz, $CDCl_3$) δ (ppm) = 82.6, 24.9, 7.4, 5.0.

HRMS: (ESI-TOF) m/*z* : $[M+NH_4]^+$ Calcd for $C_{13}H_{29}BO_2SiNH_4$ 274.2368; Found 274.2357.

*Synthesis of benzyldiisopropyl((4,4,5,5-tetramethyl-1,3,2-dioxaborolan-2-yl)methyl)silane (**1c**)*

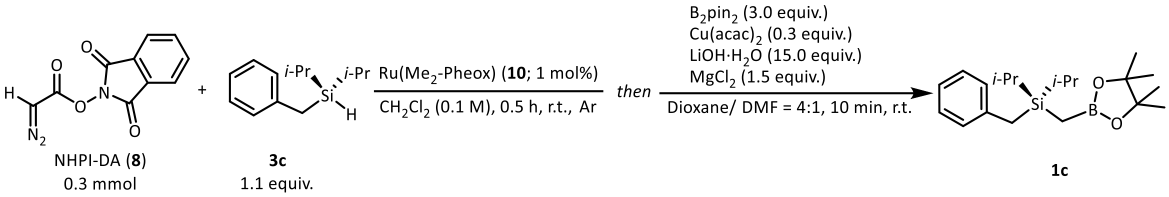

General procedure F was applied using NHPI-DA (**8**; 69 mg, 0.30 mmol, 1.0 equiv.), benzyldiisopropylsilane (**3c**; 68 mg, 0.33 mmol, 1.1 equiv.) and Ru(Me_2-Pheox) (**10**; 1.8 mg, 3.0 µmol, 0.01 equiv.) in DCM (0.1 M) for 30 min at room temperature to obtain the crude redox-active ester. Then, B_2pin_2 (0.23 g, 0.90 mmol, 3.0 equiv.), $Cu(acac)_2$ (24 mg, 90 µmol, 30 mol%), $LiOH·H_2O$ (0.19 g, 4.5 mmol, 15 equiv.) and $MgCl_2$ (43 mg, 0.45 mmol, 1.5 equiv.) were added and stirred in dioxane/DMF (4:1, 2.1 mL, 0.14 M) for 10 min at room temperature. The crude was purified by flash chromatography on SiO_2 (pentane to pentane/EtOAc = 98:2) to afford compound **1c** (69 mg, 0.20 mmol, 66%).

Appearance: colourless oil.

TLC: R_f = 0.6 (Pentane/EtOAc = 98:2; stains blue in vanillin).

^{1}H-NMR (400 MHz, $CDCl_3$) δ (ppm) = 7.21 – 7.15 (m, 2H), 7.15 – 7.11 (m, 2H), 7.07 – 7.01 (m, 1H), 2.20 (s, 2H), 1.24 (s, 12H), 0.97 (s, 14H), 0.03 (s, 2H).

^{13}C-NMR (101 MHz, $CDCl_3$) δ (ppm) = 140.8, 128.6, 128.1, 123.8, 82.7, 25.0, 21.4, 18.12, 18.06, 12.3.

HRMS: (ESI-TOF) m/*z* : $[M+Na]^+$ Calcd for $C_{20}H_{35}BO_2SiNa$ 369.2396; Found 369.2391.

*Synthesis of benzyldimethyl((4,4,5,5-tetramethyl-1,3,2-dioxaborolan-2-yl)methyl)silane (**1d**)*

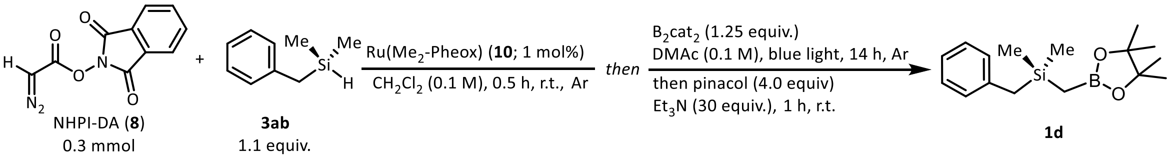

General procedure G was applied using NHPI-DA (**8**; 69 mg, 0.30 mmol, 1.0 equiv.), benzyldimethylsilane (**3ab**; 50 mg, 0.33 mmol, 1.1 equiv.) and Ru(Me_2-Pheox) (**10**; 1.8 mg, 3.0 µmol, 0.01 equiv.) in DCM (0.1 M) for 30 min at room temperature to obtain the crude redox-active ester. Then, B_2cat_2 (89 mg, 0.37 mmol, 1.25 equiv.), pinacol (0.14 g, 1.2 mmol, 4.0 equiv.) and Et_3N (1.3 mL, 9.0 mmol, 30 equiv.) were added and stirred in DMAc (0.1 M) under blue light irradiation for 14 h at room temperature. The crude was purified by flash chromatography on SiO_2 (pentane to pentane/EtOAc = 98:2) to afford compound **1d** (57 mg, 0.20 mmol, 65%).

Appearance: colourless oil.

TLC: R_f = 0.75 (Pentane/EtOAc = 98:2; stains blue in vanillin).

^{1}H-NMR (400 MHz, $CDCl_3$) δ (ppm) = 7.20 (t, *J* = 7.5 Hz, 2H), 7.06 (d, *J* = 7.3 Hz, 1H), 7.02 (d, *J* = 7.6 Hz, 2H), 2.12 (s, 2H), 1.24 (s, 12H), 0.09 (s, 2H), 0.02 (s, 6H).

^{13}C-NMR (101 MHz, $CDCl_3$) δ (ppm) = 140.2, 128.2, 128.1, 123.9, 82.8, 27.3, 25.0, -1.8.

All the data are in accordance with the literature.[11]

*Synthesis of diisopropyl(pent-4-en-1-yl)((4,4,5,5-tetramethyl-1,3,2-dioxaborolan-2-yl)methyl)silane (**1e**)*

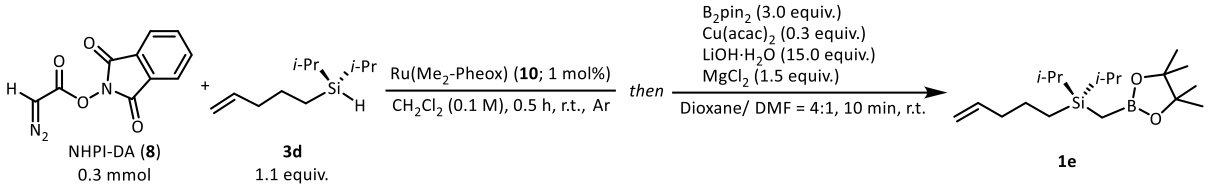

General procedure F was applied using NHPI-DA (**8**; 69 mg, 0.30 mmol, 1.0 equiv.), diisopropyl(pent-4-en-1-yl)silane (**3d**; 61 mg, 0.33 mmol, 1.1 equiv.) and Ru(Me_2-Pheox) (**10**; 1.8 mg, 3.0 µmol, 0.01 equiv.) in DCM (0.1 M) for 30 min at room temperature to obtain the crude redox-active ester. Then, B_2pin_2 (0.23 g, 0.90 mmol, 3.0 equiv.), $Cu(acac)_2$ (24 mg, 90 µmol, 30 mol%), $LiOH·H_2O$ (0.19 g, 4.5 mmol, 15 equiv.) and $MgCl_2$ (43 mg, 0.45 mmol, 1.5 equiv.) were added and stirred in dioxane/DMF (4:1, 2.1 mL, 0.14 M) for 10 min at room temperature. The crude was purified by flash chromatography on SiO_2 (pentane to pentane/EtOAc = 98:2) to afford compound **1e** (33 mg, 0.10 mmol, 34%).

Appearance: colourless oil.

TLC: R_f = 0.55 (Pentane/EtOAc = 98:2; stains blue in vanillin).

^{1}H-NMR (400 MHz, $CDCl_3$) δ (ppm) = 5.80 (ddt, *J* = 17.0, 10.2, 6.7 Hz, 1H), 5.07 – 4.87 (m, 2H), 2.09 – 2.04 (m, 2H), 1.51 – 1.40 (m, 2H), 1.22 (s, 12H), 1.07 – 0.96 (m, 14H), 0.64 – 0.54 (m, 2H), 0.00 (s, 2H).

^{13}C-NMR (101 MHz, $CDCl_3$) δ (ppm) = 139.1, 114.4, 82.6, 38.2, 25.0, 23.6, 18.27, 18.26, 12.4, 11.2.

HRMS: (ESI-TOF) m/*z* : $[M+Na]^+$ Calcd for $C_{18}H_{37}BO_2SiNa$ 347.2552; Found 347.2558.

*Synthesis of (4-chlorobutyl)diisopropyl((4,4,5,5-tetramethyl-1,3,2-dioxaborolan-2-yl)methyl)silane (**1f**)*

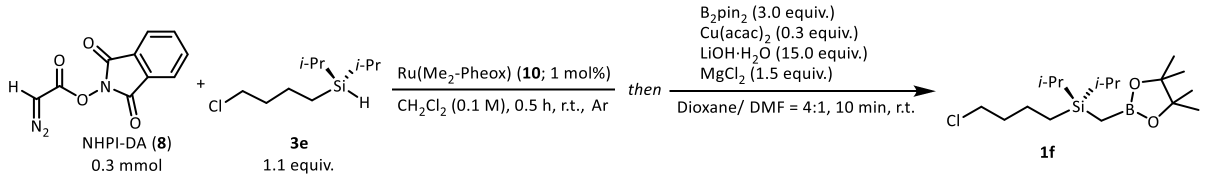

General procedure F was applied using NHPI-DA (**8**; 69 mg, 0.30 mmol, 1.0 equiv.), (4-chlorobutyl)diisopropylsilane (**3e**; 68 mg, 0.33 mmol, 1.1 equiv.) and Ru(Me_2-Pheox) (**10**; 1.8 mg, 3.0 µmol, 0.01 equiv.) in DCM (0.1 M) for 30 min at room temperature to obtain the crude redox-active ester. Then, B_2pin_2 (0.23 g, 0.90 mmol, 3.0 equiv.), $Cu(acac)_2$ (24 mg, 90 µmol, 30 mol%), $LiOH·H_2O$ (0.19 g, 4.5 mmol, 15 equiv.) and $MgCl_2$ (43 mg, 0.45 mmol,

1.5 equiv.) were added and stirred in dioxane/DMF (4:1, 2.1 mL, 0.14 M) for 10 min at room temperature. The crude was purified by flash chromatography on SiO_2 (pentane to pentane/EtOAc = 98:2) to afford compound **1f** (64 mg, 0.18 mmol, 61%).

Appearance: colourless oil.

TLC: R_f = 0.5 (Pentane/EtOAc = 98:2; stains blue in vanillin).

^{1}H-NMR (400 MHz, $CDCl_3$) δ (ppm) = 3.55 (t, *J* = 6.7 Hz, 2H), 1.79 (p, *J* = 6.9 Hz, 2H), 1.51 (dtd, *J* = 11.6, 8.5, 6.3 Hz, 2H), 1.22 (s, 12H), 1.03 – 0.88 (m, 14H), 0.62 – 0.54 (m, 2H), 0.01 (s, 2H).

^{13}C-NMR (101 MHz, $CDCl_3$) δ (ppm) = 82.6, 44.7, 36.7, 25.0, 21.4, 18.3, 12.4, 10.8.

HRMS: (ESI-TOF) m/*z* : $[M+Na]^+$ Calcd for $C_{17}H_{36}BClO_2SiNa$ 369.2162; Found 369.2167.

*Synthesis of (3-(benzyloxy)propyl)diisopropyl((4,4,5,5-tetramethyl-1,3,2-dioxaborolan-2-yl)methyl)silane (**1g**)*

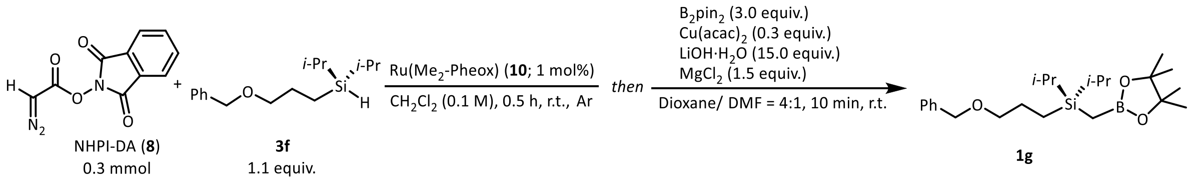

General procedure F was applied using NHPI-DA (**8**; 69 mg, 0.30 mmol, 1.0 equiv.), (3-(benzyloxy)propyl)diisopropylsilane (**3f**; 98 mg, 0.33 mmol, 1.1 equiv.) and Ru(Me_2-Pheox) (**10**; 1.8 mg, 3.0 µmol, 0.01 equiv.) in DCM (0.1 M) for 30 min at room temperature to obtain the crude redox-active ester. Then, B_2pin_2 (0.23 g, 0.90 mmol, 3.0 equiv.), $Cu(acac)_2$ (24 mg, 90 µmol, 30 mol%), $LiOH{\cdot}H_2O$ (0.19 g, 4.5 mmol, 15 equiv.) and $MgCl_2$ (43 mg, 0.45 mmol, 1.5 equiv.) were added and stirred in dioxane/DMF (4:1, 2.1 mL, 0.14 M) for 10 min at room temperature. The crude was purified by flash chromatography on SiO_2 (pentane to pentane/EtOAc = 98:2) to afford compound **1g** (49 mg, 0.12 mmol, 40%).

Appearance: colourless oil.

TLC: R_f = 0.4 (Pentane/EtOAc = 98:2; stains blue in vanillin).

^{1}H-NMR (400 MHz, $CDCl_3$) δ (ppm) = 7.35 – 7.26 (m, 5H), 4.51 (s, 2H), 3.43 (t, *J* = 7.0 Hz, 2H), 1.68 (dt, *J* = 14.3, 7.2 Hz, 2H), 1.21 (s, 12H), 1.07 – 0.90 (m, 14H), 0.64 – 0.54 (m, 2H), 0.01 (s, 2H).

^{13}C-NMR (101 MHz, $CDCl_3$) δ (ppm) = 138.8, 128.3, 127.6, 127.4, 82.6, 73.8, 72.7, 25.0, 24.3, 18.3, 12.4, 7.4.

HRMS: (ESI-TOF) m/*z* : $[M+Na]^+$ Calcd for $C_{23}H_{41}BO_3SiNa$ 427.2815; Found 427.2816.

*Synthesis of diisopropyl((4,4,5,5-tetramethyl-1,3,2-dioxaborolan-2-yl)methyl)(vinyl)silane (**1h**)*

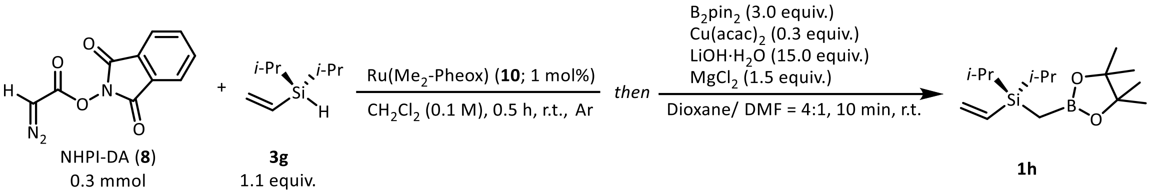

General procedure F was applied using NHPI-DA (**8**; 69 mg, 0.30 mmol, 1.0 equiv.), diisopropyl(vinyl)silane (**3g**; 47 mg, 0.33 mmol, 1.1 equiv.) and Ru(Me_2-Pheox) (**10**; 1.8 mg, 3.0 µmol, 0.01 equiv.) in DCM (0.1 M) for 30 min at room temperature to obtain the crude redox-active ester. Then, B_2pin_2 (0.23 g, 0.90 mmol, 3.0 equiv.), $Cu(acac)_2$ (24 mg, 90 µmol, 30 mol%), $LiOH{\cdot}H_2O$ (0.19 g, 4.5 mmol, 15 equiv.) and $MgCl_2$ (43 mg, 0.45 mmol, 1.5 equiv.) were added and stirred in dioxane/DMF (4:1, 2.1 mL, 0.14 M) for 10 min at room temperature. The crude was purified by flash chromatography on SiO_2 (pentane to pentane/EtOAc = 98:2) to afford compound **1h** (35 mg, 0.12 mmol, 41%).

Appearance: colourless oil.

TLC: R_f = 0.45 (Pentane/EtOAc = 99:1; stains blue in vanillin).

^{1}H-NMR (400 MHz, $CDCl_3$) δ (ppm) = 6.15 – 5.99 (m, 2H), 5.76 (dd, *J* = 18.7, 5.7 Hz, 1H), 1.22 (s, 12H), 1.09 – 0.92 (m, 14H), 0.09 (s, 2H).

^{13}C-NMR (101 MHz, $CDCl_3$) δ (ppm) = 135.5, 133.1, 82.7, 24.9, 18.0, 17.8, 11.9.

HRMS: (ESI-TOF) m/*z* : $[M+Na]^+$ Calcd for $C_{15}H_{31}BO_2SiNa$ 305.2082; Found 305.2088.

*Synthesis of diisopropyl(phenyl)((4,4,5,5-tetramethyl-1,3,2-dioxaborolan-2-yl)methyl)silane (**1i**)*

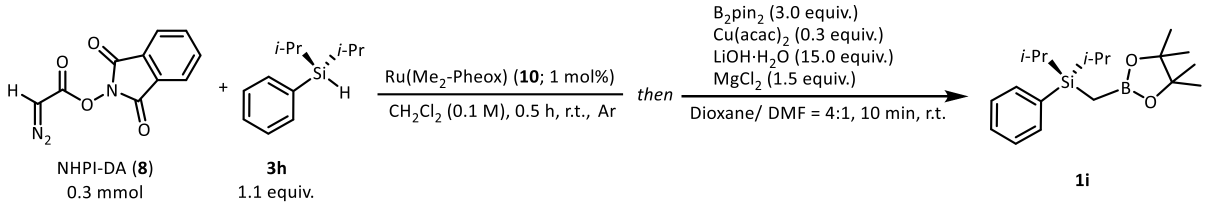

General procedure F was applied using NHPI-DA (**8**; 69 mg, 0.30 mmol, 1.0 equiv.), diisopropyl(phenyl)silane (**3h**; 63 mg, 0.33 mmol, 1.1 equiv.) and Ru(Me_2-Pheox) (**10**; 1.8 mg, 3.0 µmol, 0.01 equiv.) in DCM (0.1 M) for 30 min at room temperature to obtain the crude redox-active ester. Then, B_2pin_2 (0.23 g, 0.90 mmol, 3.0 equiv.), $Cu(acac)_2$ (24 mg, 90 µmol, 30 mol%), $LiOH{\cdot}H_2O$ (0.19 g, 4.5 mmol, 15 equiv.) and $MgCl_2$ (43 mg, 0.45 mmol, 1.5 equiv.) were added and stirred in dioxane/DMF (4:1, 2.1 mL, 0.14 M) for 10 min at room temperature. The crude was purified by flash chromatography on SiO_2 (pentane to pentane/EtOAc = 98:2) to afford compound **1i** (63 mg, 0.19 mmol, 63%).

Appearance: colourless oil.

TLC: R_f = 0.55 (Pentane/EtOAc = 98:2; stains blue in vanillin).

^{1}**H-NMR** (400 MHz, $CDCl_3$) δ (ppm) = 7.60 – 7.53 (m, 2H), 7.35 – 7.29 (m, 3H), 1.29 – 1.19 (m, 2H), 1.19 (s, 12H), 1.04 (d, *J* = 7.3 Hz, 6H), 0.98 (d, *J* = 7.4 Hz, 6H), 0.33 (s, 2H).

^{13}**C-NMR** (101 MHz, $CDCl_3$) δ (ppm) = 136.2, 134.9, 128.5, 127.3, 82.8, 24.9, 18.0, 17.8, 12.0.

HRMS: (ESI-TOF) m/*z* : $[M+Na]^+$ Calcd for $C_{19}H_{33}BO_2SiNa$ 355.2239; Found 355.2236.

*Synthesis of dimethyl(phenyl)((4,4,5,5-tetramethyl-1,3,2-dioxaborolan-2-yl)methyl)silane (**1j**)*

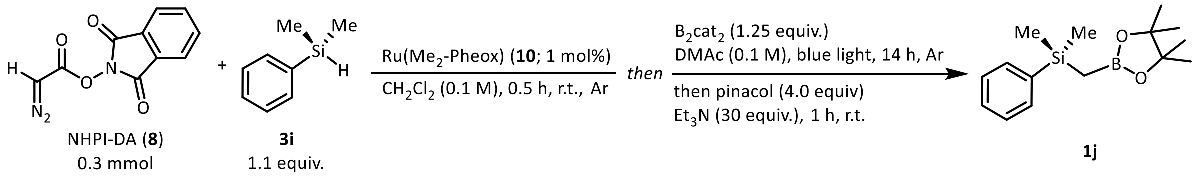

General procedure G was applied using NHPI-DA (**8**; 69 mg, 0.30 mmol, 1.0 equiv.), dimethyl(phenyl)silane (**3i**; 45 mg, 0.33 mmol, 1.1 equiv.) and Ru(Me_2-Pheox) (**10**; 1.8 mg, 3.0 µmol, 0.01 equiv.) in DCM (0.1 M) for 30 min at room temperature to obtain the crude redox-active ester. Then, B_2cat_2 (89 mg, 0.37 mmol, 1.25 equiv.), pinacol (0.14 g, 1.2 mmol, 4.0 equiv.) and Et_3N (1.3 mL, 9.0 mmol, 30 equiv.) were added and stirred in DMAc (0.1 M) under blue light irradiation for 14 h at room temperature. The crude was purified by flash chromatography on SiO_2 (pentane to pentane/EtOAc = 98:2) to afford compound **1j** (52 mg, 0.19 mmol, 63%).

Appearance: colourless oil.

TLC: R_f = 0.65 (Pentane/EtOAc = 98:2; stains blue in vanillin).

^{1}**H-NMR** (400 MHz, $CDCl_3$) δ (ppm) = 7.58 – 7.52 (m, 2H), 7.37 – 7.31 (m, 3H), 1.18 (s, 12H), 0.36 (s, 2H), 0.33 (s, 6H).

^{13}**C-NMR** (101 MHz, $CDCl_3$) δ (ppm) = 140.2, 133.4, 128.8, 127.6, 82.8, 24.9, -0.9.

All the data are in accordance with the literature.[13]

*Synthesis of (4-fluorophenyl)diisopropyl((4,4,5,5-tetramethyl-1,3,2-dioxaborolan-2-yl)methyl)silane (**1k**)*

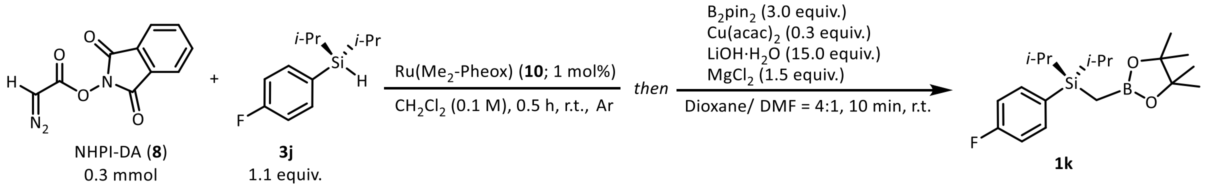

General procedure F was applied using NHPI-DA (**3**; 69 mg, 0.30 mmol, 1.0 equiv.), (4-fluorophenyl)diisopropylsilane (**3j**; 69 mg, 0.33 mmol, 1.1 equiv.) and Ru(Me_2-Pheox) (**10**; 1.8 mg, 3.0 µmol, 0.01 equiv.) in DCM (0.1 M) for 30 min at room temperature to obtain the crude redox-active ester. Then, B_2pin_2 (0.23 g, 0.90 mmol, 3.0 equiv.), $Cu(acac)_2$ (24 mg, 90 µmol, 30 mol%), $LiOH{\cdot}H_2O$ (0.19 g, 4.5 mmol, 15 equiv.) and $MgCl_2$ (43 mg, 0.45 mmol, 1.5 equiv.) were added and stirred in dioxane/DMF (4:1, 2.1 mL, 0.14 M) for 10 min at room temperature. The crude was purified by flash chromatography on SiO_2 (pentane to pentane/EtOAc = 98:2) to afford compound **1k** (56 mg, 0.16 mmol, 53%).

Appearance: colourless oil.

TLC: R_f = 0.53 (Pentane/EtOAc = 98:2; stains blue in vanillin).

^{1}H-NMR (400 MHz, $CDCl_3$) δ (ppm) = 7.56 – 7.51 (m, 2H), 7.05 – 7.00 (m, 2H), 1.25 – 1.15 (m, 14H), 1.02 (d, *J* = 7.3 Hz, 6H), 0.96 (d, *J* = 7.3 Hz, 6H), 0.31 (s, 2H).

^{13}C-NMR (101 MHz, $CDCl_3$) δ (ppm) = 164.8 (d, $J_{C\text{-}F}$ = 247.8 Hz), 136.8 (d, $J_{C\text{-}F}$ = 7.4 Hz), 131.7 (d, $J_{C\text{-}F}$ = 3.9 Hz), 114.3(d, $J_{C\text{-}F}$ = 19.2 Hz), 82.8, 24.9, 17.9, 17.7, 12.0.

^{19}F-NMR (377 MHz, $CDCl_3$) δ (ppm) = –112.96.

HRMS: (ESI-TOF) m/*z* : $[M+Na]^+$ Calcd for $C_{19}H_{32}BFO_2SiNa$ 373.2145; Found 373.2143.

*Synthesis of (4-chlorophenyl)dimethyl((4,4,5,5-tetramethyl-1,3,2-dioxaborolan-2-yl)methyl)silane (**1l**)*

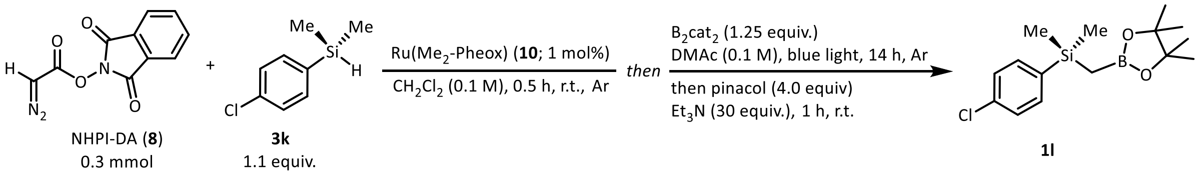

General procedure G was applied using NHPI-DA (**8**; 69 mg, 0.30 mmol, 1.0 equiv.), (4-chlorophenyl)dimethylsilane (**3k**; 56 mg, 0.33 mmol, 1.1 equiv.) and Ru(Me_2-Pheox) (**10**; 1.8 mg, 3.0 µmol, 0.01 equiv.) in DCM (0.1 M) for 30 min at room temperature to obtain the crude redox-active ester. Then, B_2cat_2 (89 mg, 0.37 mmol, 1.25 equiv.), pinacol (0.14 g, 1.2 mmol, 4.0 equiv.) and Et_3N (1.3 mL, 9.0 mmol, 30 equiv.) were added and stirred in DMAc (0.1 M) under blue light irradiation for 14 h at room temperature. The crude was purified by flash chromatography on SiO_2 (pentane to pentane/EtOAc = 98:2) to afford compound **1l** (56 mg, 0.18 mmol, 60%).

Appearance: colourless oil.

TLC: R_f = 0.5 (Pentane/EtOAc = 98:2; stains blue in vanillin).

^{1}H-NMR (400 MHz, $CDCl_3$) δ (ppm) = 7.47 (d, *J* = 8.1 Hz, 2H), 7.31 (d, *J* = 8.1 Hz, 2H), 1.18 (s, 12H), 0.34 (s, 2H), 0.31 (s, 6H).

^{13}C-NMR (101 MHz, $CDCl_3$) δ (ppm) = 138.5, 135.1, 134.9, 127.8, 82.9, 24.9, -0.9.

HRMS: (ESI-TOF) m/*z* : $[M+Na]^+$ Calcd for $C_{15}H_{24}BClO_2SiNa$ 333.1222; Found 333.1222.

*Synthesis of ((4-(diisopropyl((4,4,5,5-tetramethyl-1,3,2-dioxaborolan-2-yl)methyl)silyl)phenyl)ethynyl)trimethylsilane (**1m**)*

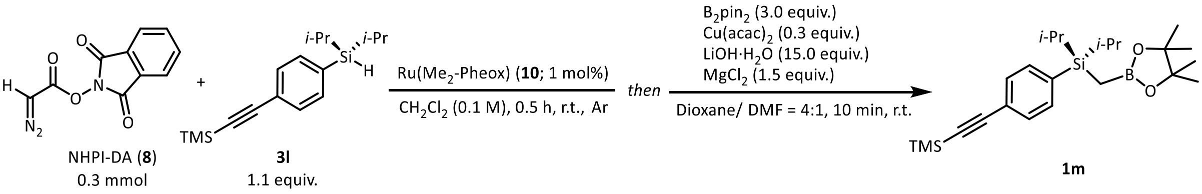

General procedure F was applied using NHPI-DA (**8**; 69 mg, 0.30 mmol, 1.0 equiv.), ((4-(diisopropylsilyl)phenyl)ethynyl)trimethylsilane (**3l**; 95 mg, 0.33 mmol, 1.1 equiv.) and

Ru(Me$_2$-Pheox) (**10**; 1.8 mg, 3.0 µmol, 0.01 equiv.) in DCM (0.1 M) for 30 min at room temperature to obtain the crude redox-active ester. Then, B$_2$pin$_2$ (0.23 g, 0.90 mmol, 3.0 equiv.), Cu(acac)$_2$ (24 mg, 90 µmol, 30 mol%), LiOH·H$_2$O (0.19 g, 4.5 mmol, 15 equiv.) and MgCl$_2$ (43 mg, 0.45 mmol, 1.5 equiv.) were added and stirred in dioxane/DMF (4:1, 2.1 mL, 0.14 M) for 10 min at room temperature. The crude was purified by flash chromatography on SiO$_2$ (pentane to pentane/EtOAc = 98:2) to afford compound **1m** (67 mg, 0.16 mmol, 52%).

Appearance: colourless oil.

TLC: R_f = 0.55 (Pentane/EtOAc = 99:1; stains blue in vanillin).

^{1}H NMR (400 MHz, CDCl$_3$) δ (ppm) = 7.50 (d, *J* = 7.9 Hz, 2H), 7.41 (d, *J* = 7.9 Hz, 2H), 1.26 – 1.18 (s, 14H), 1.01 (d, *J* = 7.3 Hz, 6H), 0.94 (d, *J* = 7.4 Hz, 6H), 0.31 (s, 2H), 0.24 (s, 9H).

^{13}C NMR (101 MHz, CDCl$_3$) δ (ppm) = 137.4, 134.7, 130.6, 123.0, 105.4, 94.4, 82.8, 24.9, 17.9, 17.7, 12.0, 0.0.

HRMS: (ESI-TOF) m/*z* : [M+Na]$^+$ Calcd for $C_{24}H_{41}BO_2Si_2Na$ 451.2634; Found 451.2635.

*Synthesis of diisopropyl((4,4,5,5-tetramethyl-1,3,2-dioxaborolan-2-yl)methyl)(thiophen-2-yl)silane (**1n**)*

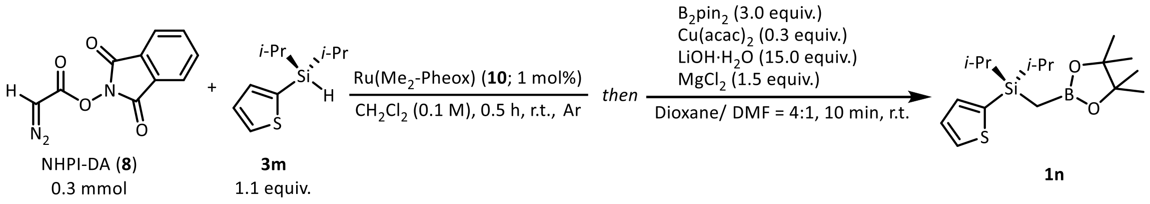

General procedure F was applied using NHPI-DA (**8**; 69 mg, 0.30 mmol, 1.0 equiv.), diisopropyl(thiophen-2-yl)silane (**3m**; 66 mg, 0.33 mmol, 1.1 equiv.) and Ru(Me$_2$-Pheox) (**10**; 1.8 mg, 3.0 µmol, 0.01 equiv.) in DCM (0.1 M) for 30 min at room temperature to obtain the crude redox-active ester. Then, B$_2$pin$_2$ (0.23 g, 0.90 mmol, 3.0 equiv.), Cu(acac)$_2$ (24 mg, 90 µmol, 30 mol%), LiOH·H$_2$O (0.19 g, 4.5 mmol, 15 equiv.) and MgCl$_2$ (43 mg, 0.45 mmol, 1.5 equiv.) were added and stirred in dioxane/DMF (4:1, 2.1 mL, 0.14 M) for 10 min at room temperature. The crude was purified by flash chromatography on SiO$_2$ (pentane to pentane/EtOAc = 98:2) to afford compound **1n** (55 mg, 0.16 mmol, 54%).

Appearance: colourless oil.

TLC: R_f = 0.5 (Pentane/EtOAc = 98:2; stains blue in vanillin).

^{1}H-NMR (400 MHz, CDCl$_3$) δ (ppm) = 7.59 (d, *J* = 4.6 Hz, 1H), 7.36 (d, *J* = 3.2 Hz, 1H), 7.19 (t, *J* = 3.9 Hz, 1H), 1.21 (s, 14H), 1.04 (dd, *J* = 13.2, 7.3 Hz, 12H), 0.38 (s, 2H).

^{13}C-NMR (101 MHz, CDCl$_3$) δ (ppm) = 135.7, 135.3, 130.3, 127.7, 82.9, 24.9, 17.9, 17.7, 12.9.

HRMS: (ESI-TOF) m/*z* : [M+Na]$^+$ Calcd for $C_{17}H_{31}BO_2SSiNa$ 361.1803; Found 361.1804.

*Synthesis of triphenyl((4,4,5,5-tetramethyl-1,3,2-dioxaborolan-2-yl)methyl)silane (**1o**)*

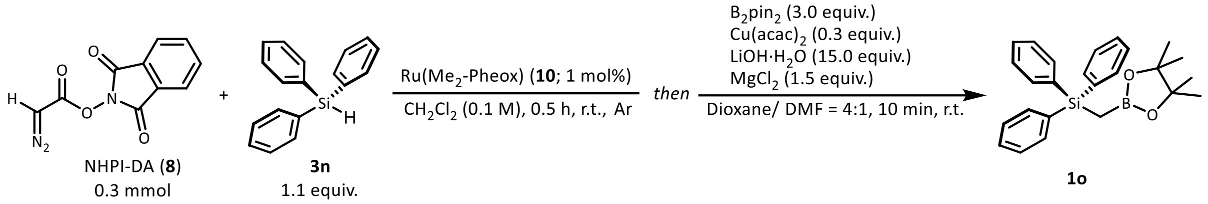

General procedure F was applied using NHPI-DA (**8**; 69 mg, 0.30 mmol, 1.0 equiv.), triphenylsilane (**3n**; 86 mg, 0.33 mmol, 1.1 equiv.) and Ru(Me$_2$-Pheox) (**10**; 1.8 mg, 3.0 µmol, 0.01 equiv.) in DCM (3 mL, 0.1 M) for 30 min at room temperature to obtain the crude redox-active ester. Then, B$_2$pin$_2$ (0.23 g, 0.90 mmol, 3.0 equiv.), Cu(acac)$_2$ (24 mg, 90 µmol, 30 mol%), LiOH·H$_2$O (0.19 g, 4.5 mmol, 15 equiv.) and MgCl$_2$ (43 mg, 0.45 mmol, 1.5 equiv.) were added and stirred in dioxane/DMF (4:1, 2.1 mL, 0.14 M) for 10 min at room temperature. The crude was purified by flash chromatography on SiO$_2$ (pentane to pentane/EtOAc = 98:2) to afford compound **1o** (44 mg, 0.11 mmol, 36%).

Appearance: white solid.

TLC: R$_f$ = 0.65 (Pentane/EtOAc = 98:2; stains blue in vanillin).

m.p.: 94-5 – 98.3 °C

^{1}H-NMR (400 MHz, CDCl$_3$) δ (ppm) = 7.58 – 7.56 (m, 6H), 7.40 – 7.32 (m, 9H), 1.01 (s, 12H), 0.95 (s, 2H).

^{13}C-NMR (101 MHz, CDCl$_3$) δ (ppm) = 136.2, 135.7, 129.2, 127.6, 83.0, 24.7.

HRMS: (ESI-TOF) m/*z* : [M+Na]$^+$ Calcd for $C_{25}H_{29}BO_2SiNa$ 423.1927; Found 423.1923.

*Synthesis of 1,1,1,3,3,3-hexamethyl-2-((4,4,5,5-tetramethyl-1,3,2-dioxaborolan-2-yl)methyl)-2-(trimethylsilyl)trisilane (**1p**)*

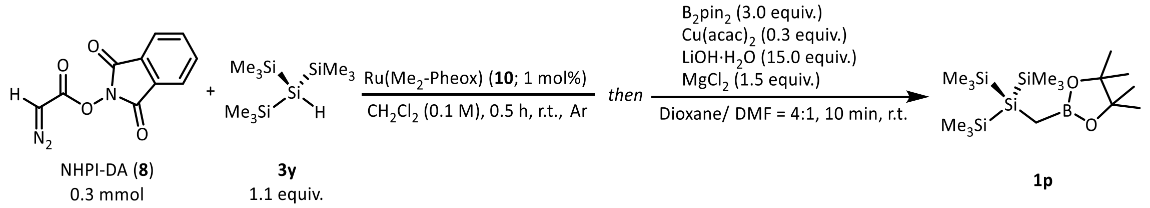

General procedure F was applied using NHPI-DA (**8**; 69 mg, 0.30 mmol, 1.0 equiv.), 1,1,1,3,3,3-hexamethyl-2-(trimethylsilyl)trisilane (**3y**; 42 mg, 0.33 mmol, 1.1 equiv.) and Ru(Me$_2$-Pheox) (**10**; 1.8 mg, 3.0 µmol, 0.01 equiv.) in DCM (0.1 M) for 30 min at room temperature to obtain the crude redox-active ester. Then, B$_2$pin$_2$ (0.23 g, 0.90 mmol, 3.0 equiv.), Cu(acac)$_2$ (24 mg, 90 µmol, 30 mol%), LiOH·H$_2$O (0.19 g, 4.5 mmol, 15 equiv.) and MgCl$_2$ (43 mg, 0.45 mmol, 1.5 equiv.) were added and stirred in dioxane/DMF (4:1, 2.1 mL, 0.14 M) for 10 min at room temperature. The crude was purified by flash chromatography on SiO$_2$ (pentane to pentane/EtOAc = 98:2) to afford compound **1p** (51 mg, 0.13 mmol, 44%).

Appearance: colourless oil.

TLC: R$_f$ = 0.45 (Pentane/EtOAc = 98:2; stains blue in vanillin).

^{1}**H-NMR** (400 MHz, $CDCl_3$) δ (ppm) = 1.23 (s, 12H), 0.16 (s, 27H), 0.04 (s, 2H).

^{13}**C-NMR** (101 MHz, $CDCl_3$) δ (ppm) = 82.7, 25.2, 0.7.

HRMS: (ESI-TOF) m/*z* : $[M+Na]^+$ Calcd for $C_{16}H_{41}BO_2Si_4Na$ 411.2173; Found 411.2170.

*Synthesis of (benzyloxy)diisopropyl((4,4,5,5-tetramethyl-1,3,2-dioxaborolan-2-yl)methyl)silane (**1q**)*

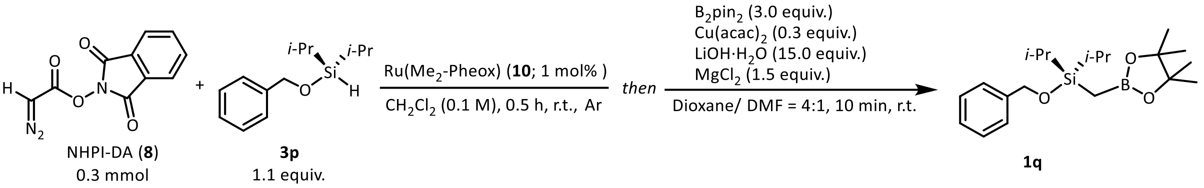

General procedure F was applied using NHPI-DA (**8**; 69 mg, 0.30 mmol, 1.0 equiv.), (benzyloxy)diisopropylsilane (**3p**; 73 mg, 0.33 mmol, 1.1 equiv.) and Ru(Me_2-Pheox) (**10**; 1.8 mg, 3.0 µmol, 0.01 equiv.) in DCM (0.1 M) for 30 min at room temperature to obtain the crude redox-active ester. Then, B_2pin_2 (0.23 g, 0.90 mmol, 3.0 equiv.), $Cu(acac)_2$ (24 mg, 90 µmol, 30 mol%), $LiOH·H_2O$ (0.19 g, 4.5 mmol, 15 equiv.) and $MgCl_2$ (43 mg, 0.45 mmol, 1.5 equiv.) were added and stirred in dioxane/DMF (4:1, 2.1 mL, 0.14 M) for 10 min at room temperature. The crude was purified by flash chromatography on SiO_2 (pentane to pentane/EtOAc = 98:2) to afford compound **1q** (55 mg, 0.15 mmol, 51%).

Appearance: colourless oil.

TLC: R_f = 0.55 (Pentane/EtOAc = 98:2; stains blue in vanillin).

^{1}**H-NMR** (400 MHz, $CDCl_3$) δ (ppm) = 7.39 – 7.34 (m, 2H), 7.34 – 7.29 (m, 2H), 7.25 – 7.20 (m, 1H), 4.83 (s, 2H), 1.21 (s, 12H), 1.09 – 1.03 (m, 14H), 0.25 (s, 2H).

^{13}**C-NMR** (101 MHz, $CDCl_3$) δ (ppm) = 141.6, 128.0, 126.6, 126.0, 82.8, 64.9, 24.9, 17.57, 17.55, 13.7.

HRMS: (ESI-TOF) m/*z* : $[M+Na]^+$ Calcd for $C_{20}H_{35}BO_3SiNa$ 385.2345; Found 385.2340.

*Synthesis of diisopropyl((4-methylpentan-2-yl)oxy)((4,4,5,5-tetramethyl-1,3,2-dioxaborolan-2-yl)methyl)silane (**1r**)*

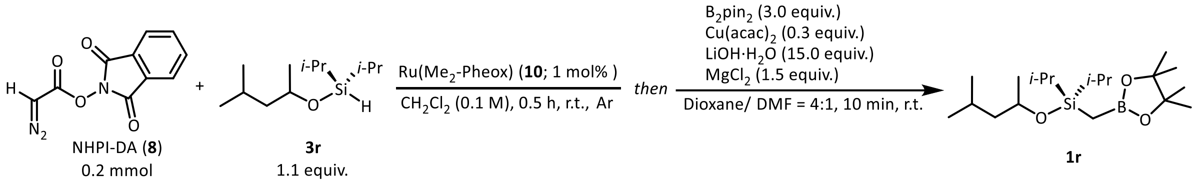

General procedure F was applied using NHPI-DA (**8**; 46 mg, 0.20 mmol, 1.0 equiv.), diisopropyl((4-methylpentan-2-yl)oxy)silane (**3r**; 47 mg, 0.22 mmol, 1.1 equiv.) and Ru(Me_2-Pheox) (**10**; 1.2 mg, 2.0 µmol, 0.01 equiv.) in DCM (0.1 M) for 30 min at room temperature to obtain the crude redox-active ester. Then, B_2pin_2 (0.15 g, 0.60 mmol, 3.0 equiv.), $Cu(acac)_2$ (16 mg, 60 µmol, 30 mol%), $LiOH·H_2O$ (0.13 g, 3.0 mmol, 15 equiv.) and $MgCl_2$ (29 mg, 0.30 mmol, 1.5 equiv.) were added and stirred in dioxane/DMF (4:1, 1.4 mL, 0.14 M) for

10 min at room temperature. The crude was purified by flash chromatography on SiO_2 (pentane to pentane/EtOAc = 98:2) to afford compound **1r** (20 mg, 56 µmol, 28%).

Appearance: colourless oil.

TLC: R_f = 0.55 (Pentane/EtOAc = 98:2; stains blue in vanillin).

^{1}H-NMR (400 MHz, $CDCl_3$) δ (ppm) = 3.95 (h, *J* = 6.2 Hz, 1H), 1.66 (dp, *J* = 13.4, 6.7 Hz, 1H), 1.41 (dt, *J* = 13.6, 6.8 Hz, 1H), 1.22 – 1.18 (m, 13H), 1.13 (d, *J* = 6.0 Hz, 3H), 1.05 – 1.00 (m, 12H), 0.98 – 0.91 (m, 2H), 0.90 – 0.85 (m, 6H), 0.17 (s, 2H).

^{13}C-NMR (101 MHz, $CDCl_3$) δ (ppm) = 82.7, 66.9, 49.3, 24.95, 24.92, 24.7, 23.9, 23.2, 22.8, 17.64, 17.62, 17.57, 14.2, 14.0.

HRMS: (ESI-TOF) m/*z* : $[M+Na]^+$ Calcd for $C_{19}H_{41}BO_3SiNa$ 379.2814; found 379.2840.

*Synthesis of methyl N-(tert-butoxycarbonyl)-O-(diisopropyl((4,4,5,5-tetramethyl-1,3,2-dioxaborolan-2-yl)methyl)silyl)-D-serinate (**1s**)*

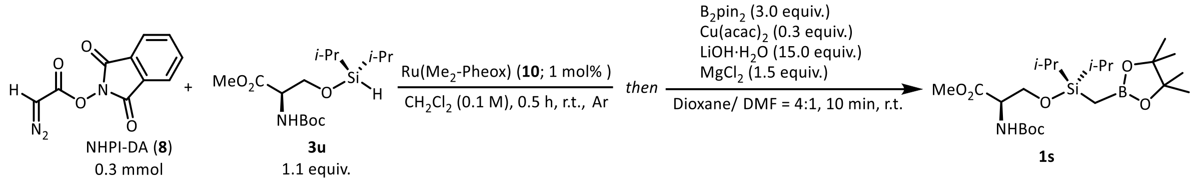

General procedure F was applied using NHPI-DA (**8**; 69 mg, 0.30 mmol, 1.0 equiv.), methyl *N*-(*tert*-butoxycarbonyl)-*O*-(diisopropylsilyl)-*D*-serinate (**3u**; 0.11 g, 0.33 mmol, 1.1 equiv.) and Ru(Me_2-Pheox) (**10**; 1.8 mg, 3.0 µmol, 0.01 equiv.) in DCM (0.1 M) for 30 min at room temperature to obtain the crude redox-active ester. Then, B_2pin_2 (0.23 g, 0.90 mmol, 3.0 equiv.), $Cu(acac)_2$ (24 mg, 90 µmol, 30 mol%), $LiOH{\cdot}H_2O$ (0.19 g, 4.5 mmol, 15 equiv.) and $MgCl_2$ (43 mg, 0.45 mmol, 1.5 equiv.) were added and stirred in dioxane/DMF (4:1, 2.1 mL, 0.14 M) for 10 min at room temperature. The crude was purified by flash chromatography on SiO_2 (pentane/EtOAc = 98:5) to afford compound **1s** (59 mg, 0.12 mmol, 41%).

Appearance: colourless oil.

TLC: R_f = 0.5 (Pentane/EtOAc = 9:1; stains blue in vanillin).

^{1}H-NMR (400 MHz, $CDCl_3$) δ (ppm) = 5.64 (d, *J* = 8.7 Hz, 1H), 4.36 (dt, *J* = 8.8, 3.0 Hz, 1H), 4.13 (dd, *J* = 9.9, 3.2 Hz, 1H), 3.92 (d, *J* = 3.1 Hz, 1H), 3.72 (s, 3H), 1.45 (s, 9H), 1.26 (s, 2H), 1.24 (d, *J* = 2.5 Hz, 12H), 1.01 – 0.96 (m, 12H), 0.13 (d, *J* = 5.3 Hz, 2H).

^{13}C-NMR (101 MHz, $CDCl_3$) δ (ppm) = 171.4, 155.7, 83.0, 79.7, 63.9, 55.7, 52.1, 28.4, 24.9, 24.8, 17.39, 17.36, 17.29, 13.5, 13.3.

HRMS: (ESI-TOF) m/*z* : $[M+Na]^+$ Calcd for $C_{22}H_{44}BNO_7SiNa$ 496.2877; Found 496.2874.

*Synthesis of diisopropyl(phenoxy)((4,4,5,5-tetramethyl-1,3,2-dioxaborolan-2-yl)methyl)silane (**1t**)*

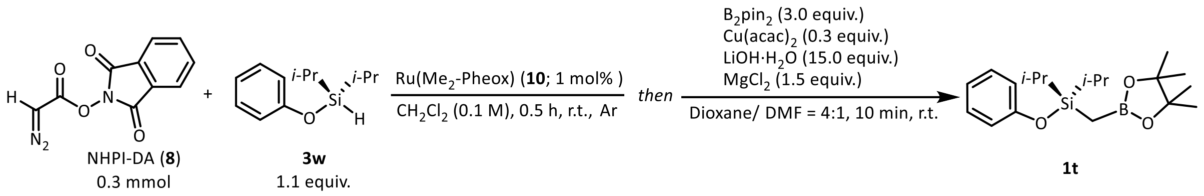

General procedure F was applied using NHPI-DA (**8**; 69 mg, 0.30 mmol, 1.0 equiv.), diisopropyl(phenoxy)silane (**3w**; 69 mg, 0.33 mmol, 1.1 equiv.) and Ru(Me$_2$-Pheox) (**10**; 1.8 mg, 3.0 µmol, 0.01 equiv.) in DCM (0.1 M) for 30 min at room temperature to obtain the crude redox-active ester. Then, B$_2$pin$_2$ (0.23 g, 0.90 mmol, 3.0 equiv.), Cu(acac)$_2$ (24 mg, 90 µmol, 30 mol%), LiOH·H$_2$O (0.19 g, 4.5 mmol, 15 equiv.) and MgCl$_2$ (43 mg, 0.45 mmol, 1.5 equiv.) were added and stirred in dioxane/DMF (4:1, 2.1 mL, 0.14 M) for 10 min at room temperature. The crude was purified by flash chromatography on SiO$_2$ (pentane to pentane/EtOAc = 98:2) to afford compound **1t** (38 mg, 0.12 mmol, 38%).

Appearance: colourless oil.

TLC: R$_f$ = 0.5 (Pentane/EtOAc = 98:2; stains blue in vanillin).

^{1}H-NMR (400 MHz, CDCl$_3$) δ (ppm) = 7.20 (t, *J* = 7.7 Hz, 2H), 6.93 – 6.89 (m, 3H), 1.20 (s, 12H), 1.17 – 1.04 (m, 14H), 0.30 (s, 2H).

^{13}C-NMR (101 MHz, CDCl$_3$) δ (ppm) = 155.7, 129.2, 121.1, 120.3, 82.9, 24.9, 17.4, 14.0.

HRMS: (ESI-TOF) m/*z* : [M+Na]$^+$ Calcd for $C_{19}H_{33}BO_3SiNa$ 371.2188; Found 371.2186.

*Synthesis of tert-butyl 5-((diisopropyl((4,4,5,5-tetramethyl-1,3,2-dioxaborolan-2-yl)methyl)silyl)oxy)-1H-indole-1-carboxylate (**1u**)*

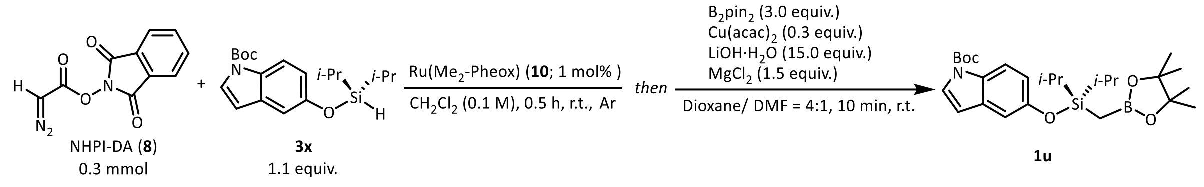

General procedure F was applied using NHPI-DA (**8**; 69 mg, 0.30 mmol, 1.0 equiv.), *tert*-butyl 5-((diisopropylsilyl)oxy)-1*H*-indole-1-carboxylate (**3x**; 80 mg, 0.33 mmol, 1.1 equiv.) and Ru(Me$_2$-Pheox) (**10**; 1.8 mg, 3.0 µmol, 0.01 equiv.) in DCM (0.1 M) for 30 min at room temperature to obtain the crude redox-active ester. Then, B$_2$pin$_2$ (0.23 g, 0.90 mmol, 3.0 equiv.), Cu(acac)$_2$ (24 mg, 90 µmol, 30 mol%), LiOH·H$_2$O (0.19 g, 4.5 mmol, 15 equiv.) and MgCl$_2$ (43 mg, 0.45 mmol, 1.5 equiv.) were added and stirred in dioxane/DMF (4:1, 2.1 mL, 0.14 M) for 10 min at room temperature. The crude was purified by flash chromatography on SiO$_2$ (pentane to pentane/EtOAc = 98:2) to afford compound **1u** (33 mg, 70 µmol, 32%).

Appearance: colourless oil.

TLC: R$_f$ = 0.45 (Pentane/EtOAc = 98:2; stains blue in vanillin).

^{1}**H-NMR** (400 MHz, $CDCl_3$) δ (ppm) = 7.93 (d, *J* = 6.8 Hz, 1H), 7.53 (d, *J* = 4.0 Hz, 1H), 7.07 (d, *J* = 2.2 Hz, 1H), 6.91 (dd, *J* = 8.9, 2.2 Hz, 1H), 6.44 (d, *J* = 3.6 Hz, 1H), 1.65 (s, 9H), 1.20 (s, 12H), 1.18 – 1.11 (m, 2H), 1.08 (t, *J* = 6.0 Hz, 12H), 0.32 (s, 2H).

^{13}**C-NMR** (101 MHz, $CDCl_3$) δ (ppm) = 151.5, 149.8, 131.4, 130.2, 126.3, 117.8, 115.4, 111.1, 107.1, 83.3, 82.9, 28.2, 24.9, 17.5, 17.4, 13.9.

HRMS: (ESI-TOF) m/*z* : $[M+Na]^+$ Calcd for $C_{26}H_{42}BNO_5SiNa$ 510.2822; Found 510.2820.

*Synthesis of 1,1,3,3-tetramethyl-1,3-bis((4,4,5,5-tetramethyl-1,3,2-dioxaborolan-2-yl)methyl)disiloxane (**1v**)*

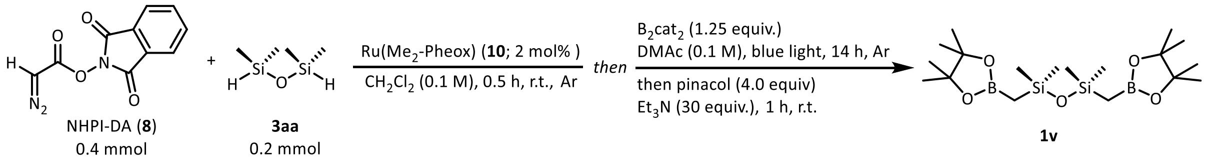

General procedure G was applied using NHPI-DA (**8**; 0.93 g, 0.40 mmol, 2.0 equiv.), 1,1,3,3-tetramethyldisiloxane (**3aa**; 27 mg, 0.20 mmol, 1. 0 equiv.) and Ru(Me_2-Pheox) (**10**; 2.3 mg, 4.0 µmol, 0.02 equiv.) in DCM (0.1 M) for 30 min at room temperature to obtain the crude redox-active ester. Then, B_2cat_2 (0.12 g, 0.50 mmol, 1.25 equiv.), pinacol (0.19 g, 1.6 mmol, 4.0 equiv.) and Et_3N (1.7 mL, 12 mmol, 30 equiv.) were added and stirred in DMAc (0.1 M) under blue light irradiation for 14 h at room temperature. The crude was purified by flash chromatography on SiO_2 (pentane to pentane/EtOAc = 98:2) to afford compound **1v** (26 mg, 63 µmol, 16%).

Appearance: colourless oil.

TLC: R_f = 0.55 (Pentane/EtOAc = 19:1; stains blue in vanillin).

^{1}**H-NMR** (400 MHz, $CDCl_3$) δ (ppm) = 1.22 (s, 24H), 0.21 (s, 4H), 0.12 (s, 12H).

^{13}**C-NMR** (101 MHz, $CDCl_3$) δ (ppm) = 82.7, 24.9, 2.2.

HRMS: (ESI-TOF) m/*z* : $[M+Na]^+$ Calcd for $C_{18}H_{40}B_2O_5Si_2Na$ 437.2500; found: 437.2502.

5. Scaled-up synthesis of triethyl((4,4,5,5-tetramethyl-1,3,2-dioxaborolan-2-yl)methyl)silane (1b)

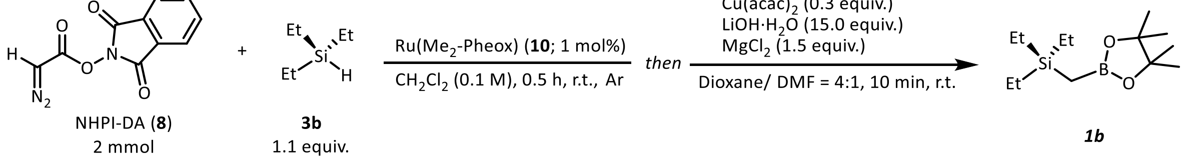

General procedure F was applied using NHPI-DA (**8**; 462 mg, 2 mmol, 1.0 equiv.), triethylsilane (**3b**; 256 mg, 2.2 mmol, 1.1 equiv.) and Ru(Me_2-Pheox) (**10**; 12 mg, 20 µmol, 0.01 equiv.) in DCM (0.1 M) for 30 min at room temperature to obtain the crude redox-active ester. Then, B_2pin_2 (1.54 g, 6 mmol,

3.0 equiv.), $Cu(acac)_2$ (162 mg, 0.6 mmol, 30 mol%), $LiOH{\cdot}H_2O$ (1.25 g, 30 mmol, 15 equiv.) and $MgCl_2$ (286 mg, 3 mmol, 1.5 equiv.) were added and stirred in dioxane/DMF (4:1, 14 mL, 0.14 M) for 10 min at room temperature. Upon completion, the reaction mixture was diluted with Et_2O (50 mL) and transferred to an extraction funnel containing saturated aqueous NH_4Cl solution (25 mL), and the resulting mixture was shaken vigorously until a clear biphasic solution was observed. The organic phase was collected and the aqueous phase was extracted with Et_2O (2 x 50 mL). The combined organic phases were dried over Na_2SO_4 and concentrated in vacuo. The crude was purified by flash chromatography on SiO_2 (pentane to pentane/EtOAc = 98:2) to afford compound **1b** (248 mg, 0.96 mmol, 48%).

The characterization data matched that reported in section 4.2 (see p. SI-28 and SI-29).

6. References

1. Krasovskiy, A.; Knochel, P. *Synthesis* **2006**, *5*, 0890-0891.

2. Moriguchi, T.; Moki, D.; Sekiguchi, T.; Kato, T.; Shinozuka, K. *Chem. Lett.* **2015**, *44*, 44-46.

3. Omann, L.; Pudasaini, B.; Irran, E.; Klare, H. F. T.; Baik, M. H.; Oestreich, M. *Chem. Sci.,* **2018**, *9,* 5600-5607.

4. Dutta, U.; Maiti, S.; Pimparkar, S.; Maiti, S.; Gahan, L. R.; Krenske, E. H.; Lupton, D. W.; Maiti, D. *Chem. Sci.* **2019**, *10*, 7426-7432.

5. Kan, S. B. J.; Lewis, R. D.; Chenand, K.; Arnold, F. H. *Science* **2016**, *354*, 1048-1051.

6. Lee, S.; Lee, H.; Tan, K. L. *J. Am. Chem. Soc.* **2013**, *135*, 18778–18781.

7. Wu, C.; Teo, W. J.; Ge, S. *ACS Catal.* **2018**, *8*, 5896–5900.

8. Huang, Z.; Kwon, O.; Huang, H.; Fadli, A.; Marat, X.; Moreau, M.; Lumb, J. P. *Angew. Chem. Int. Ed.* **2018**, *57*, 11963-11967.

9. Wang, J.; Shang, M.; Lundberg, H.; Feu, K. S.; Hecker, S. J.; Qin, T.; Blackmond, D. G.; Baran, P. S. *ACS Catal.* **2018,** *8*, 9537-9542.

10. Fawcett, A.; Pradeilles, J.; Wang, Y.; Mutsuga, T.; Myers, E. L.; Aggarwal, V. K. *Science* **2017,** *357*, 283-286.

11. Shu, C.; Noble, A.; Aggarwal, V. K. *Nature* **2020**, *586*, 714-719.

12. Montesinos-Magraner, M.; Costantini, M.; Ramírez-Contreras, R.; Muratore, M. E.; Johansson, M. J.; Mendoza, A. *Angew. Chem. Int. Ed.* **2019**, *58* (18), 5930-5935.

13. a) Chanthamath, S.; Thongjareun, S.; Shibatomi, K.; Iwasa, S. *Tetrahedron Lett.* **2012,** *53*, 4862-4865; b) Sun, Z.-C.; She, Y.-B.; Zhou, Y.; Song, X.-F.; Li, K. *Molecules* **2011**, 16, 2960-2970.

7. NMR Spectra of Synthesized Compounds

^{1}H-NMR (400 MHz, $CDCl_3$) for compound ***3d***

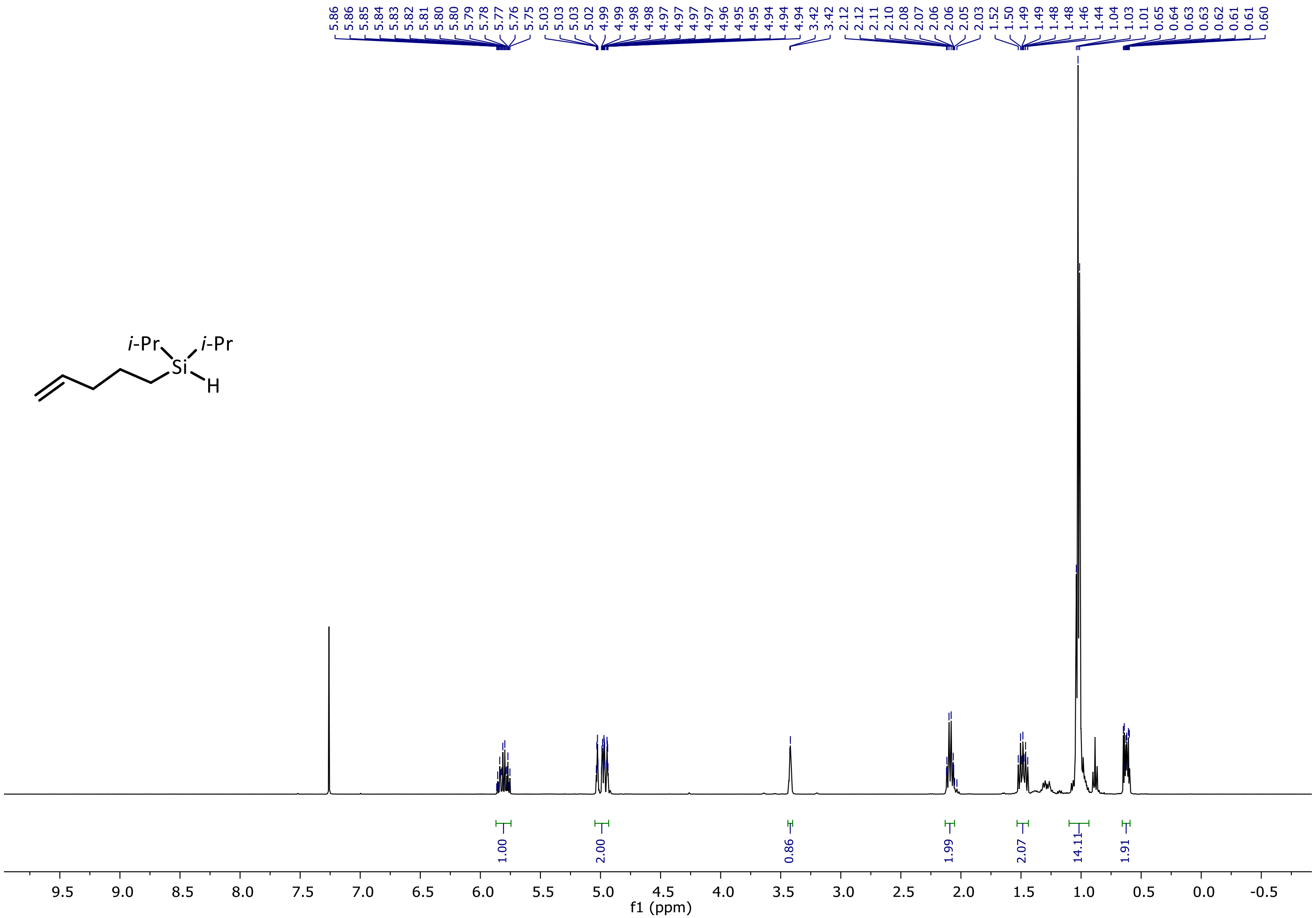

*^{13}C-NMR (101 MHz, $CDCl_3$) for compound **3d***

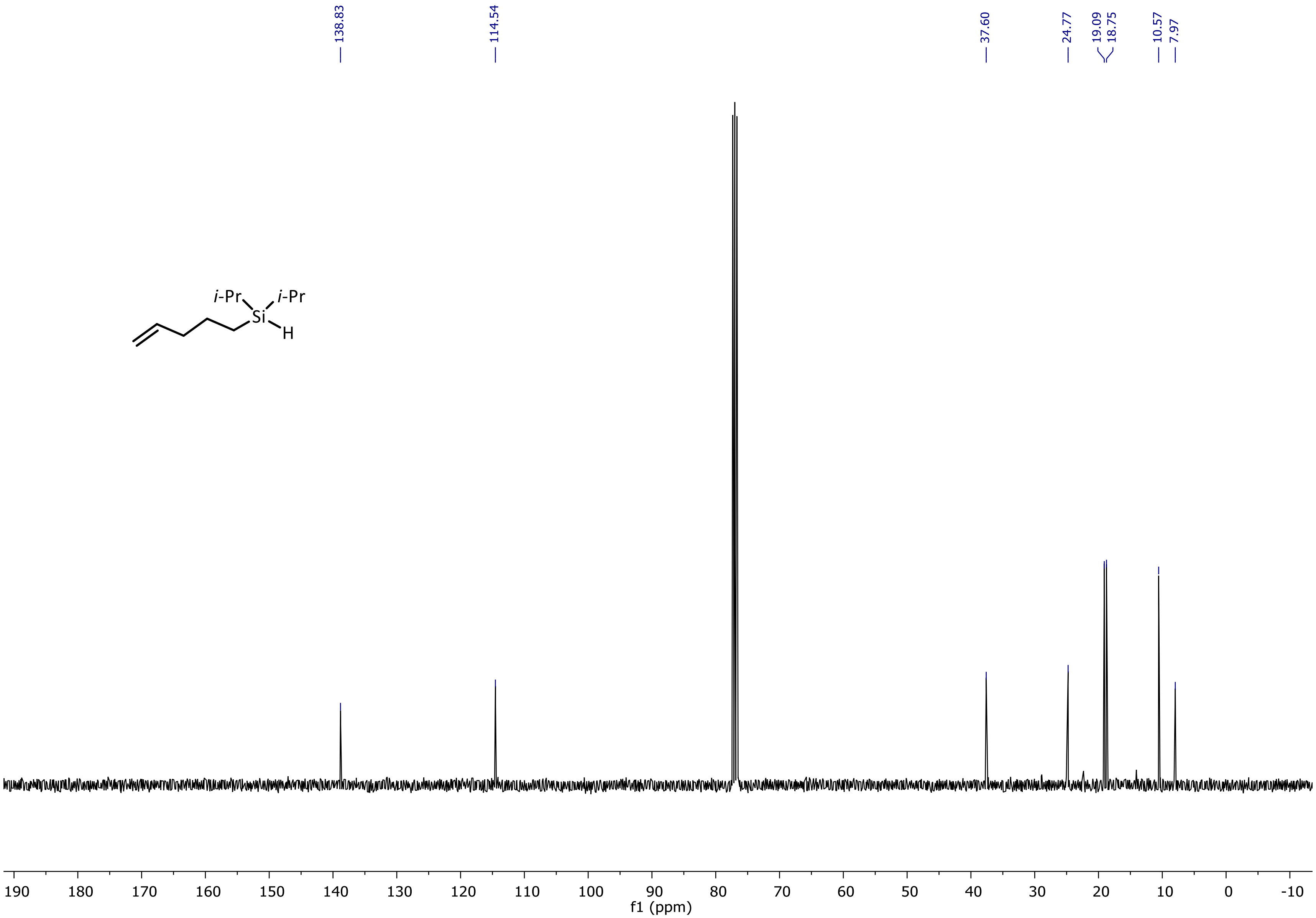

^{1}H-NMR (400 MHz, $CDCl_3$) for compound ***3e***

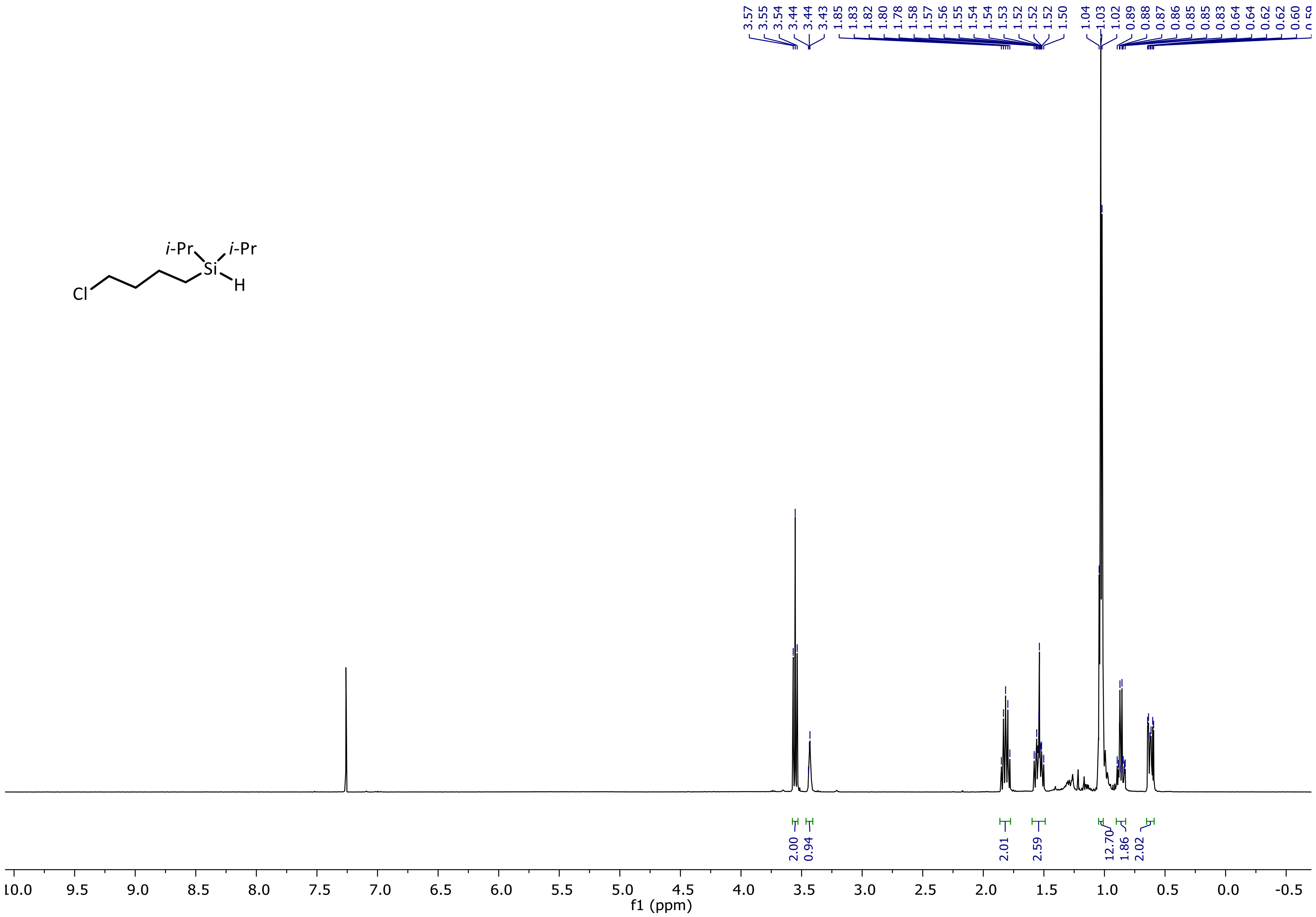

^{13}C-NMR (101 MHz, $CDCl_3$) for compound **3e**

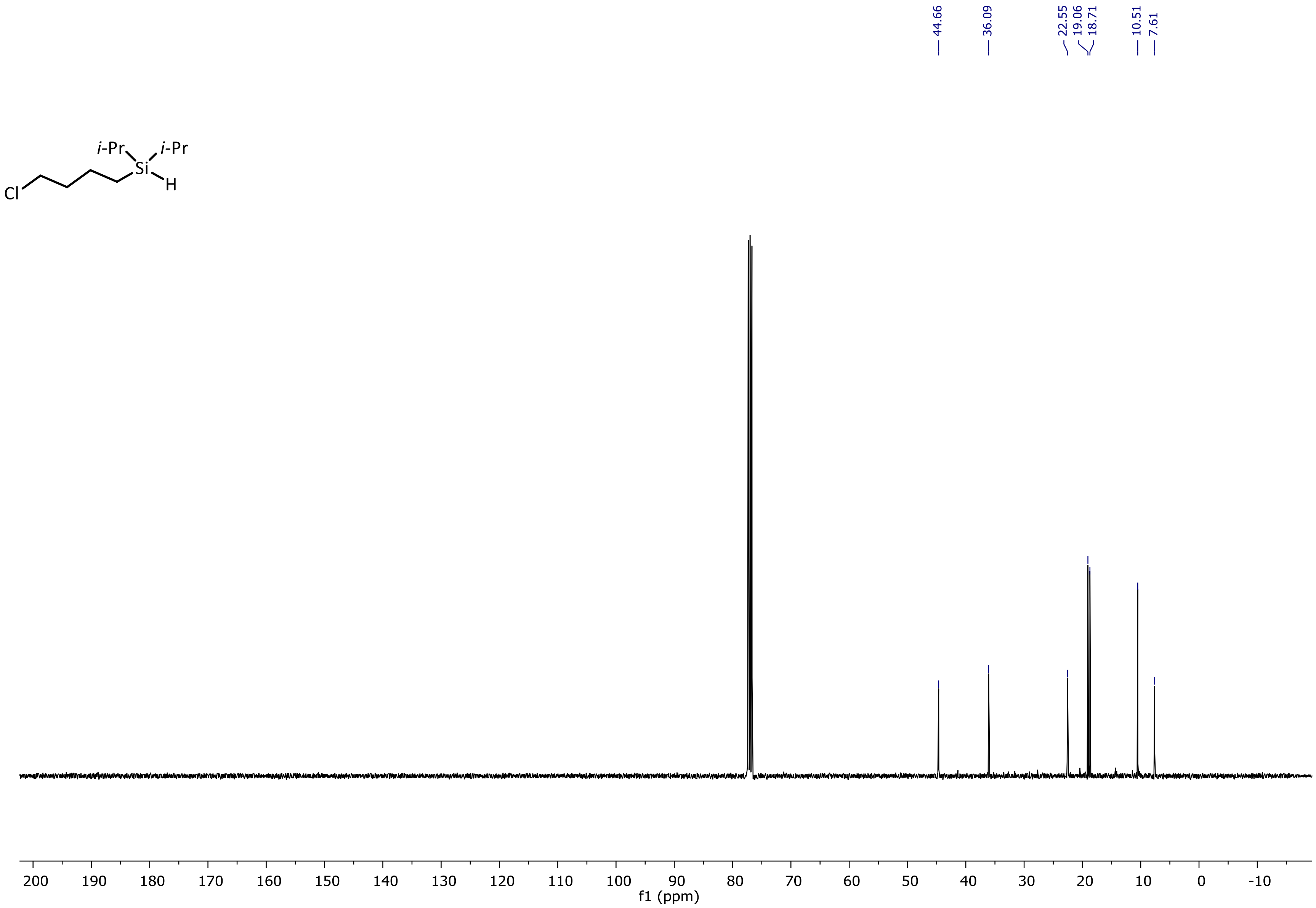

*^{1}H-NMR (400 MHz, $CDCl_3$) for compound **3f***

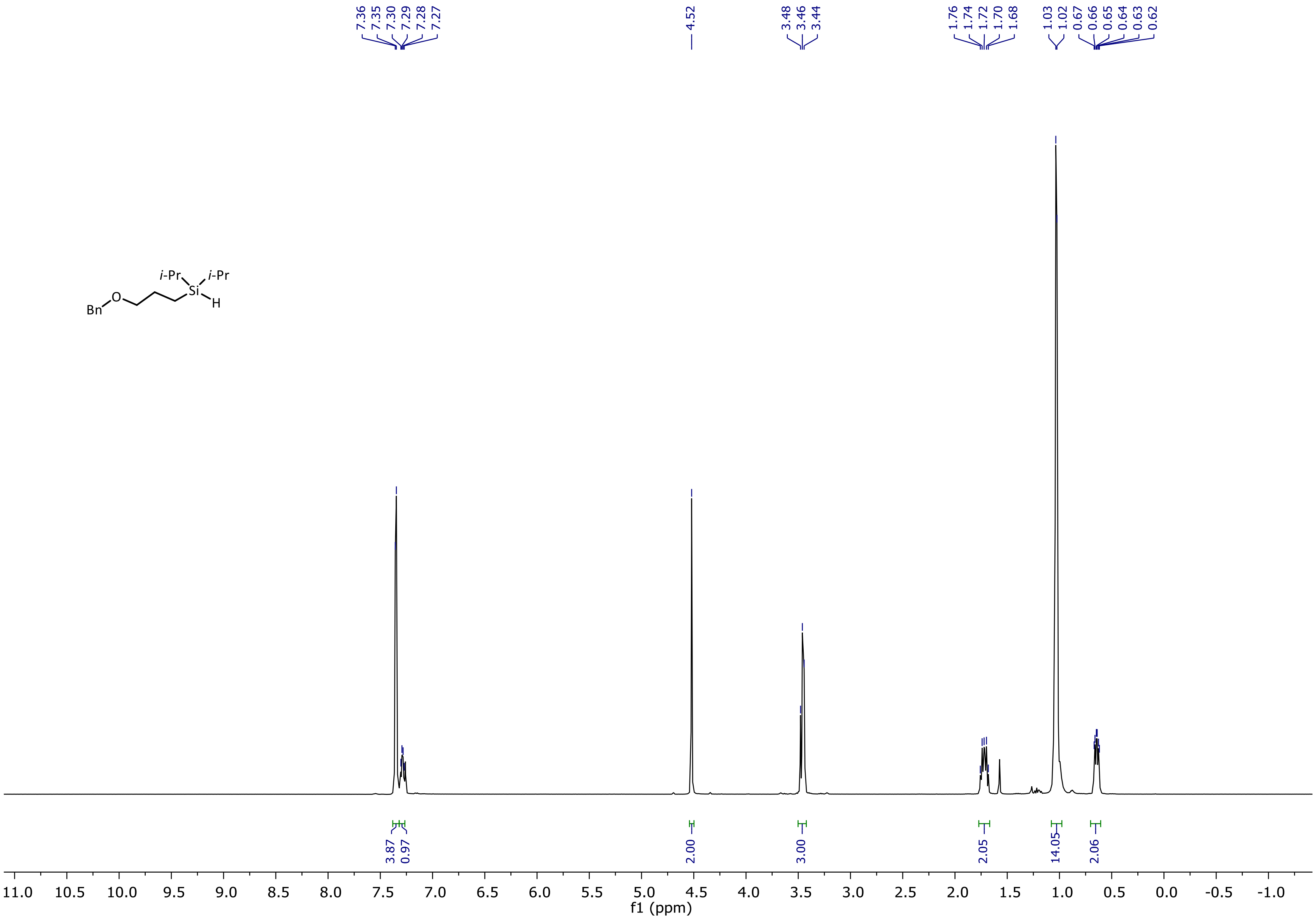

*^{13}C-NMR (101 MHz, $CDCl_3$) for compound **3f***

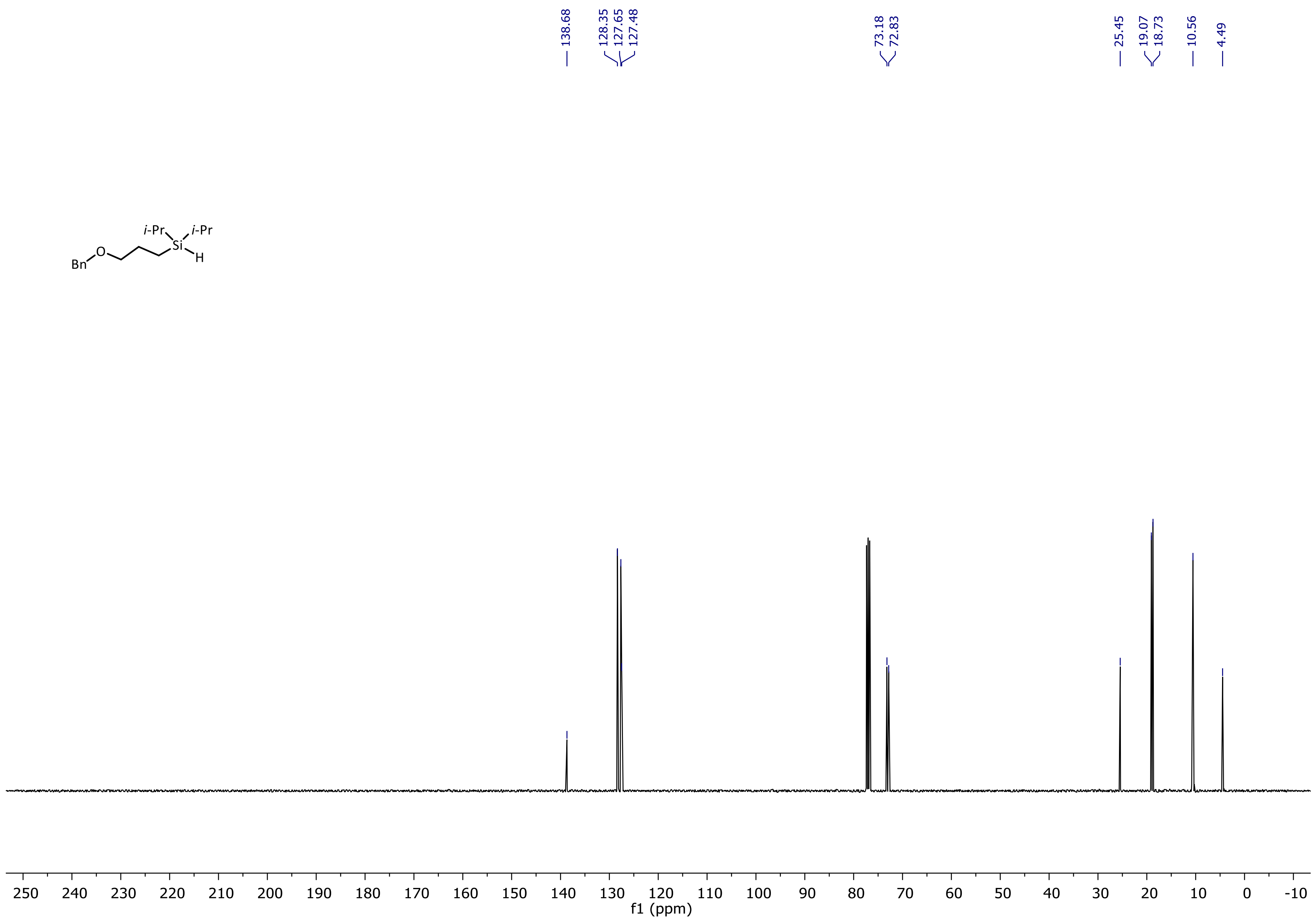

^{1}H-NMR (400 MHz, $CDCl_3$) for compound ***3j***

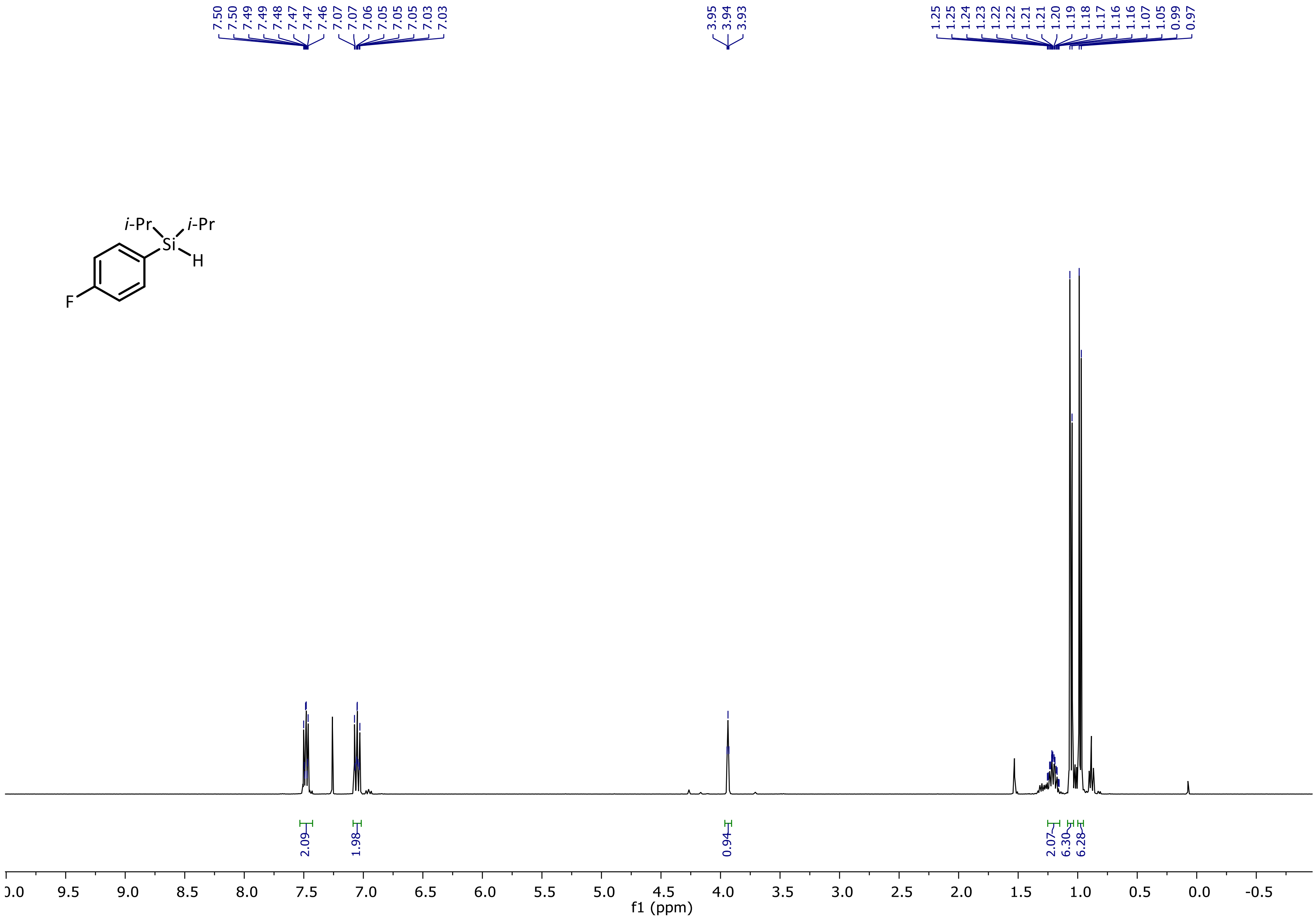

^{13}C-NMR (101 MHz, $CDCl_3$) for compound **3j**

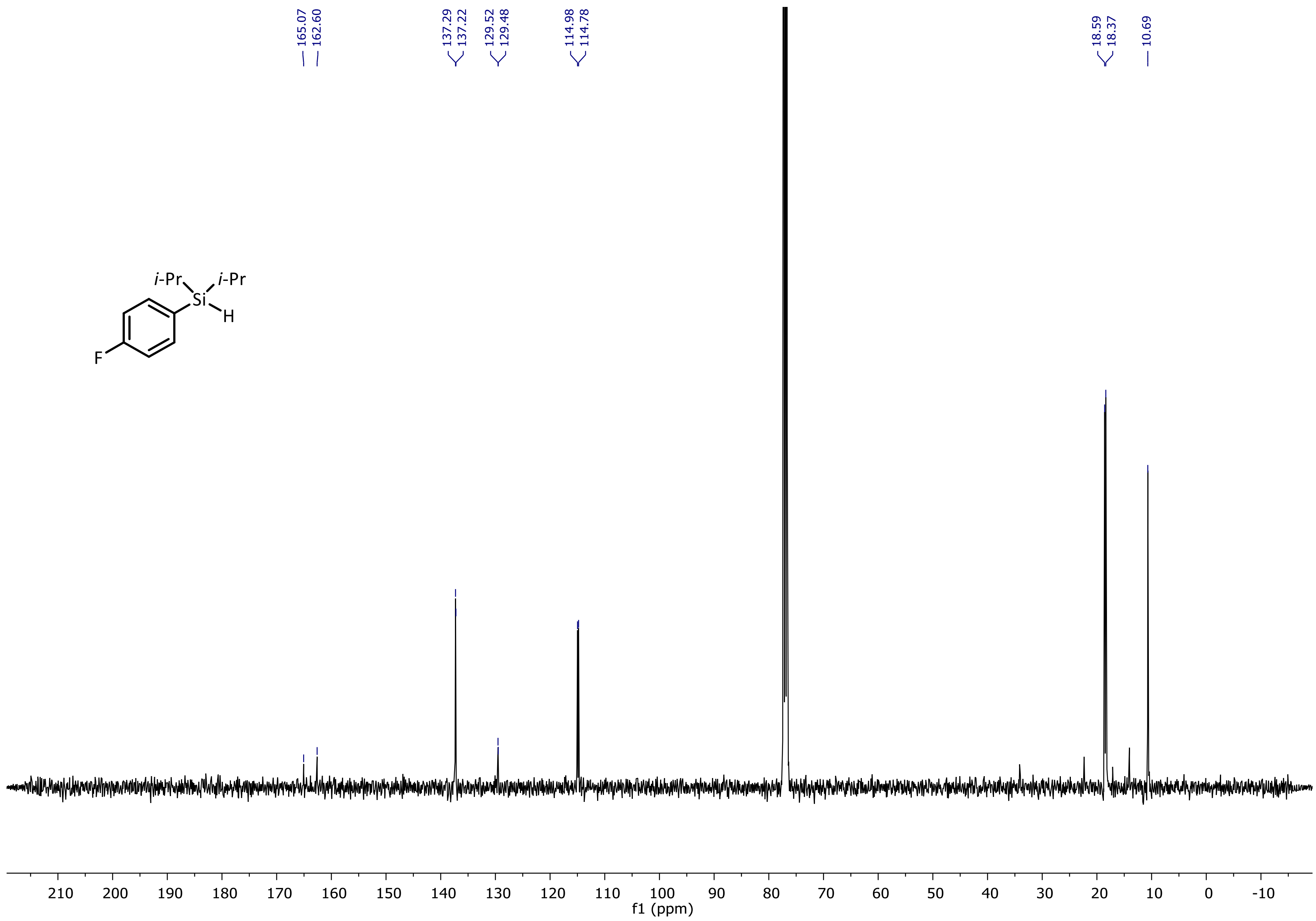

*^{19}F-NMR (377 MHz, CDCl$_3$) for compound **3j***

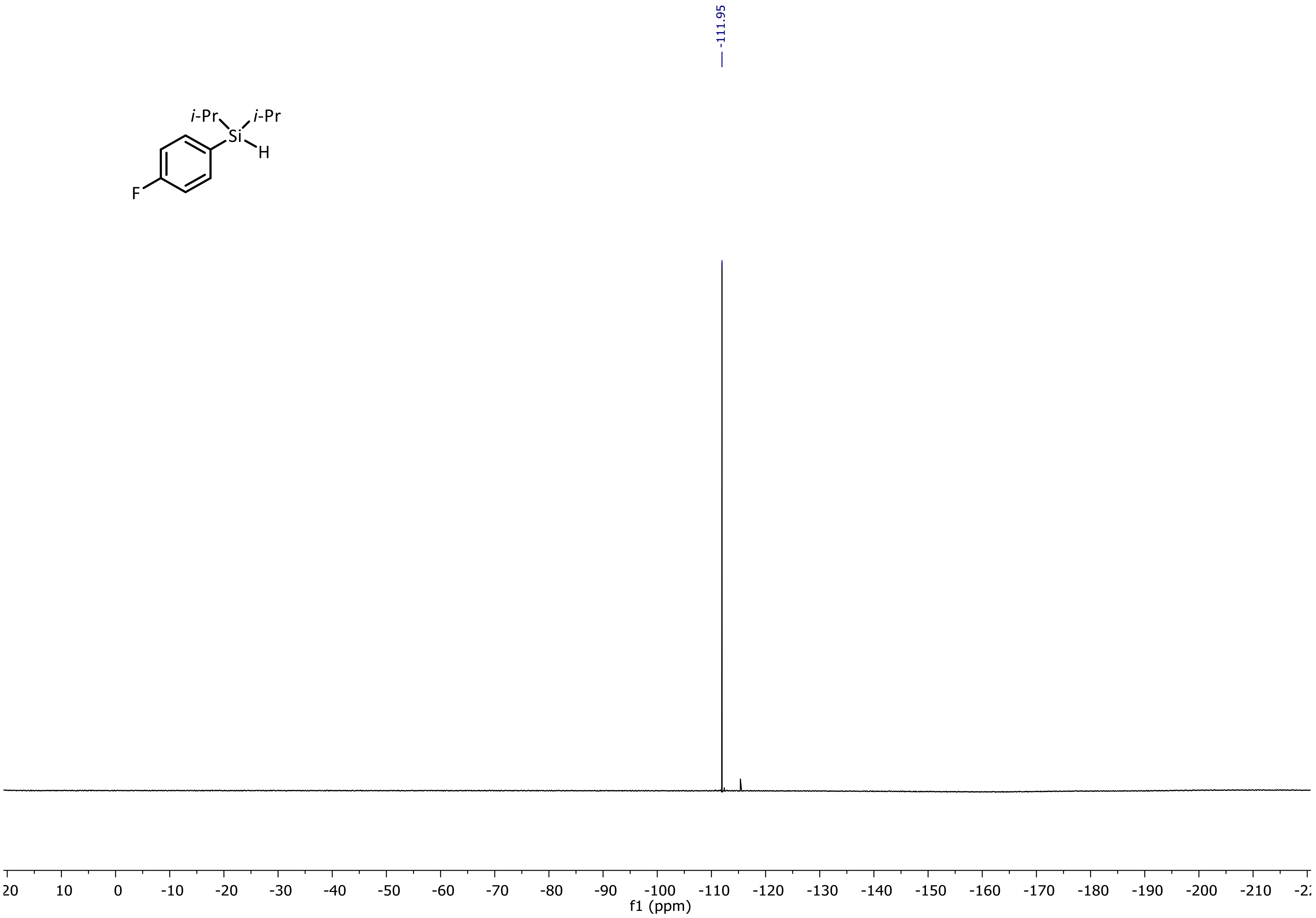

^{1}H-NMR (400 MHz, $CDCl_3$) for compound **3l**

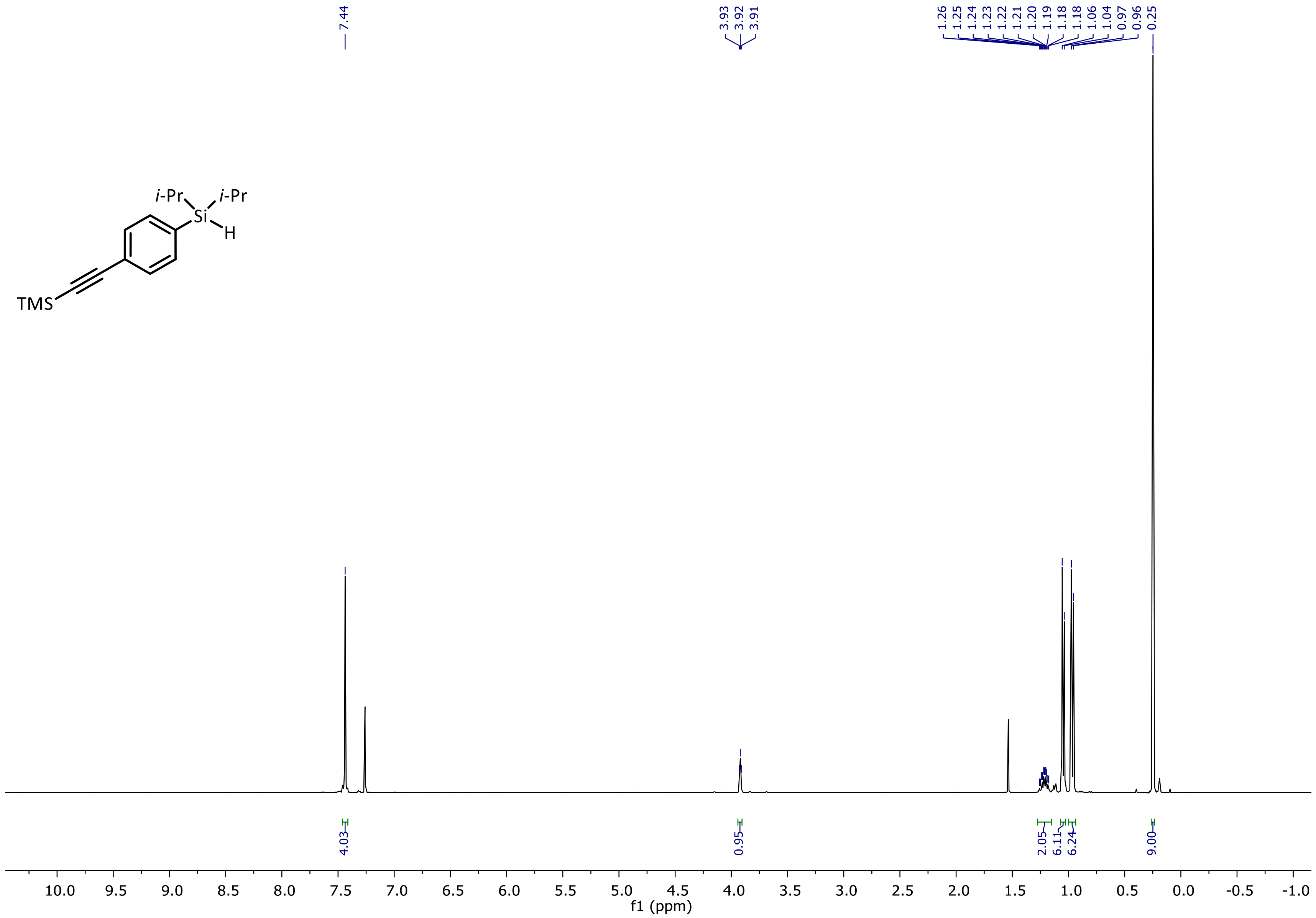

^{13}C-NMR (101 MHz, $CDCl_3$) for compound **3l**

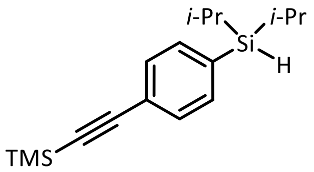

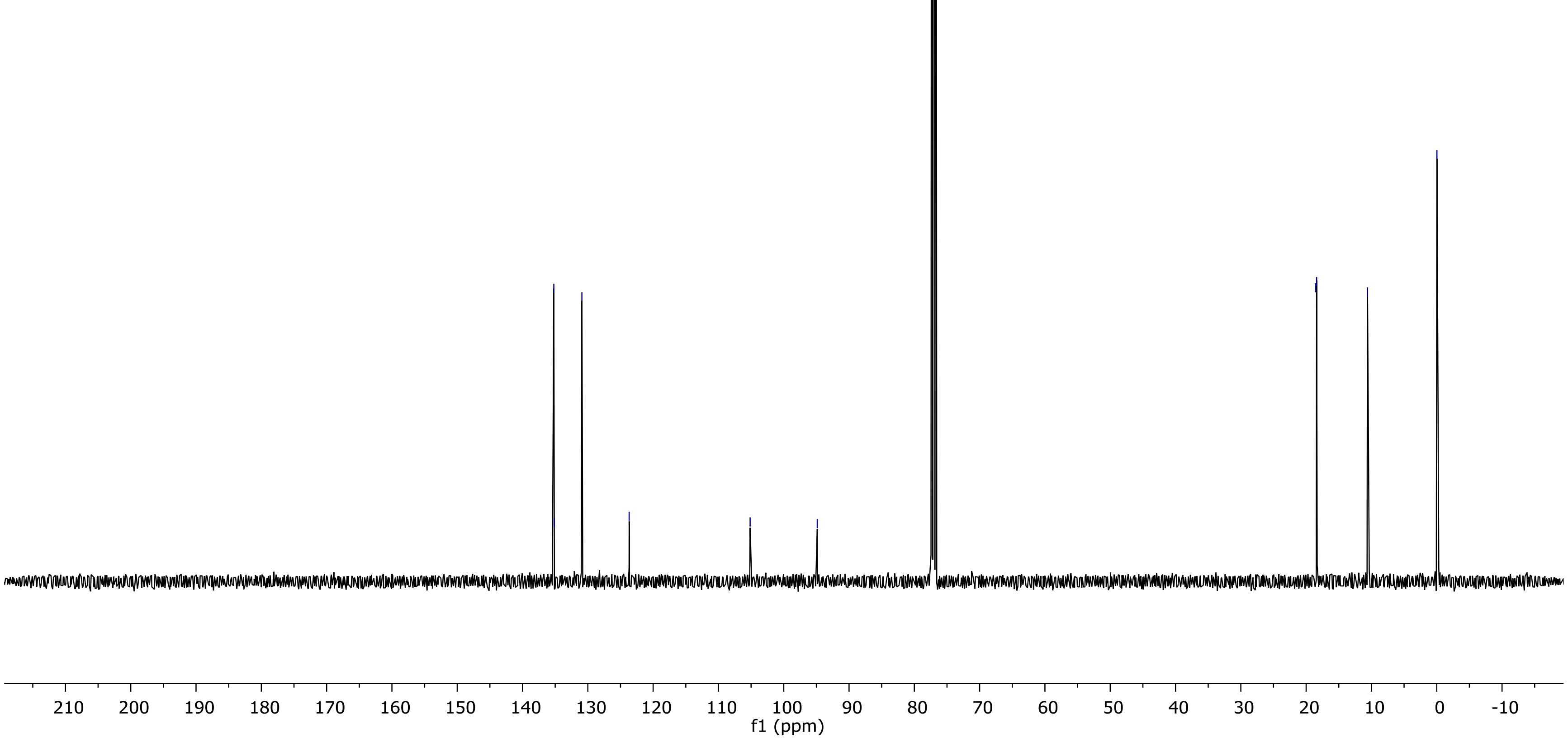

^{1}H-NMR (400 MHz, $CDCl_3$) for compound ***3m***

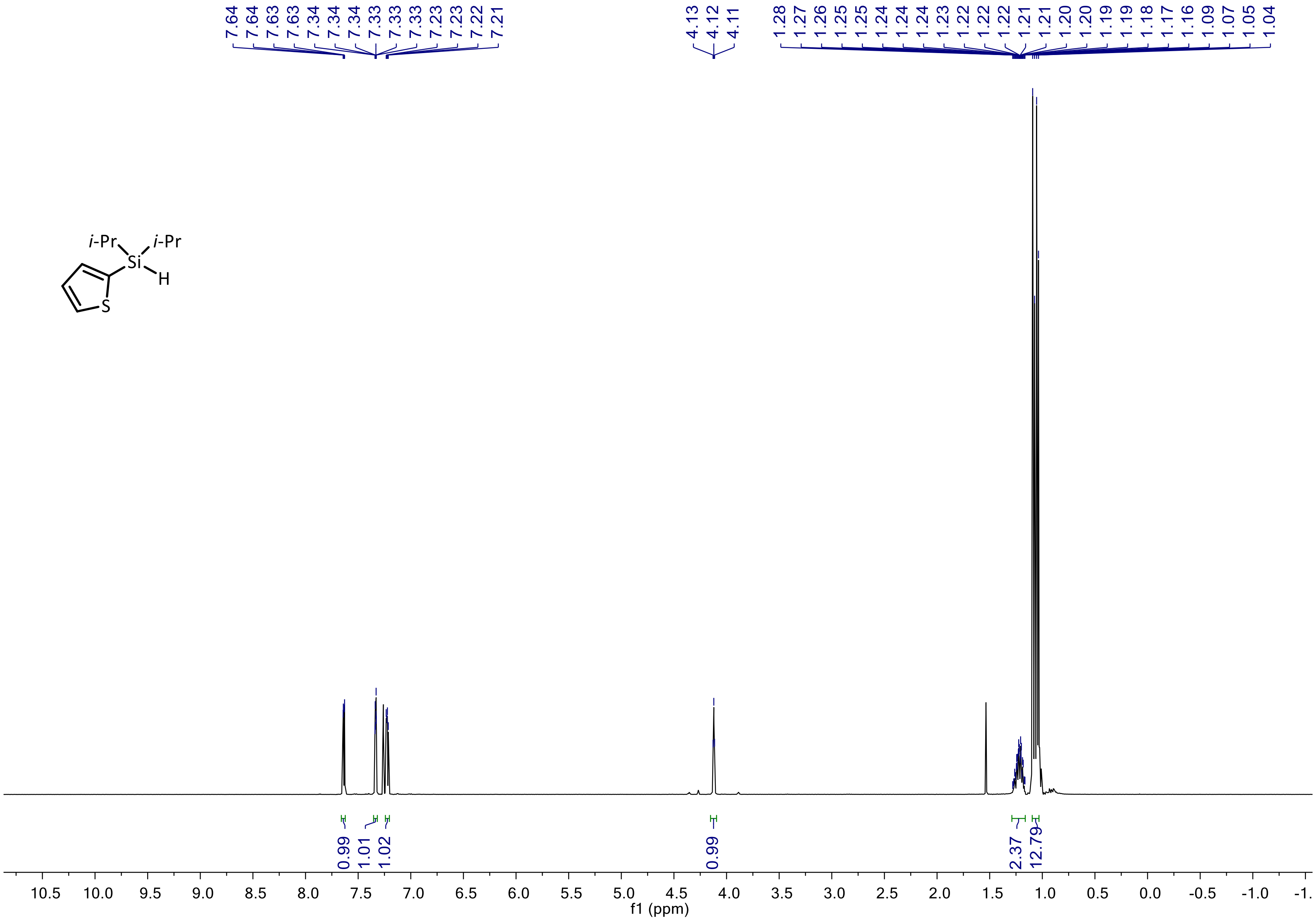

^{13}C-NMR (101 MHz, $CDCl_3$) for compound ***3m***

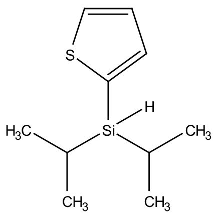

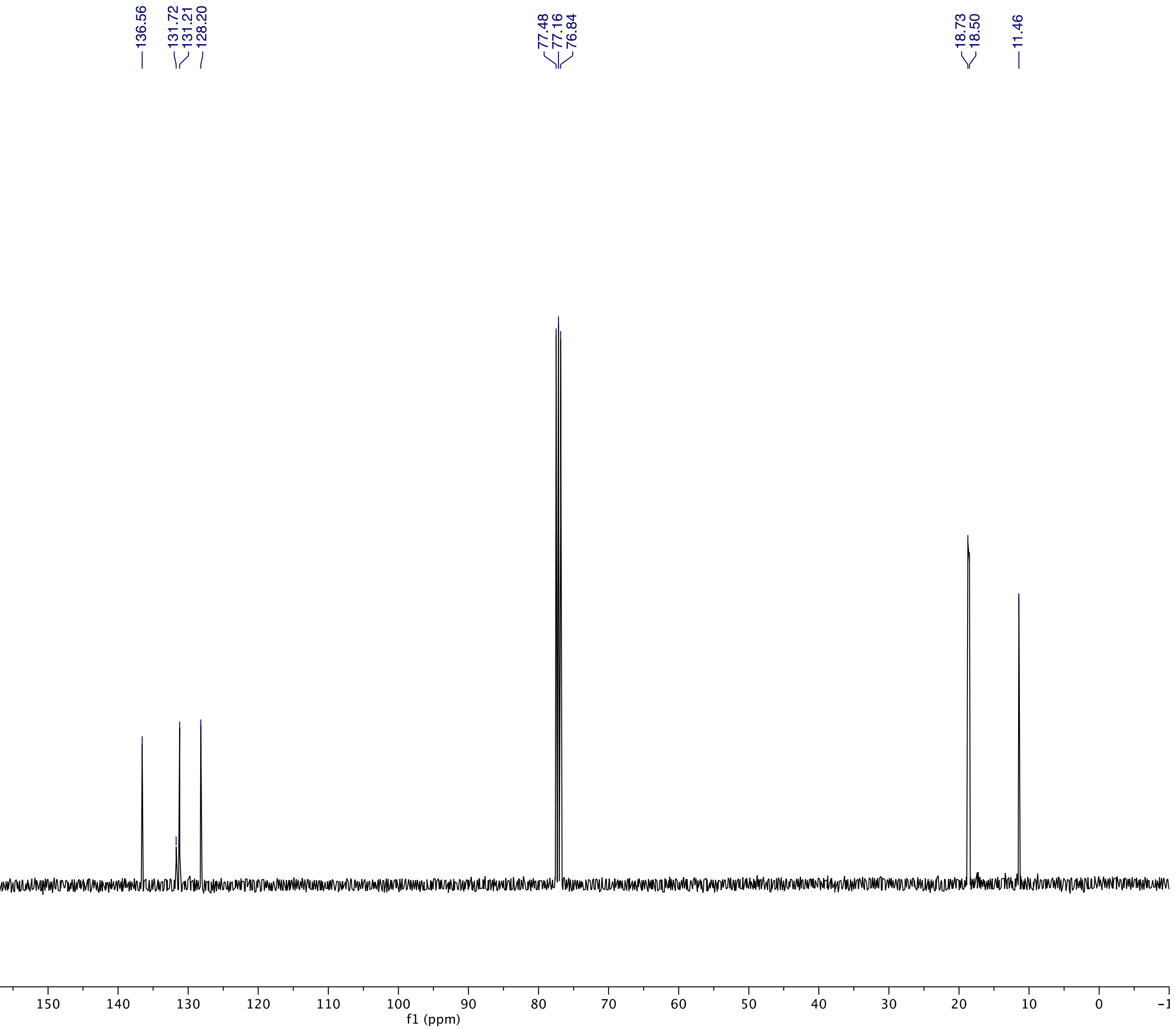

^{1}H-NMR (400 MHz, $CDCl_3$) for compound ***3q***

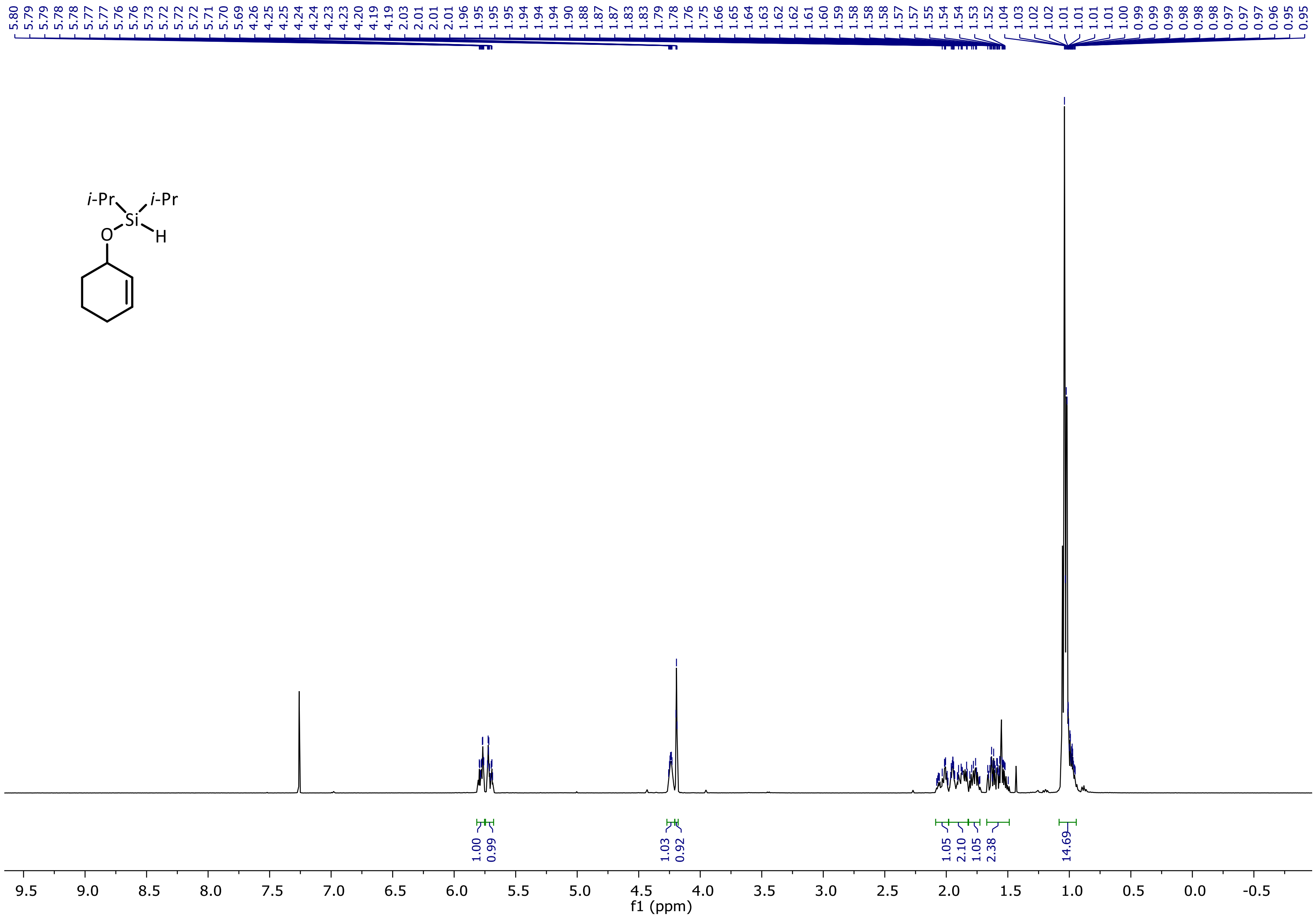

*^{13}C-NMR (101 MHz, CDCl₃) for compound **3q***

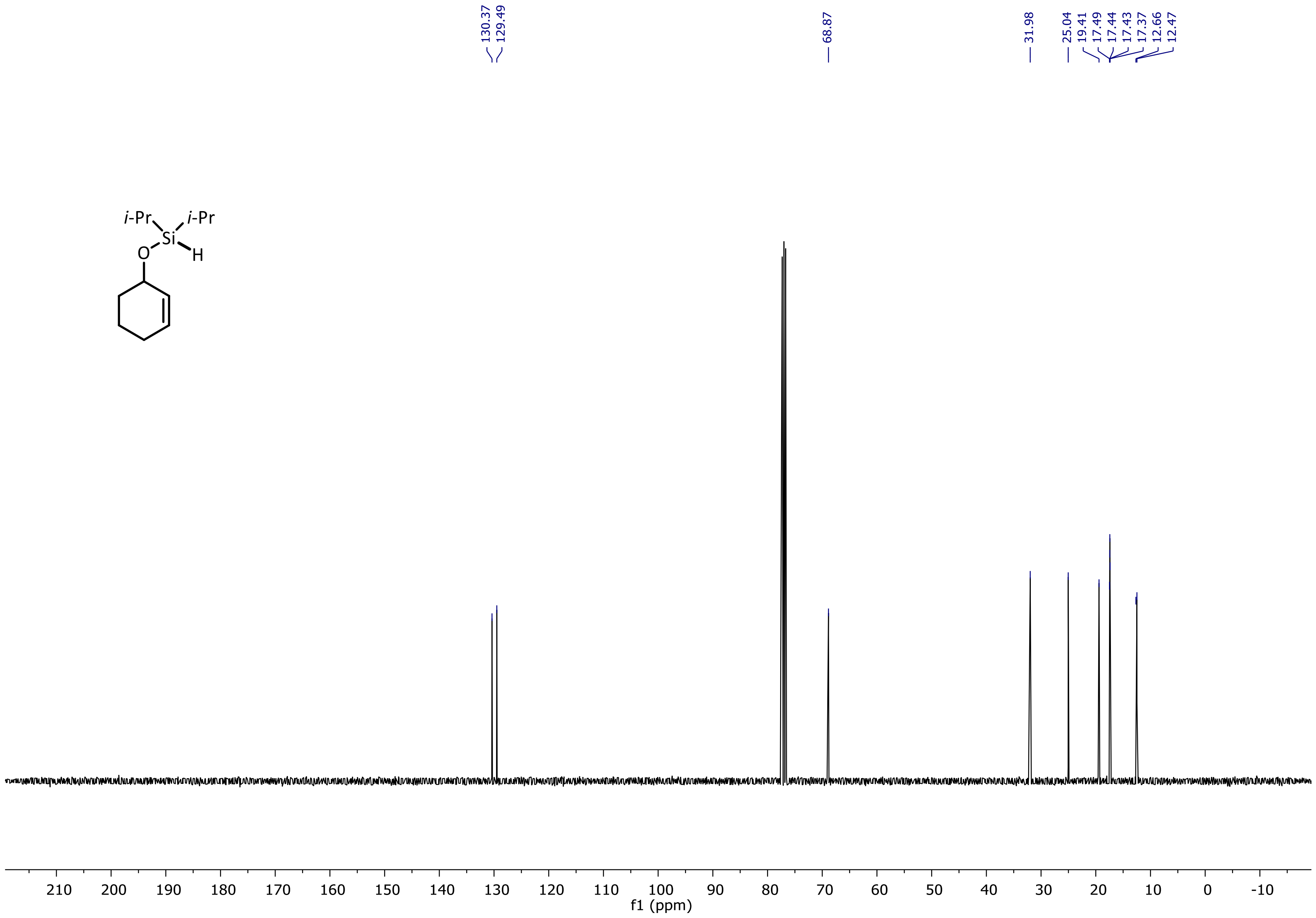

^{1}H-NMR (400 MHz, $CDCl_3$) for compound **3r**

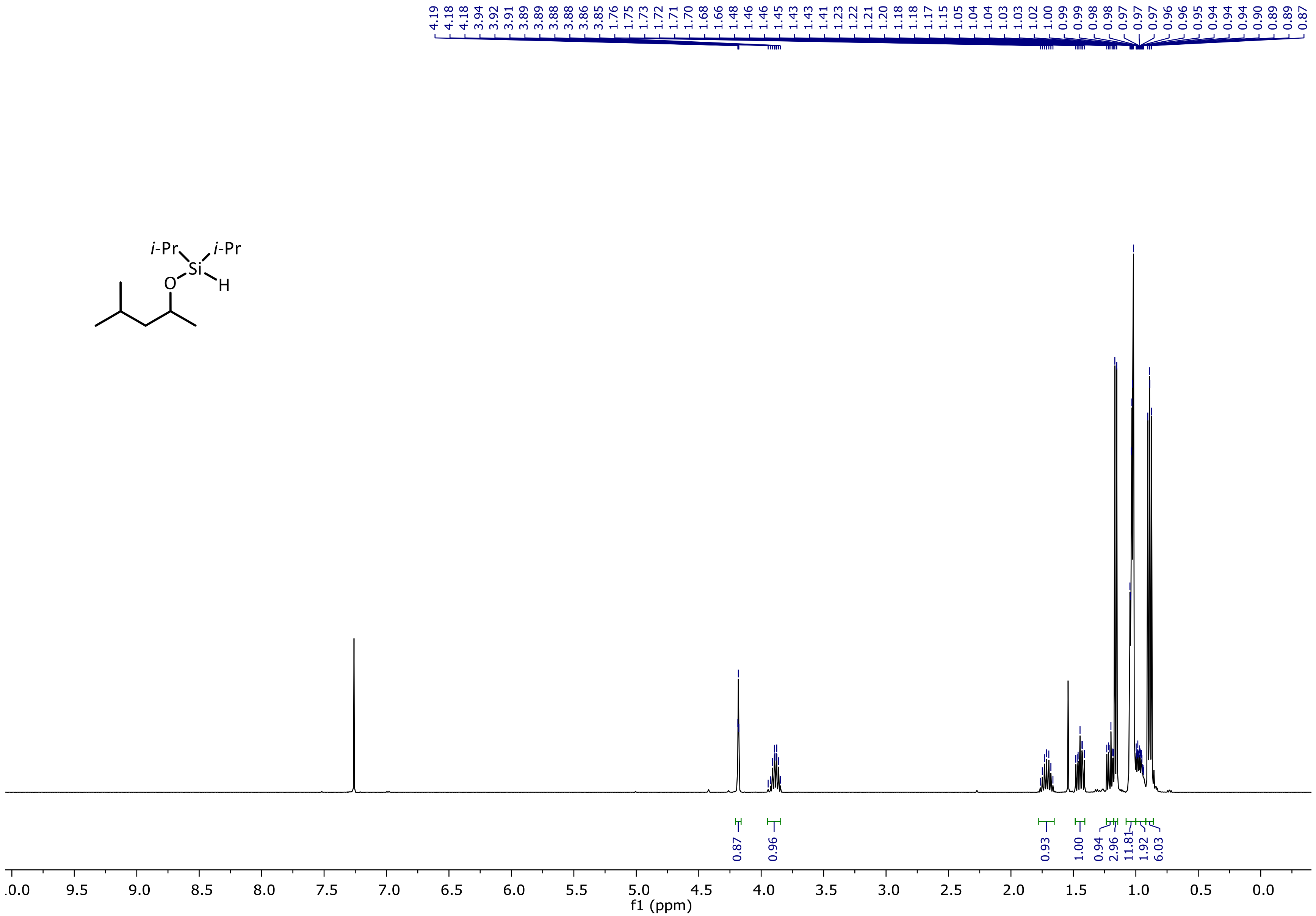

^{13}C-NMR (101 MHz, CDCl$_3$) for compound **3r**

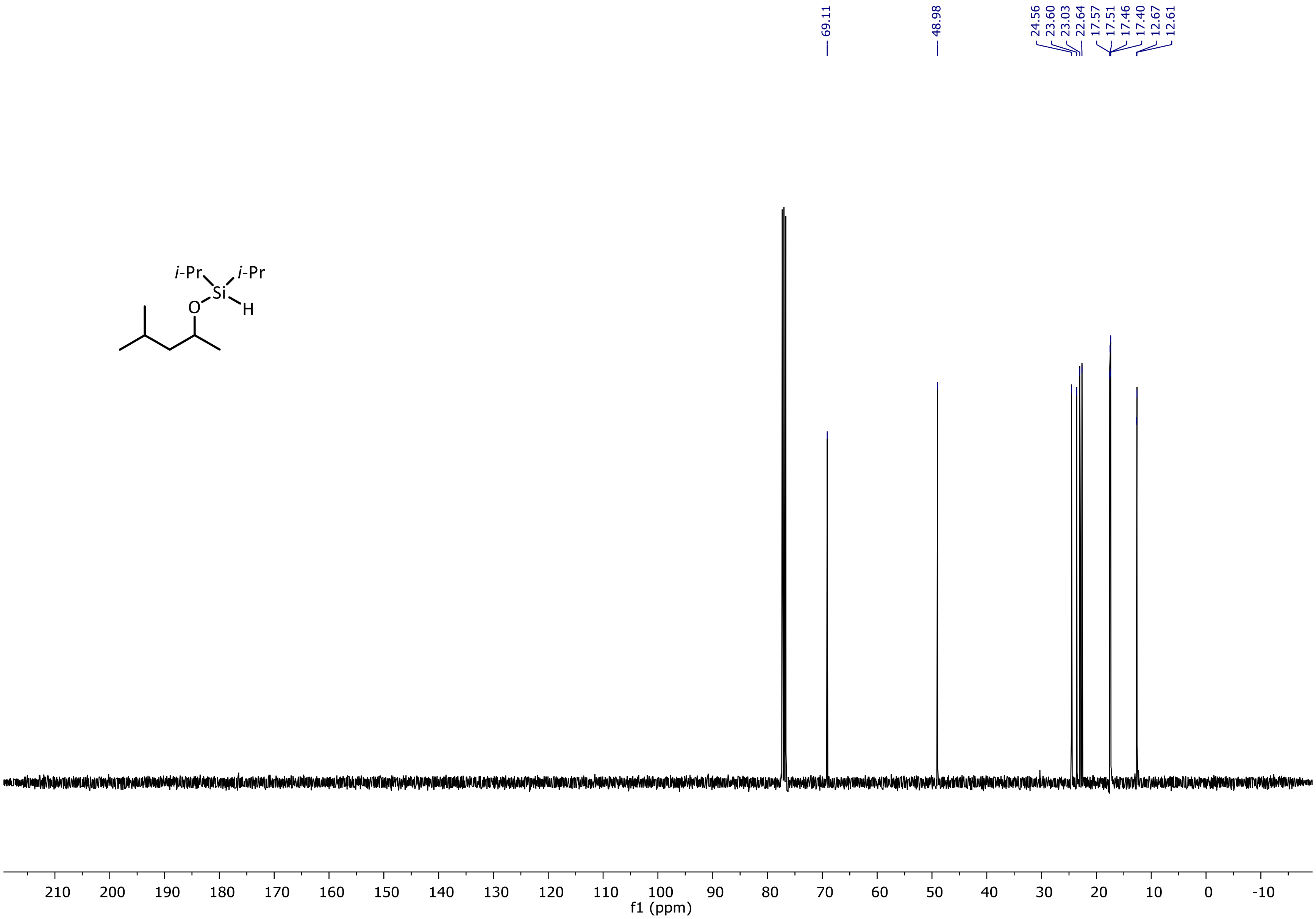

^{1}H-NMR (400 MHz, CDCl$_3$) for compound ***3s***

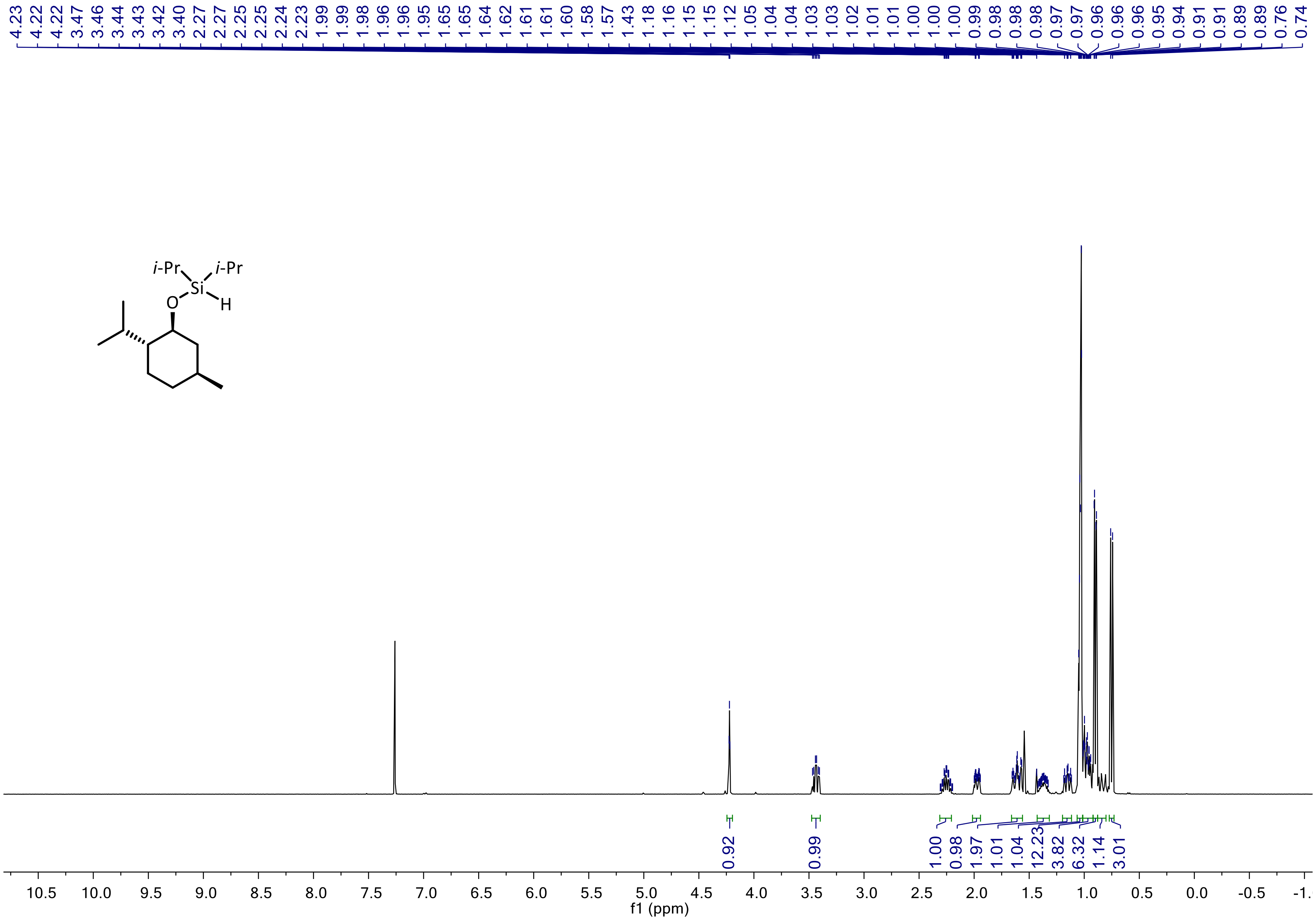

^{13}C-NMR (101 MHz, CDCl$_3$) for compound ***3s***

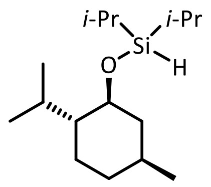

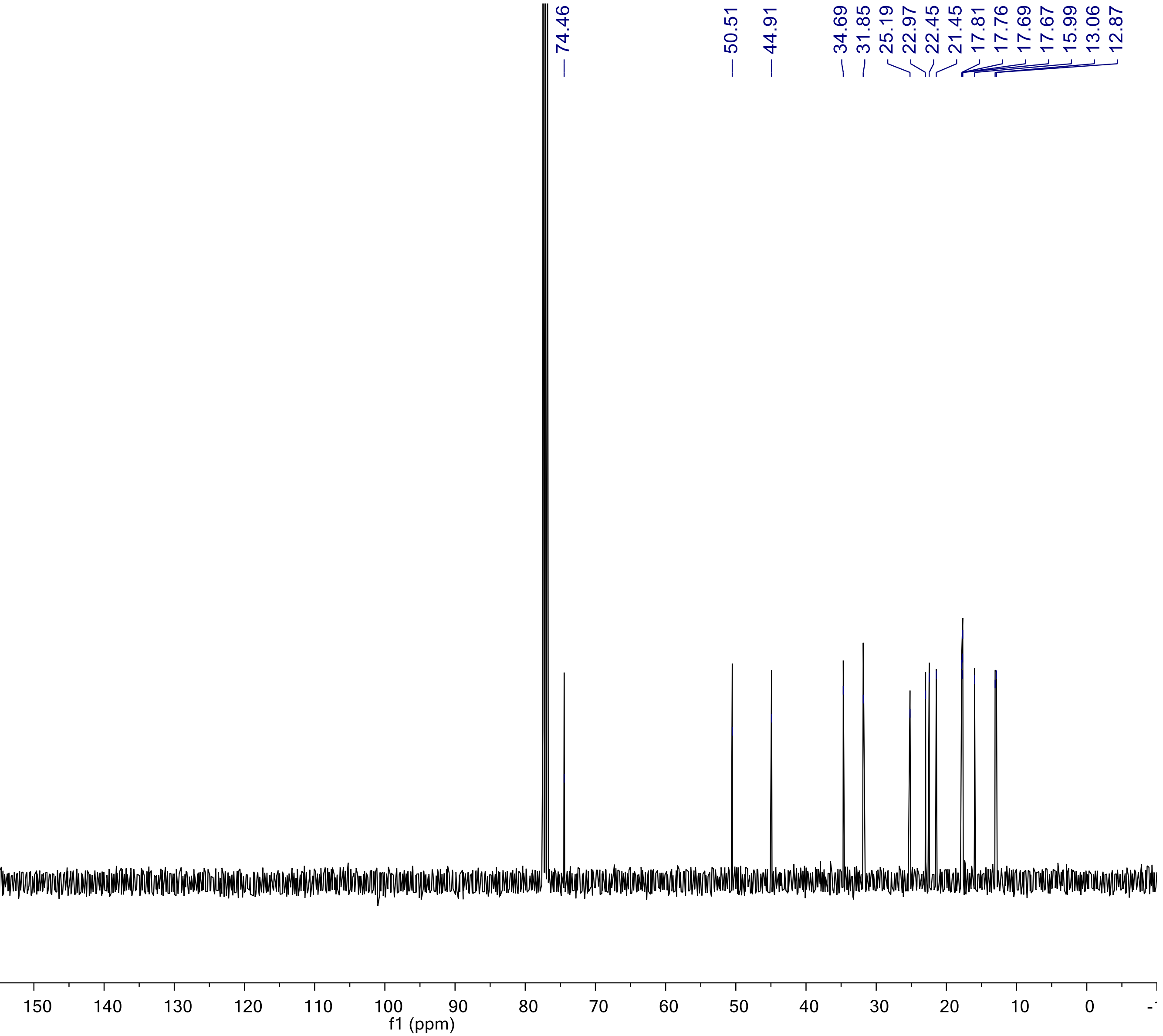

^{1}H-NMR (400 MHz, $CDCl_3$) for compound ***3t***

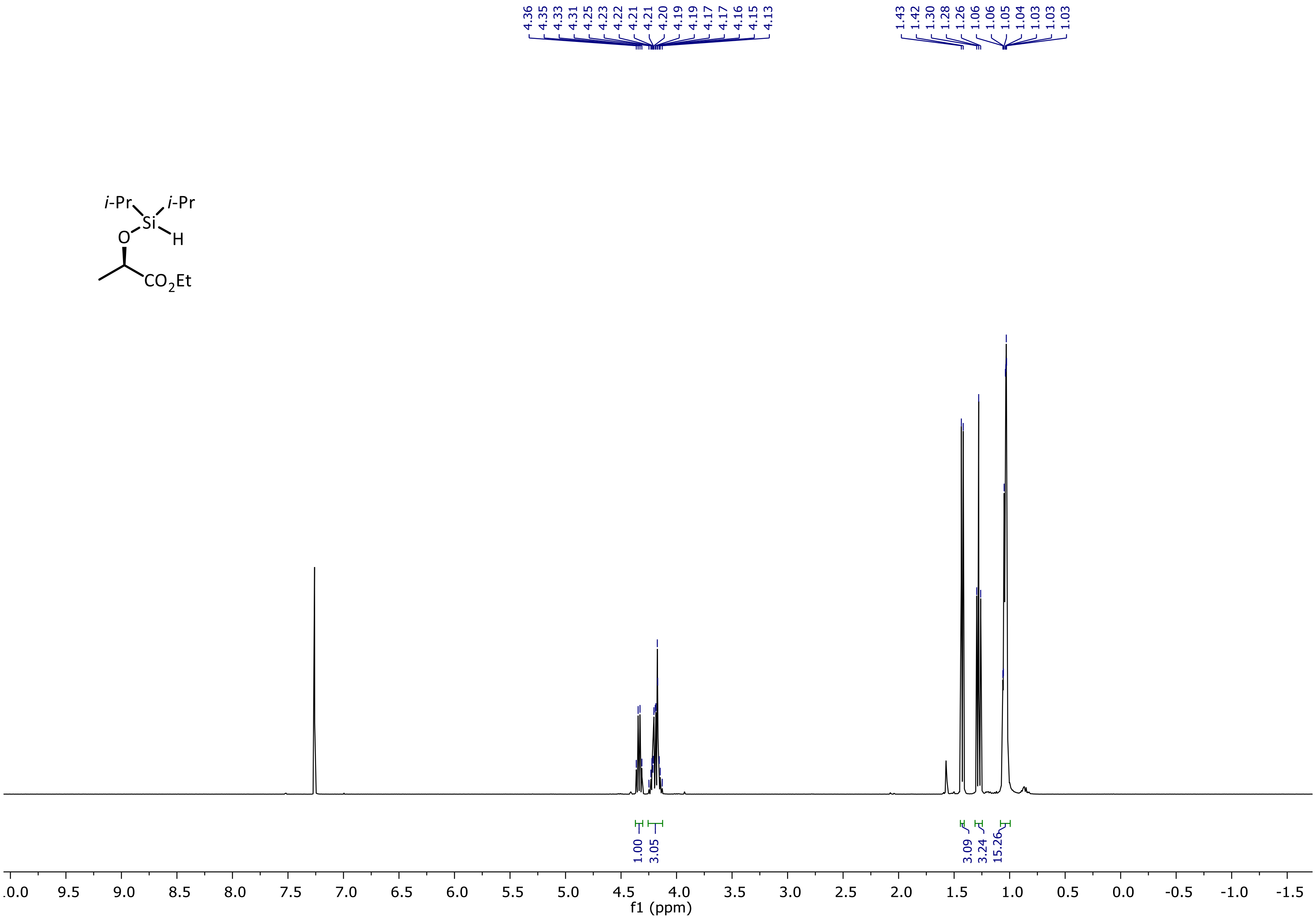

^{13}C-NMR (101 MHz, $CDCl_3$) for compound ***3t***

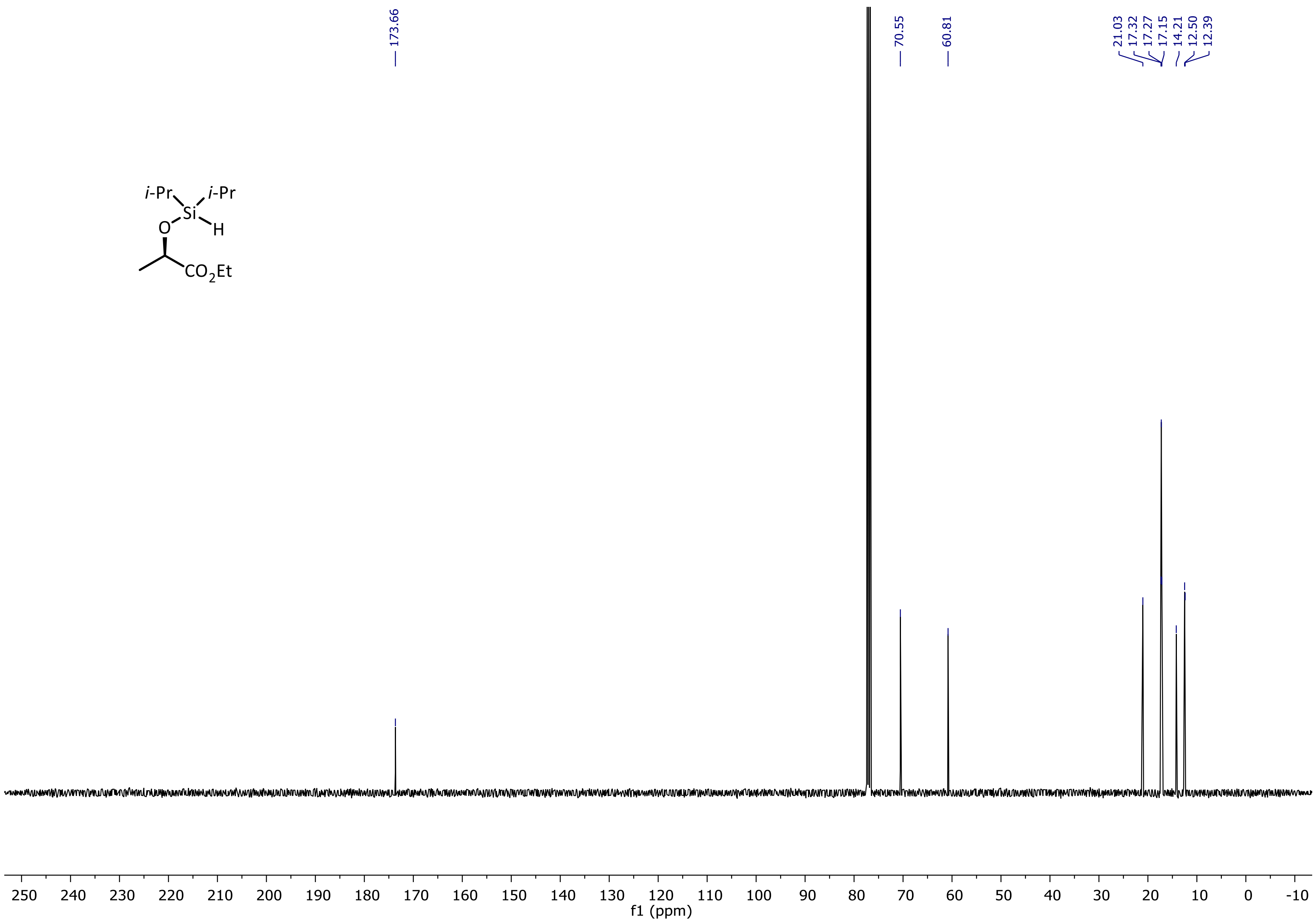

^{1}H-NMR (400 MHz, $CDCl_3$) for compound ***3u***

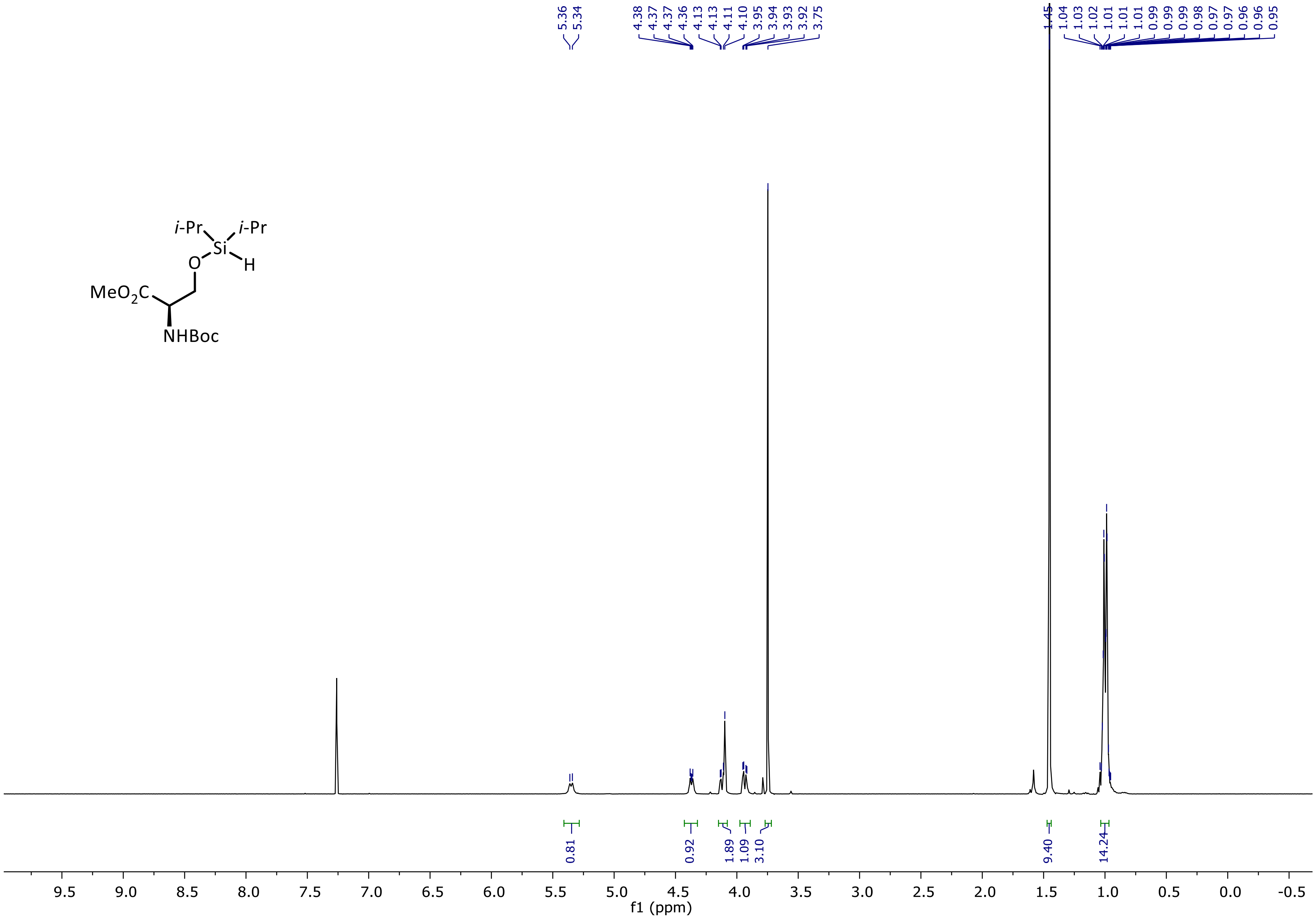

*^{13}C-NMR (101 MHz, CDCl$_3$) for compound **3u***

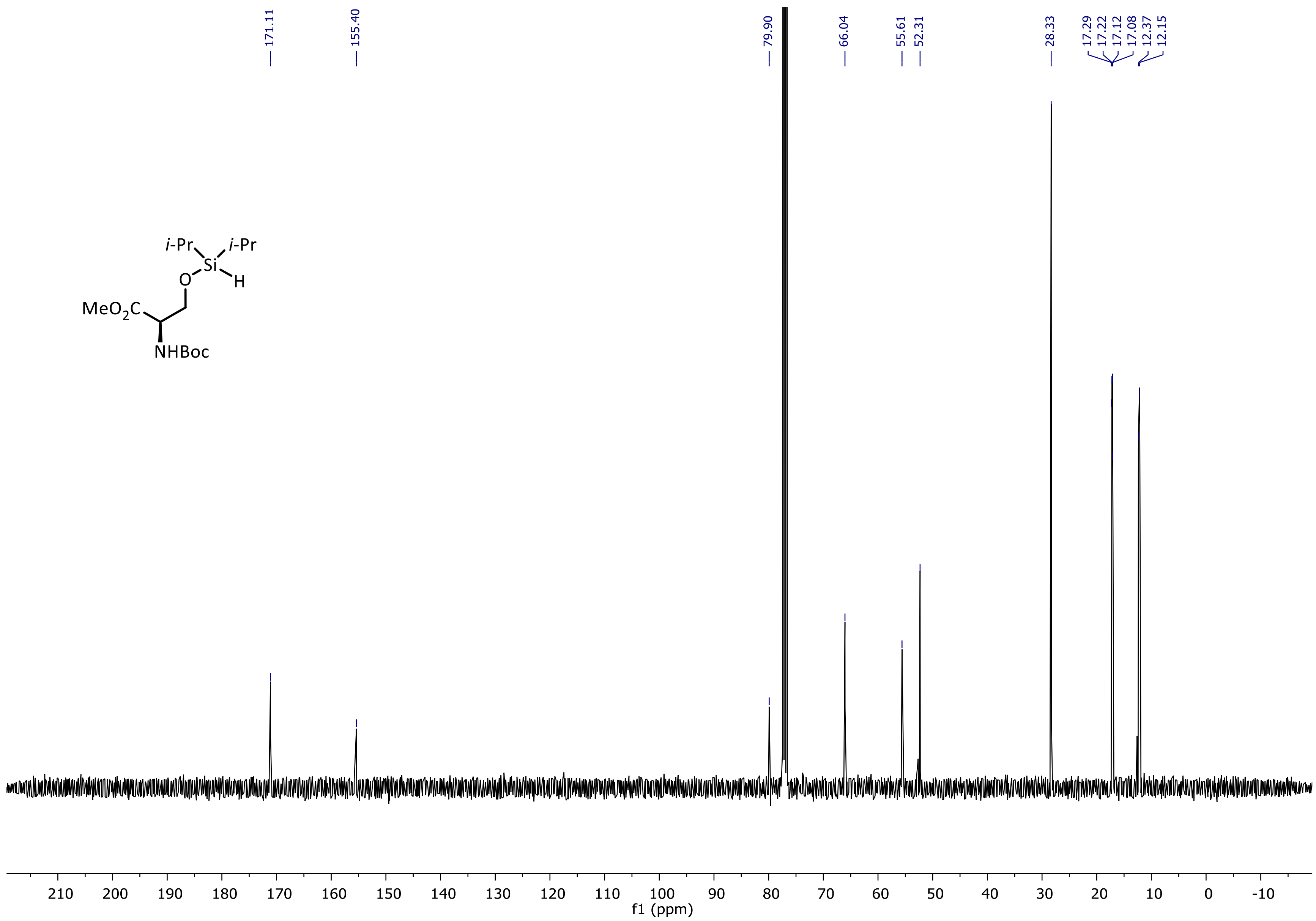

^{1}H-NMR (400 MHz, $CDCl_3$) for compound **3v**

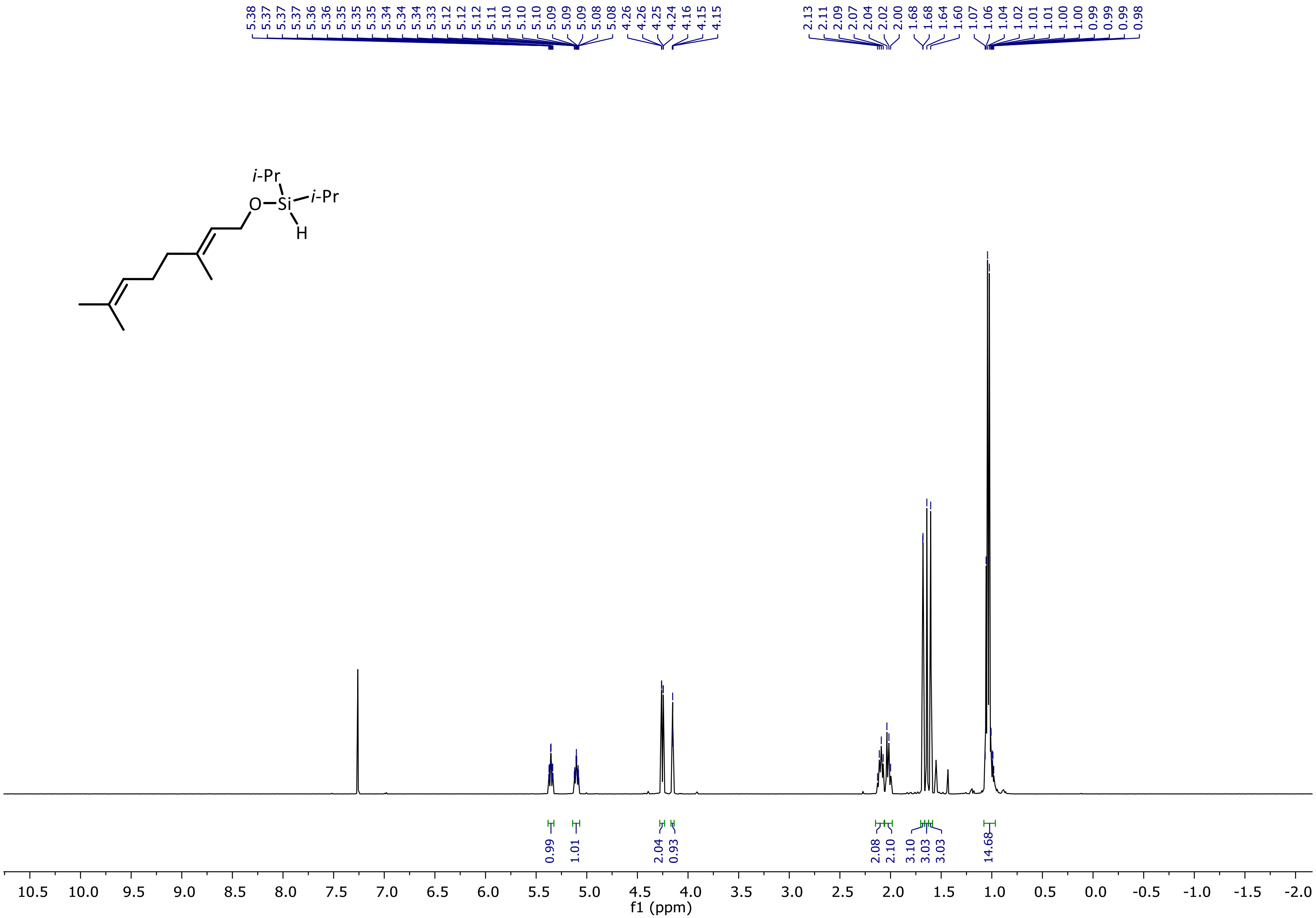

^{13}C-NMR (101 MHz, $CDCl_3$) for compound **3v**

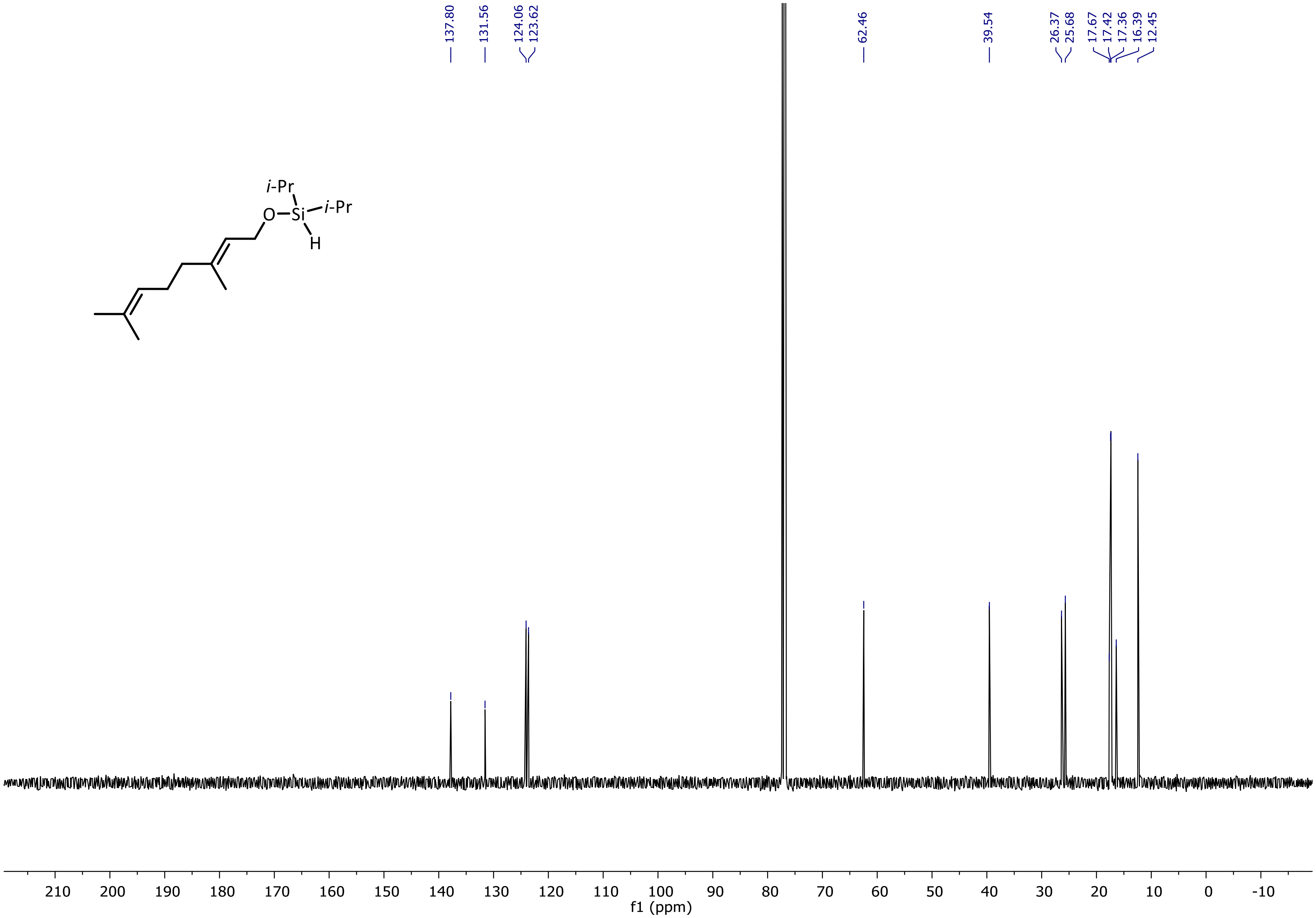

*^{1}H-NMR (400 MHz, $CDCl_3$) for compound **3x'***

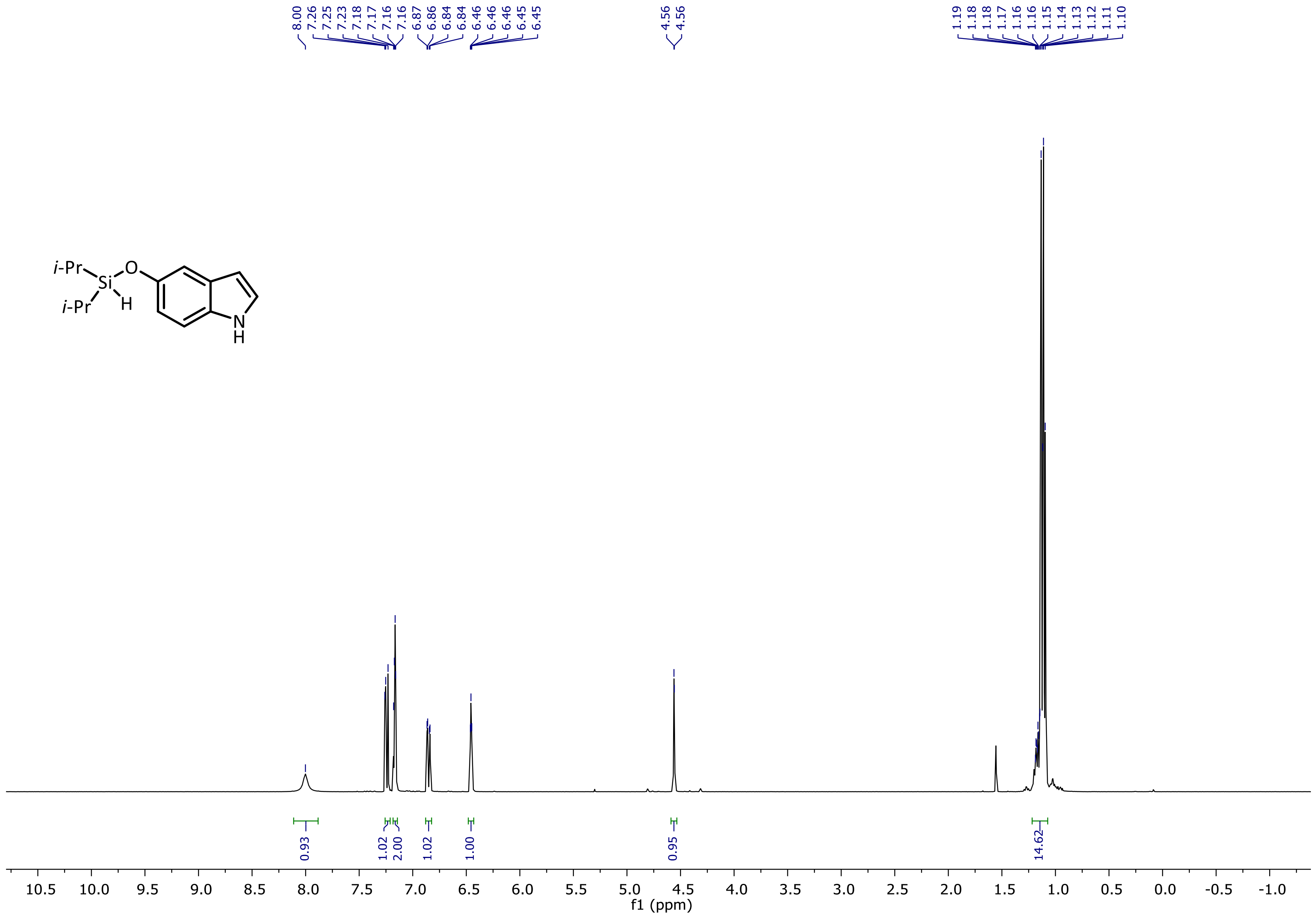

^{13}*C-NMR (101 MHz, CDCl$_3$) for compound* ***3x'***

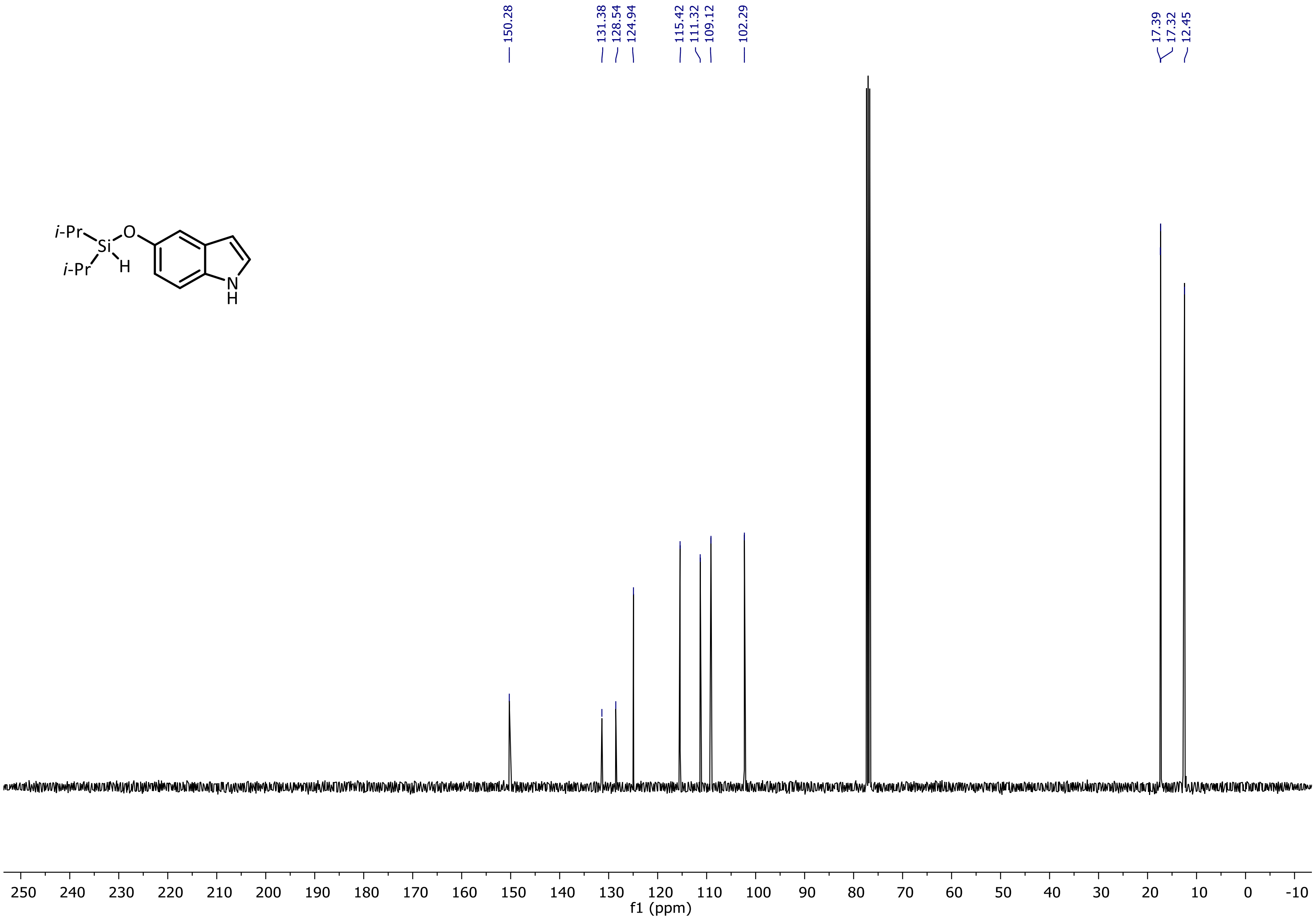

*^{1}H-NMR (400 MHz, $CDCl_3$) for compound **3x***

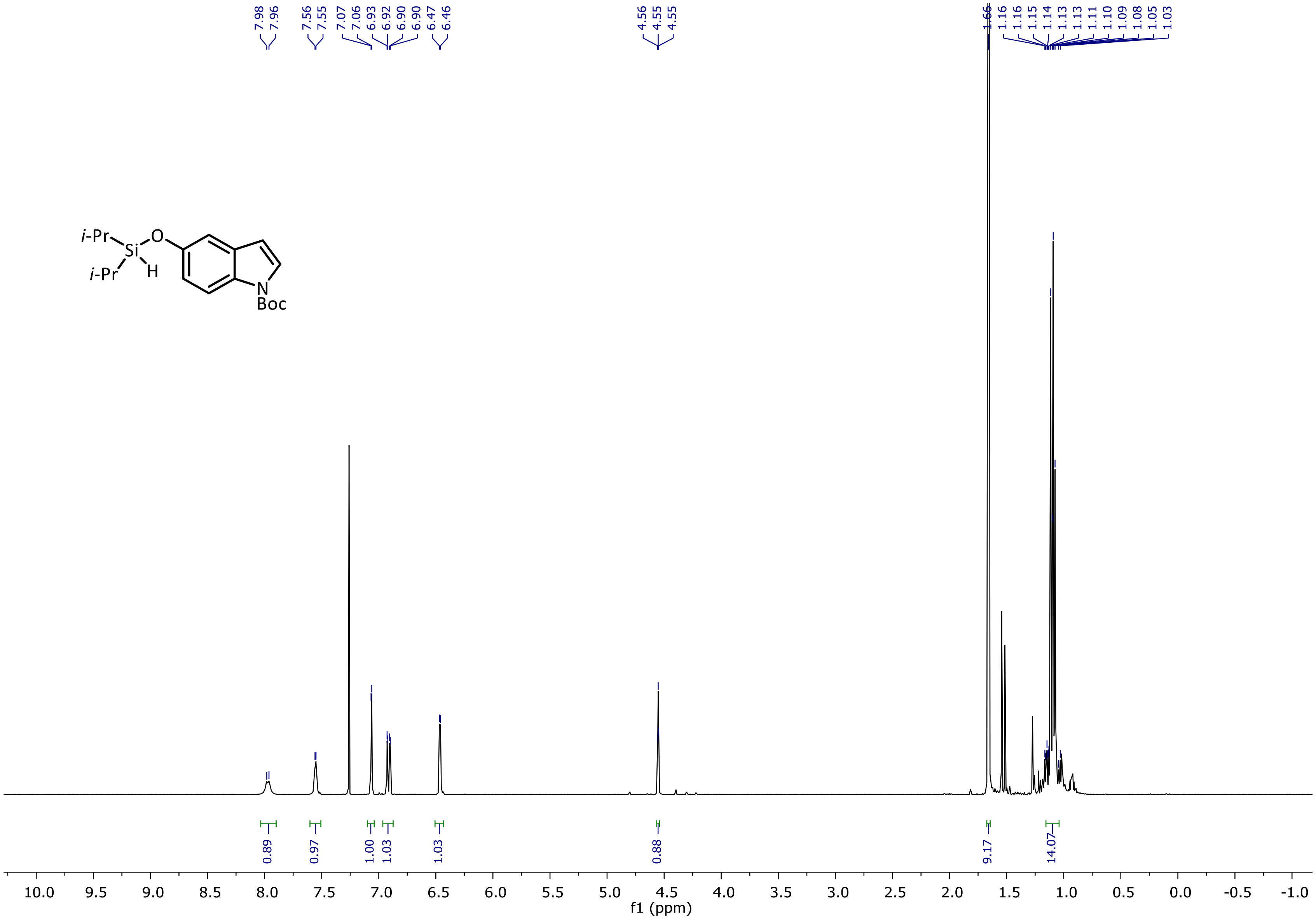

*^{13}C-NMR (101 MHz, CDCl₃) for compound **3x***

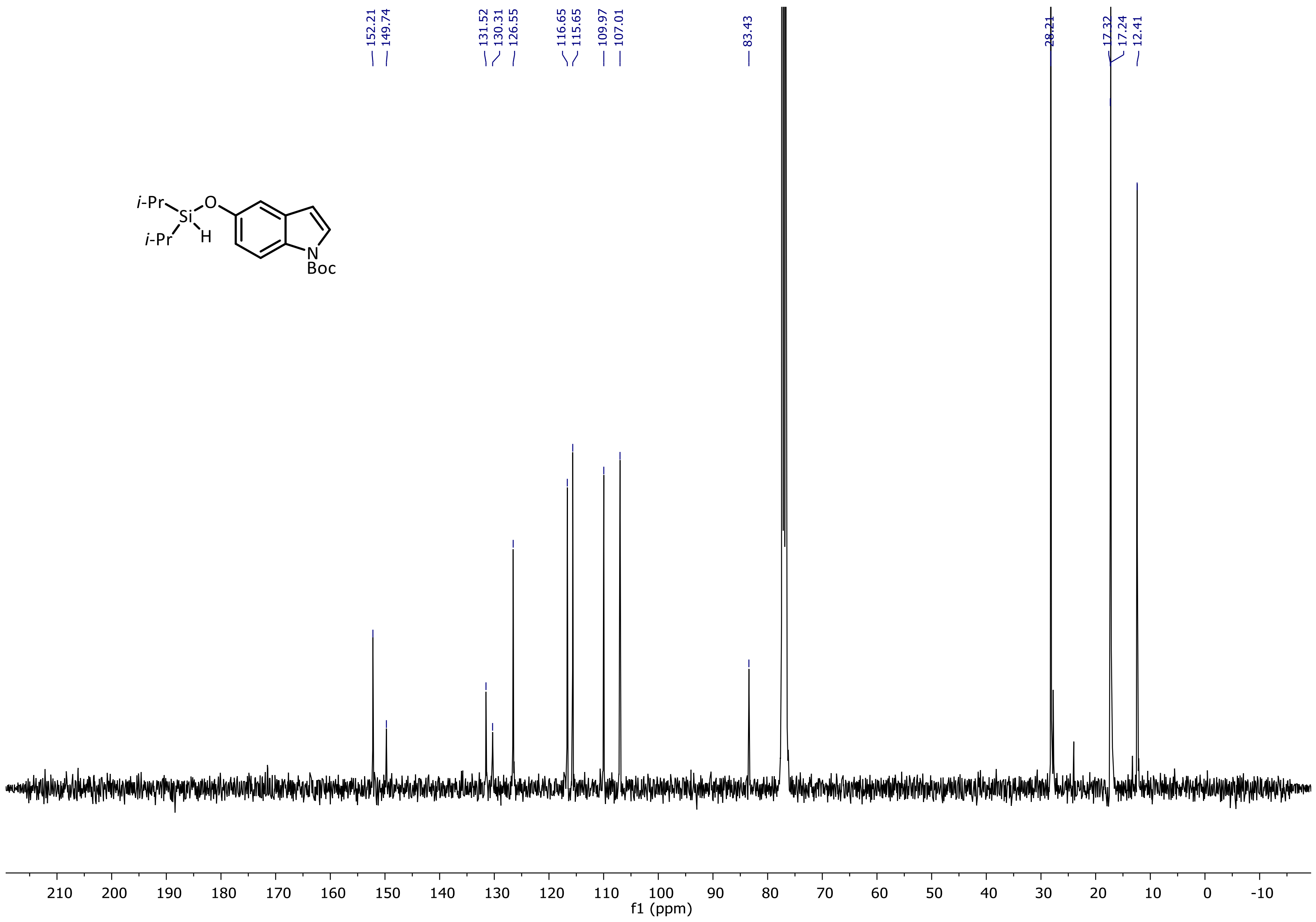

*^{1}H-NMR (400 MHz, $CDCl_3$) for compound **9a***

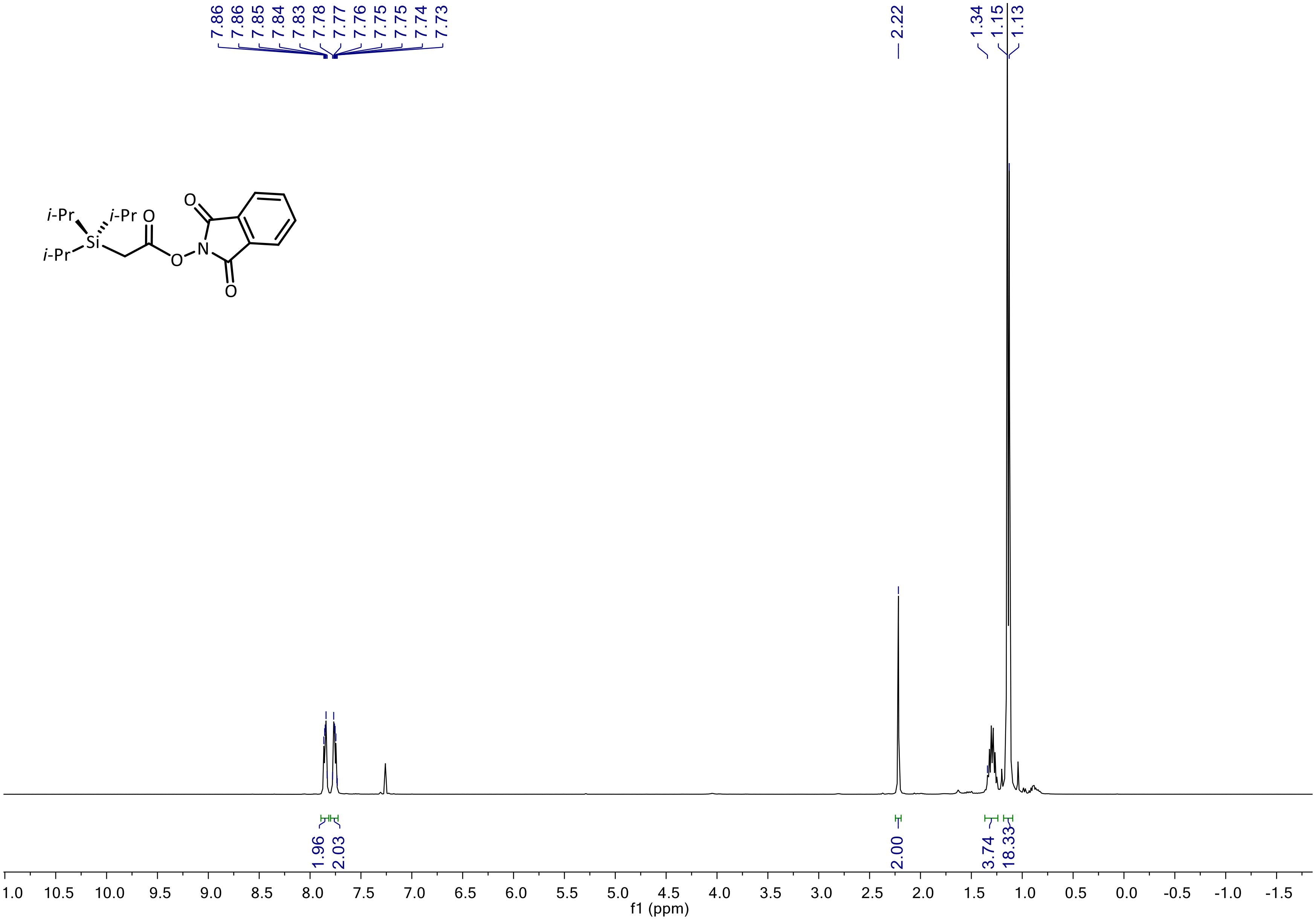

^{13}*C-NMR (101 MHz, CDCl$_3$) for compound* ***9a***

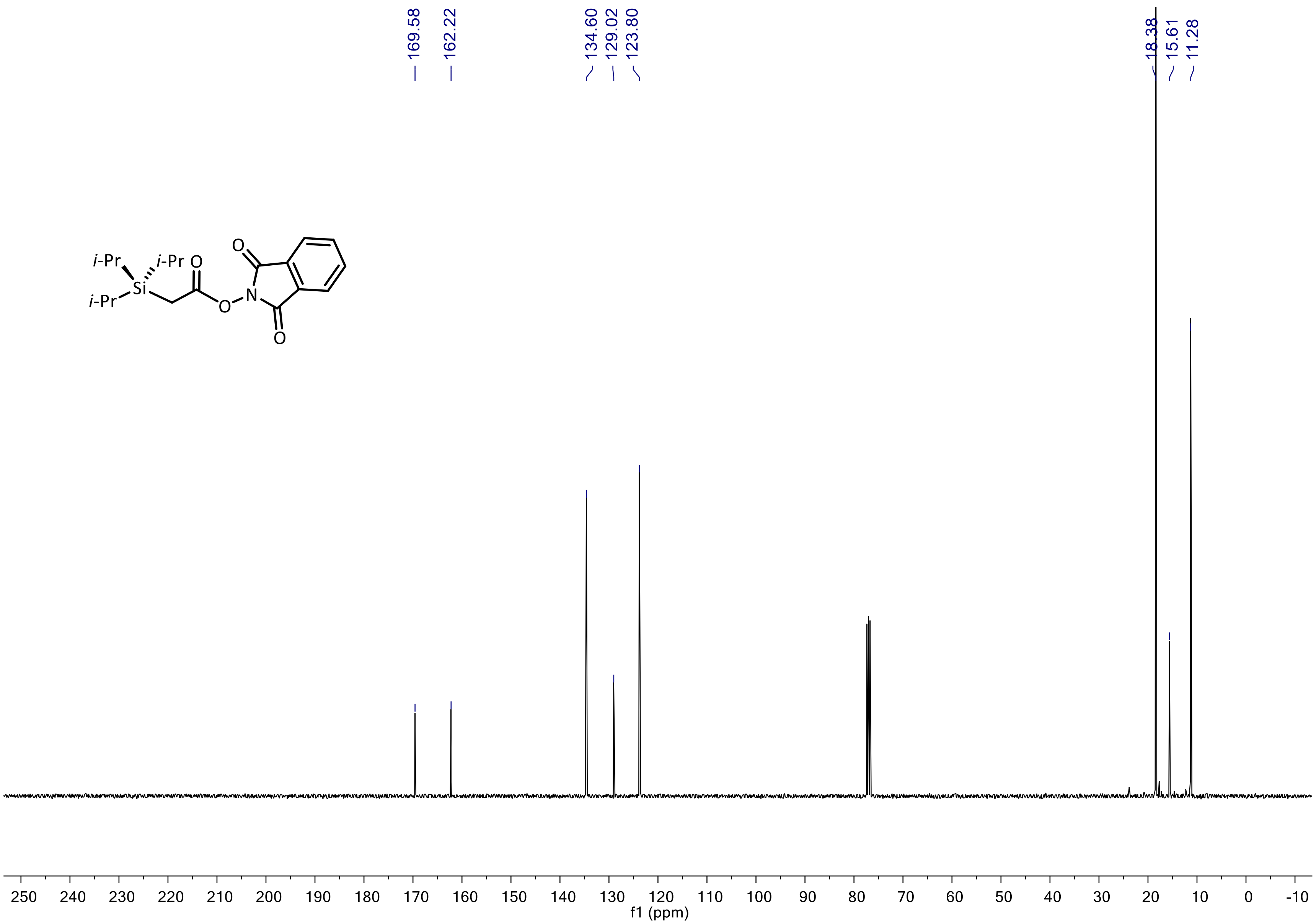

^1H-NMR (400 MHz, $CDCl_3$) for compound **9b**

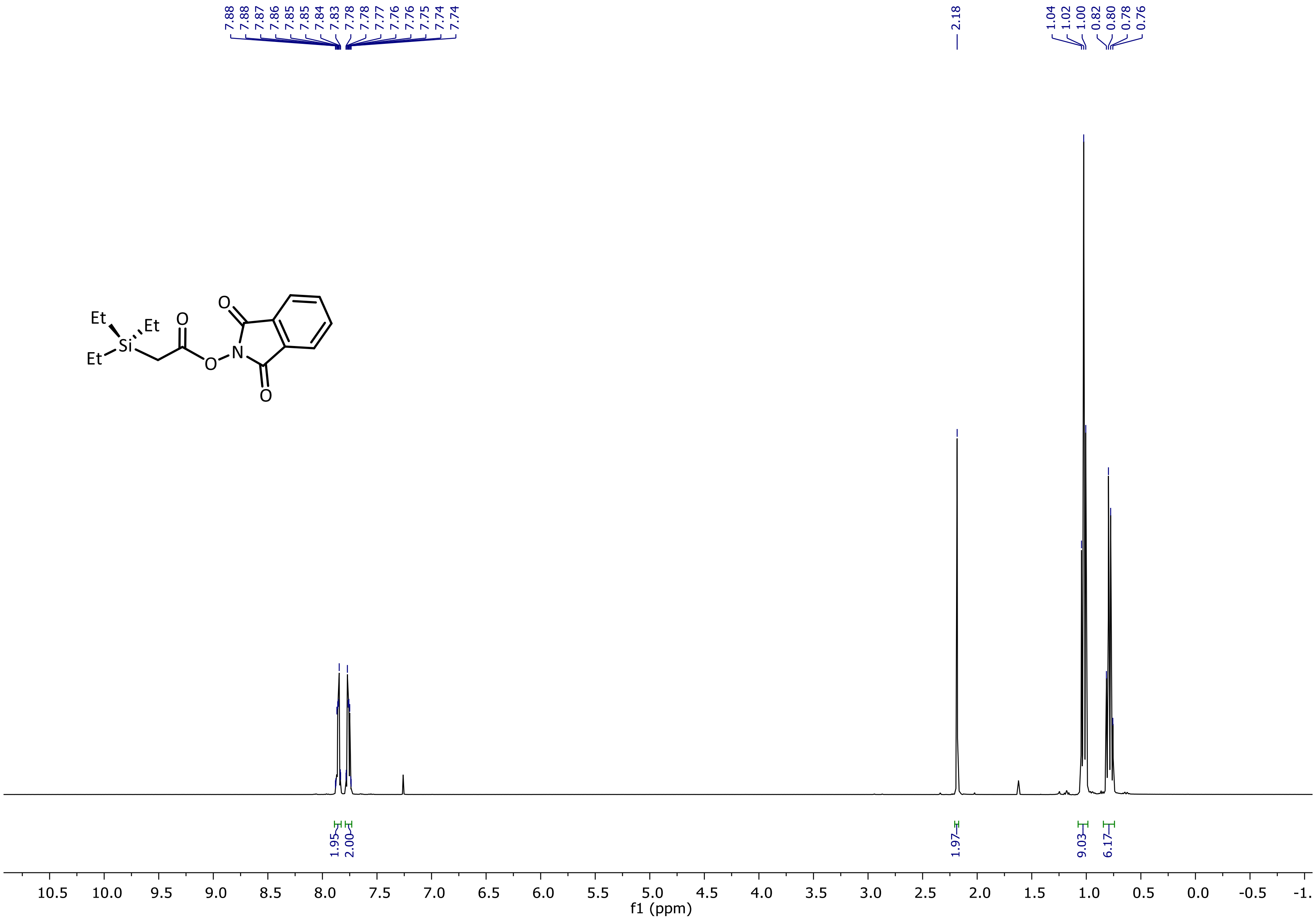

*^{13}C-NMR (101 MHz, $CDCl_3$) for compound **9b***

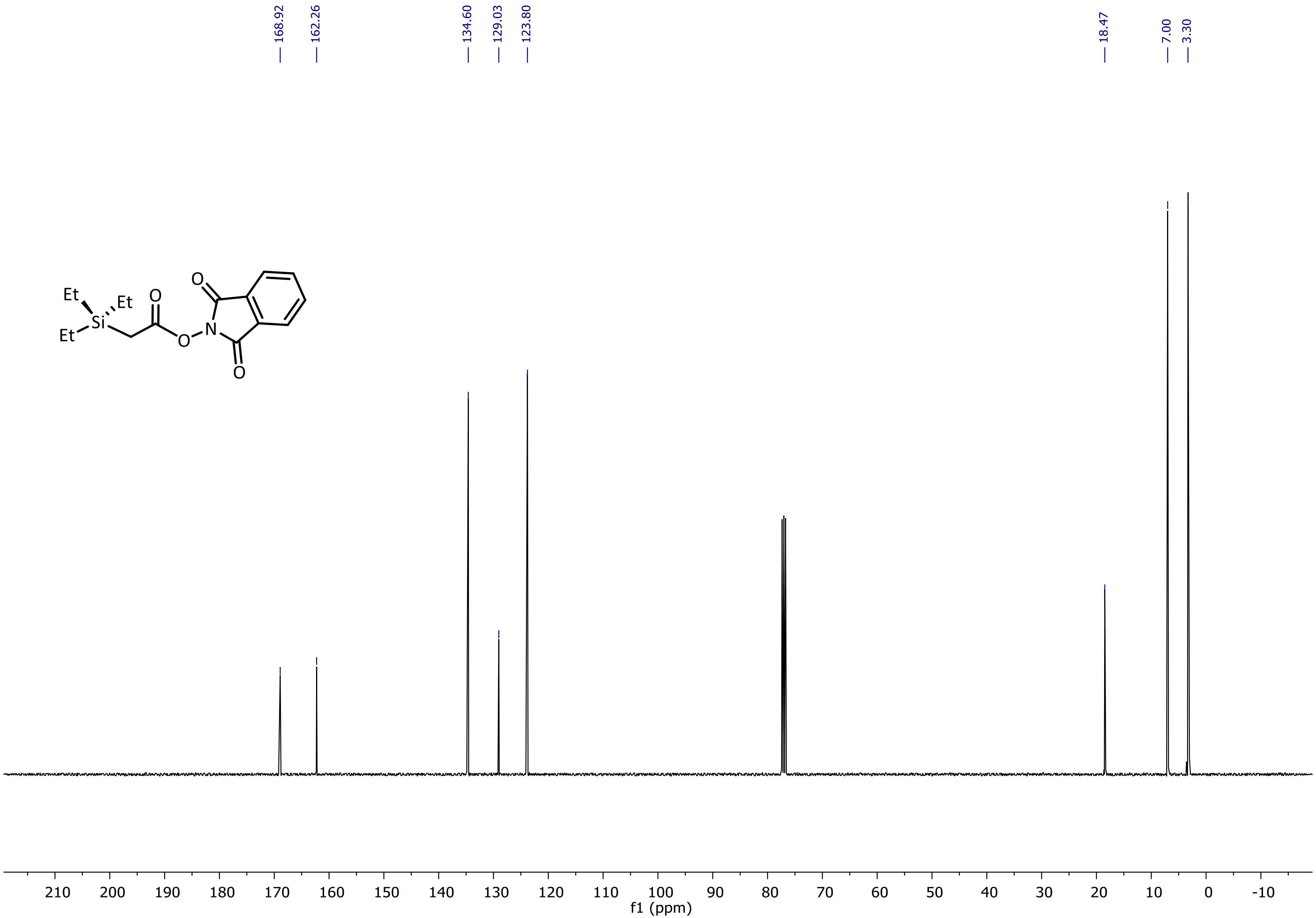

^{1}H-NMR (400 MHz, $CDCl_3$) for compound ***9c***

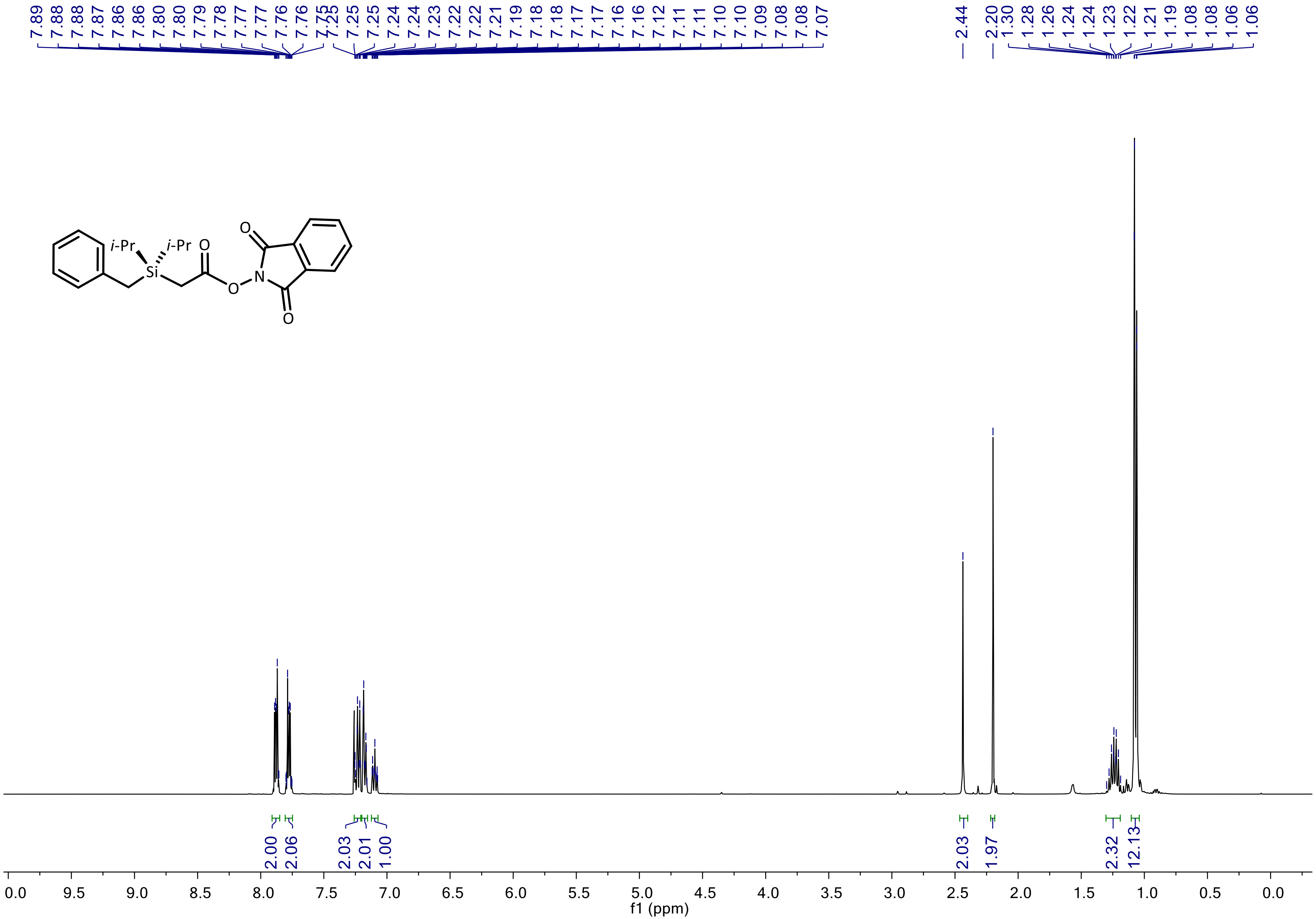

*^{13}C-NMR (101 MHz, $CDCl_3$) for compound **9c***

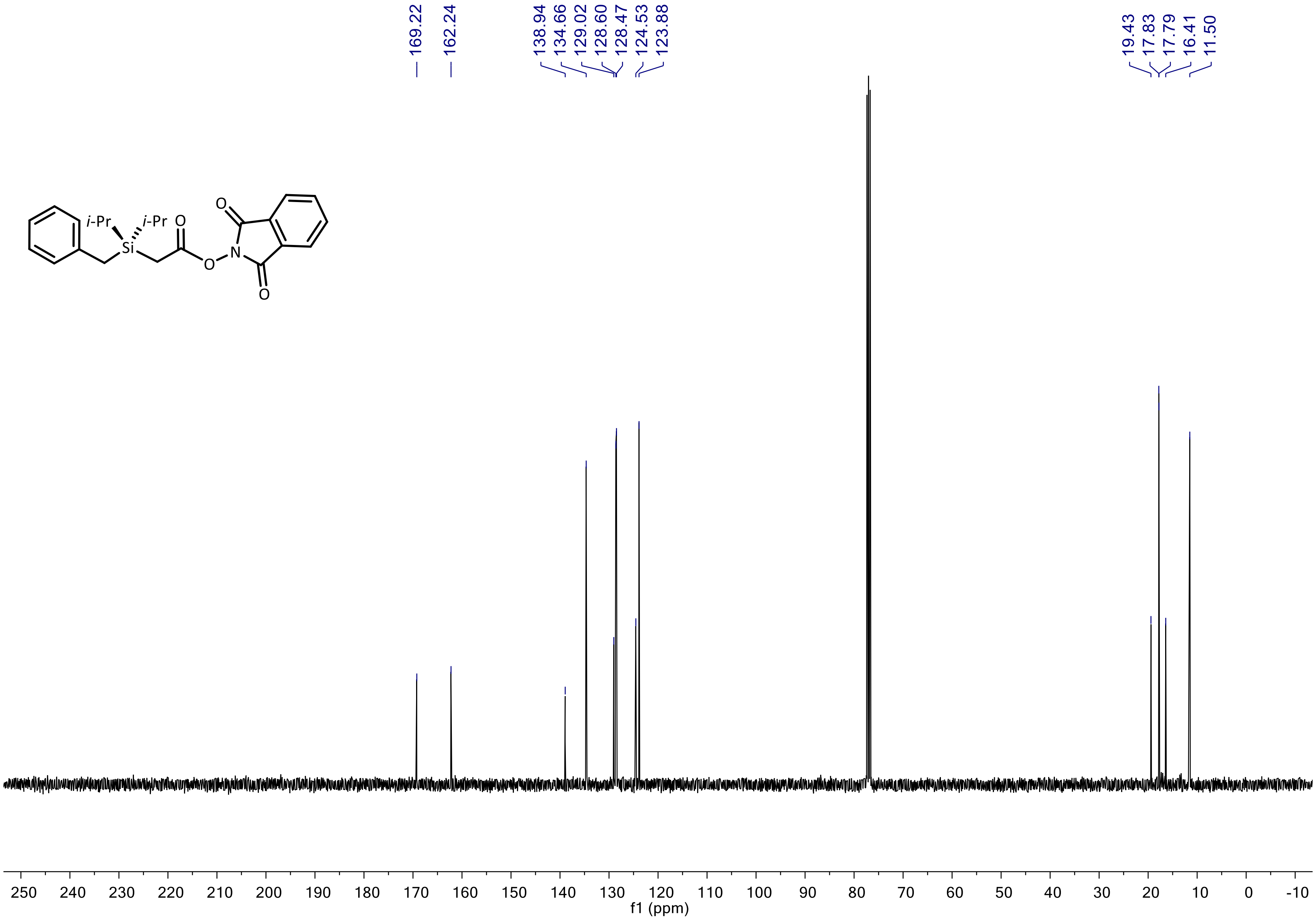

*¹H-NMR (400 MHz, CDCl₃) for compound **9d***

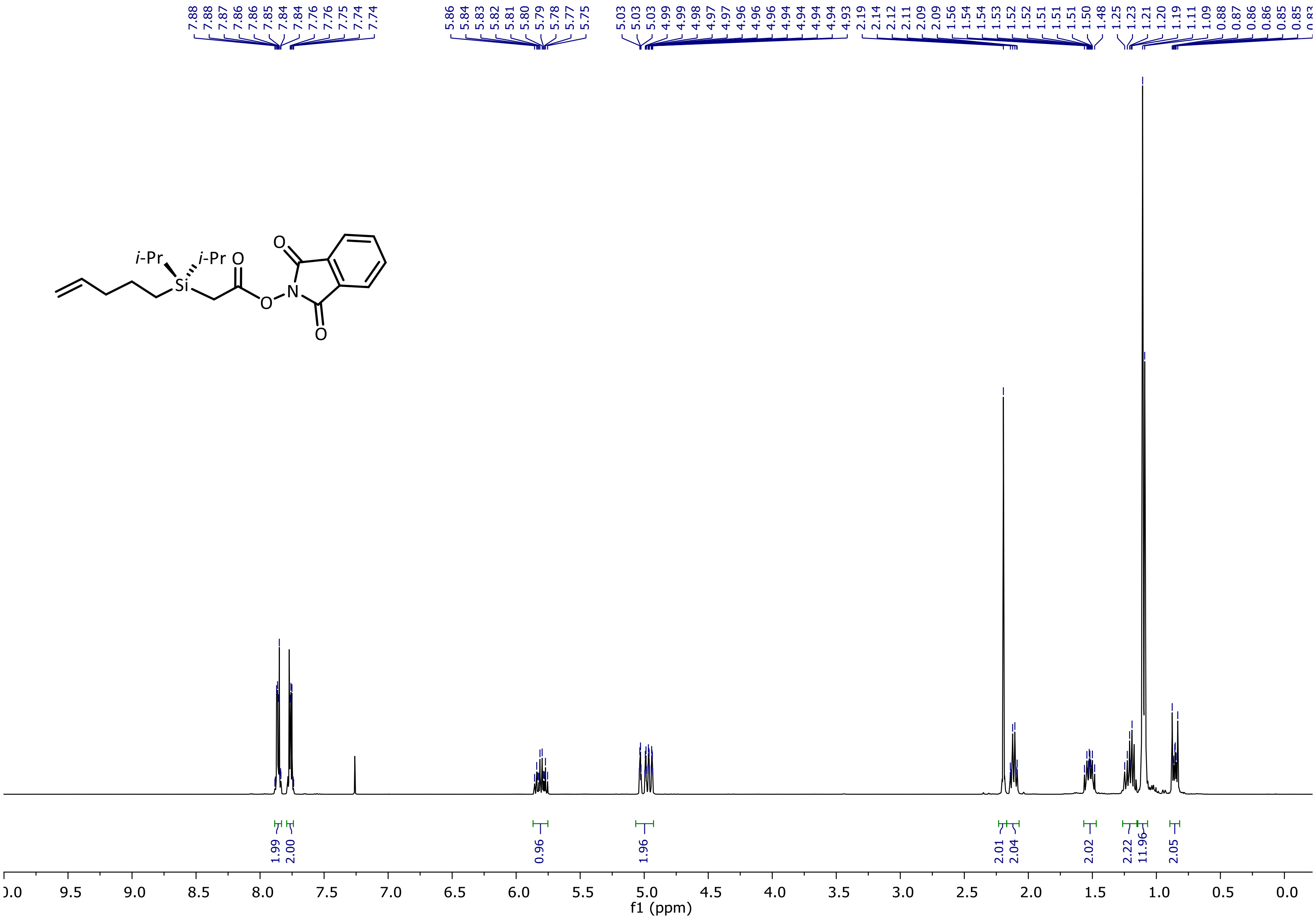

^{13}C-NMR (101 MHz, $CDCl_3$) for compound **9d**

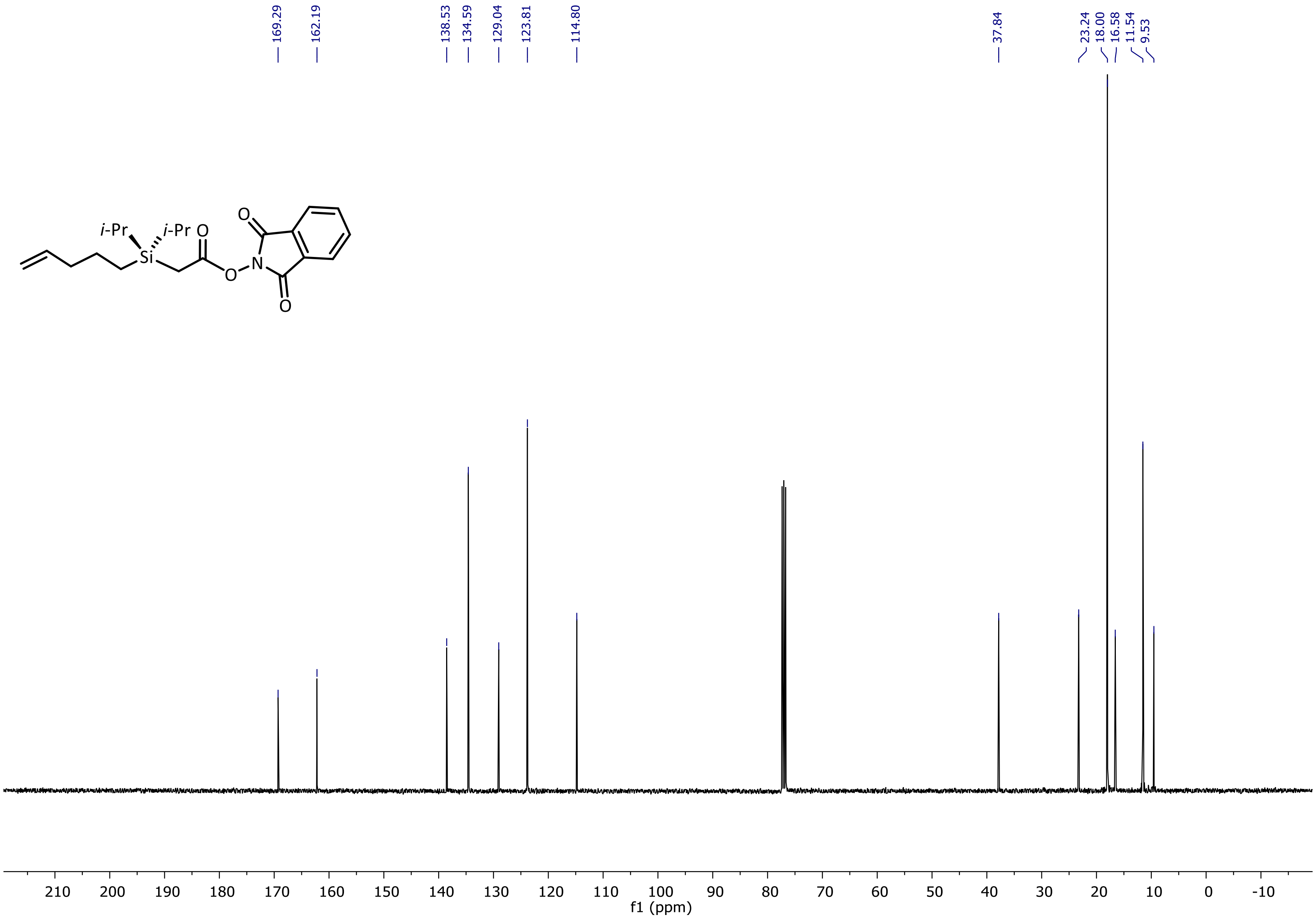

*^{1}H-NMR (400 MHz, $CDCl_3$) for compound **9e***

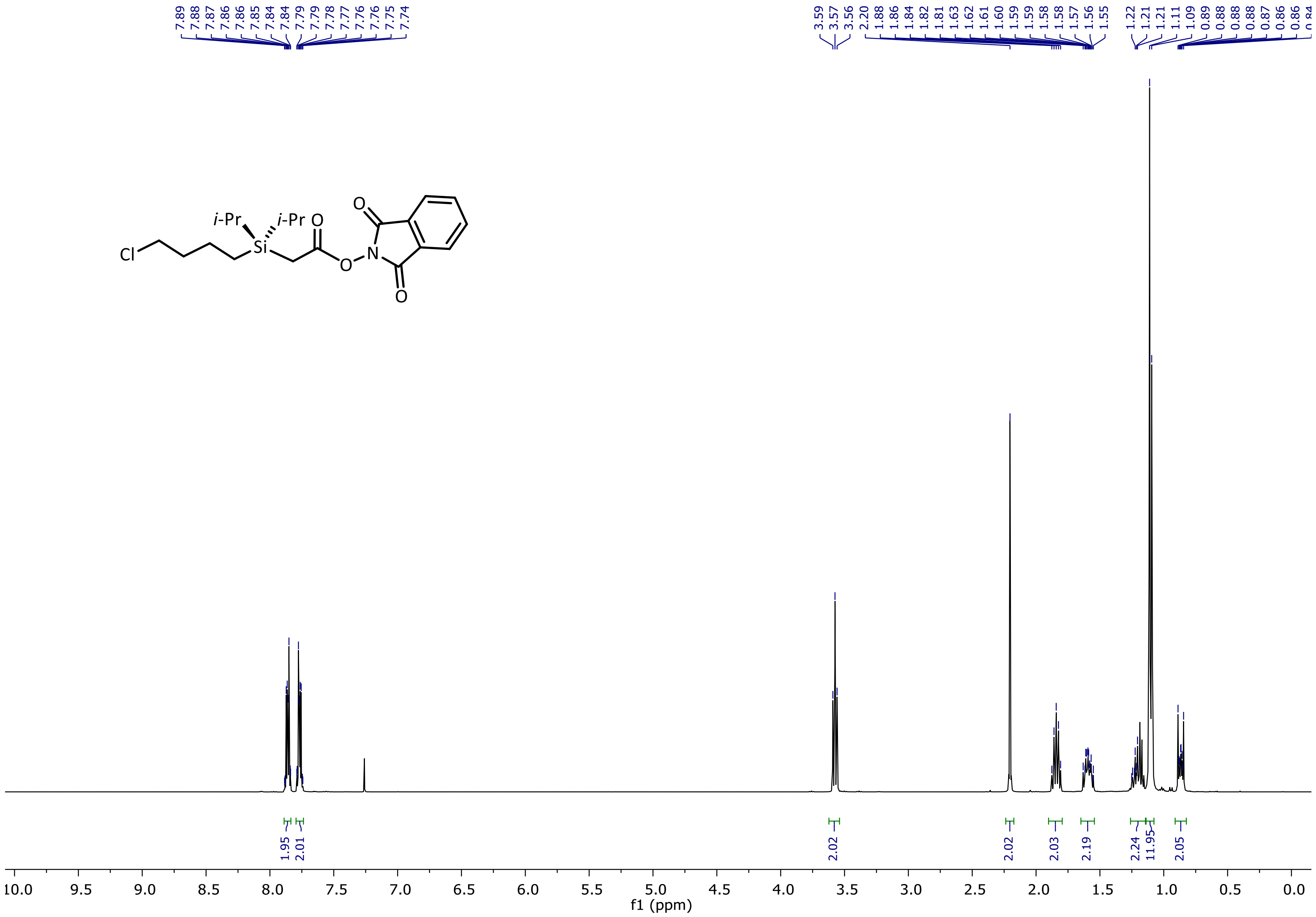

^{13}C-NMR (101 MHz, CDCl$_3$) for compound ***9e***

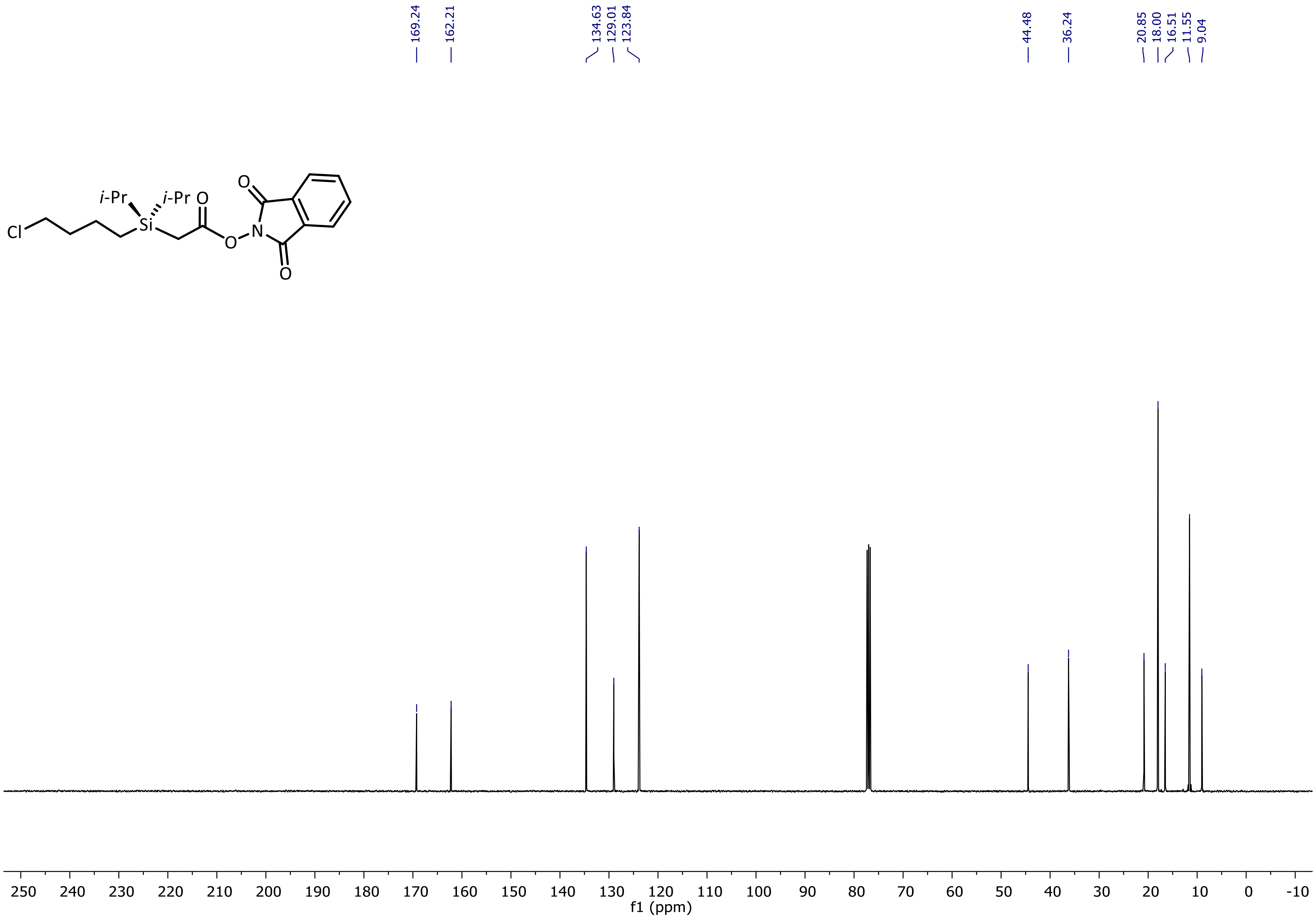

*^{1}H-NMR (400 MHz, CDCl$_3$) for compound **9f***

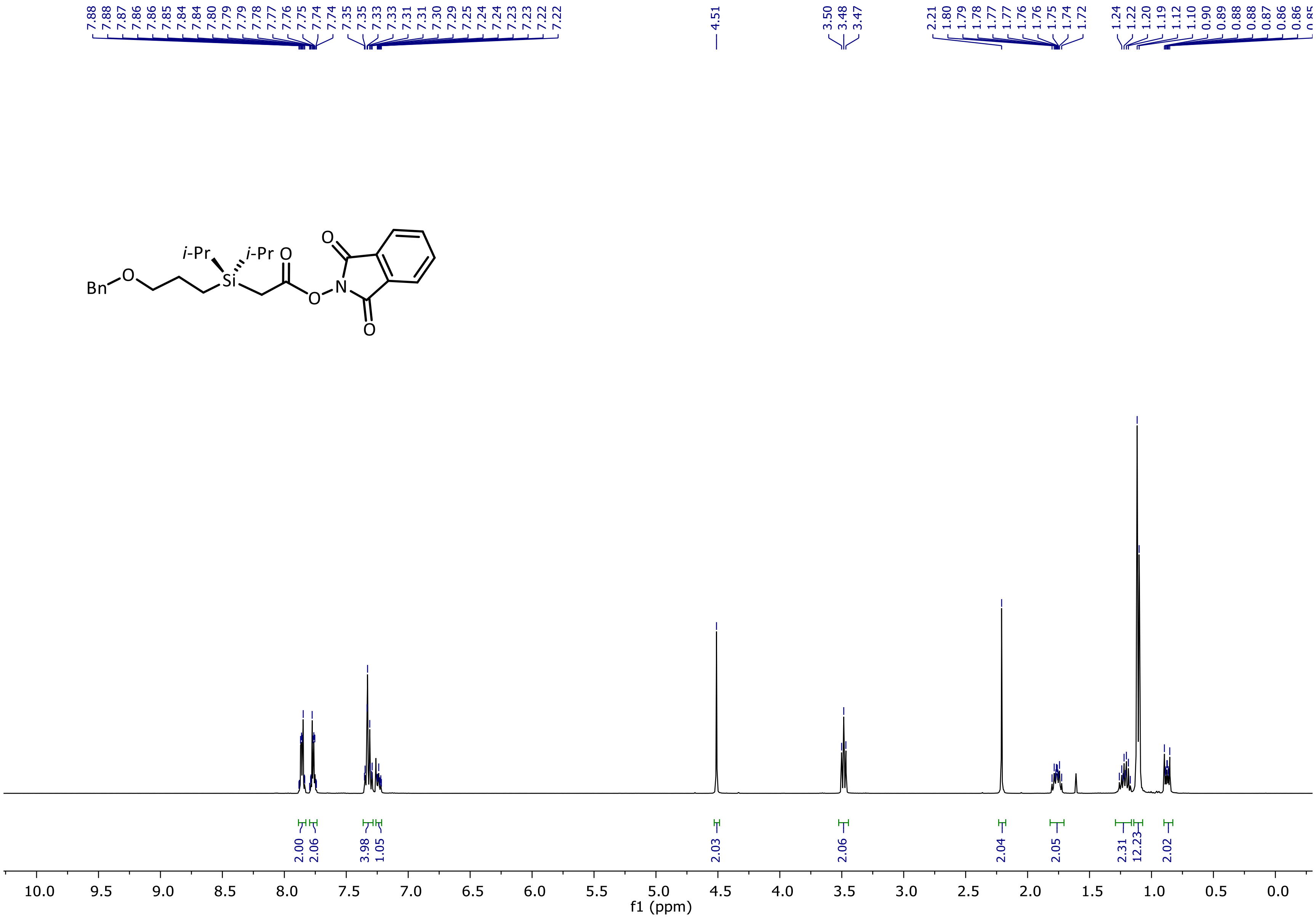

^{13}C-NMR (101 MHz, $CDCl_3$) *for compound* ***9f***

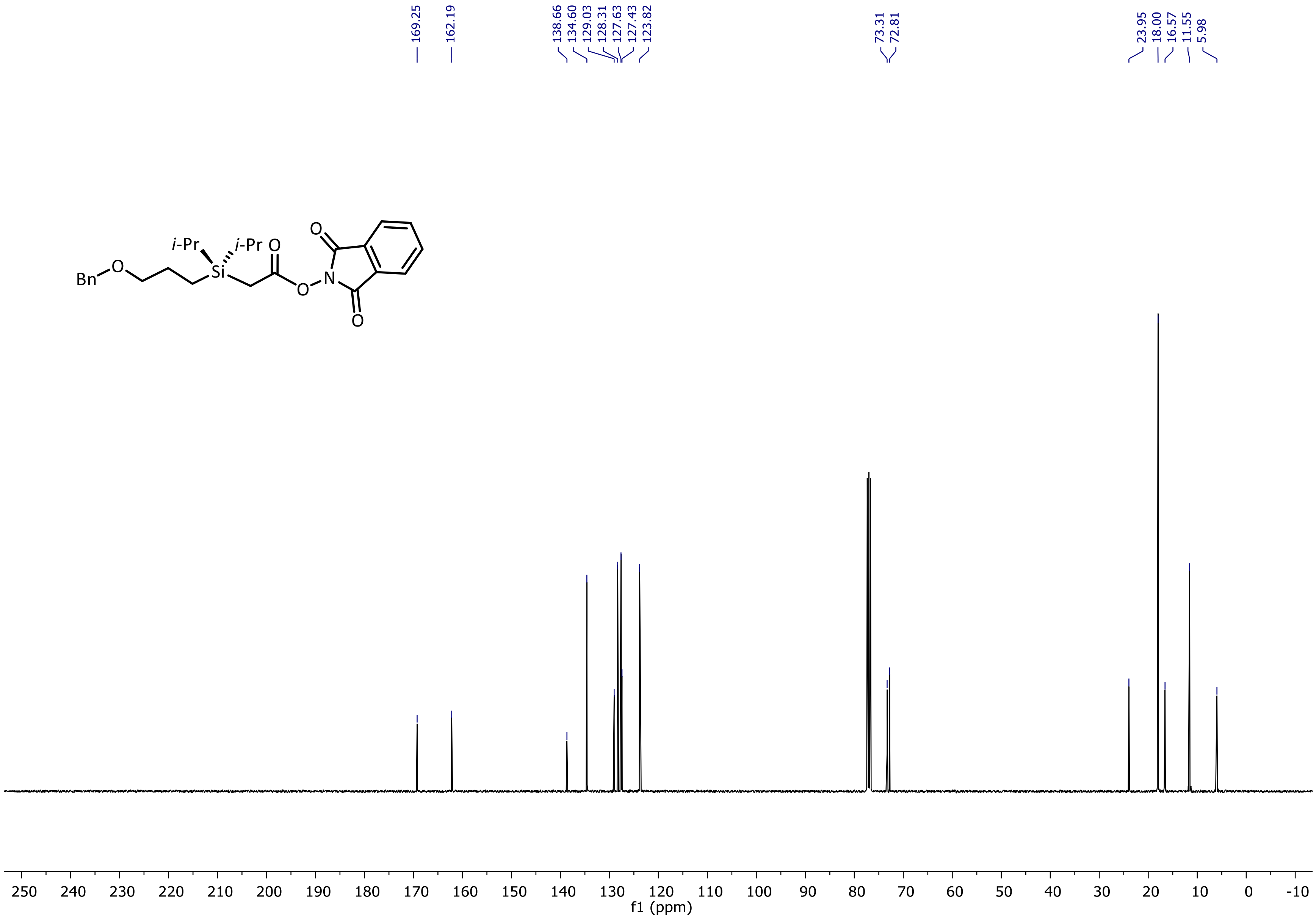

^{1}H-NMR (400 MHz, CDCl$_3$) for compound ***9g***

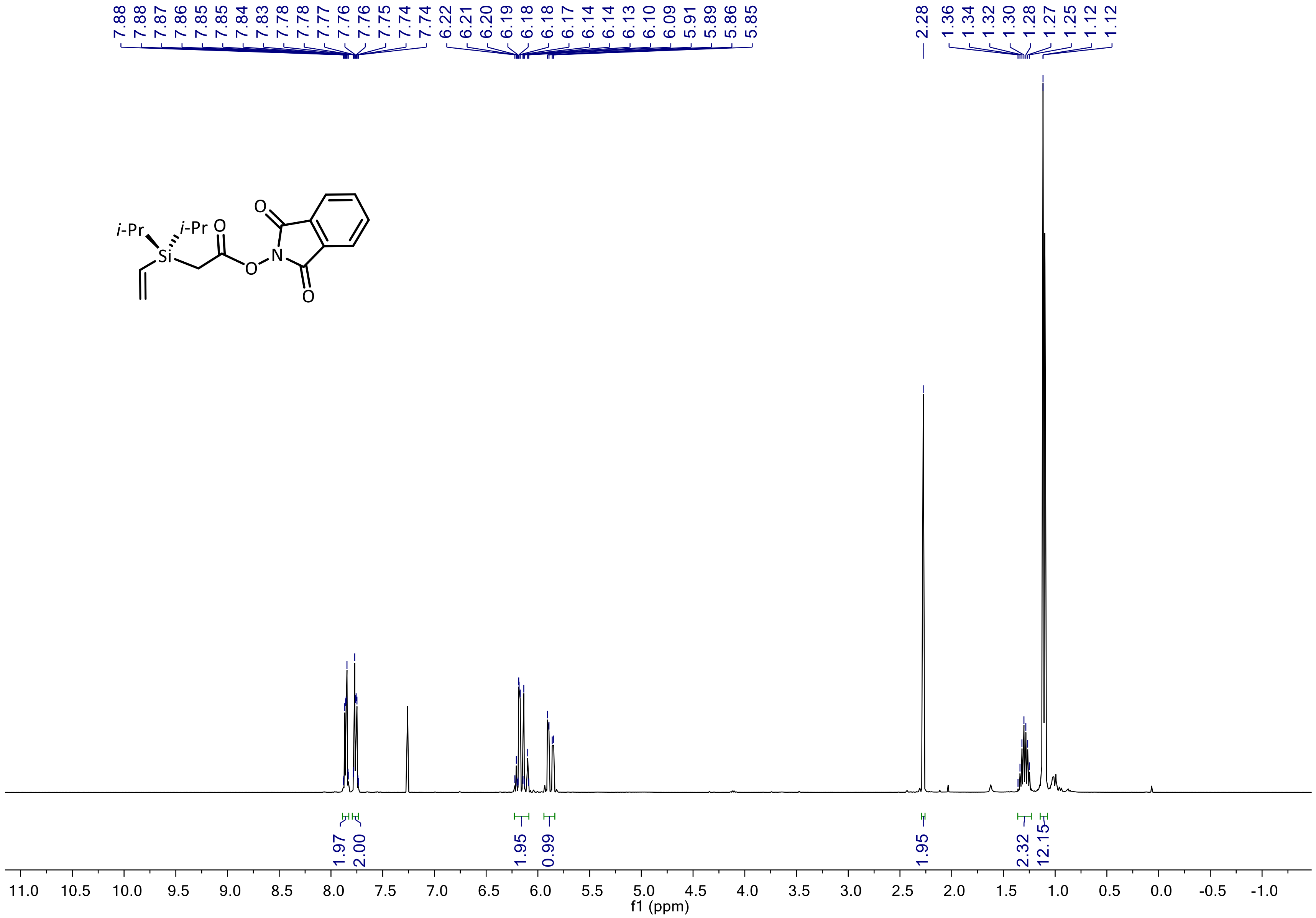

^{13}C-NMR (101 MHz, CDCl$_3$) for compound **9g**

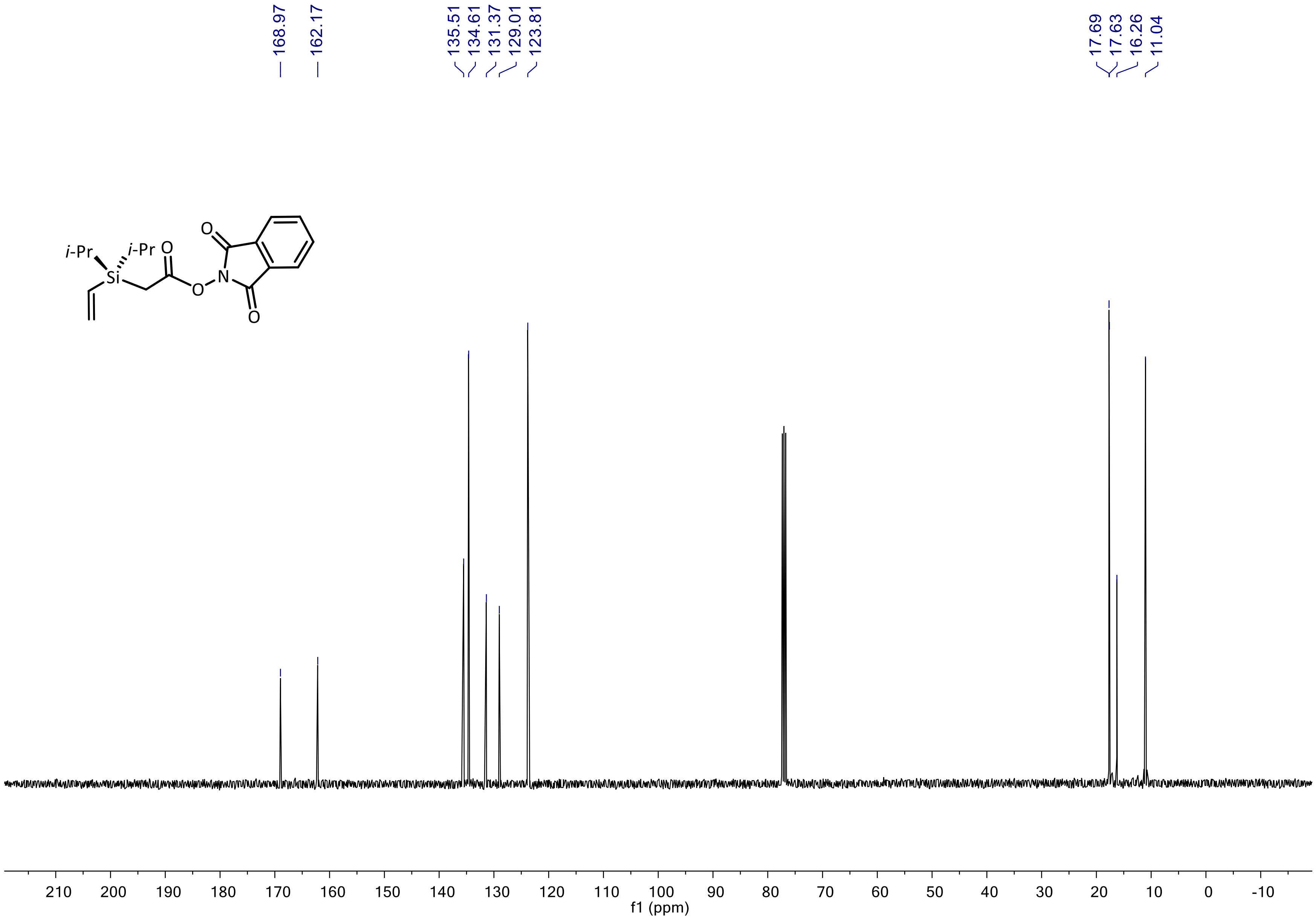

*^{1}H-NMR (400 MHz, $CDCl_3$) for compound **9h***

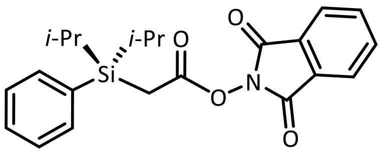

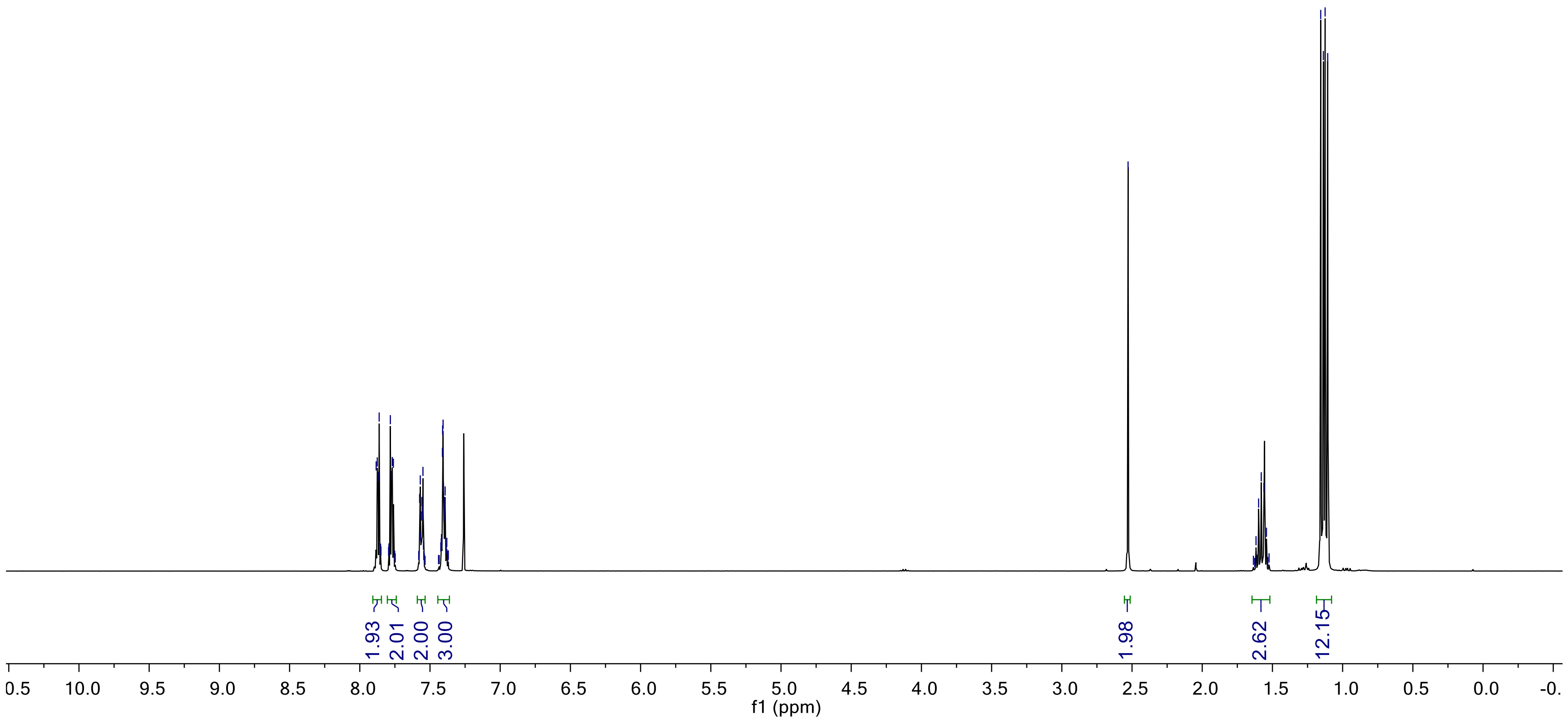

^{13}C-NMR (101 MHz, $CDCl_3$) for compound **9h**

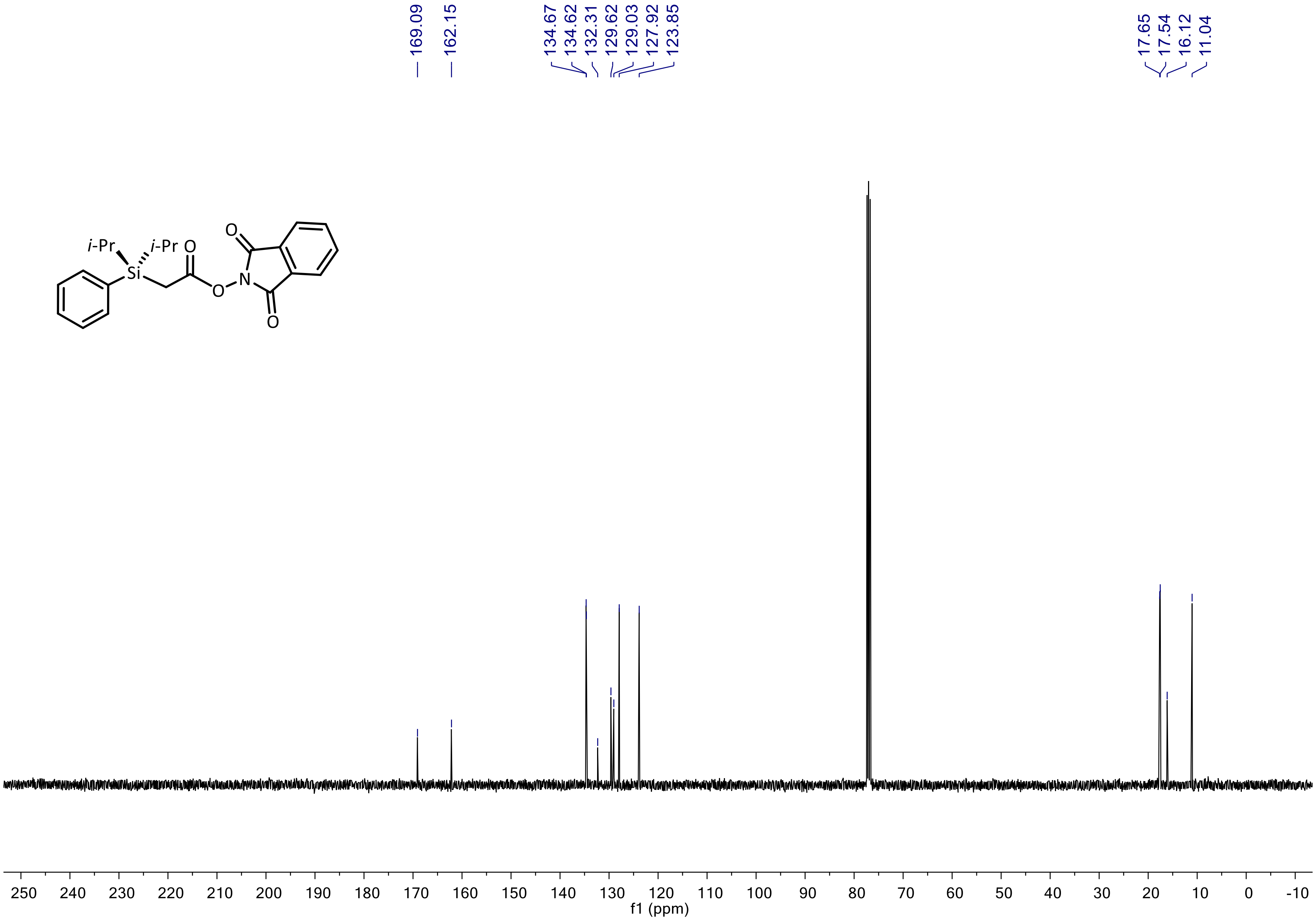

^{1}H-NMR (400 MHz, CDCl$_3$) for compound ***9i***

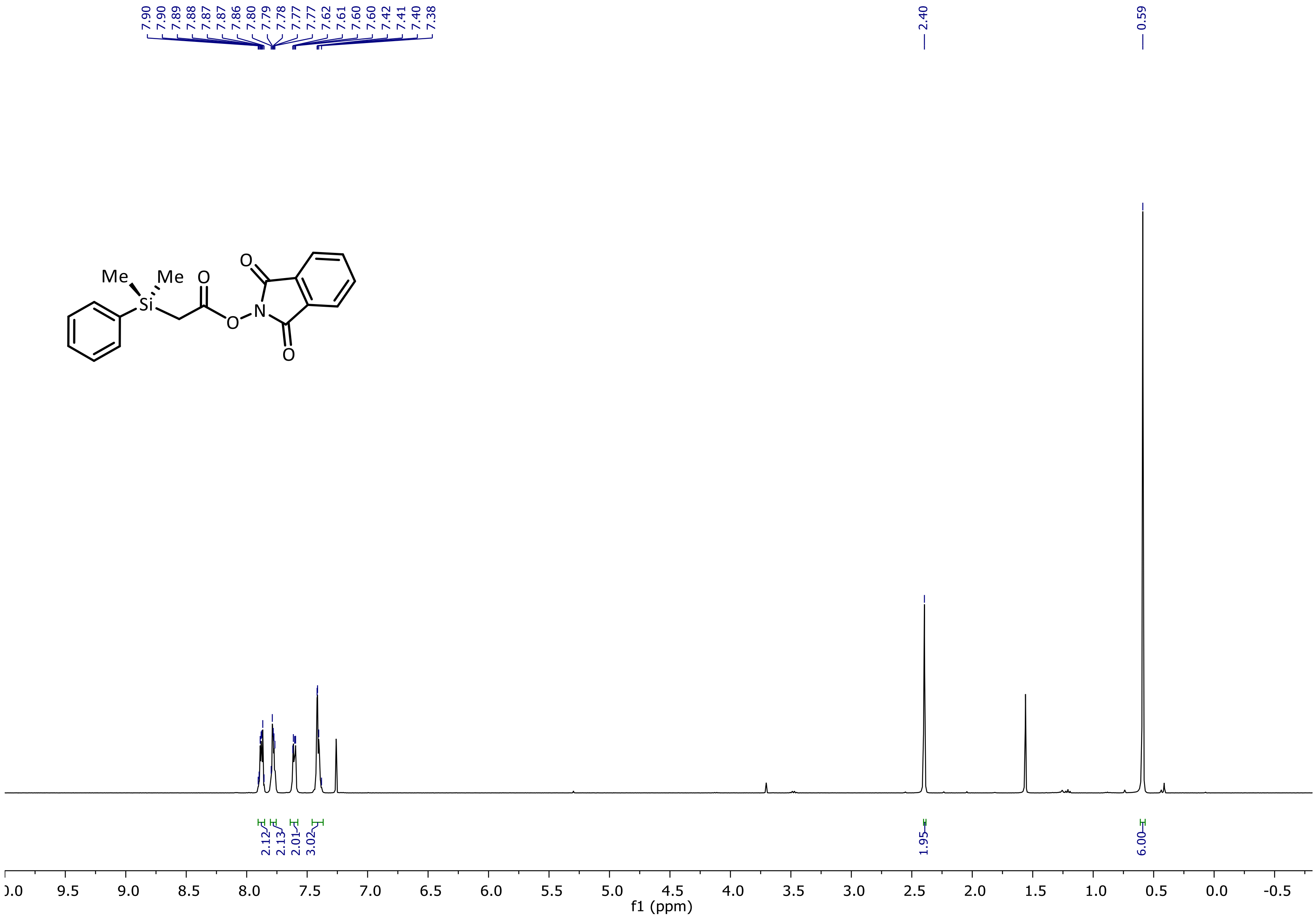

*^{13}C-NMR (101 MHz, CDCl$_3$) for compound **9i***

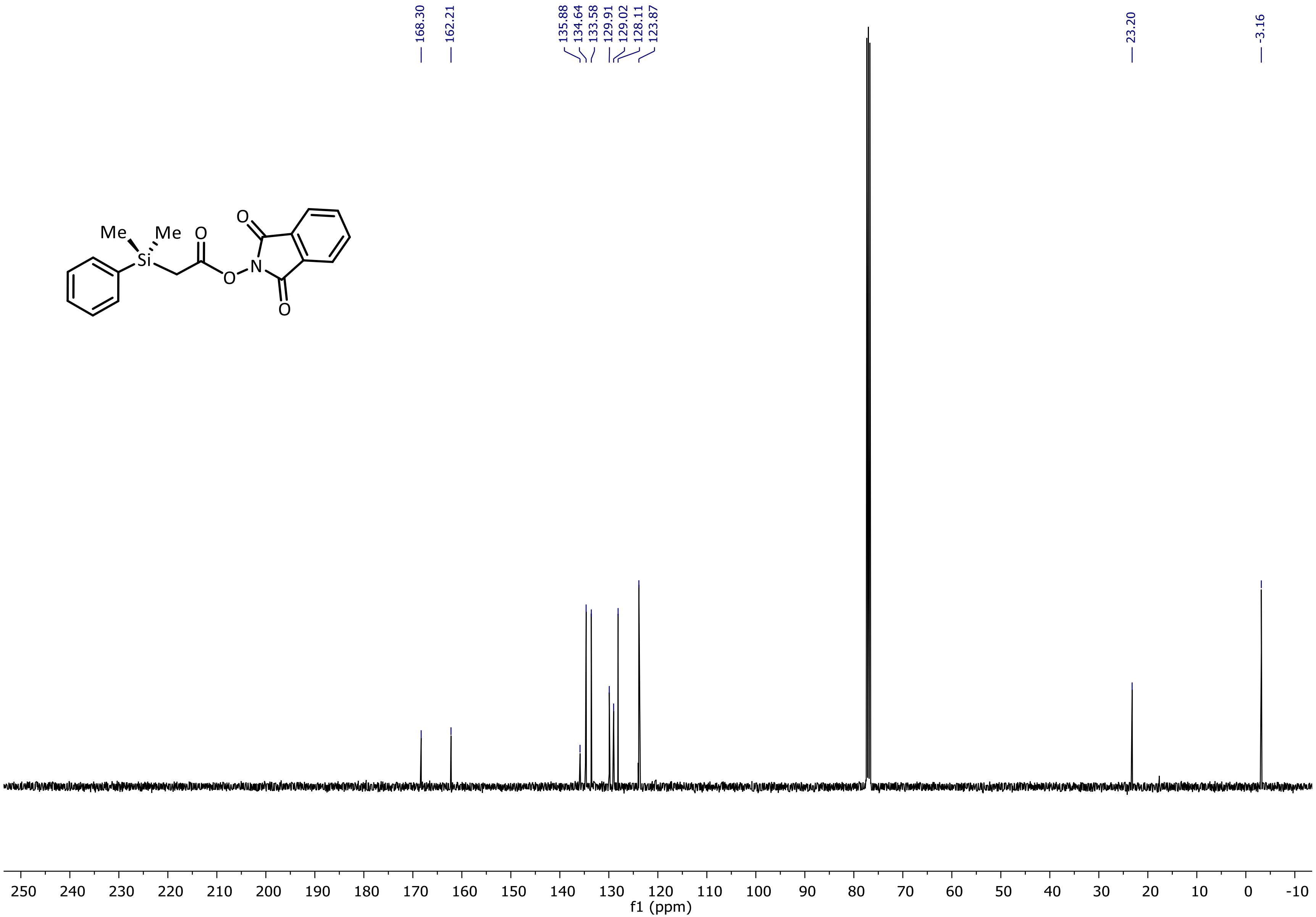

^{1}H-NMR (400 MHz, $CDCl_3$) for compound ***9j***

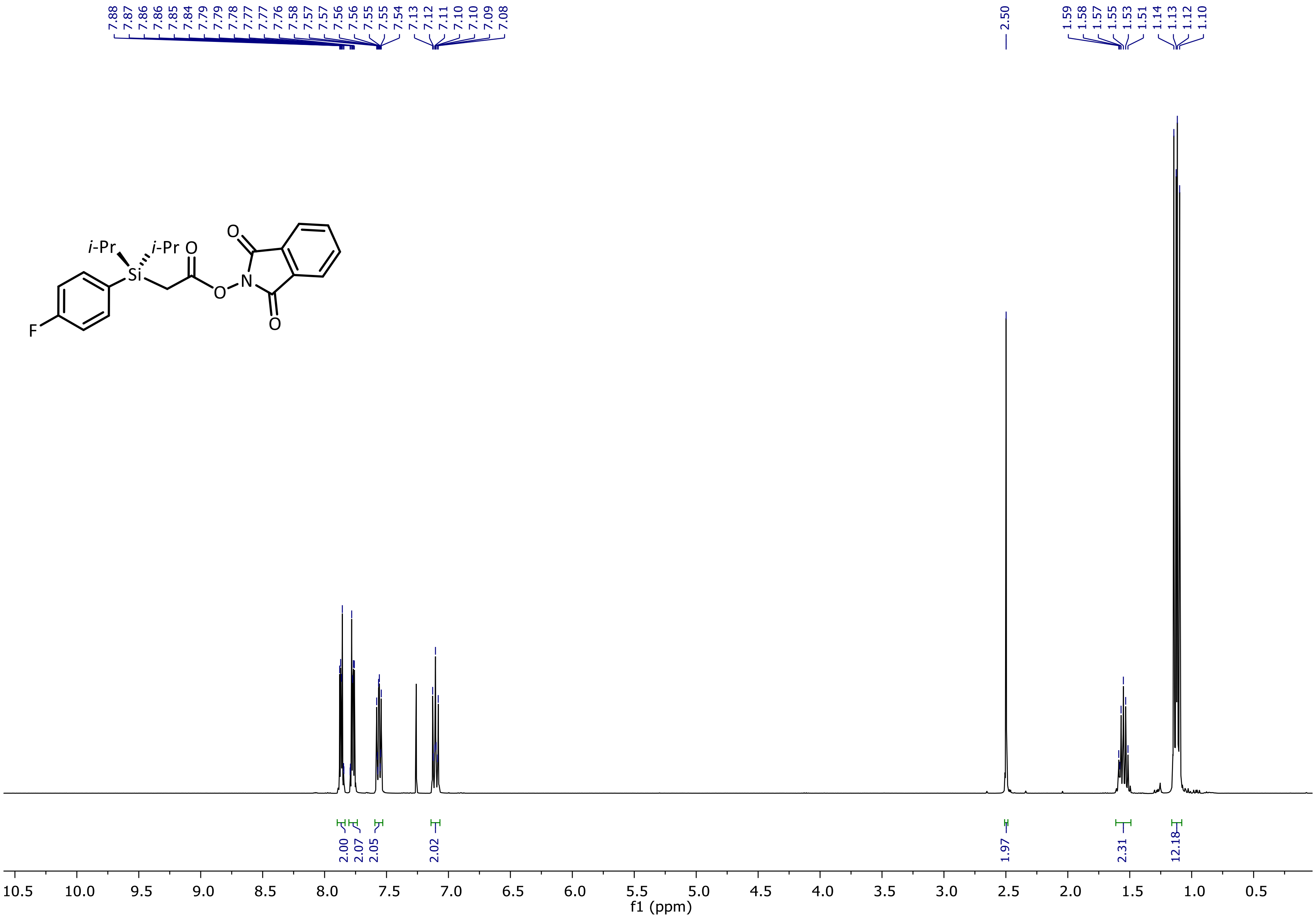

^{13}C-NMR (101 MHz, $CDCl_3$) for compound ***9j***

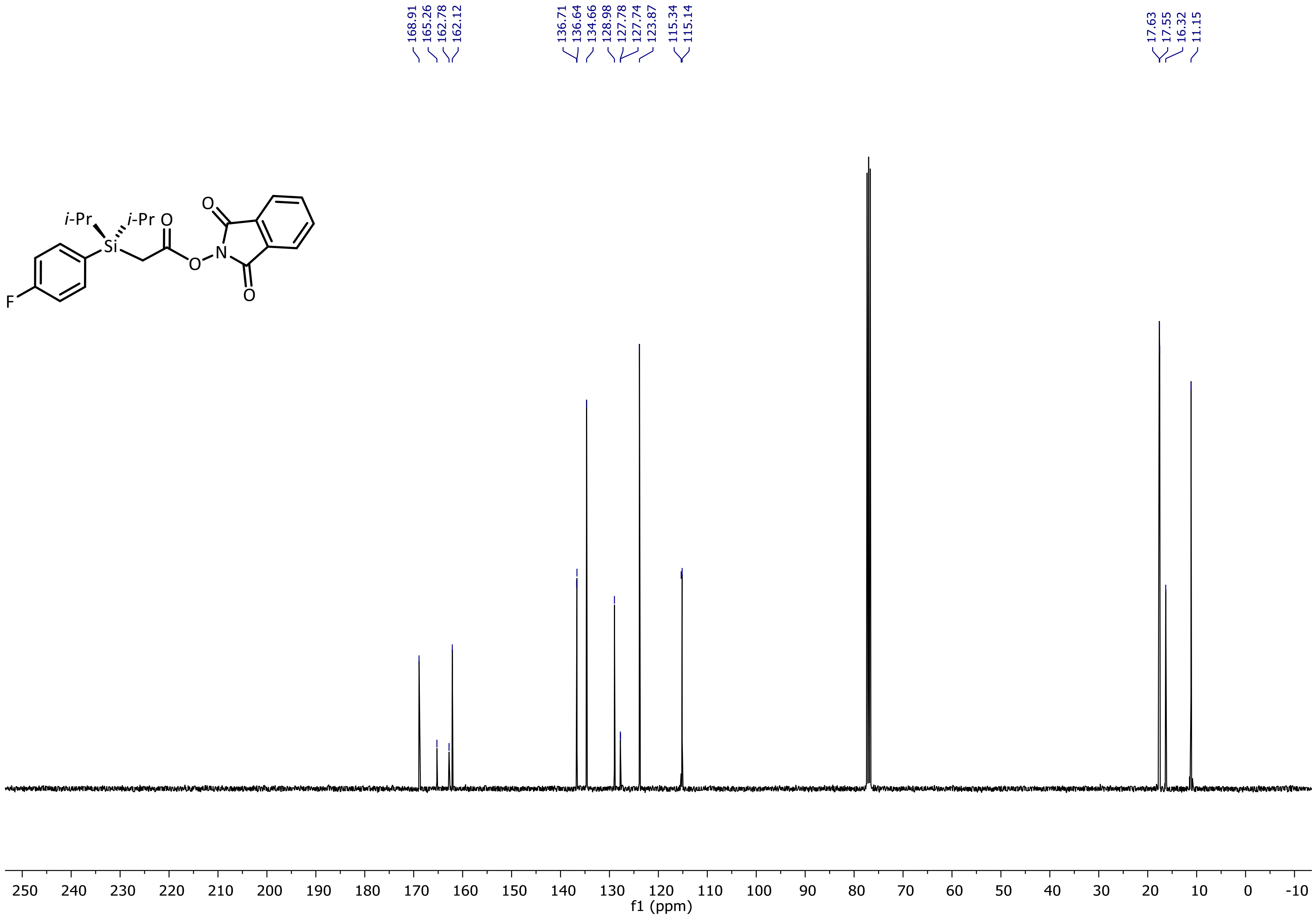

*^{19}F-NMR (377 MHz, $CDCl_3$) for compound **9j***

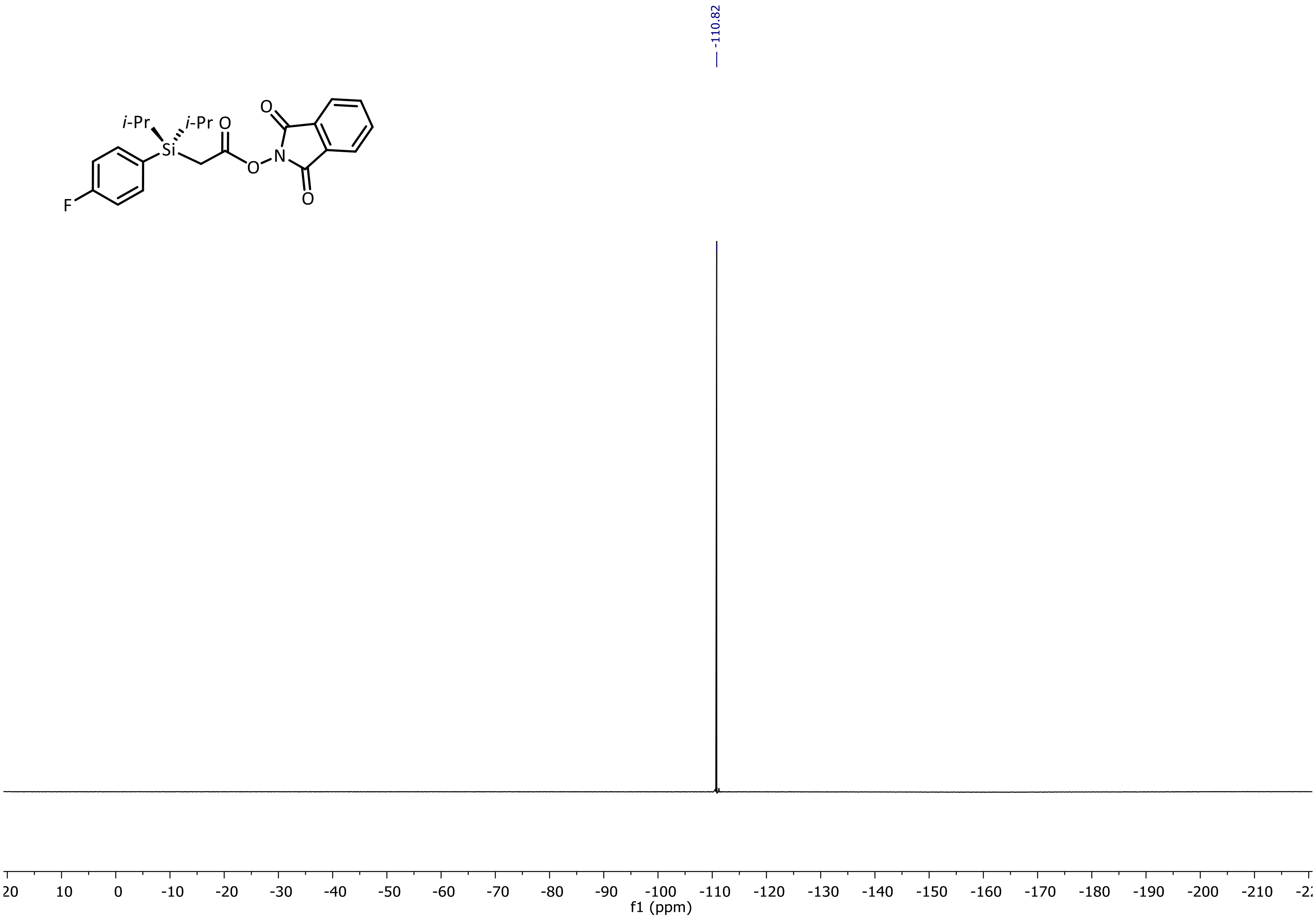

^{1}H-NMR (400 MHz, $CDCl_3$) for compound ***9k***

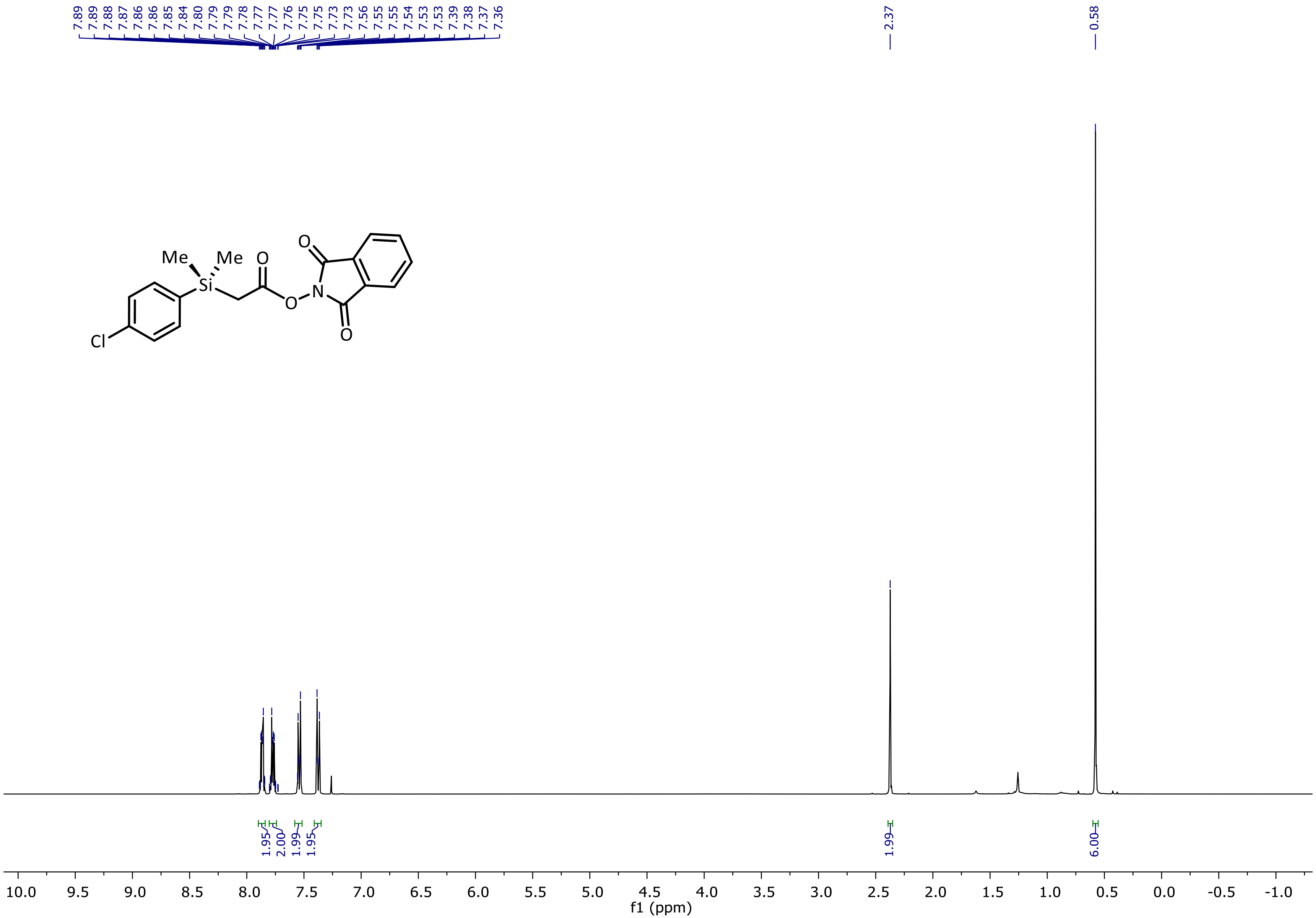

^{13}C-NMR (101 MHz, $CDCl_3$) for compound ***9k***

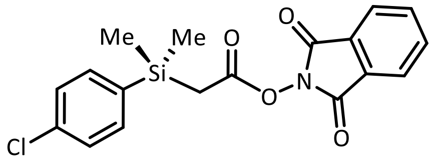

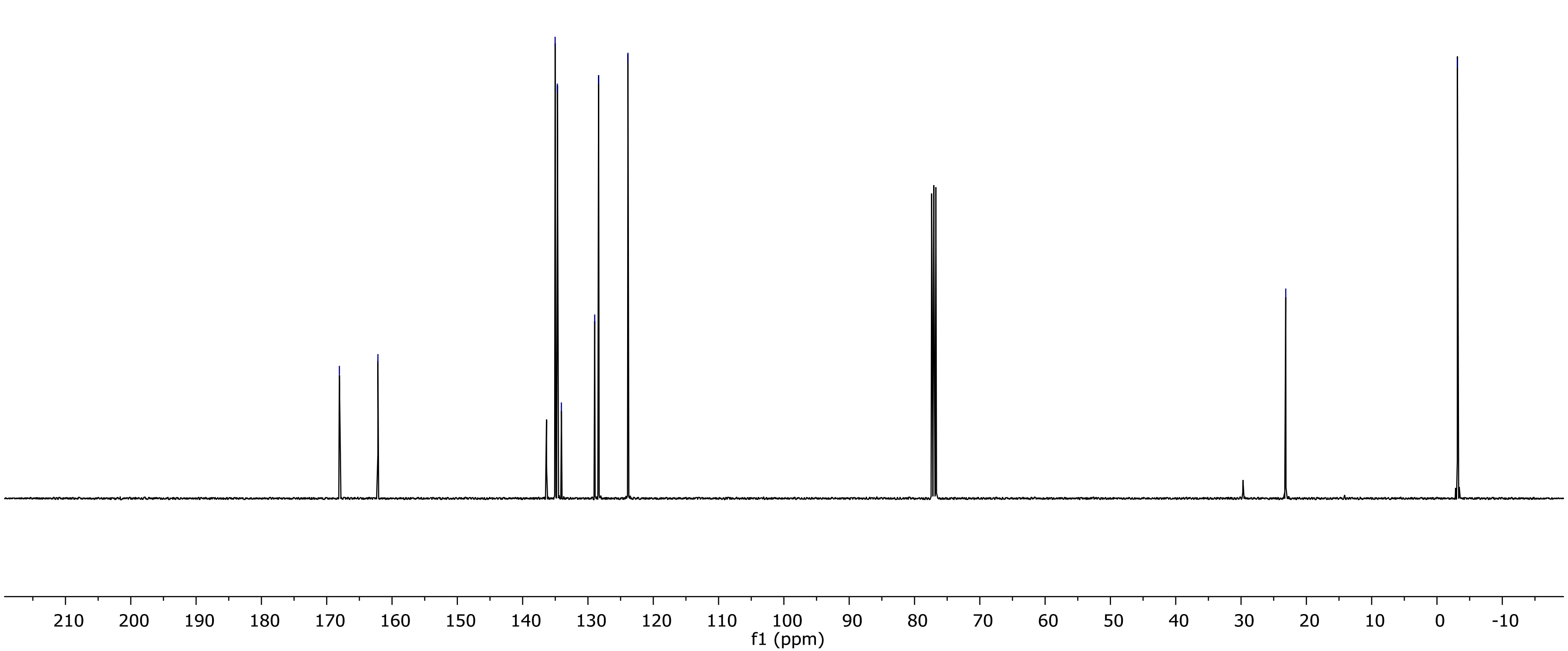

^{1}H-NMR (400 MHz, $CDCl_3$) for compound ***9l***

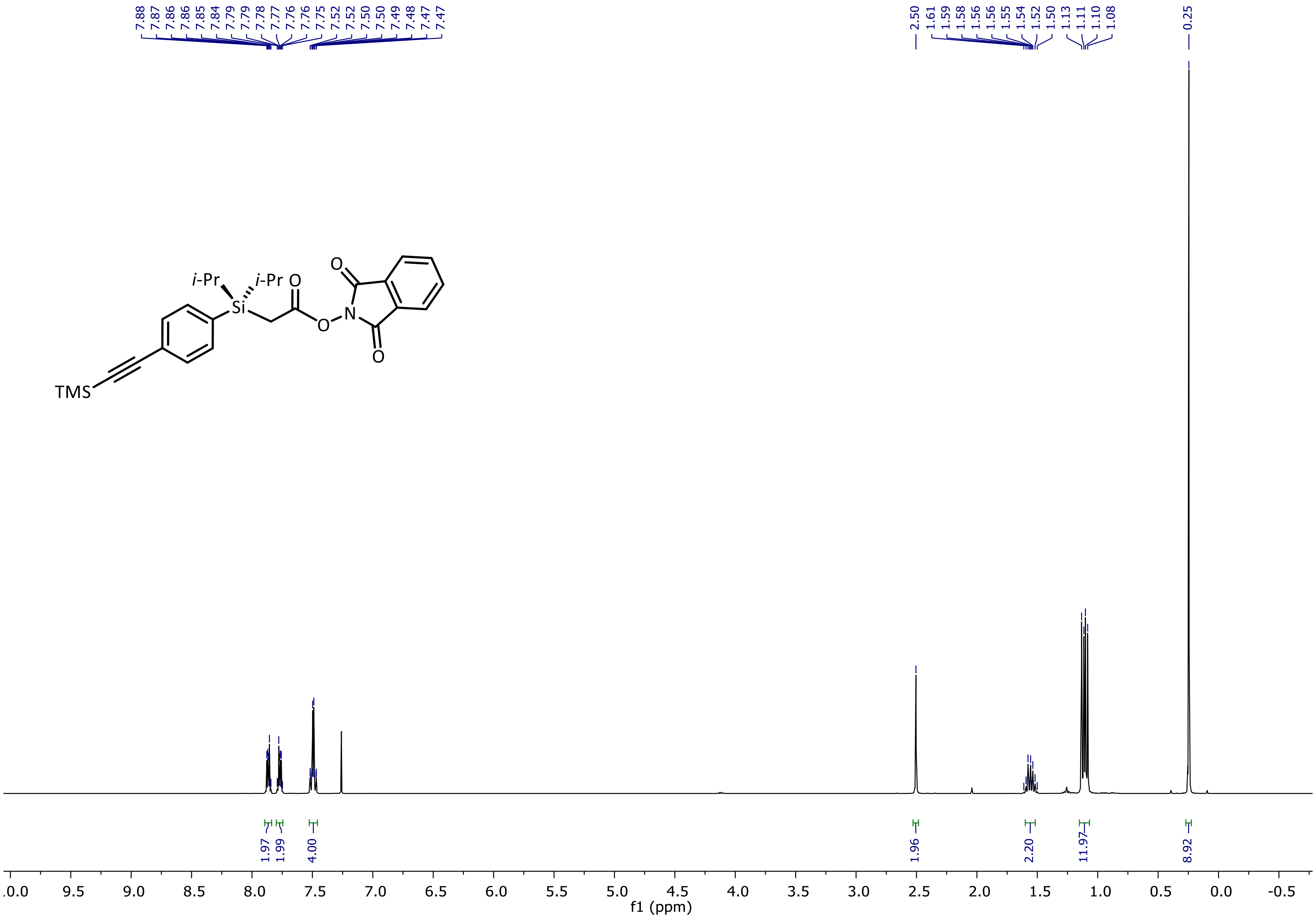

*^{13}C-NMR (101 MHz, CDCl$_3$) for compound **9l***

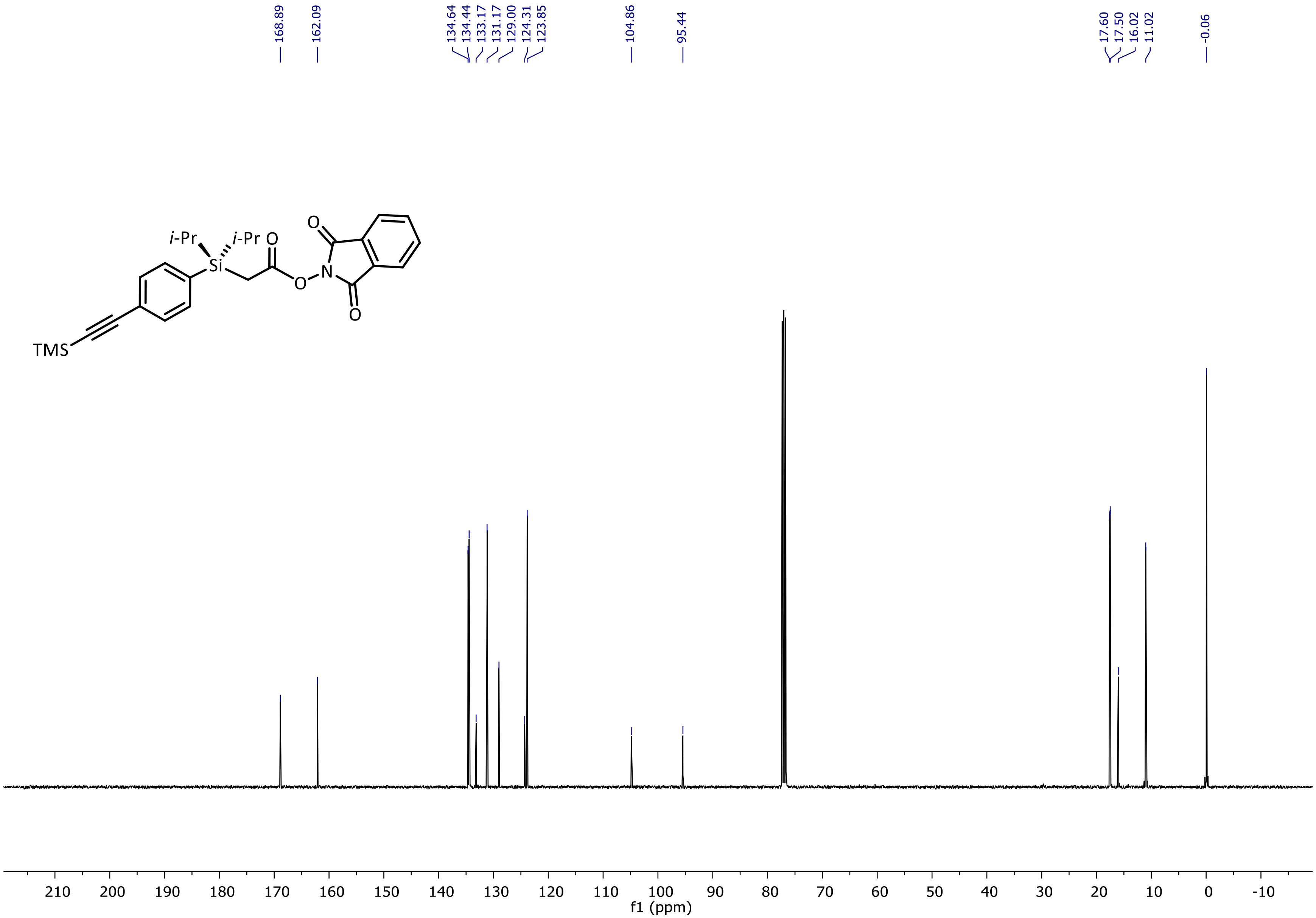

^{1}H-NMR (400 MHz, CDCl$_3$) for compound ***9m***

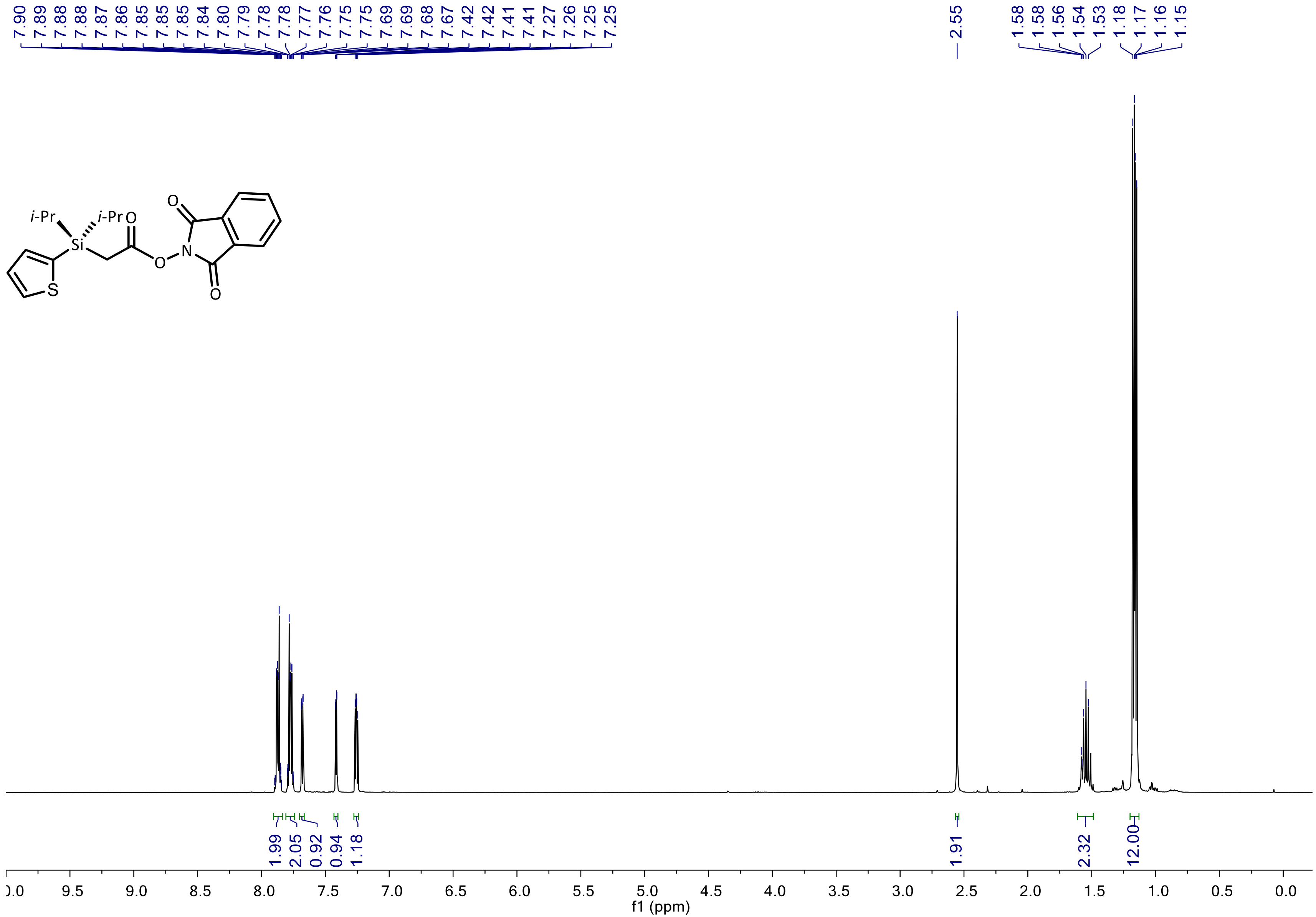

^{13}C-NMR (101 MHz, CDCl$_3$) for compound **9m**

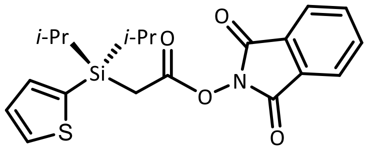

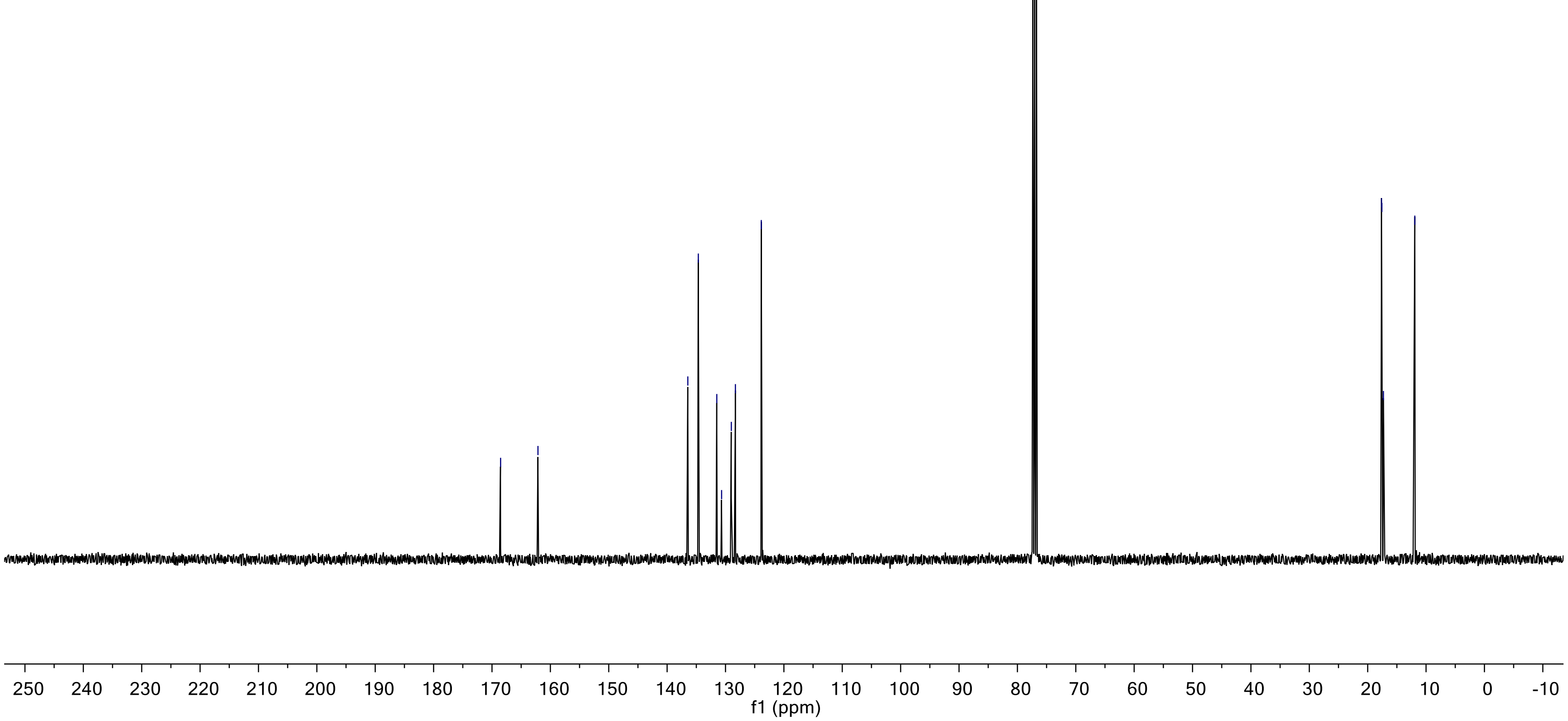

^{1}H-NMR (400 MHz, $CDCl_3$) for compound ***9n***

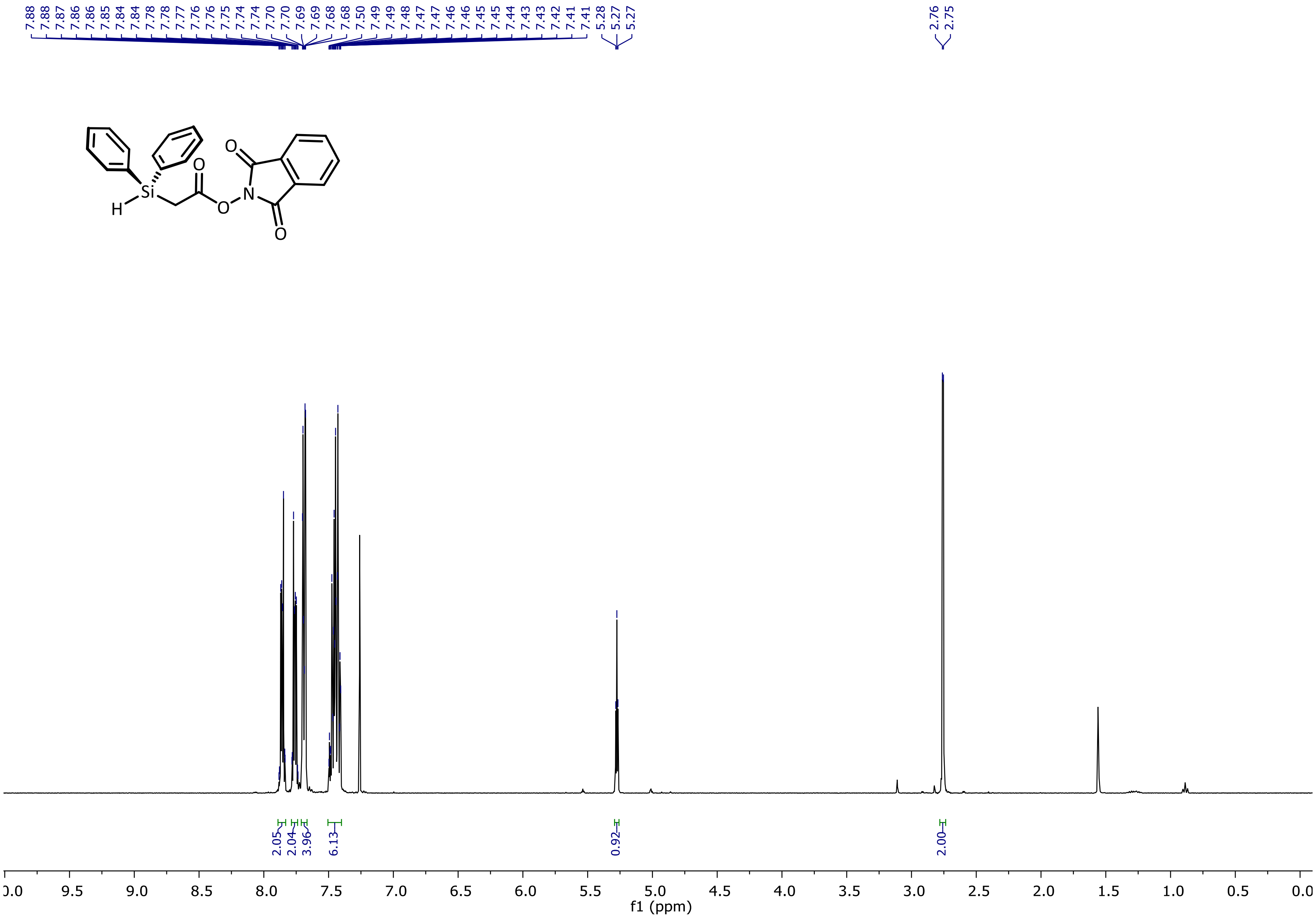

*^{13}C-NMR (101 MHz, CDCl₃) for compound **9n***

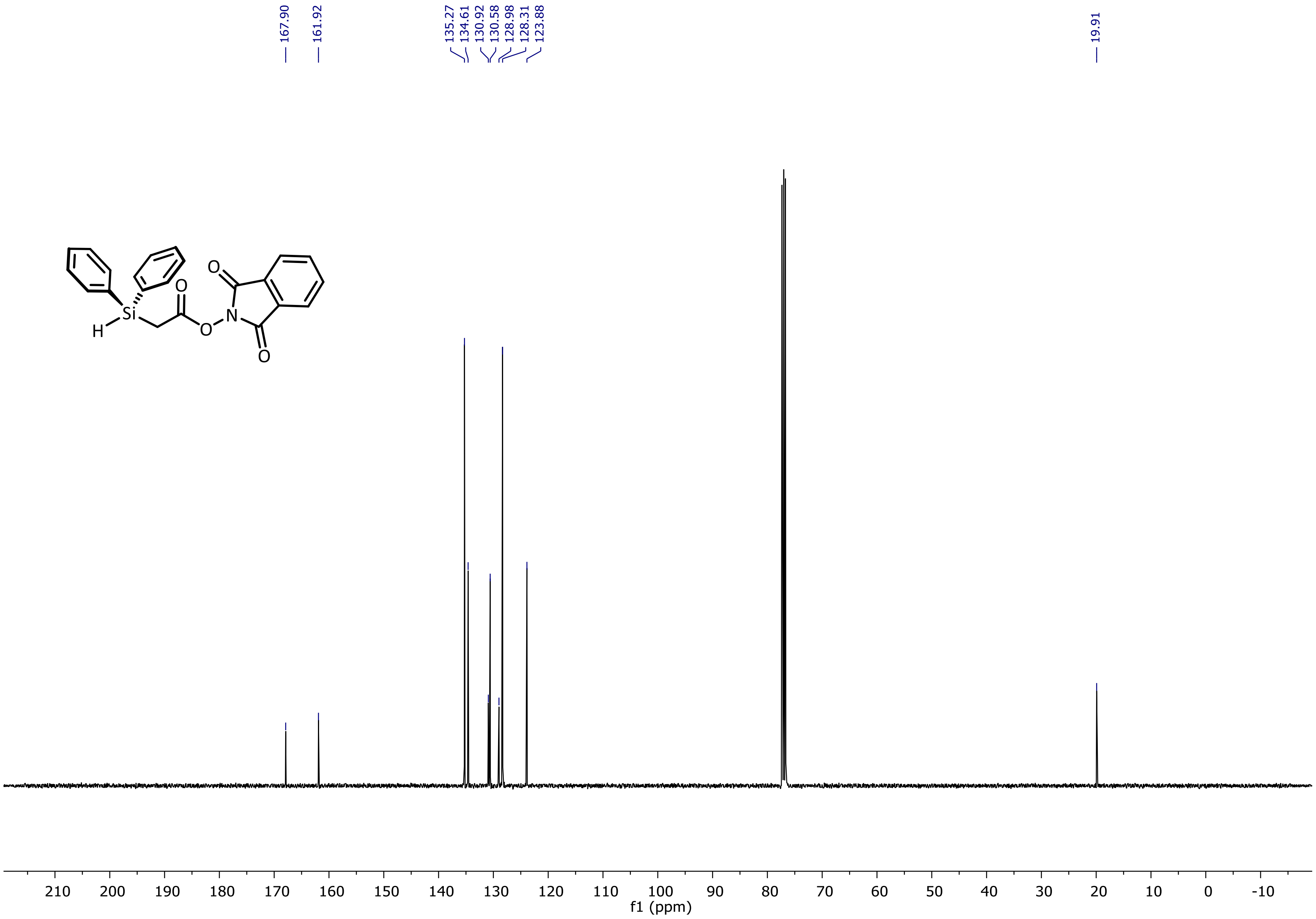

^{1}H-NMR (400 MHz, CDCl$_3$) for compound ***9o***

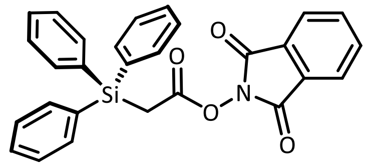

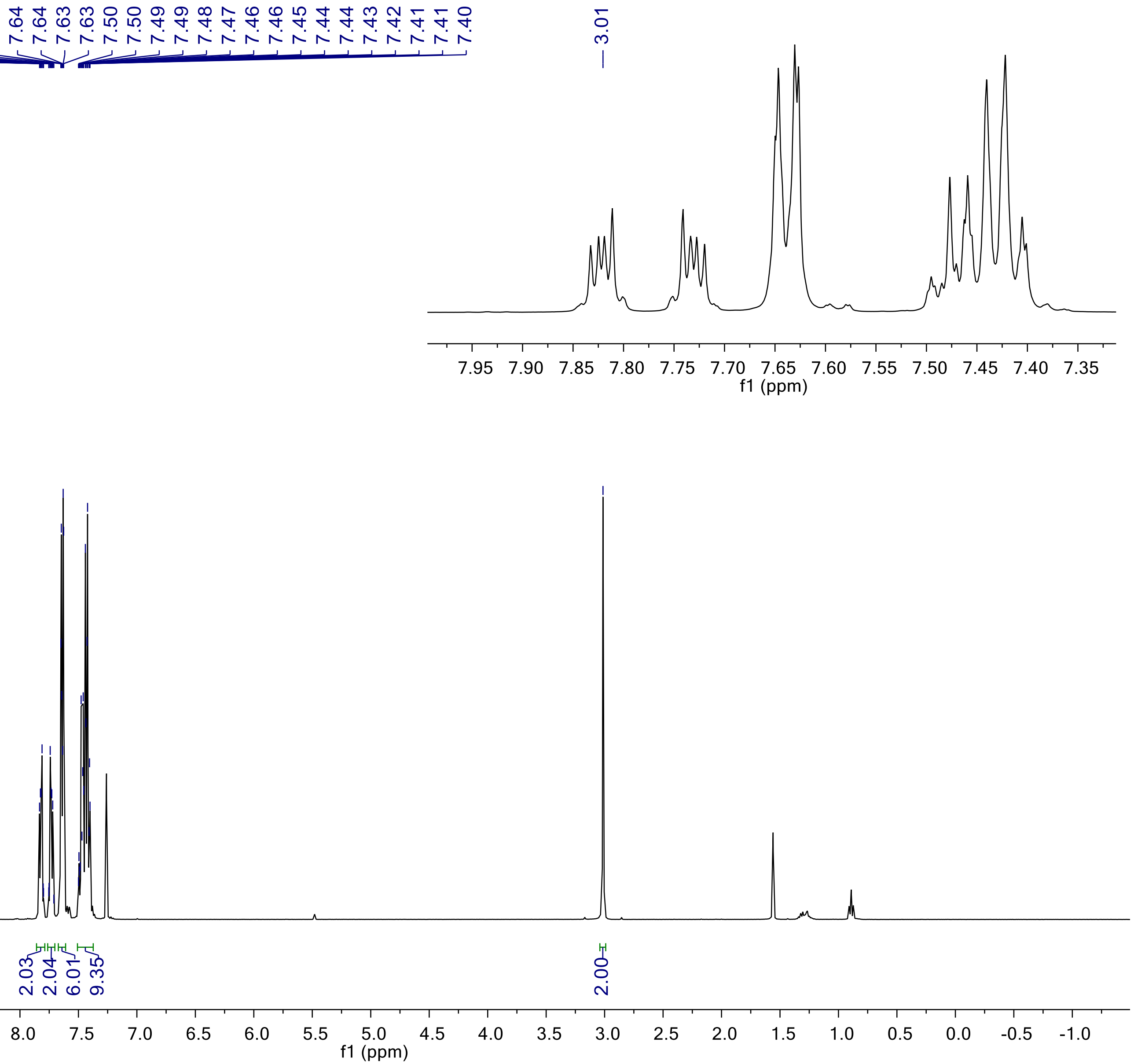

^{13}C-NMR (101 MHz, CDCl$_3$) for compound ***9o***

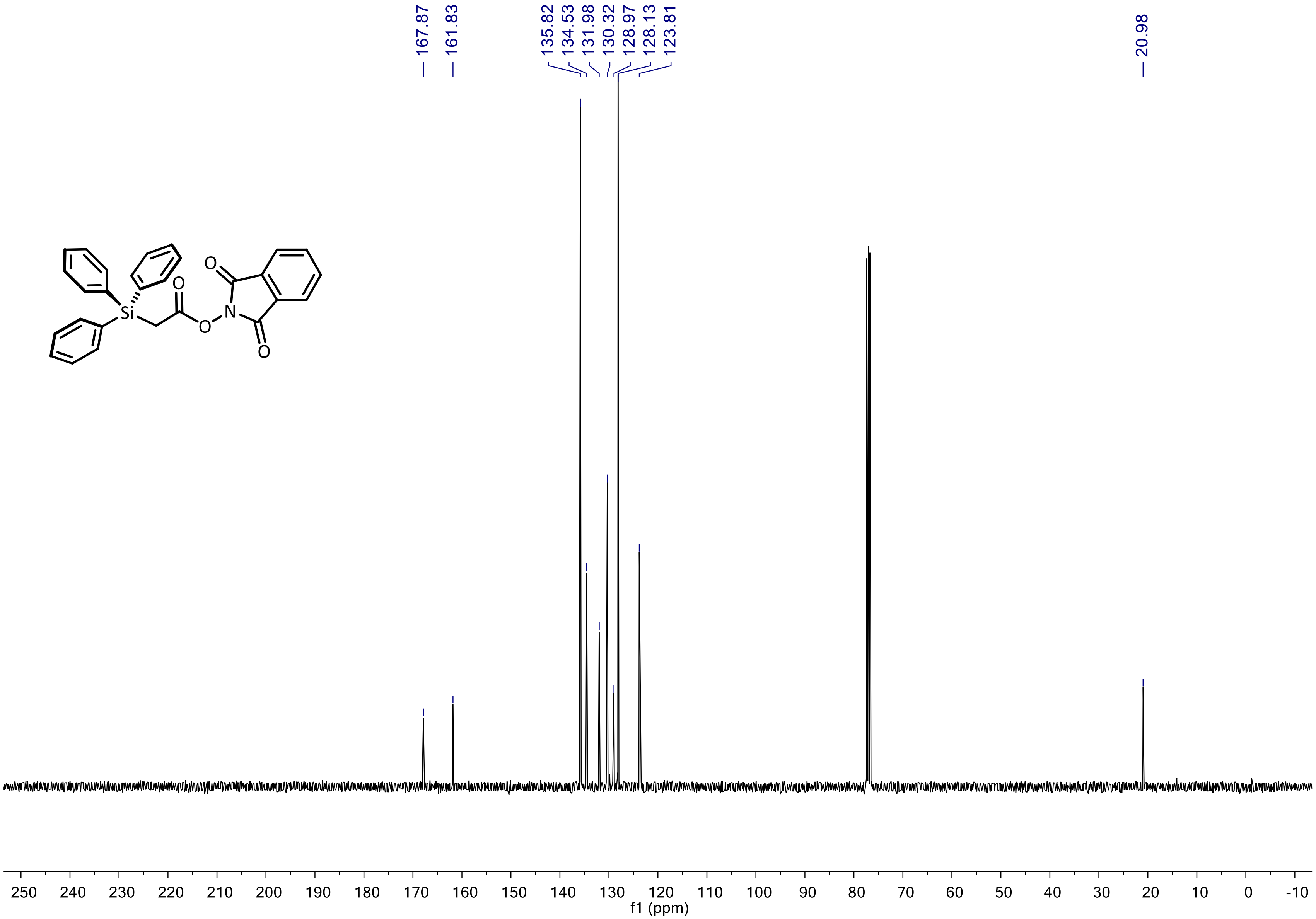

^{1}H-NMR (400 MHz, $CDCl_3$) for compound **9p**

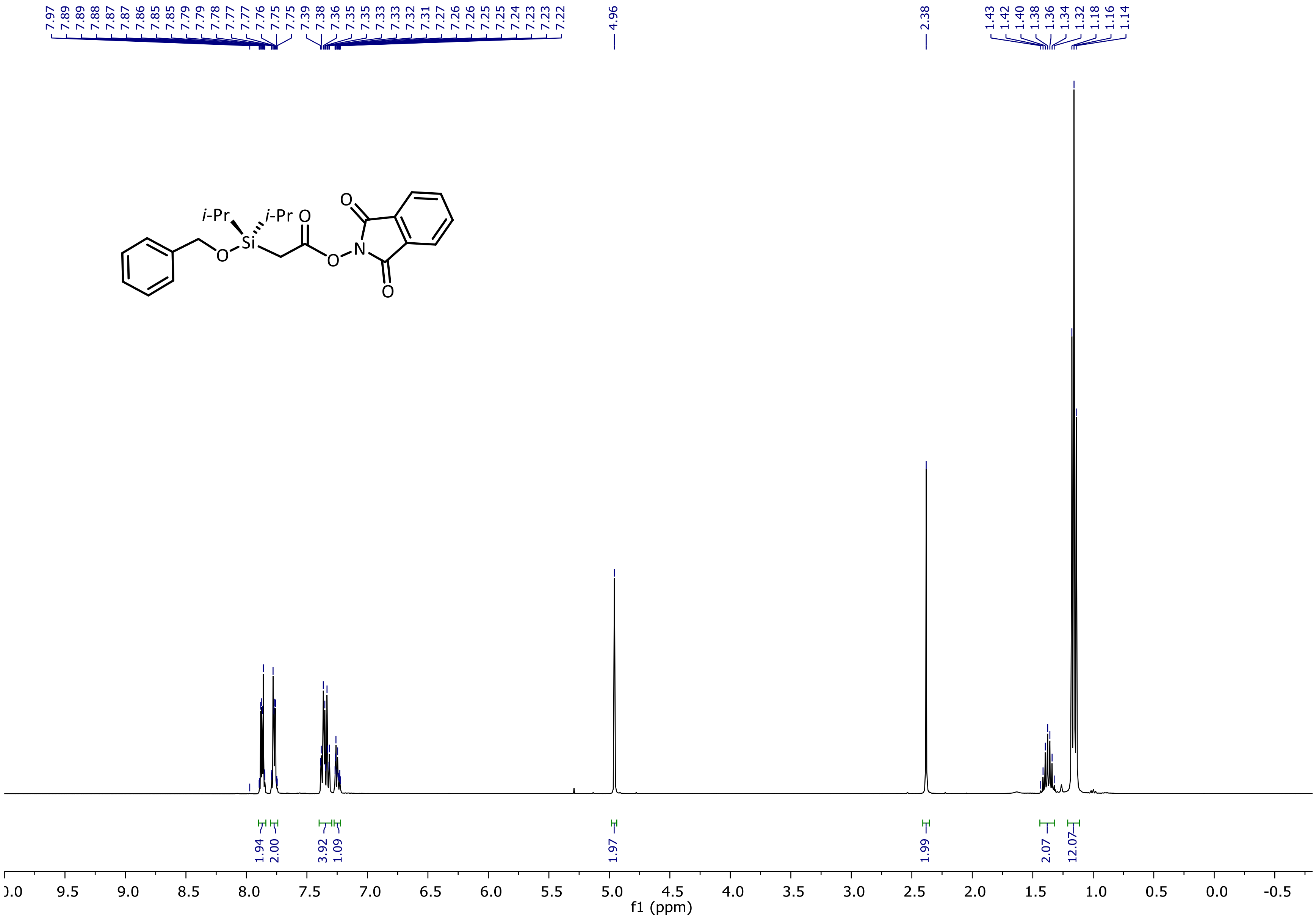

^{13}C-NMR (101 MHz, $CDCl_3$) for compound **9p**

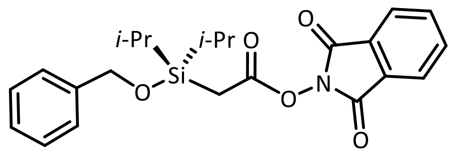

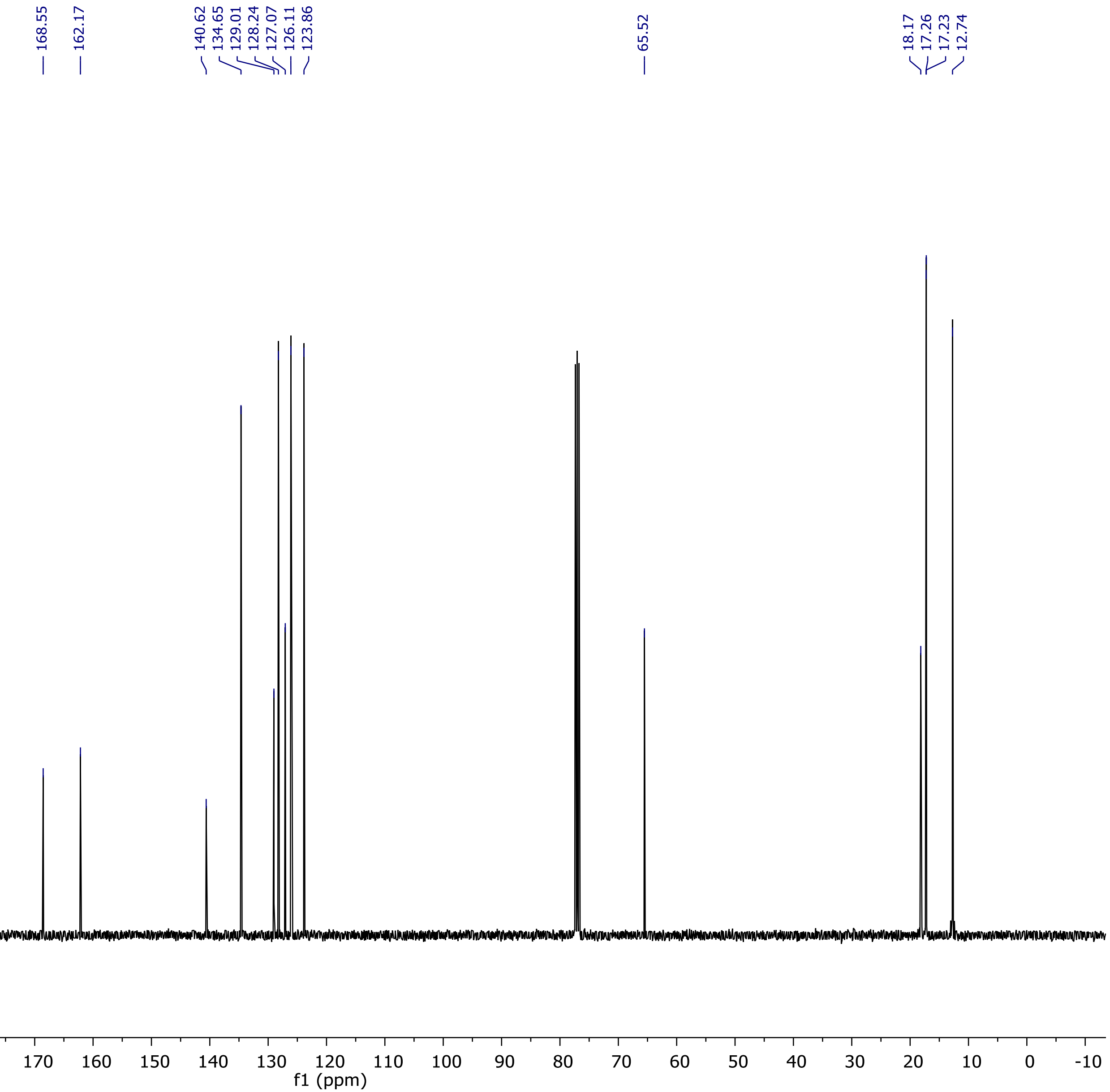

*^{1}H-NMR (400 MHz, $CDCl_3$) for compound **9q***

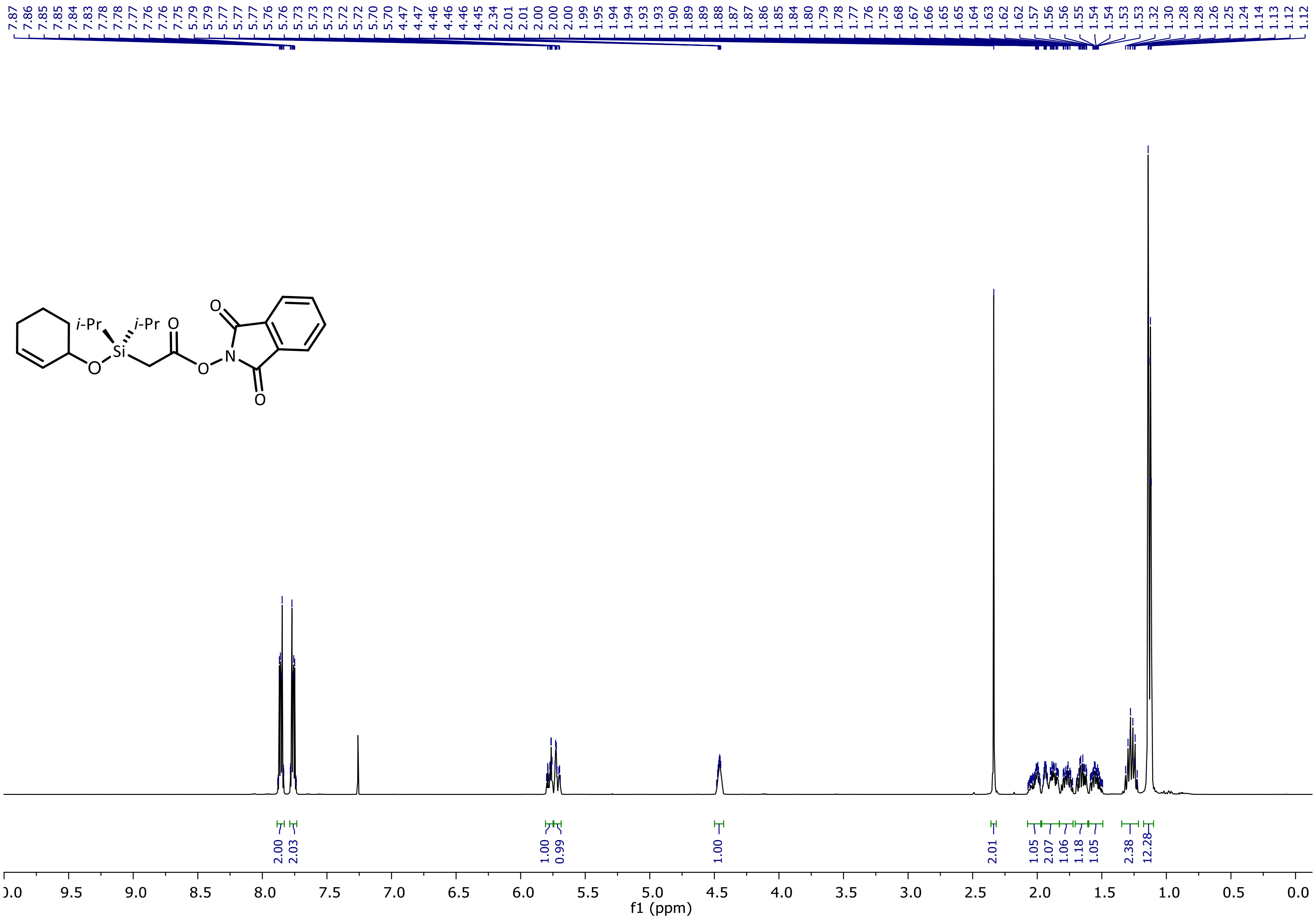

*^{13}C-NMR (101 MHz, $CDCl_3$) for compound **9q***

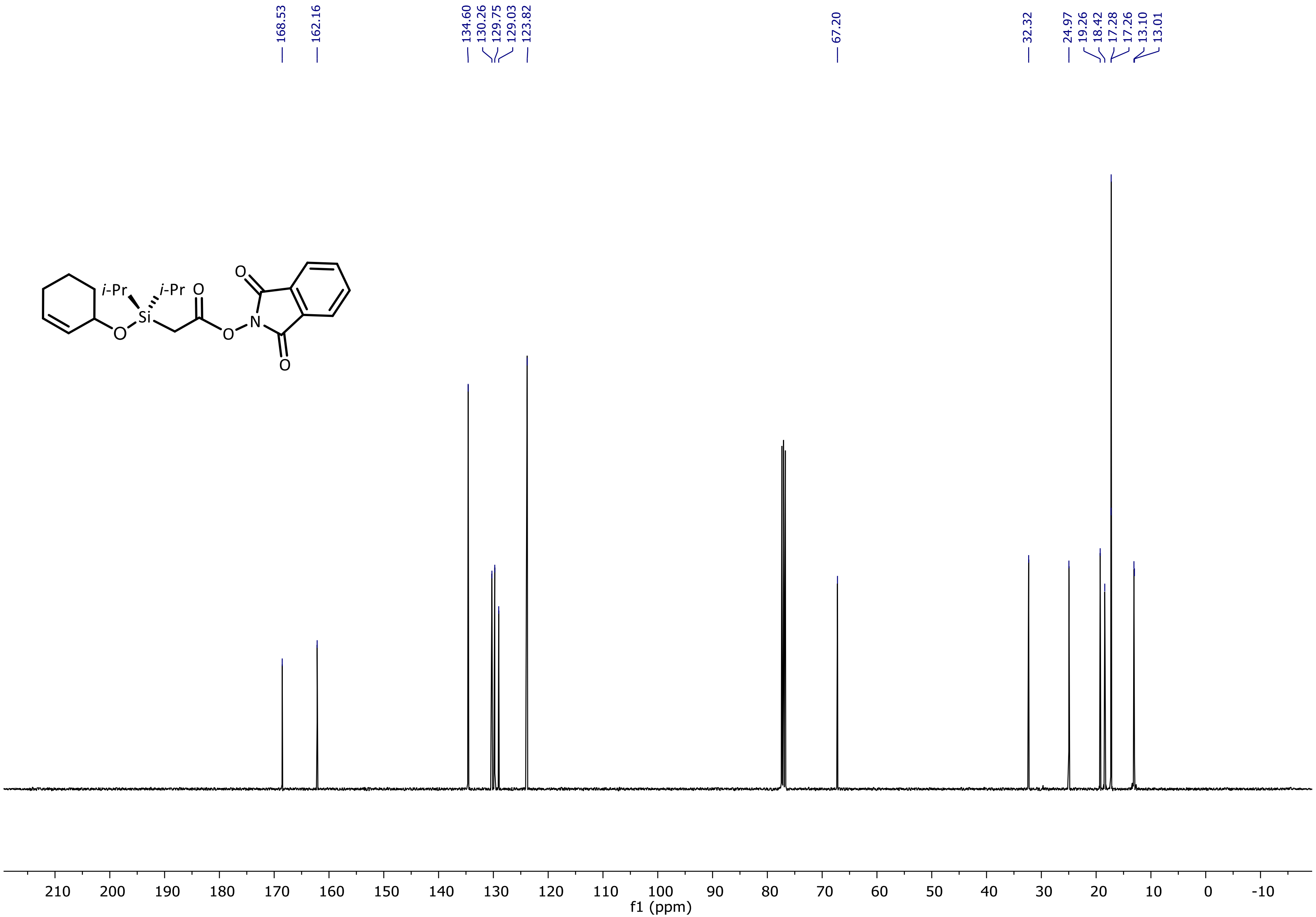

*^{1}H-NMR (400 MHz, $CDCl_3$) for compound **9r***

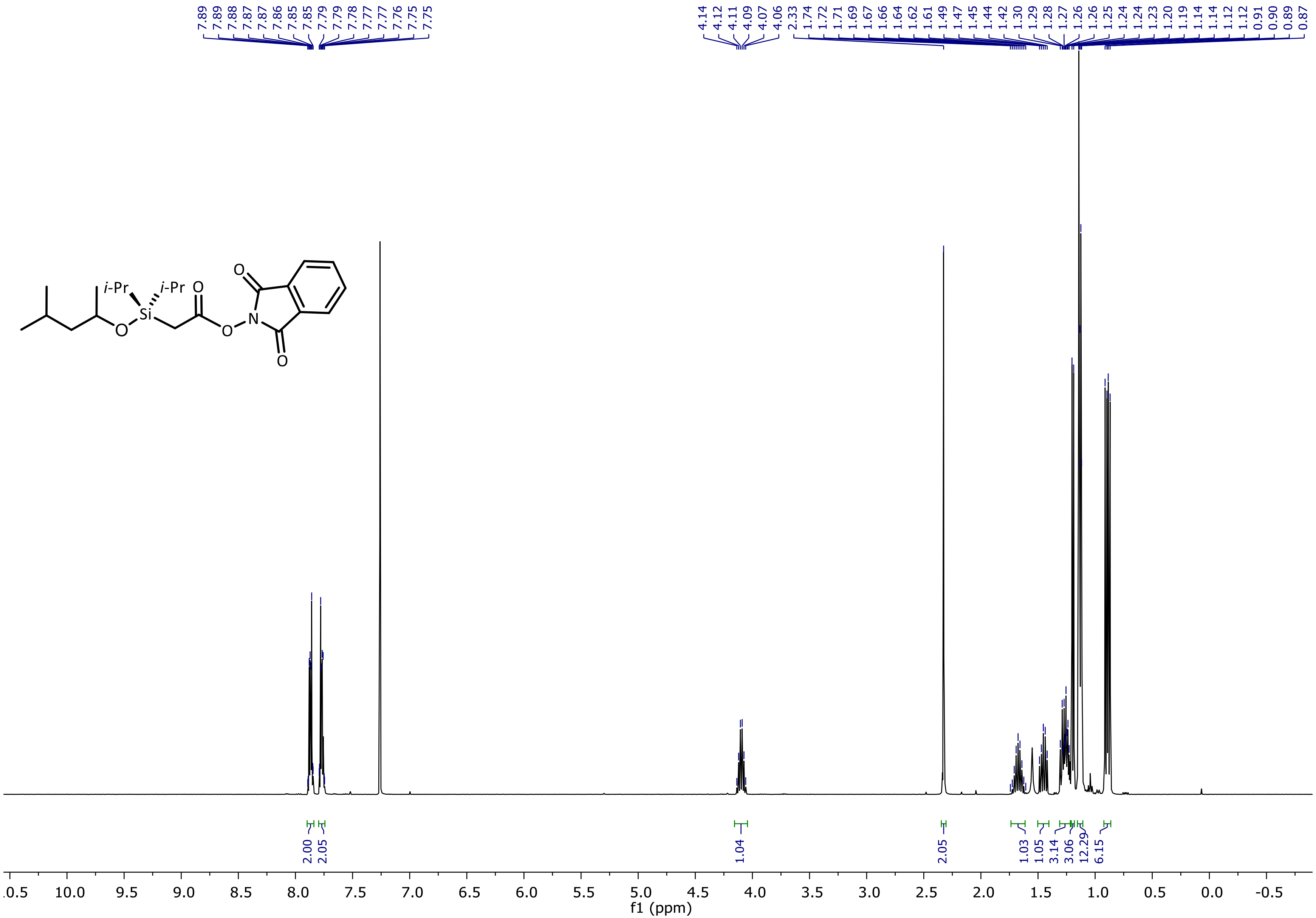

^{13}*C-NMR (101 MHz, $CDCl_3$) for compound* ***9r***

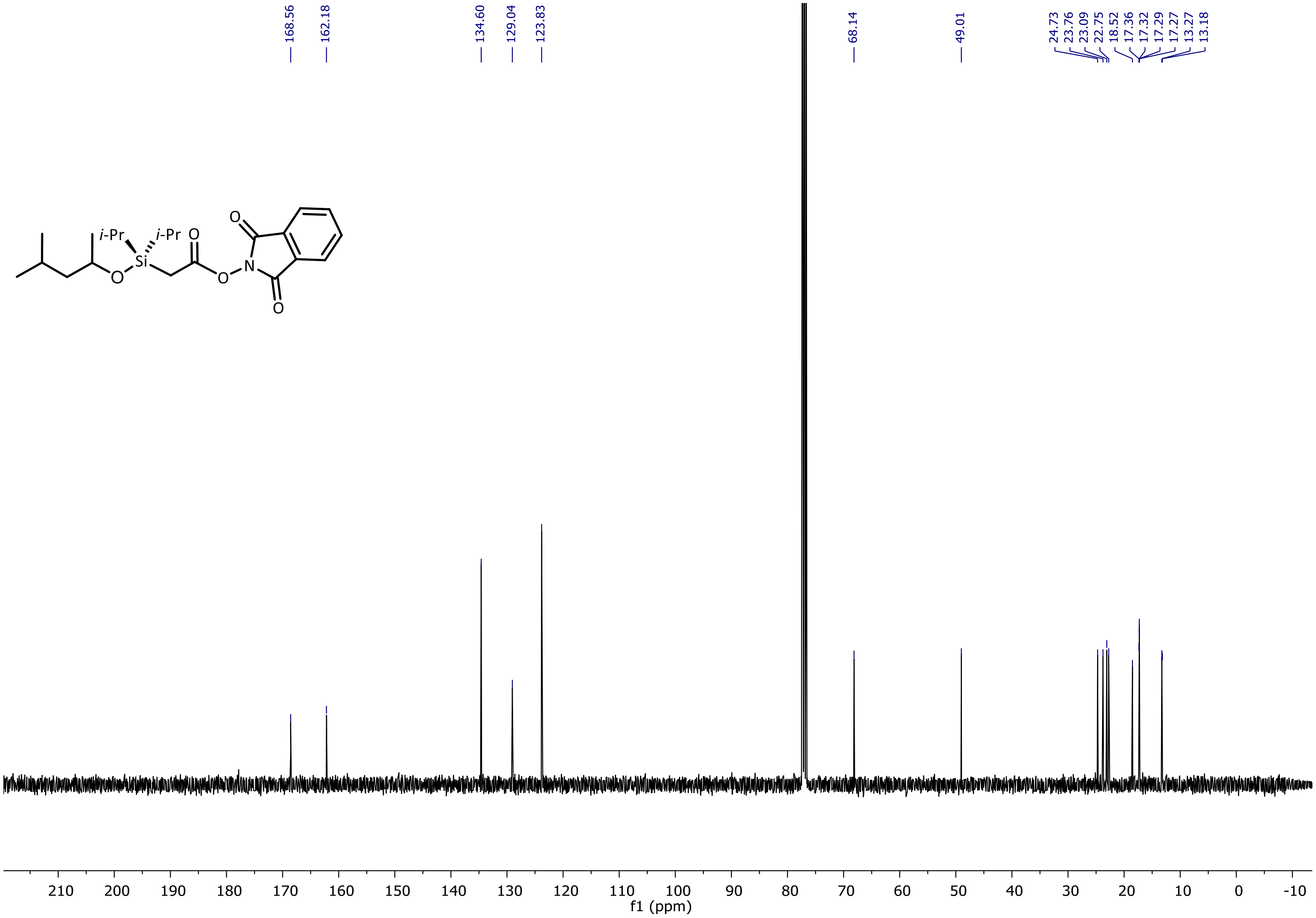

^{1}H-NMR (400 MHz, CDCl$_3$) for compound ***9s***

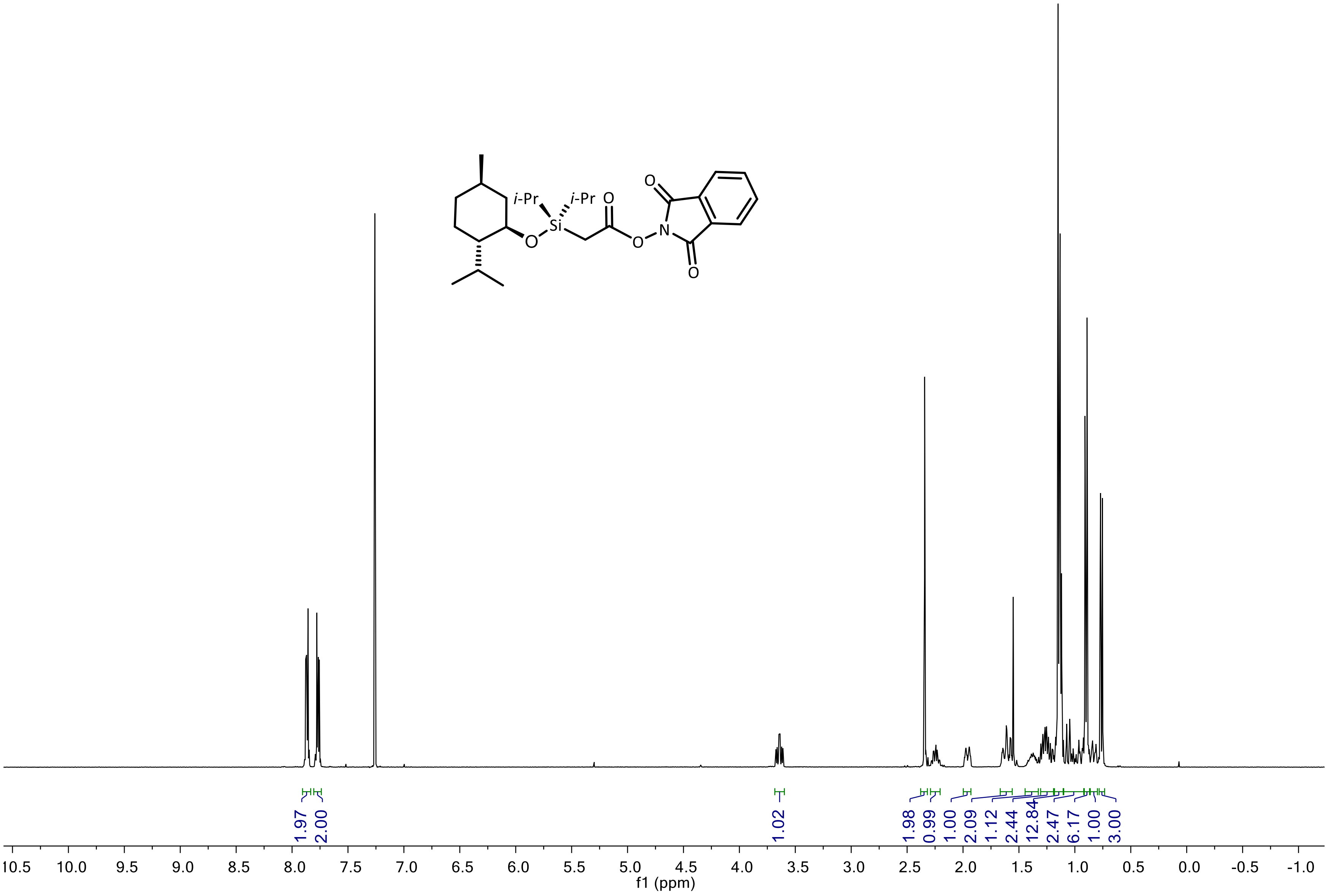

^{13}C-NMR (101 MHz, CDCl$_3$) for compound **9s**

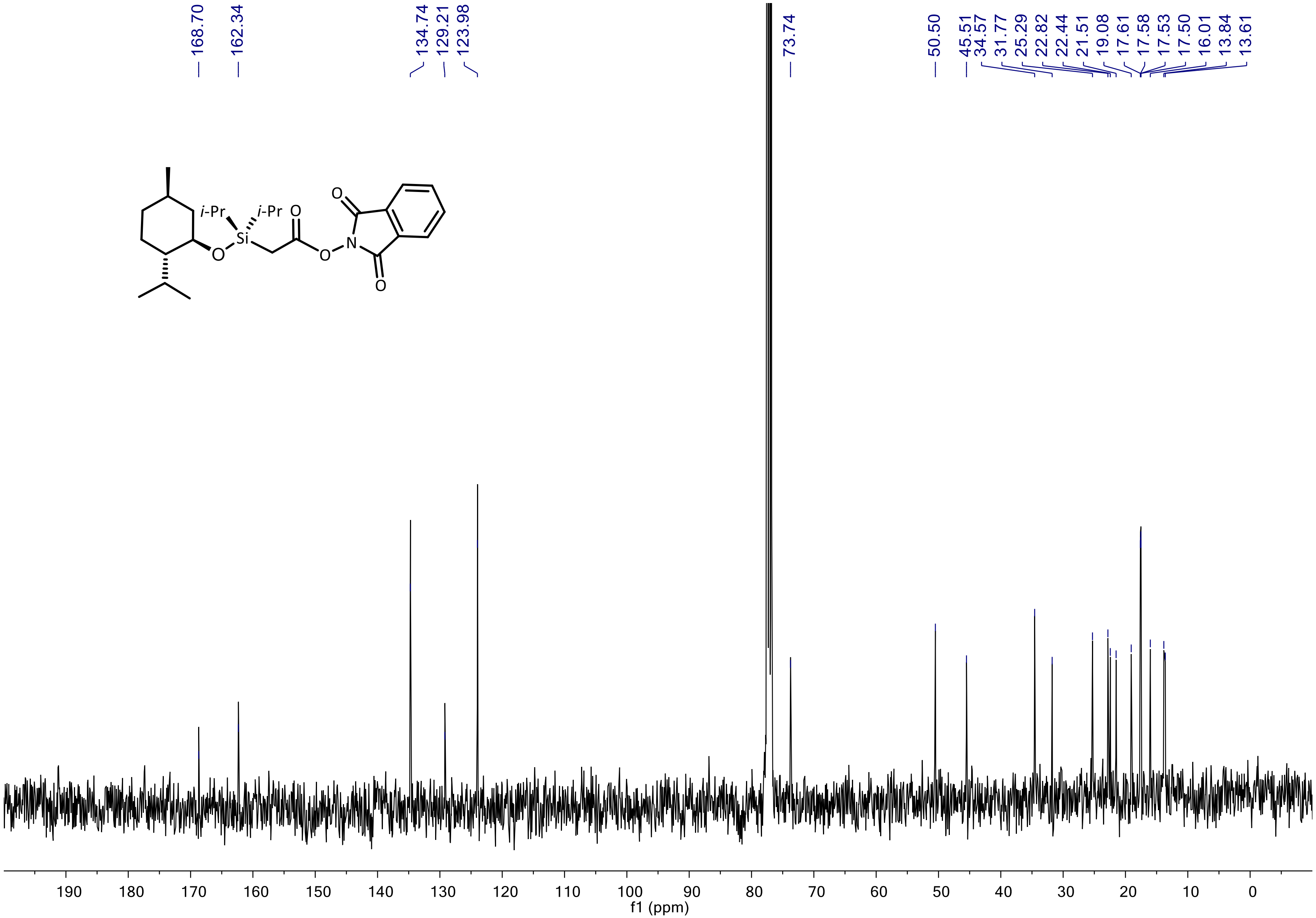

^{1}H-NMR (400 MHz, CDCl₃) for compound ***9t***

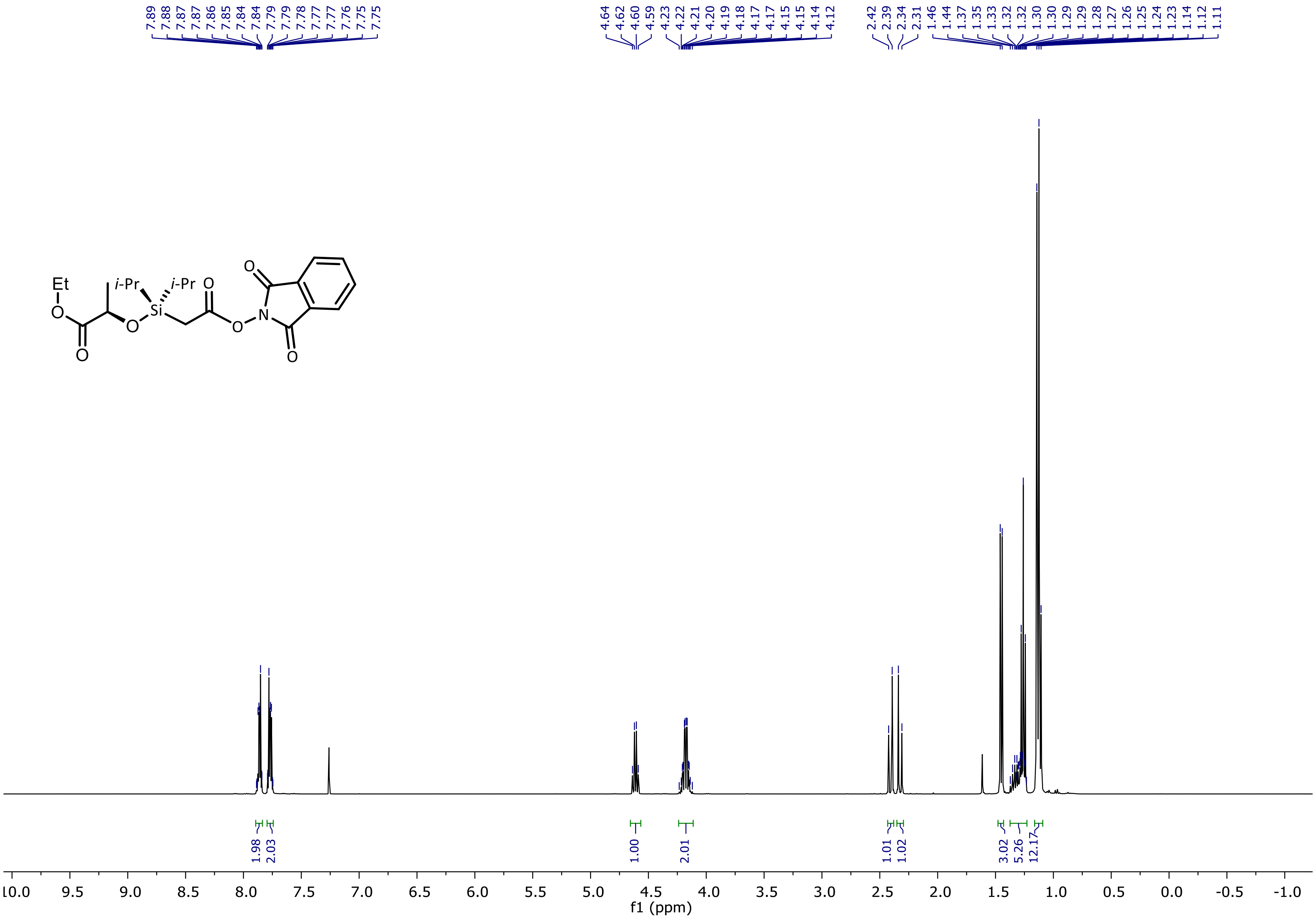

^{13}*C-NMR (101 MHz, CDCl$_3$) for compound* ***9t***

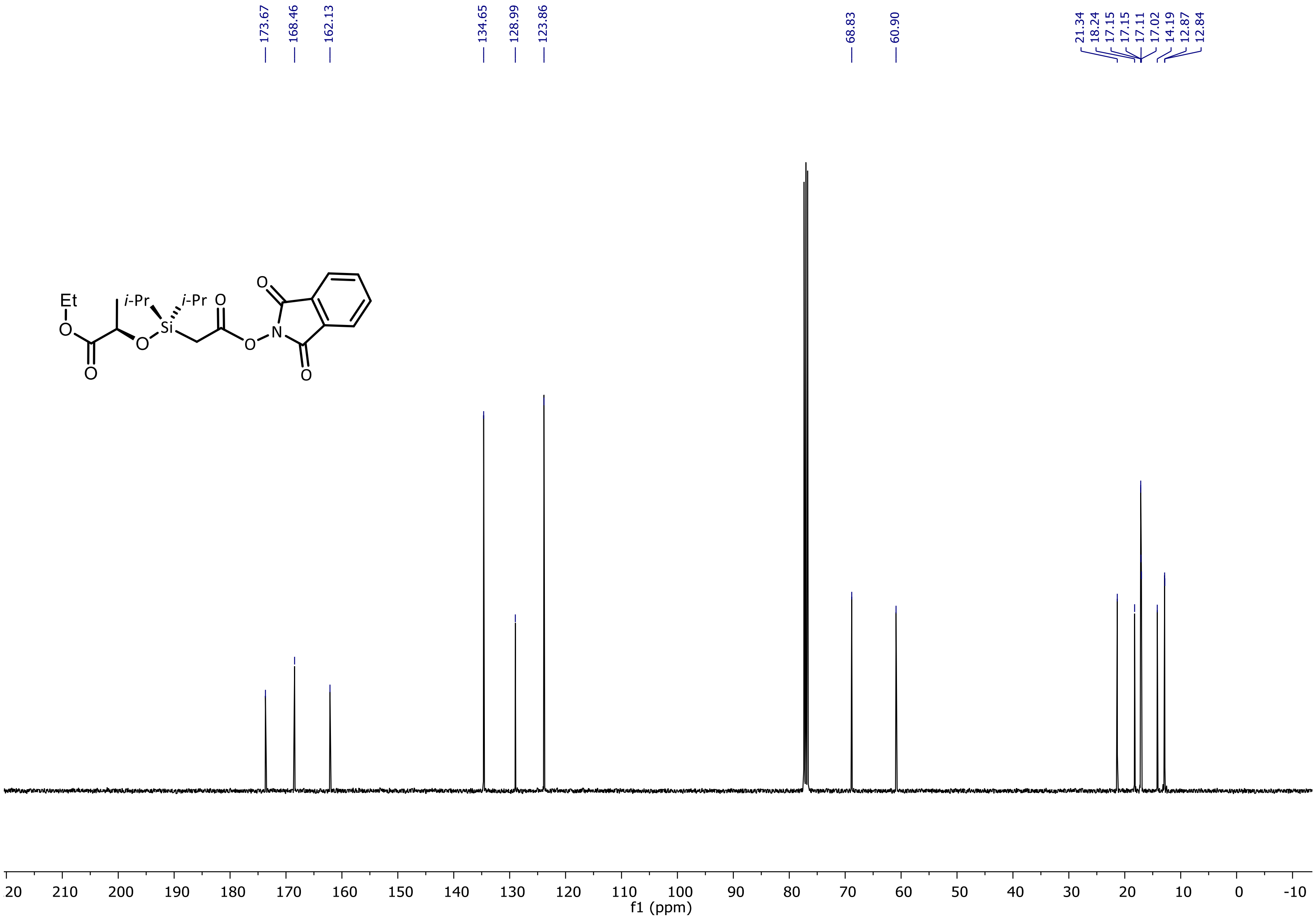

*^{1}H-NMR (400 MHz, $CDCl_3$) for compound **9u***

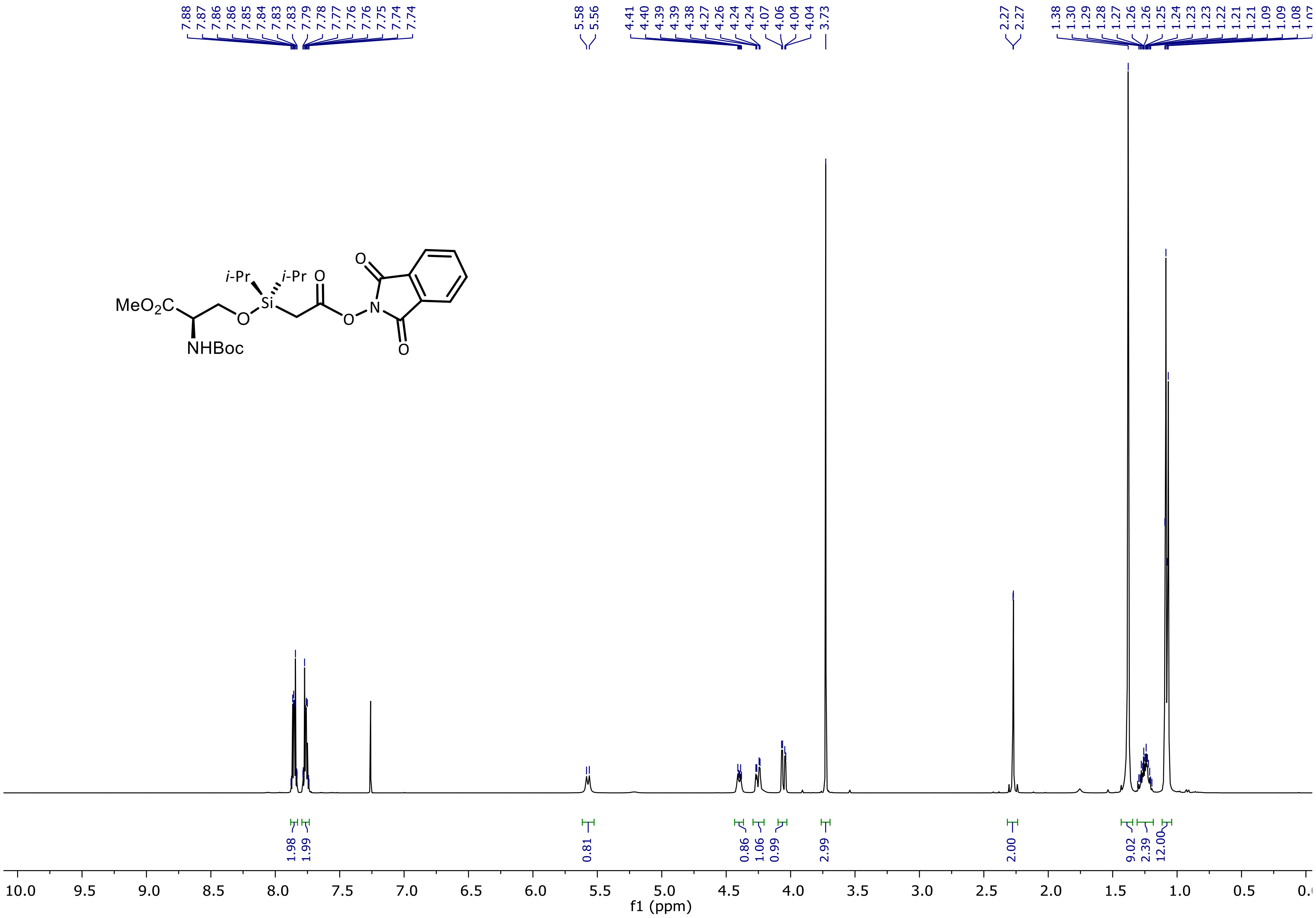

*^{13}C-NMR (101 MHz, $CDCl_3$) for compound **9u***

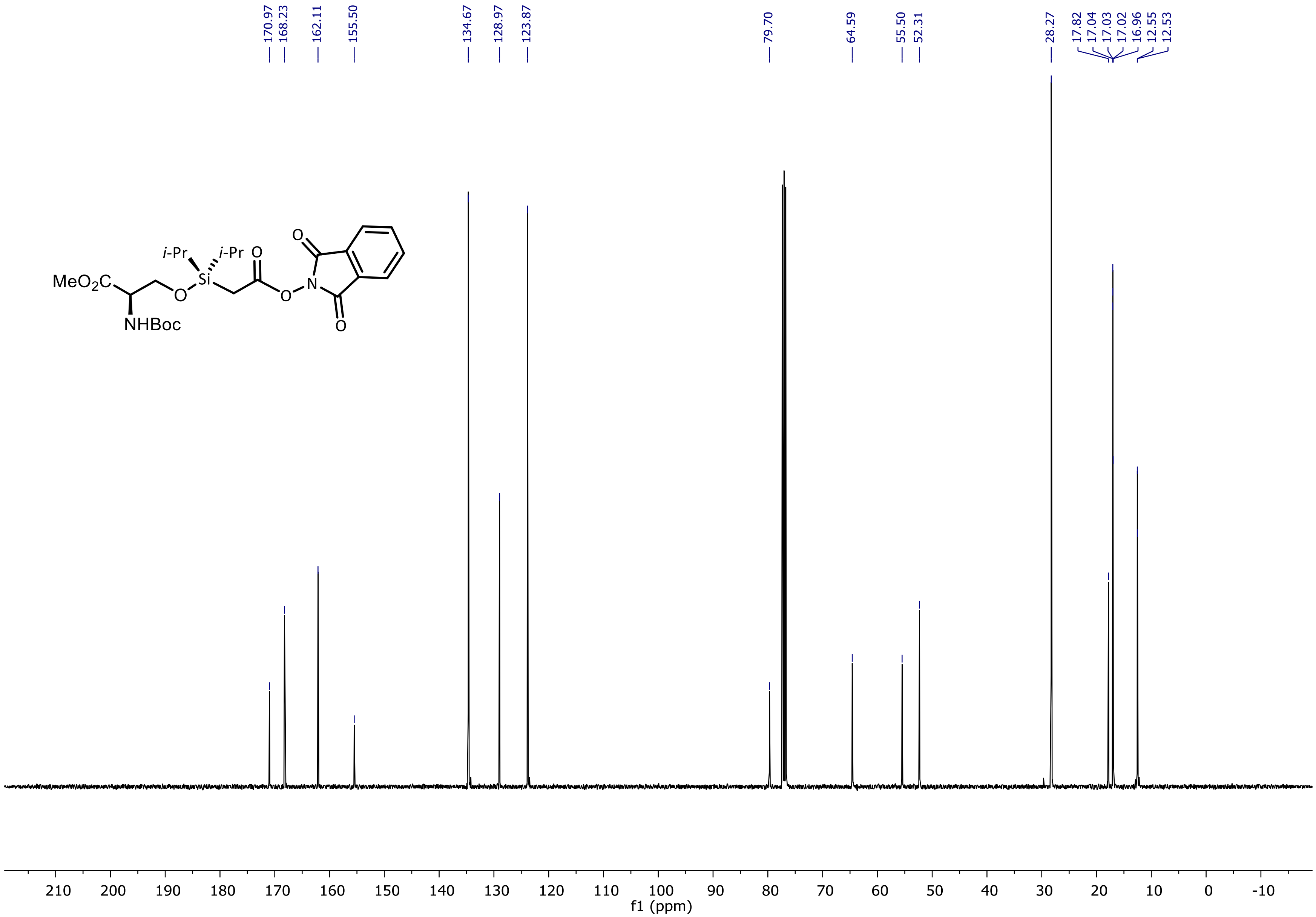

^{1}H-NMR (400 MHz, $CDCl_3$) for compound **9v**

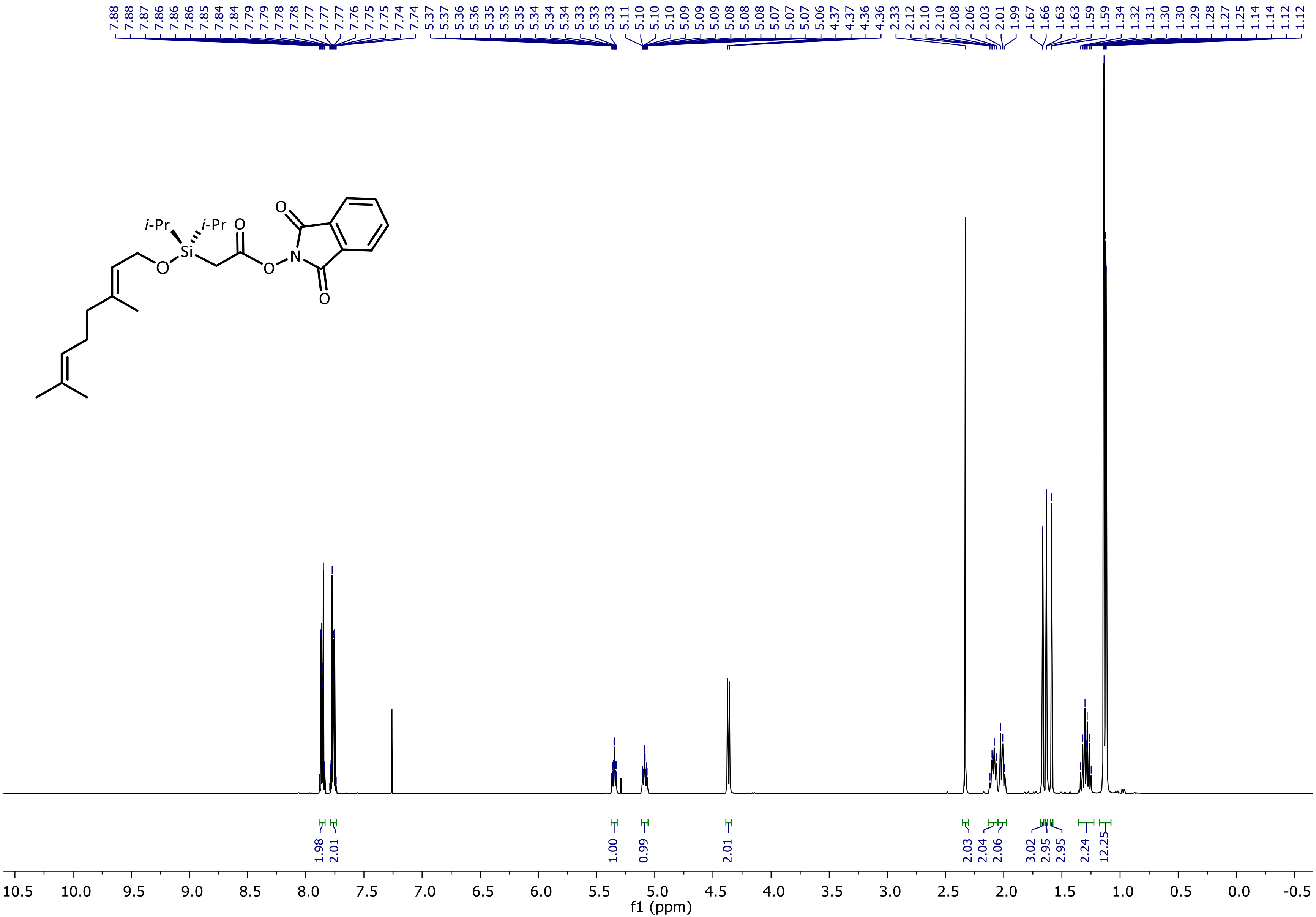

*^{13}C-NMR (101 MHz, CDCl₃) for compound **9v***

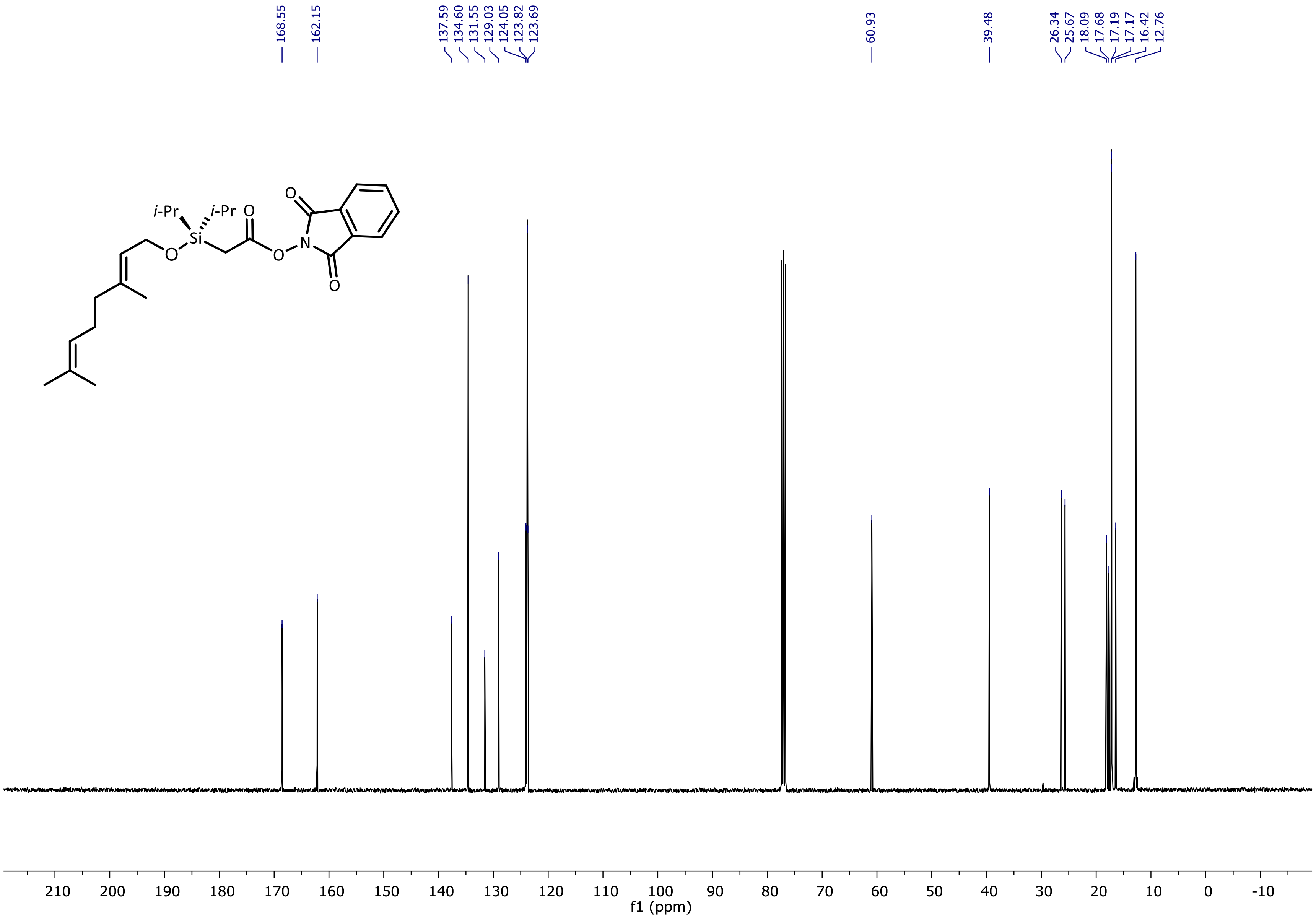

^{1}H-NMR (400 MHz, $CDCl_3$) for compound **9w**

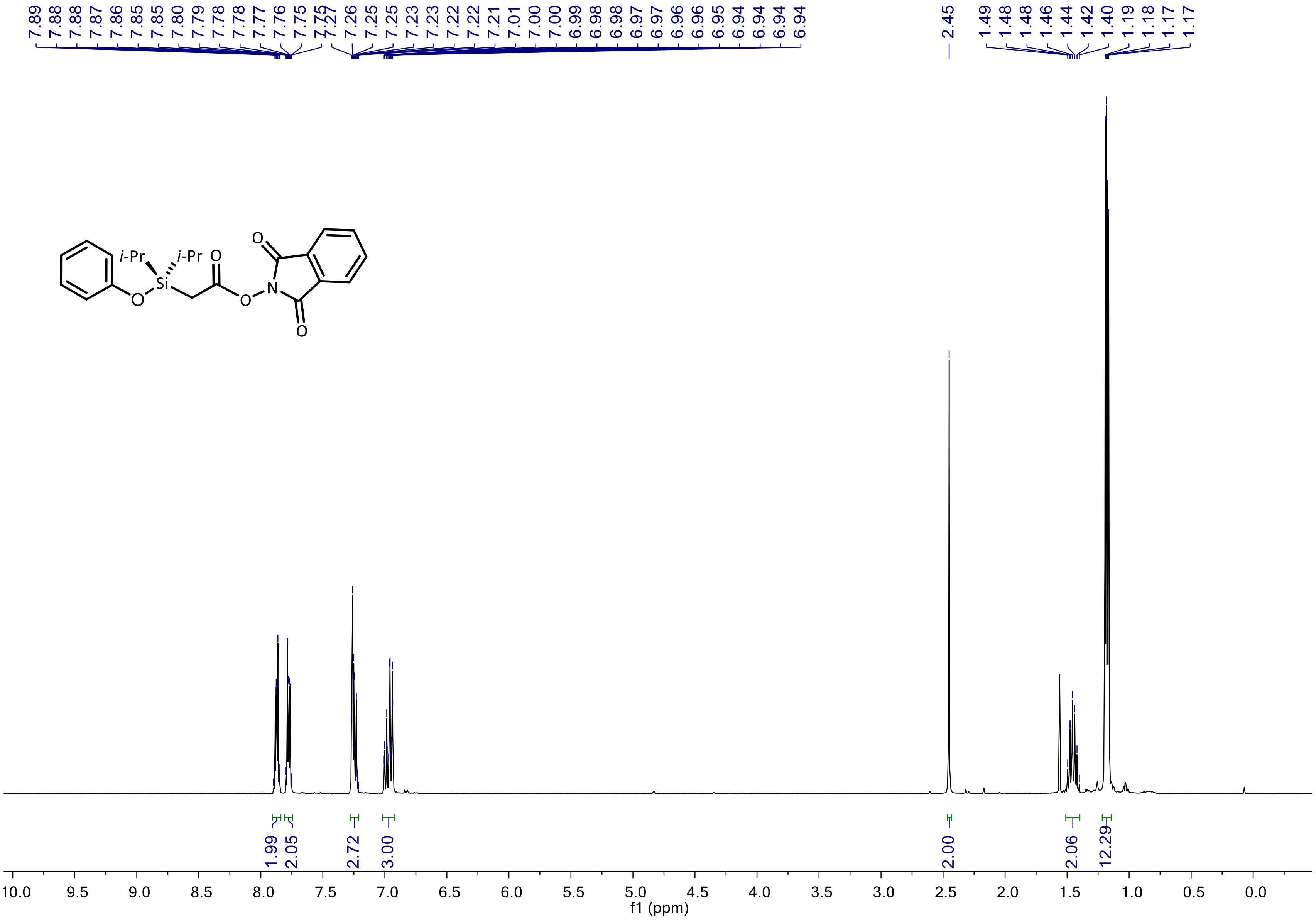

^{13}C-NMR (101 MHz, CDCl₃) for compound ***9w***

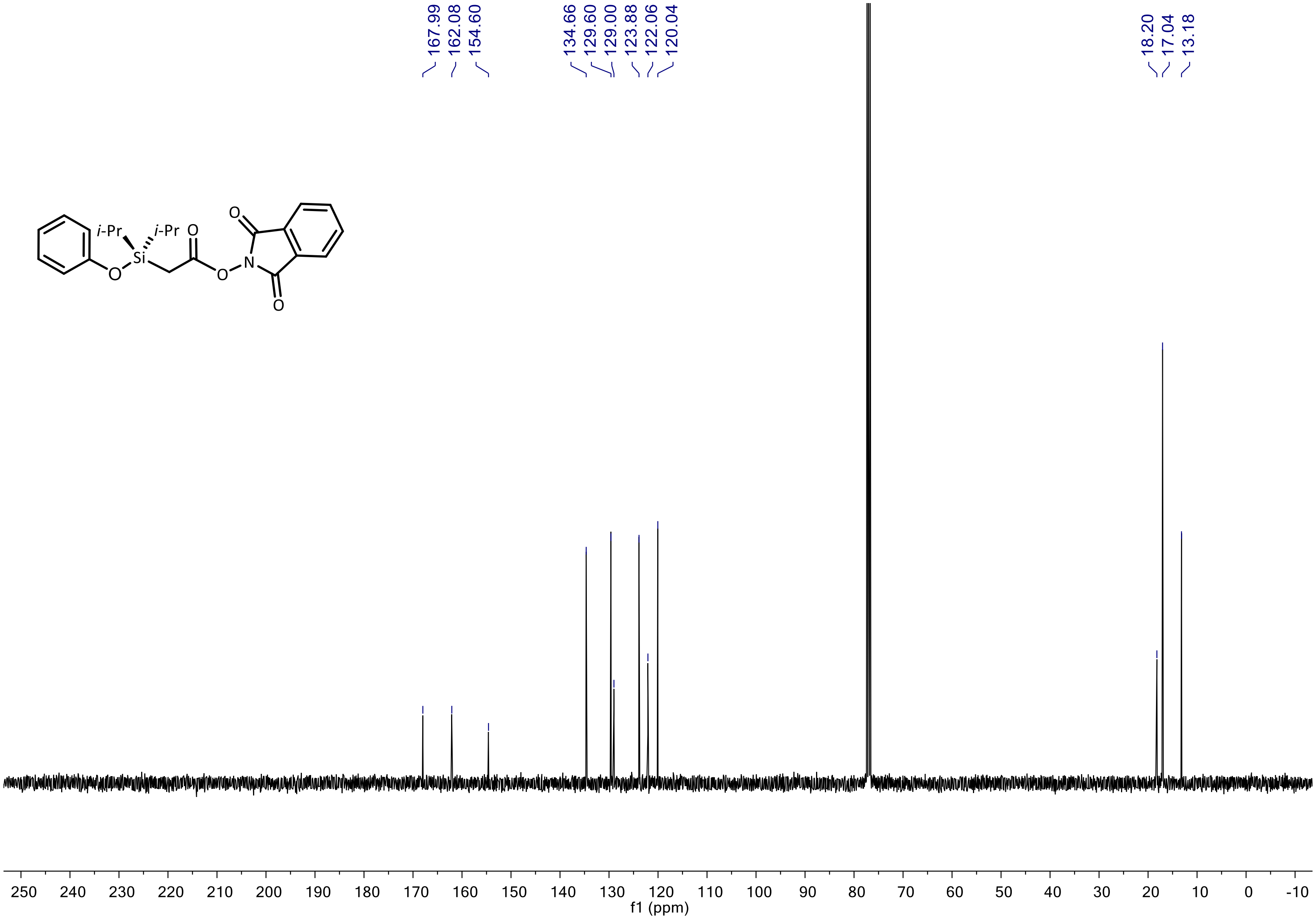

*^{1}H-NMR (400 MHz, CDCl$_3$) for compound **9x***

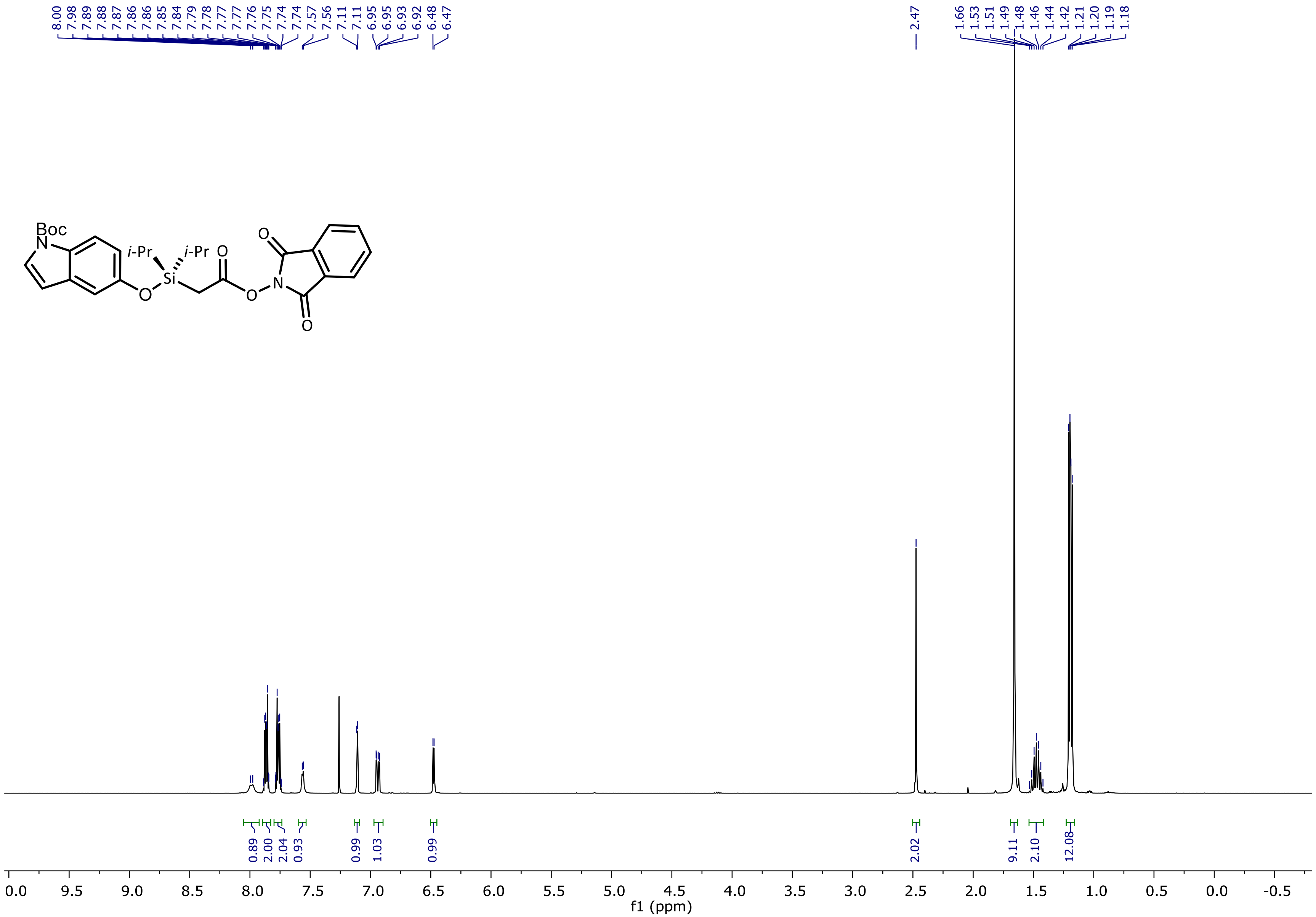

*^{13}C-NMR (101 MHz, $CDCl_3$) for compound **9x***

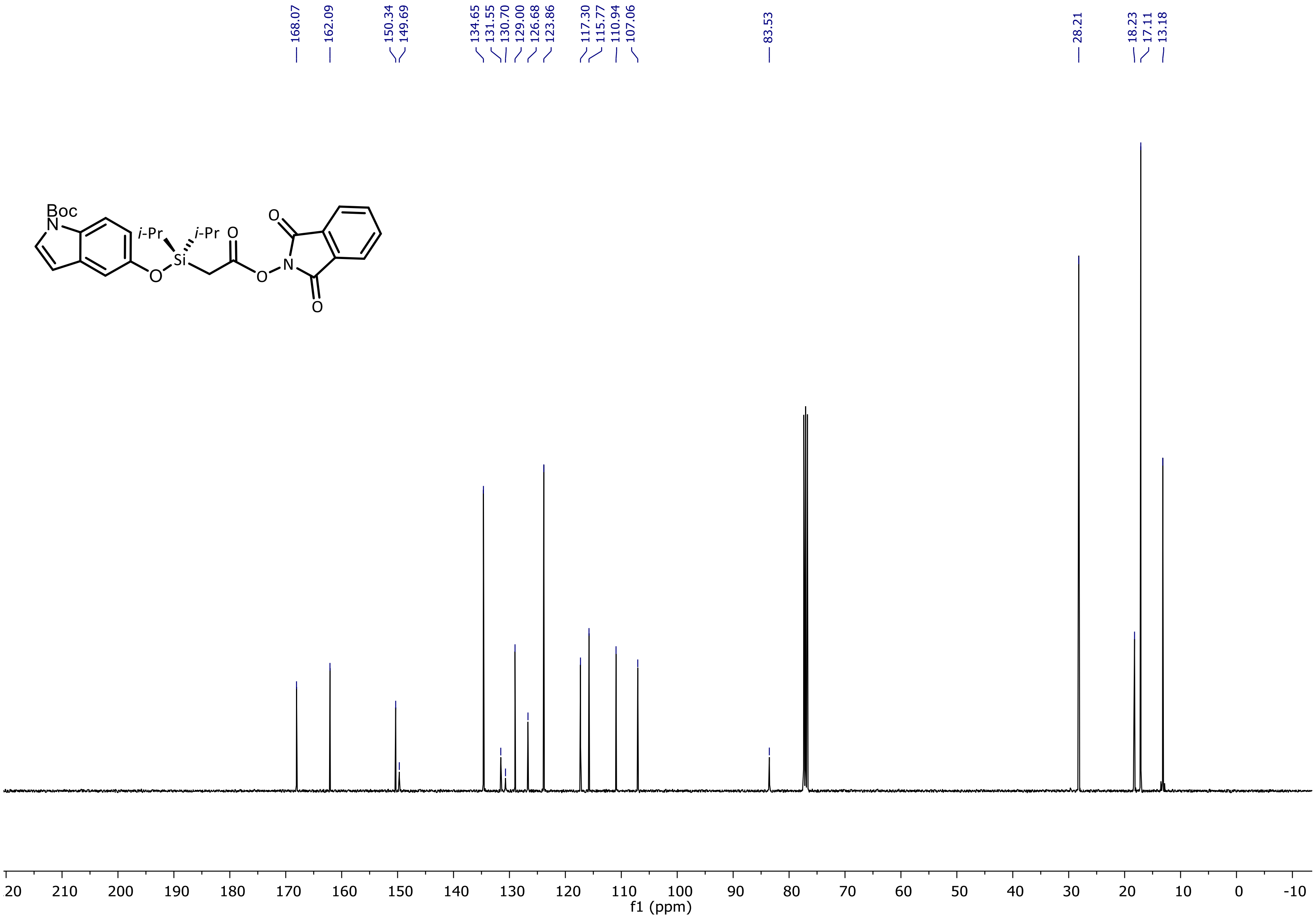

*^{1}H-NMR (400 MHz, $CDCl_3$) for compound **9y***

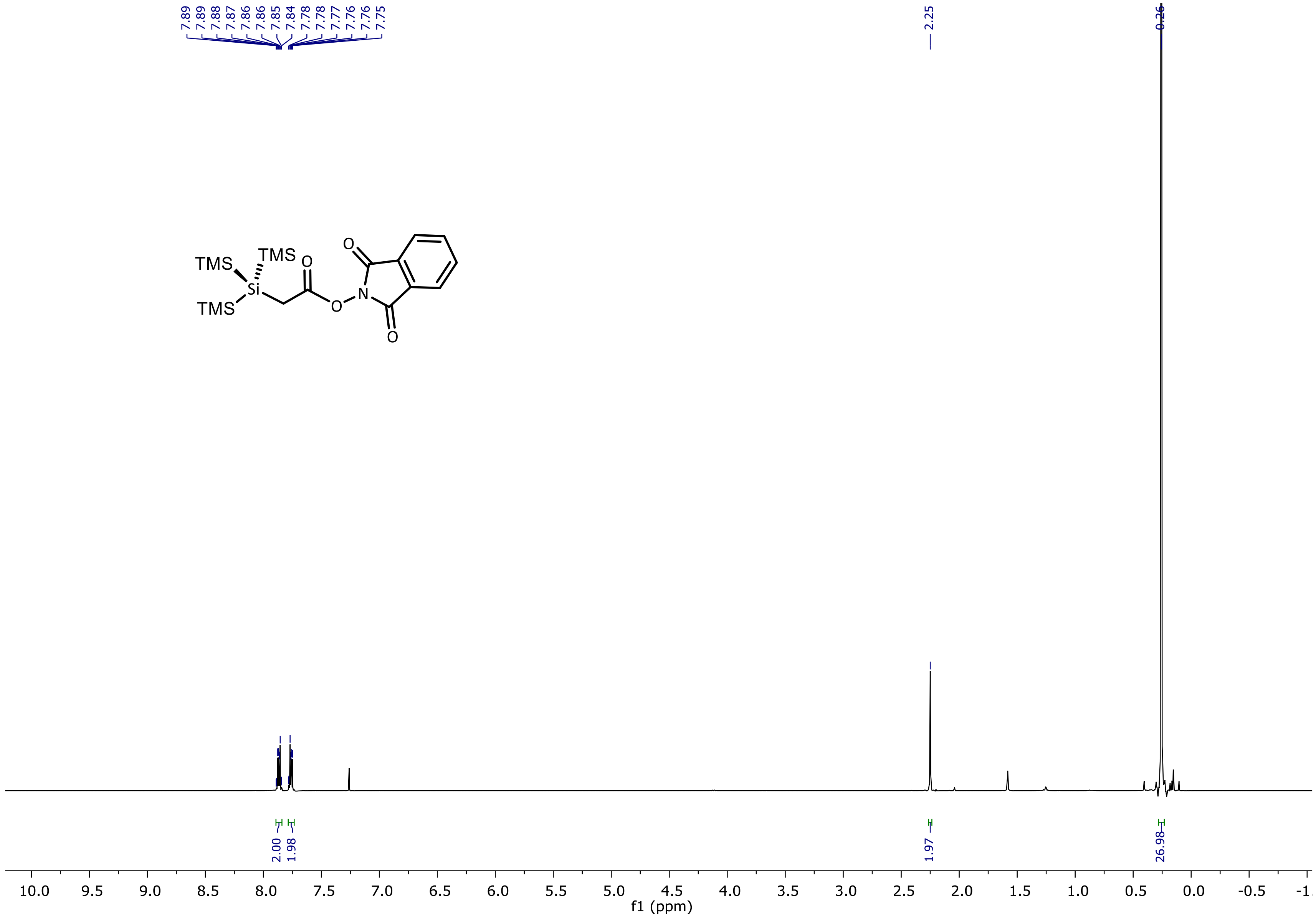

^{13}C-NMR (101 MHz, $CDCl_3$) for compound **9y**

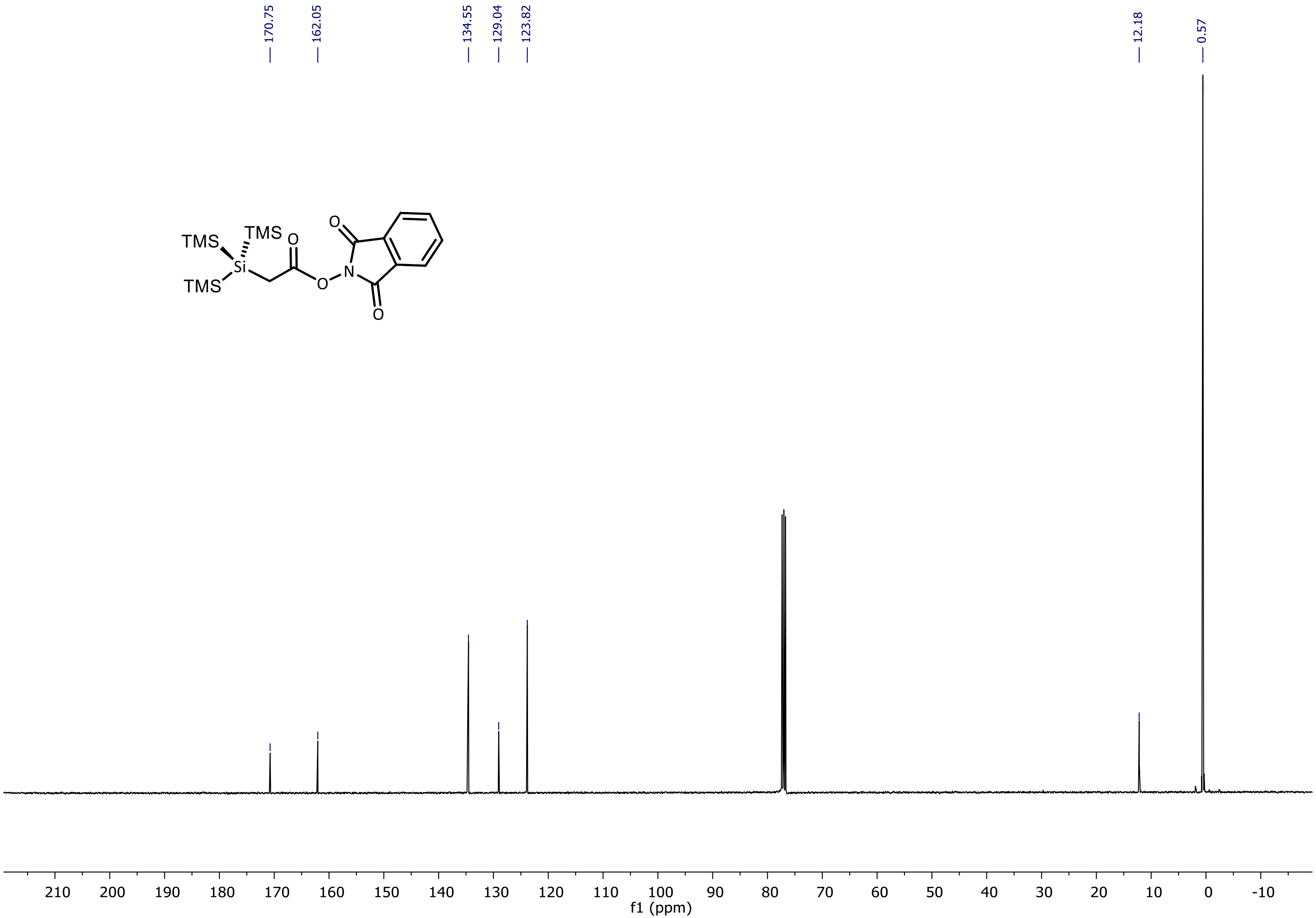

^{1}H-NMR (400 MHz, $CDCl_3$) for compound ***9z***

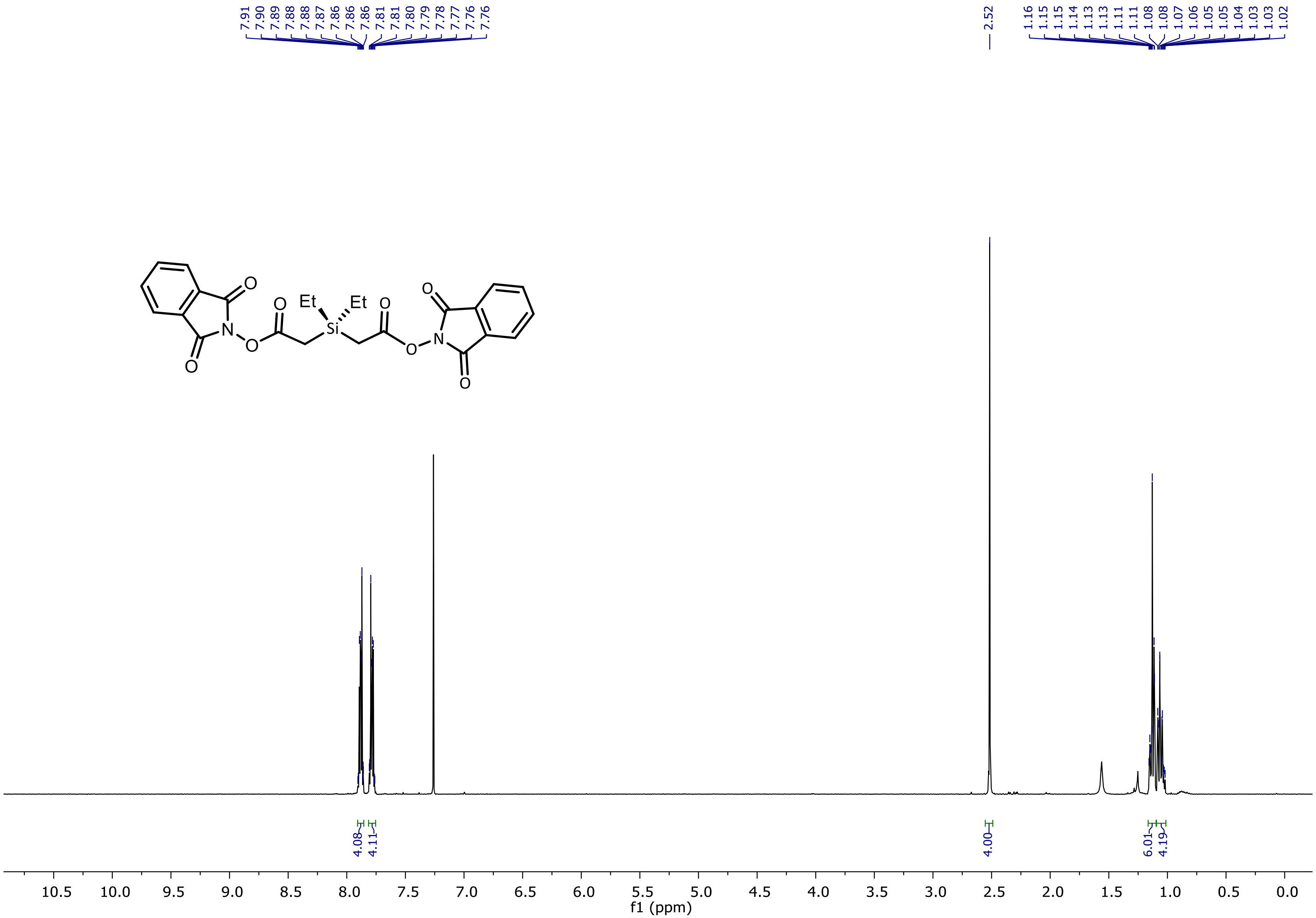

*^{13}C-NMR (101 MHz, CDCl₃) for compound **9z***

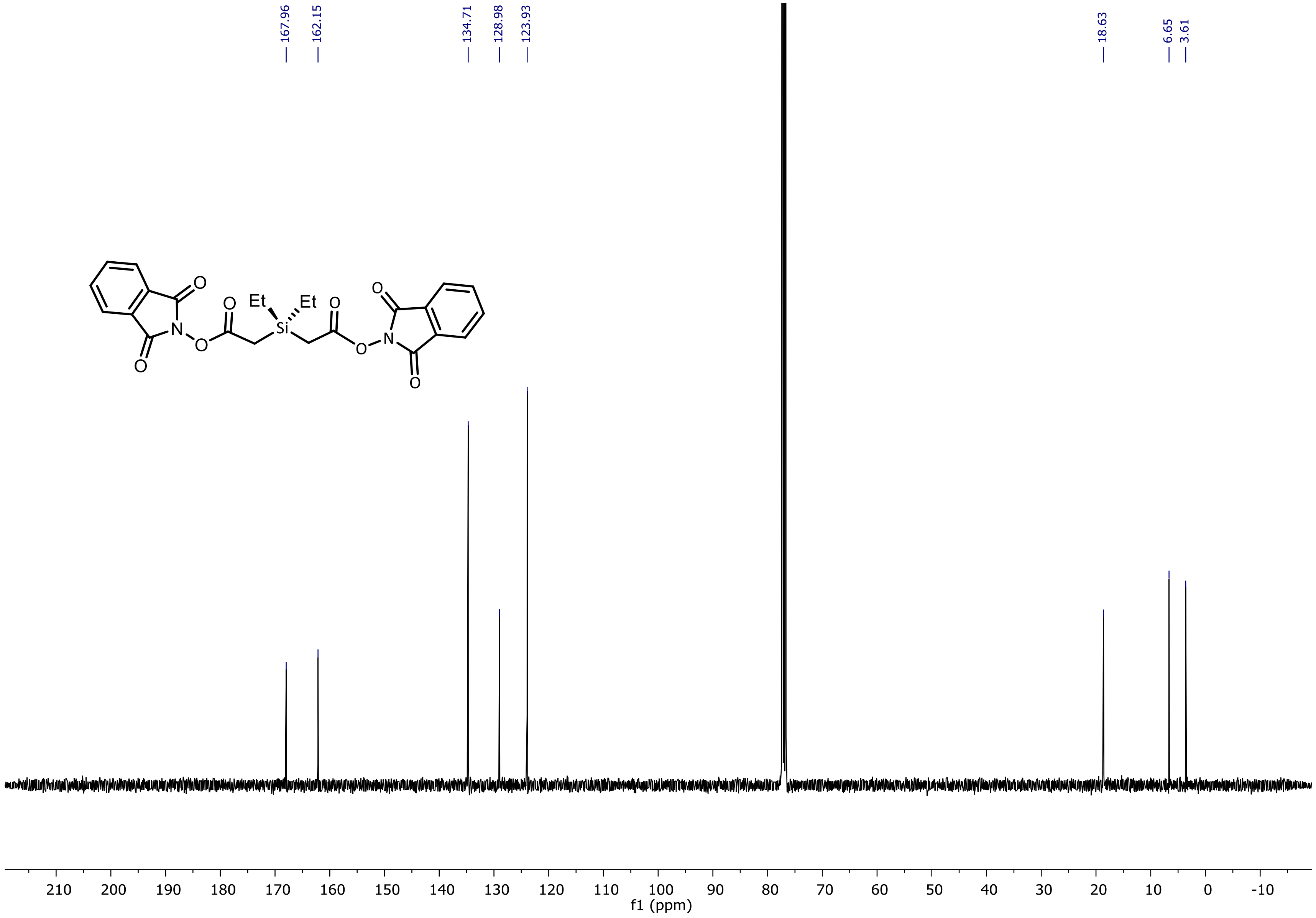

*^{1}H-NMR (400 MHz, $CDCl_3$) for compound **9aa***

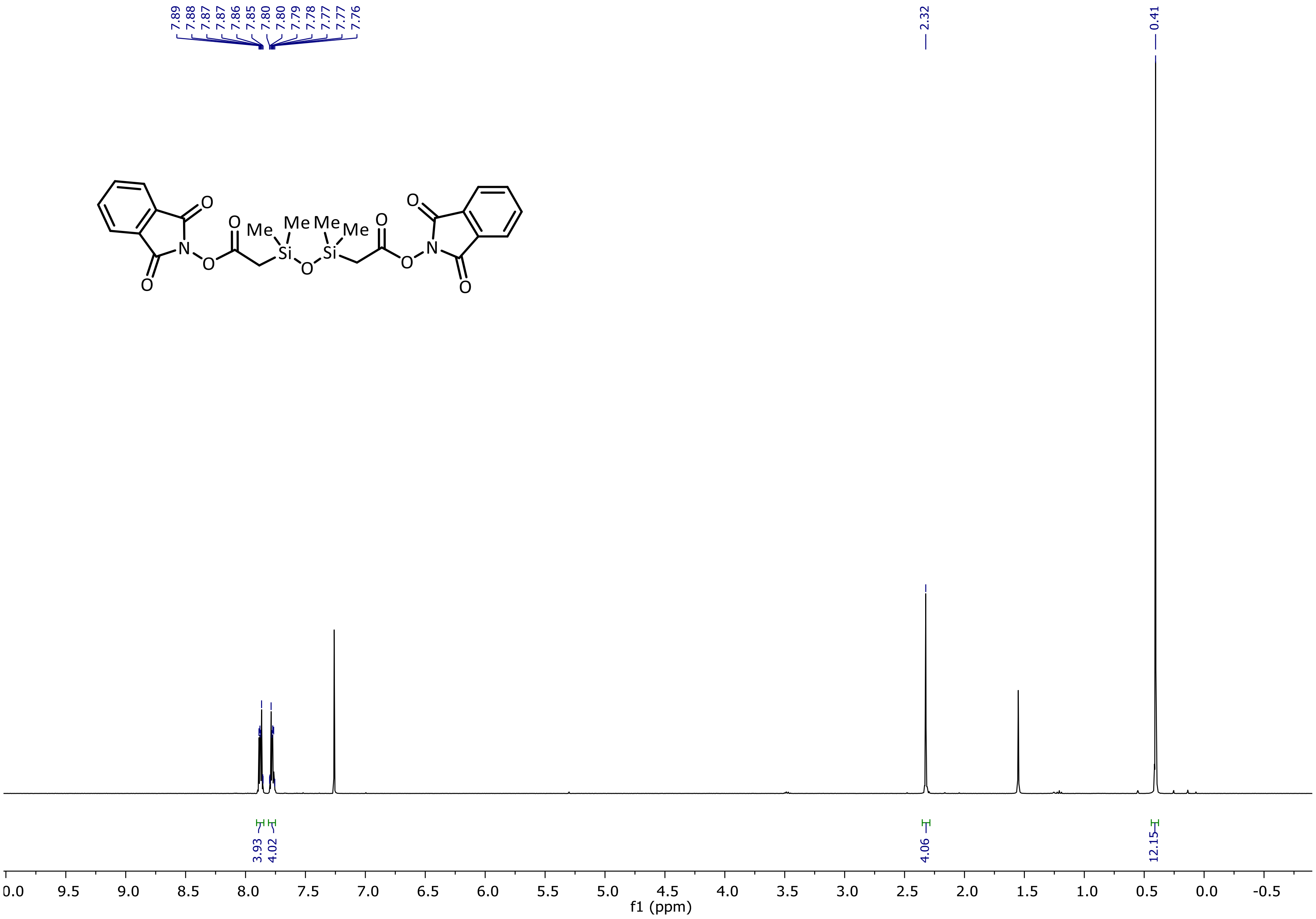

^{13}C-NMR (401 MHz, CDCl$_3$) for compound ***9aa***

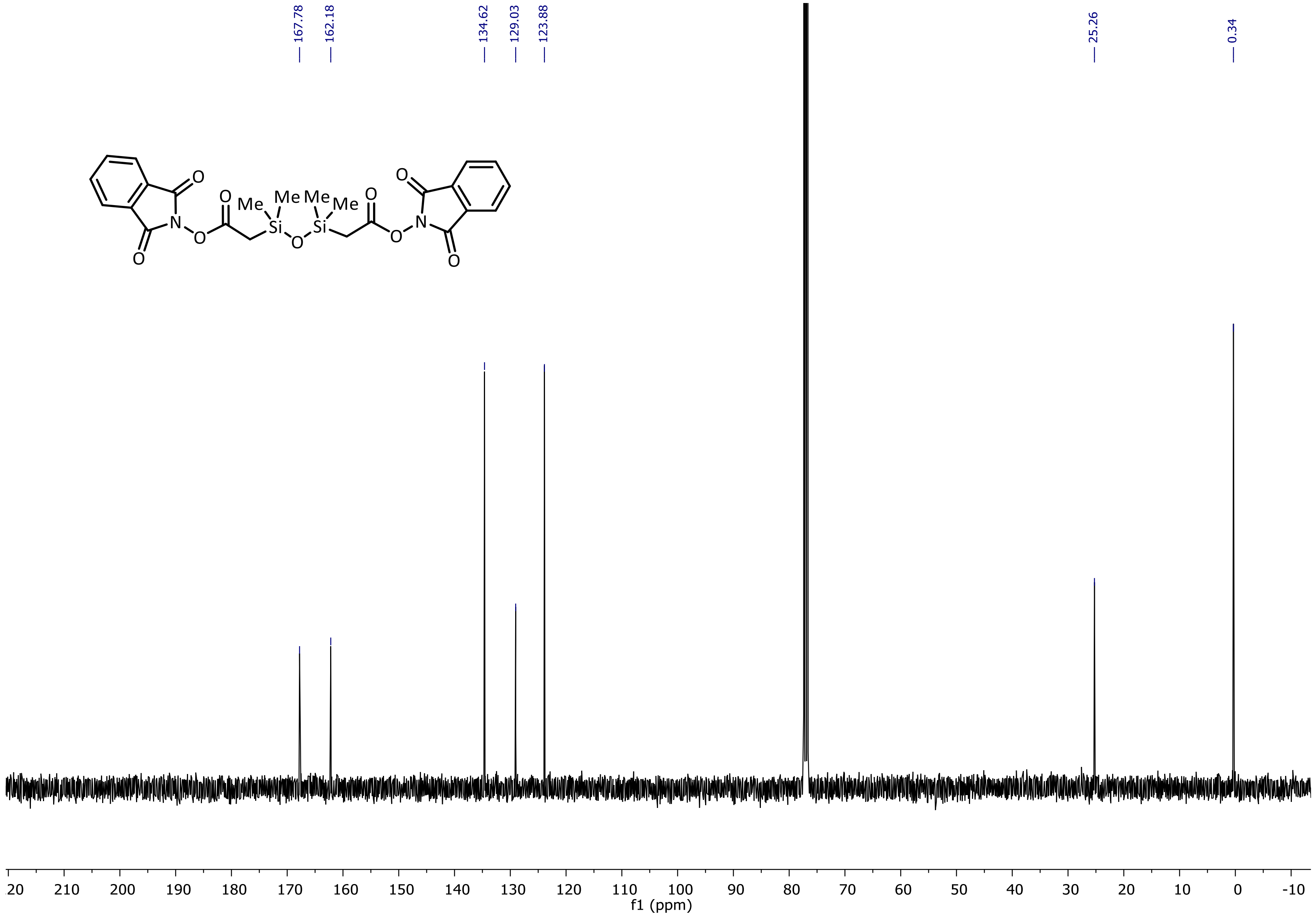

*^{1}H-NMR (400 MHz, $CDCl_3$) for compound **1a***

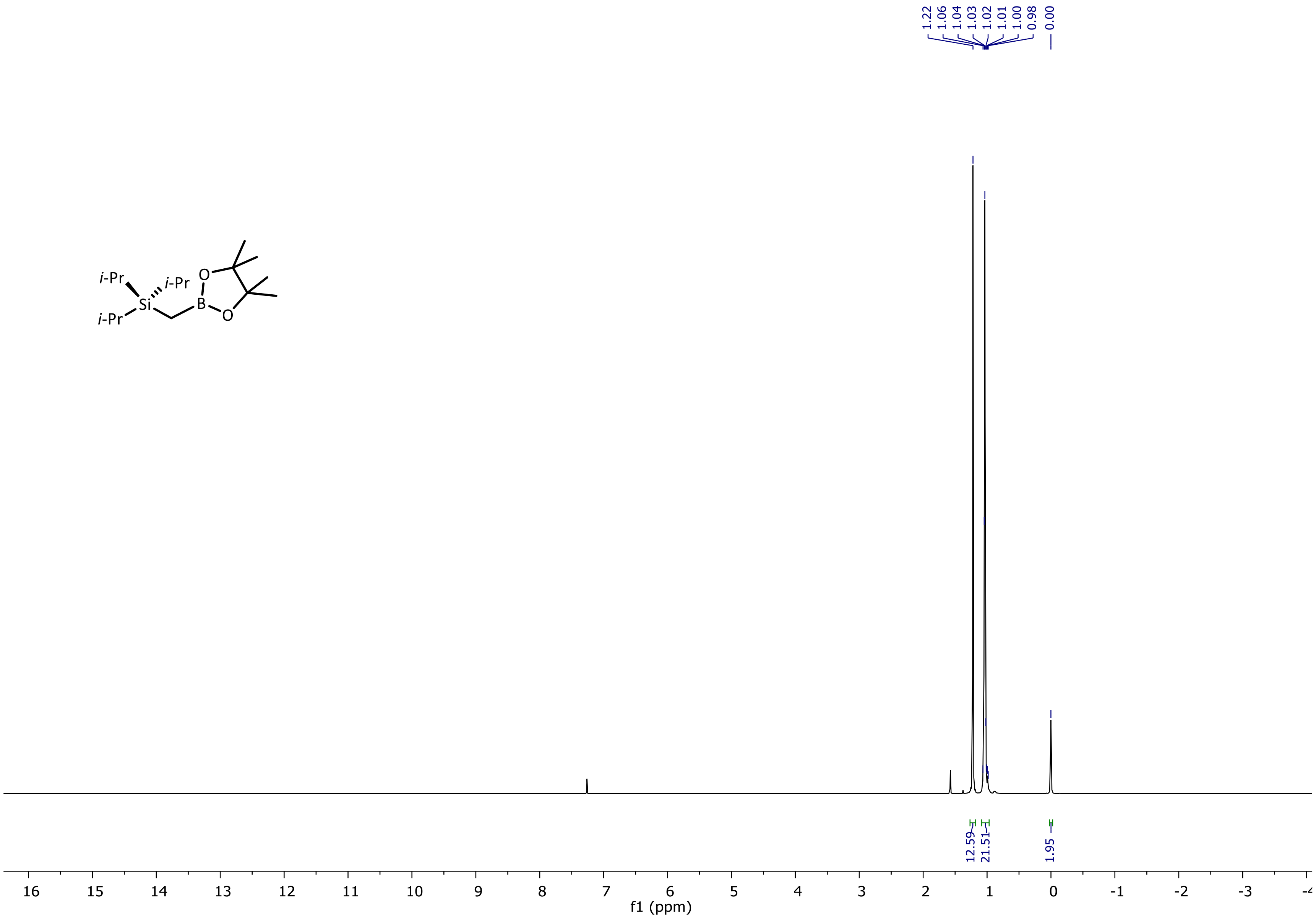

*^{13}C-NMR (101 MHz, CDCl₃) for compound **1a***

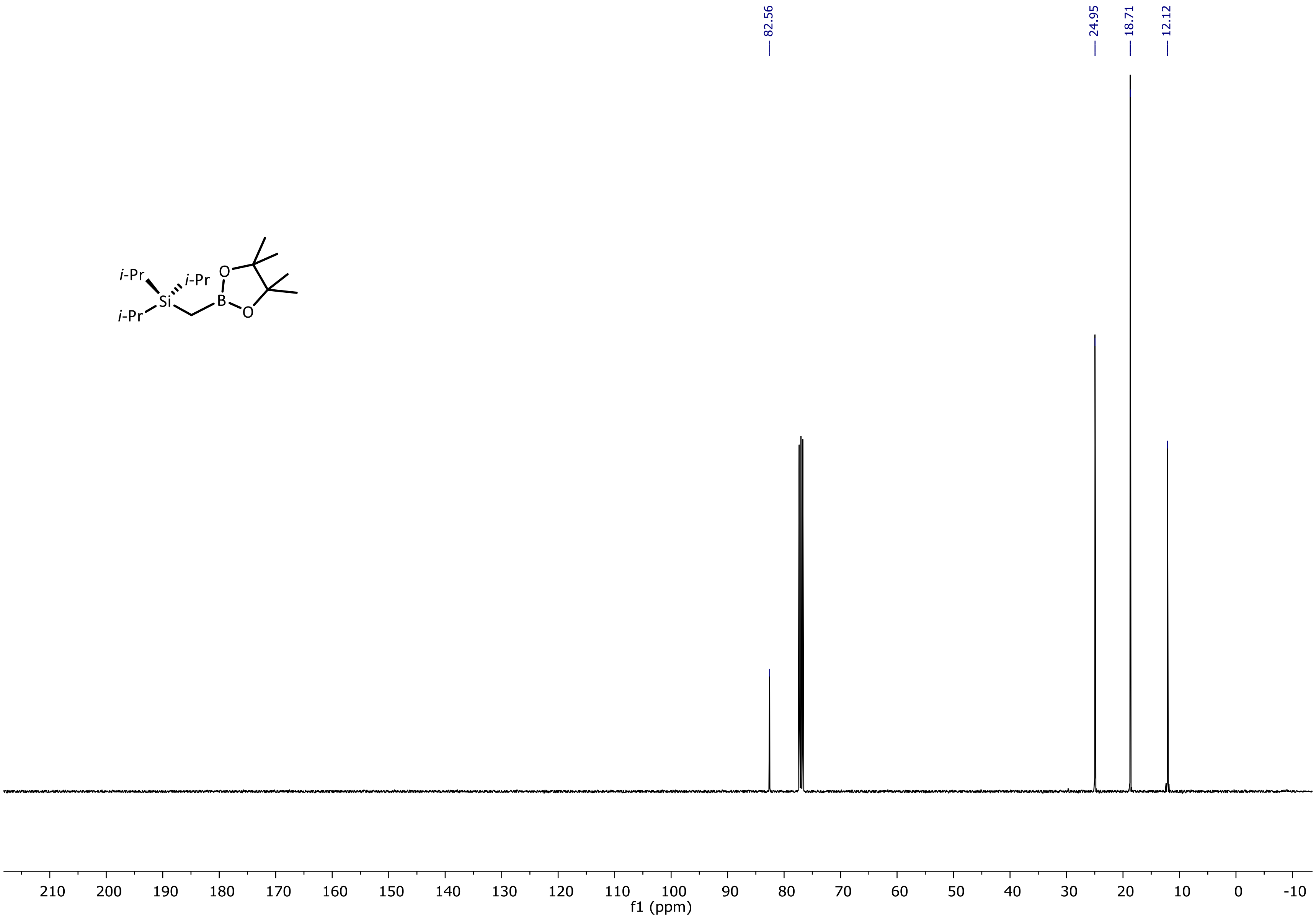

*^{1}H-NMR (400 MHz, $CDCl_3$) for compound **1b***

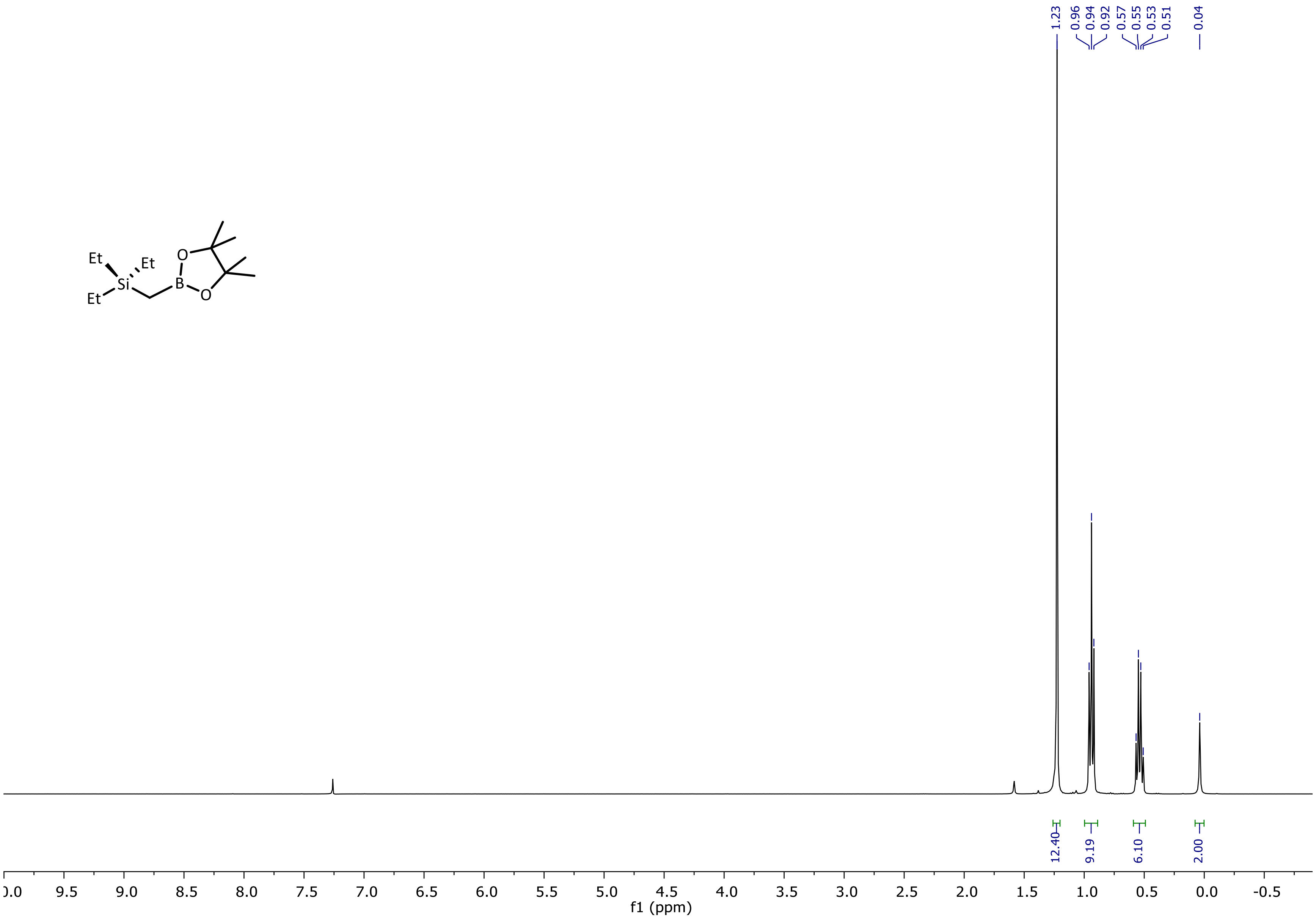

^{13}C-NMR (101 MHz, $CDCl_3$) for compound **1b**

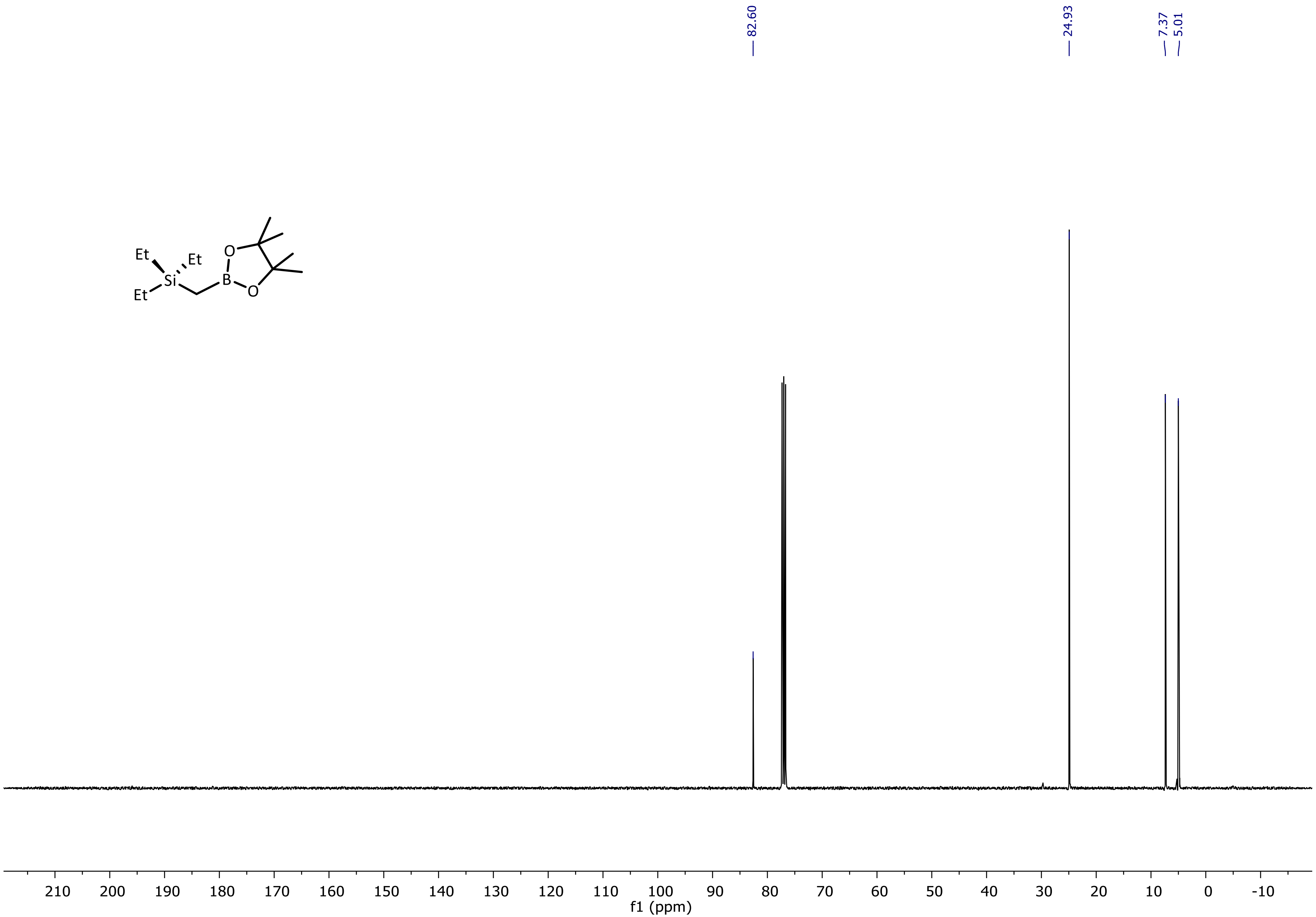

^{1}H-NMR (400 MHz, $CDCl_3$) for compound ***1c***

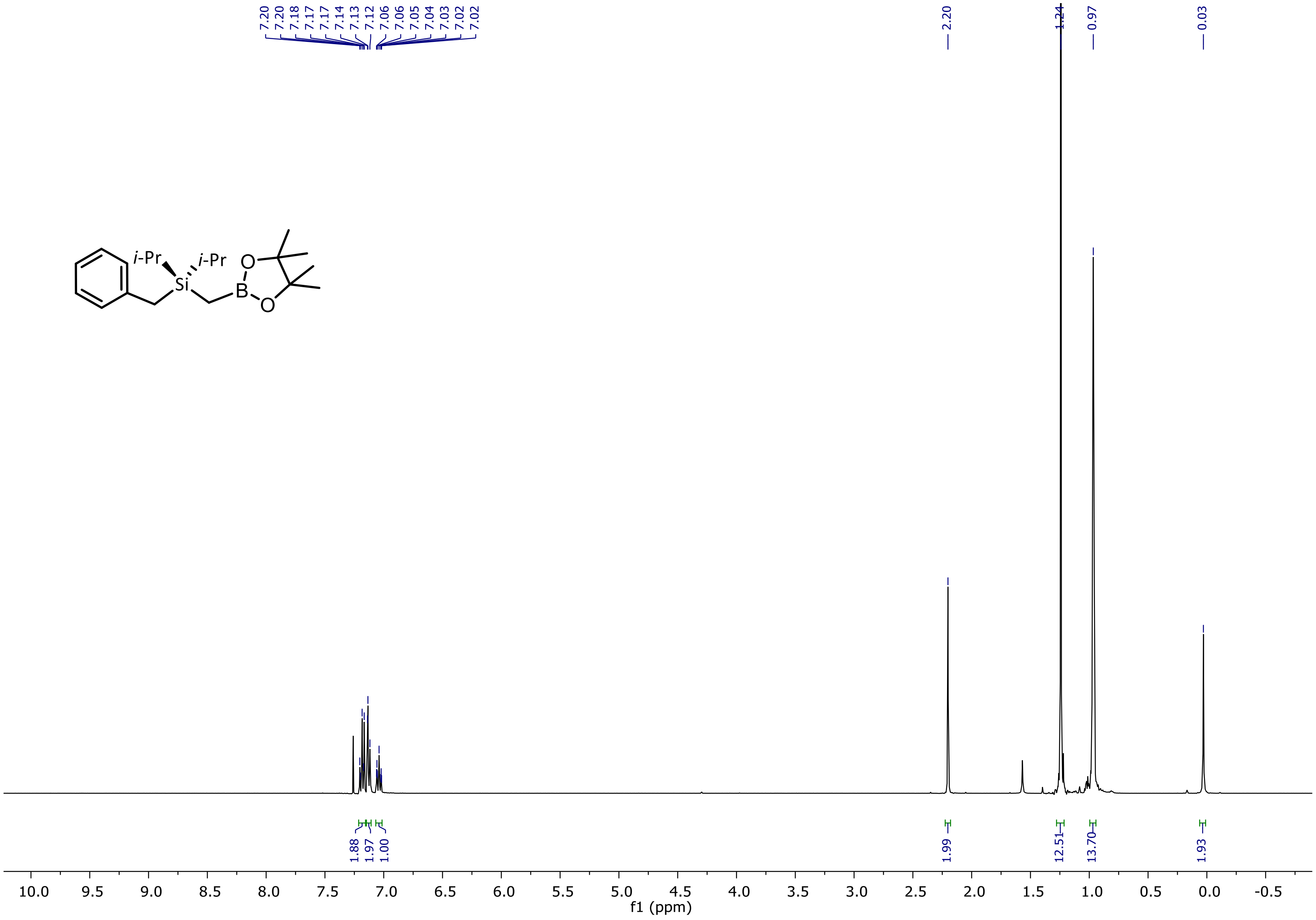

^{13}C-NMR (101 MHz, CDCl$_3$) for compound **1c**

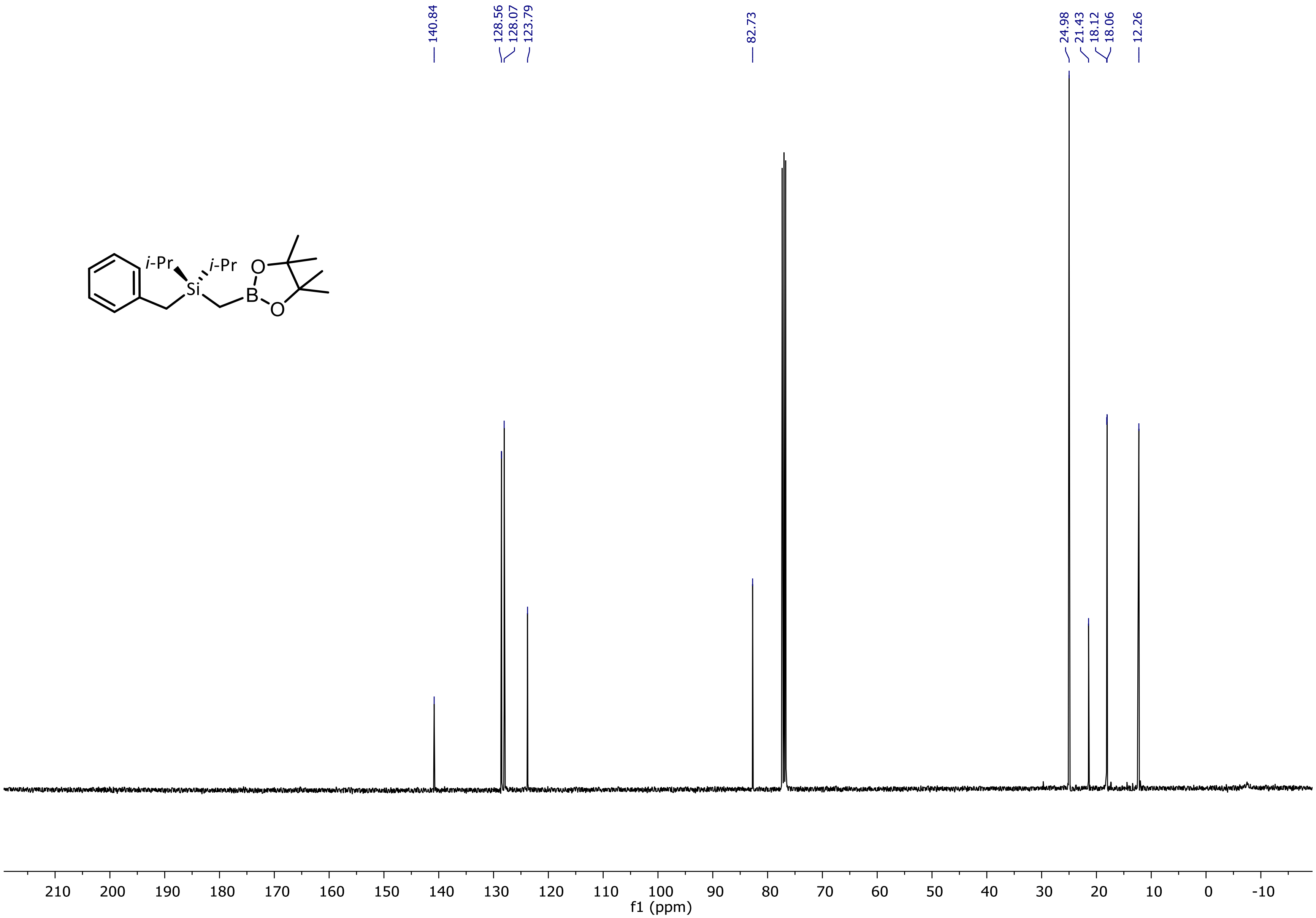

^{1}H-NMR (400 MHz, $CDCl_3$) for compound **1d**

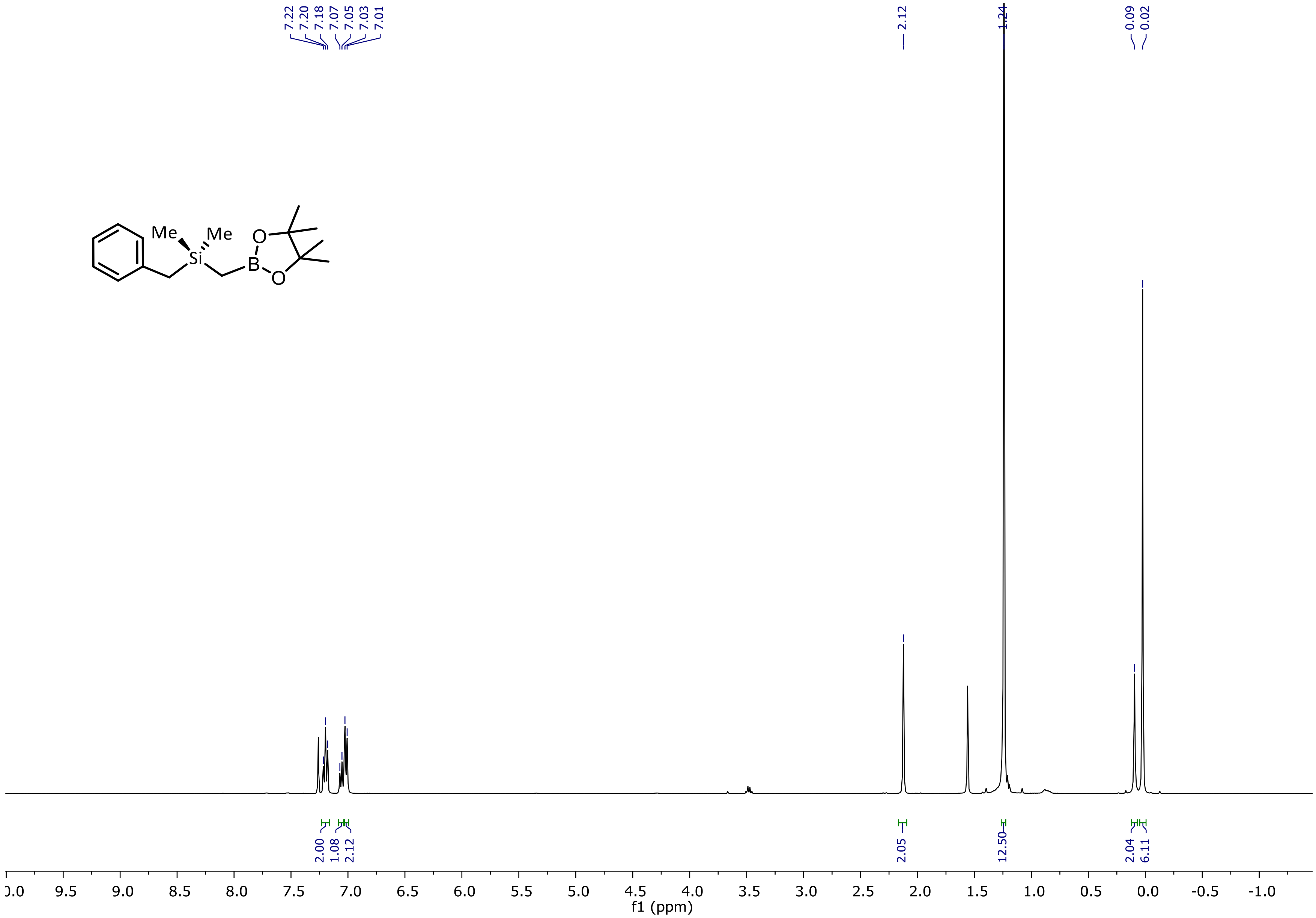

*^{13}C-NMR (101 MHz, CDCl$_3$) for compound **1d***

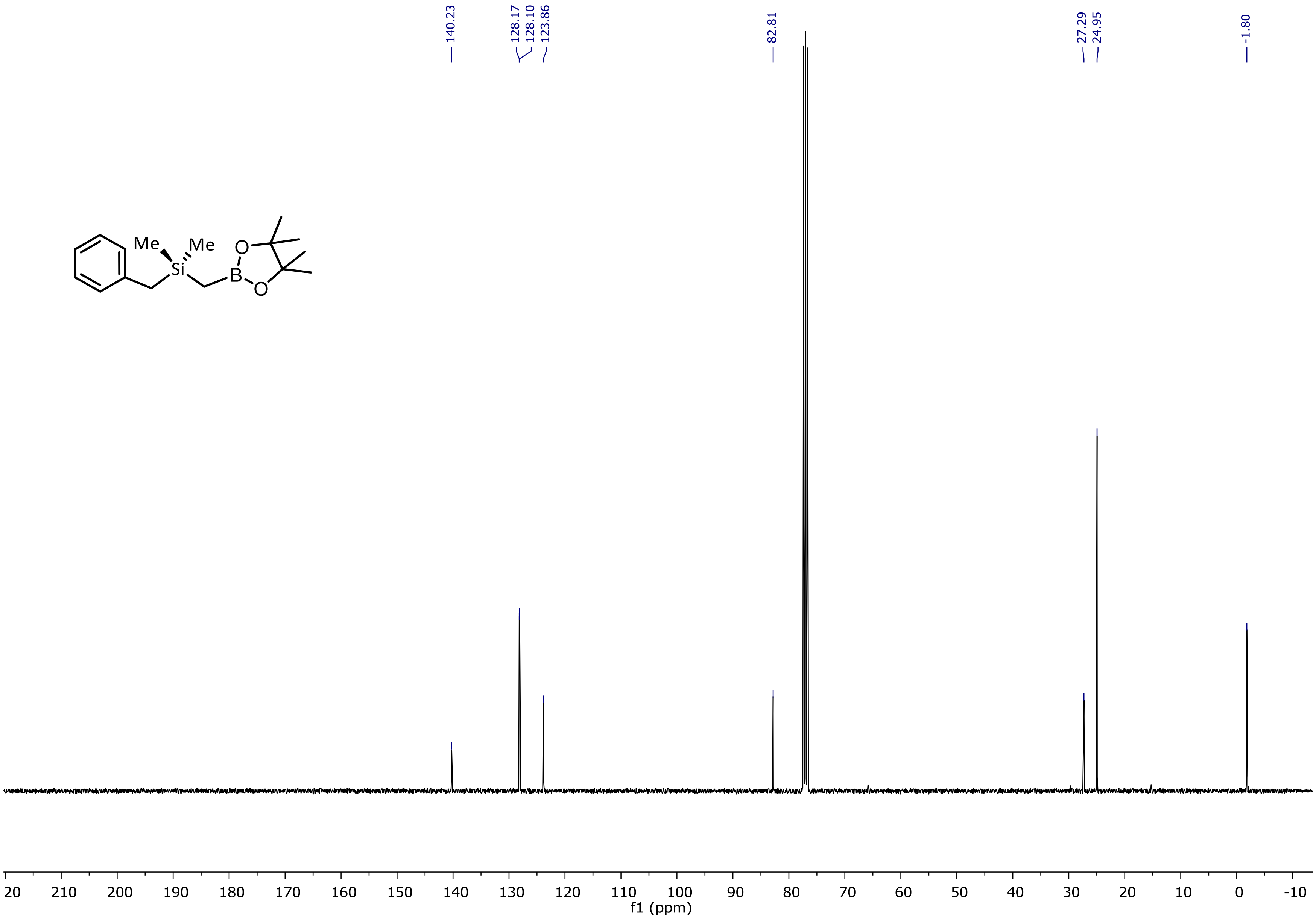

^{1}H-NMR (400 MHz, $CDCl_3$) for compound ***1e***

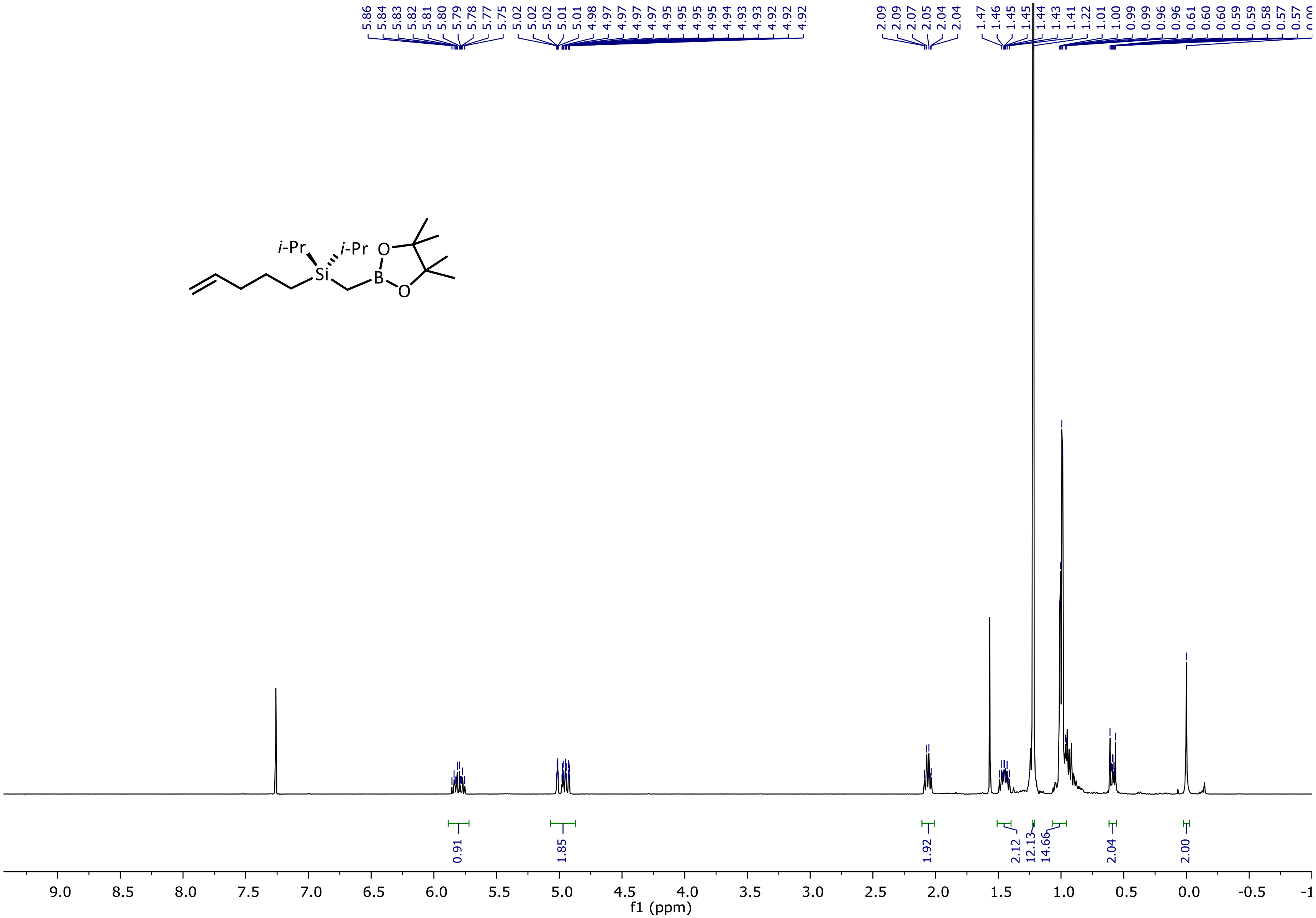

*^{13}C-NMR (101 MHz, CDCl$_3$) for compound **1e***

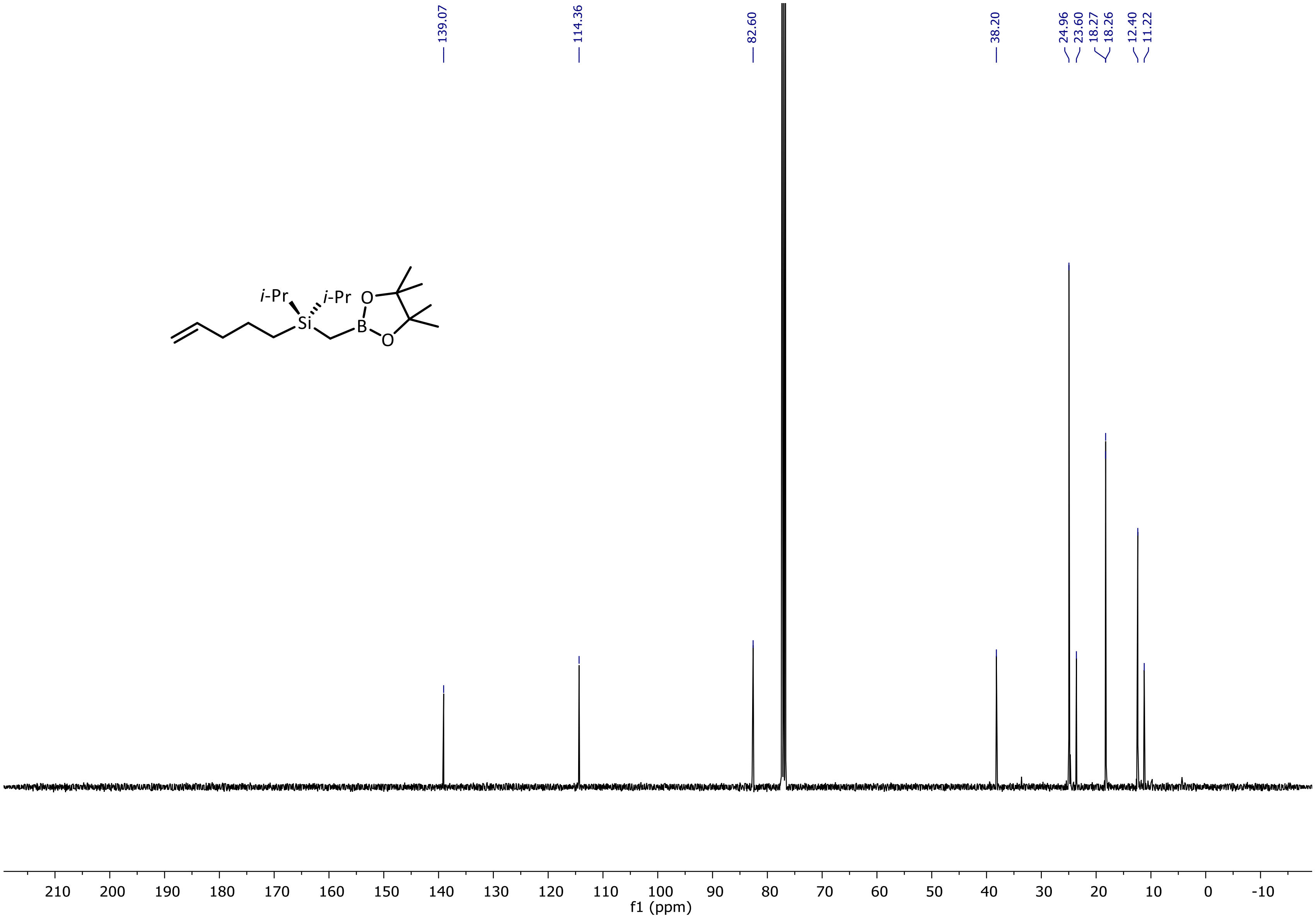

^{1}H-NMR (400 MHz, $CDCl_3$) for compound ***1f***

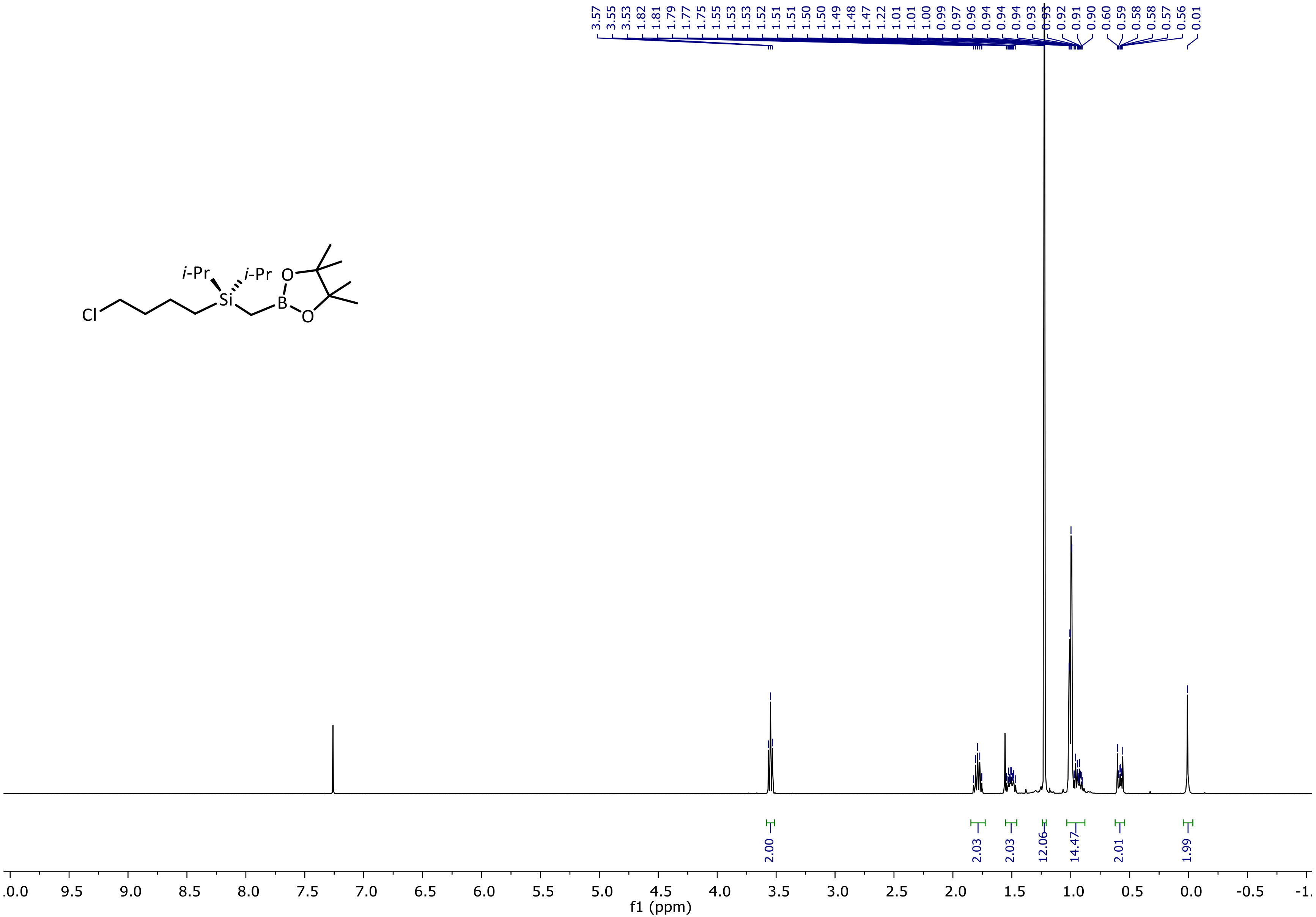

*^{13}C-NMR (101 MHz, CDCl$_3$) for compound **1f***

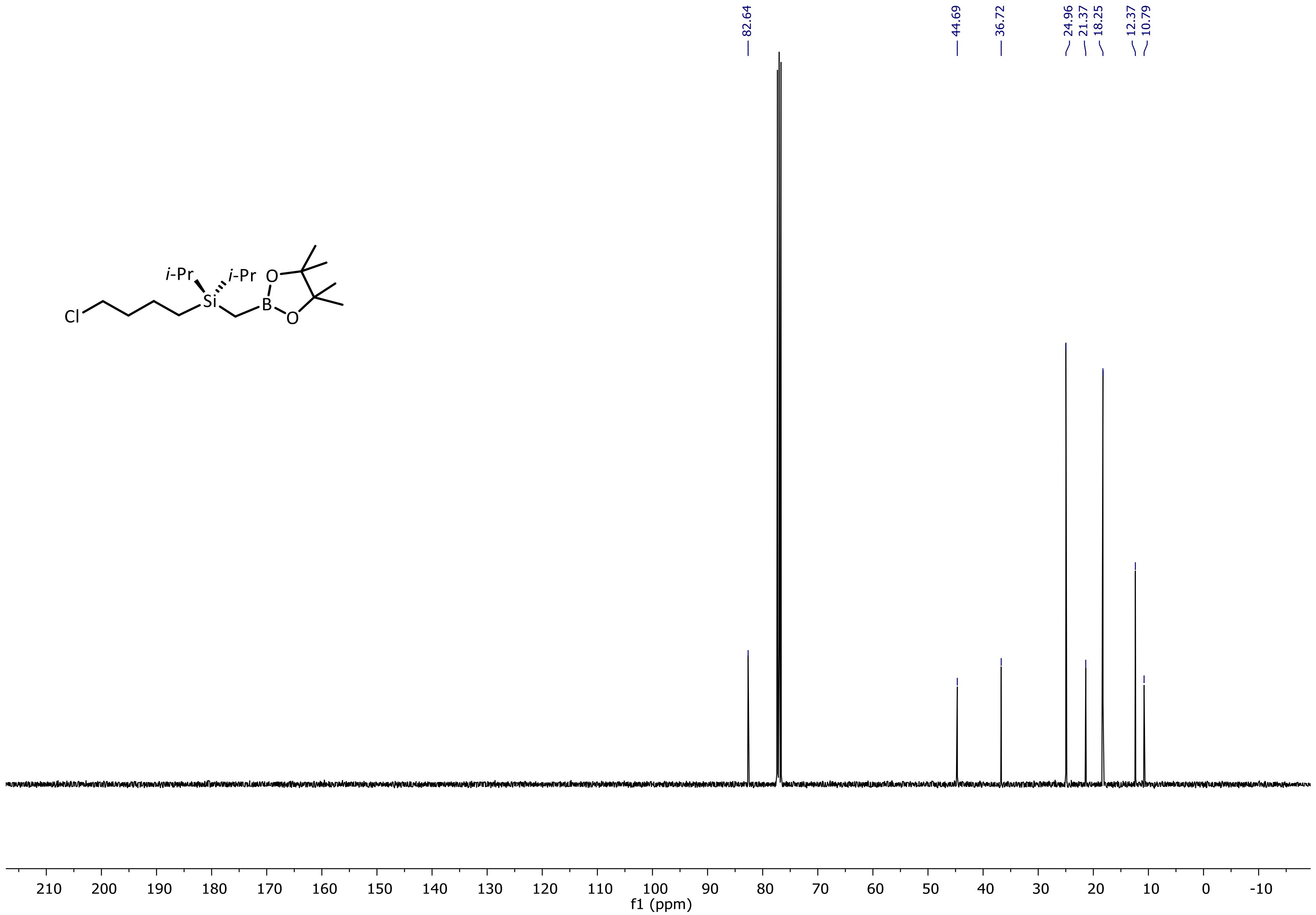

^{1}H-NMR (400 MHz, $CDCl_3$) for compound ***1g***

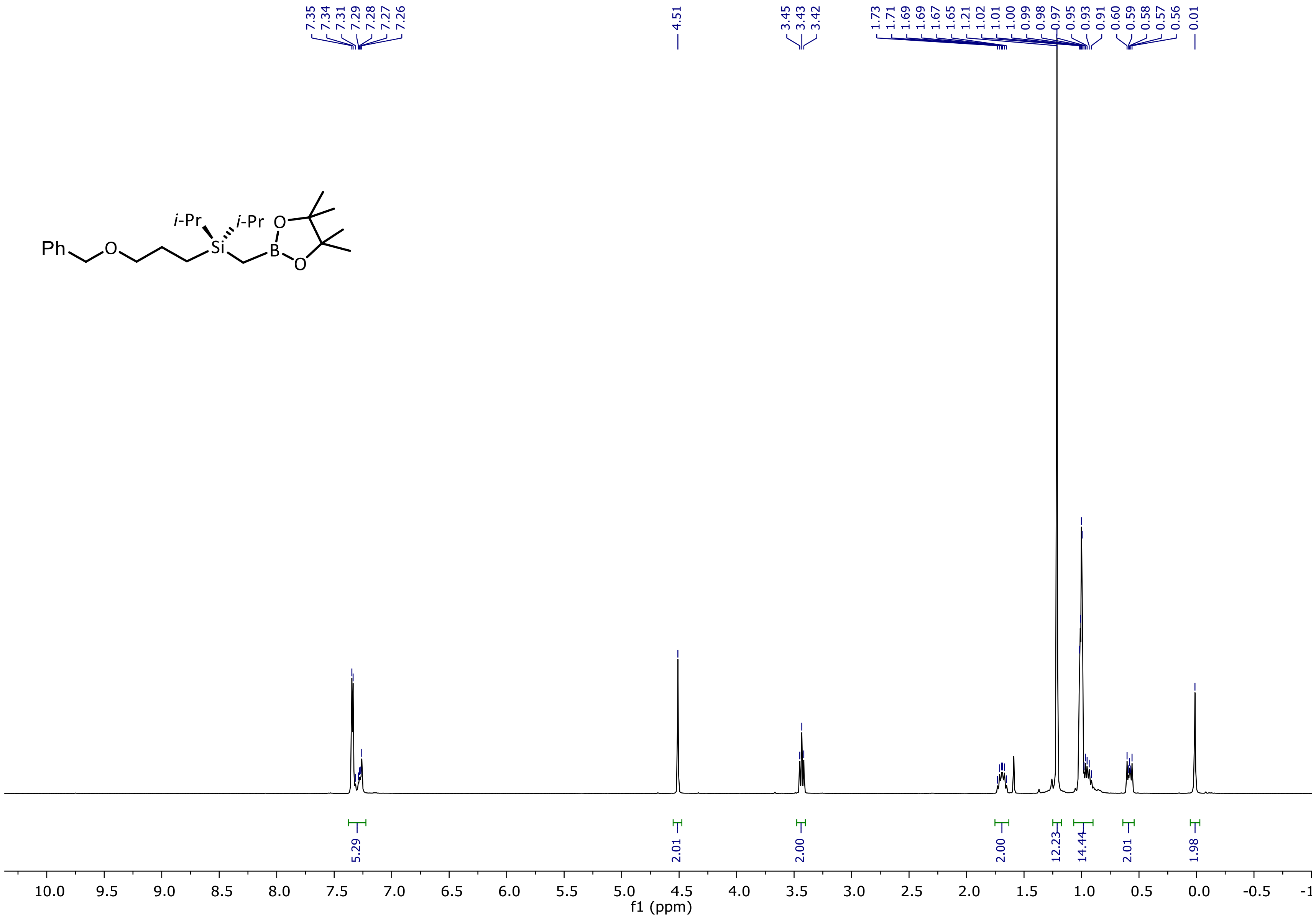

*^{13}C-NMR (101 MHz, CDCl₃) for compound **1g***

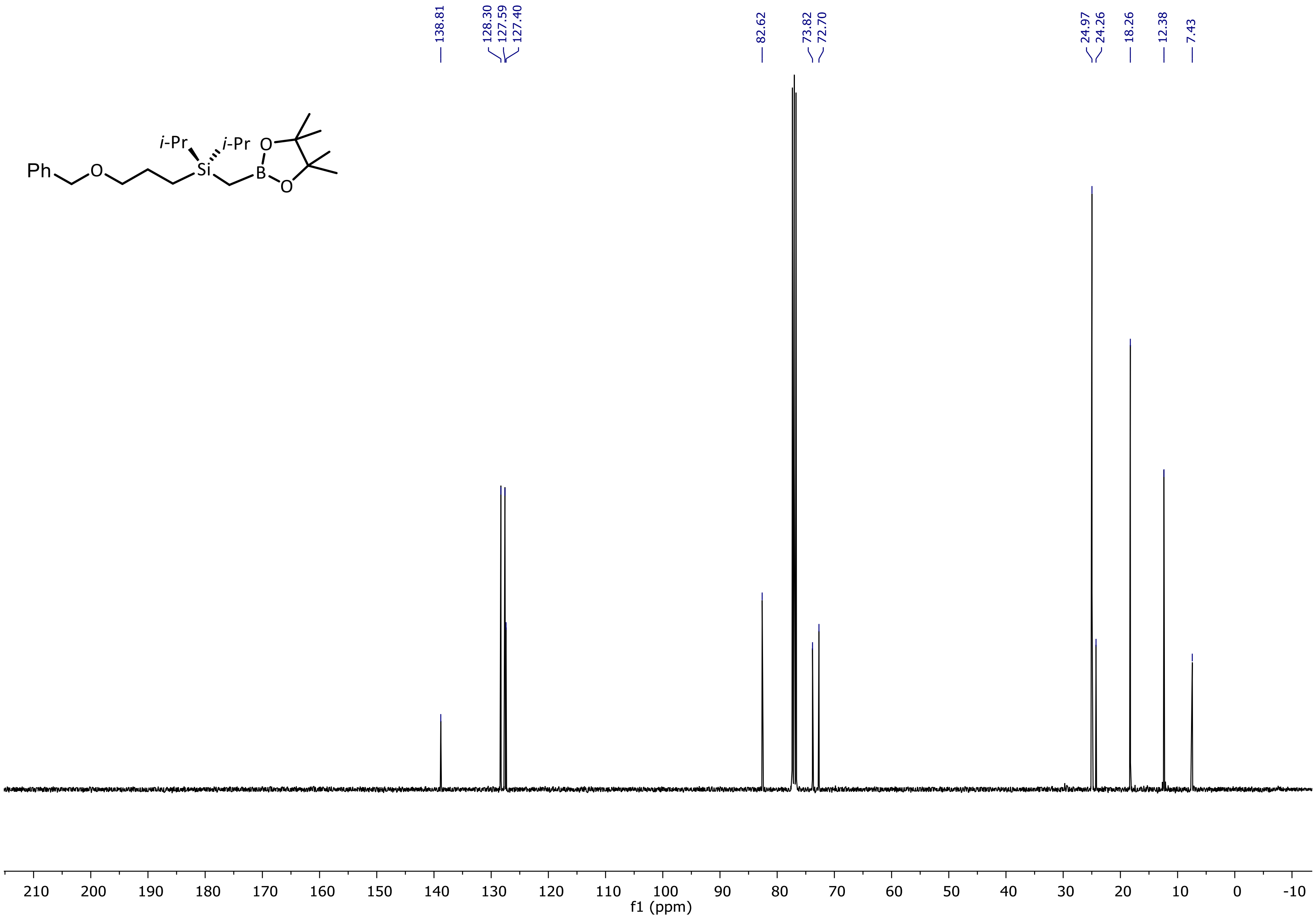

*^{1}H-NMR (400 MHz, $CDCl_3$) for compound **1h***

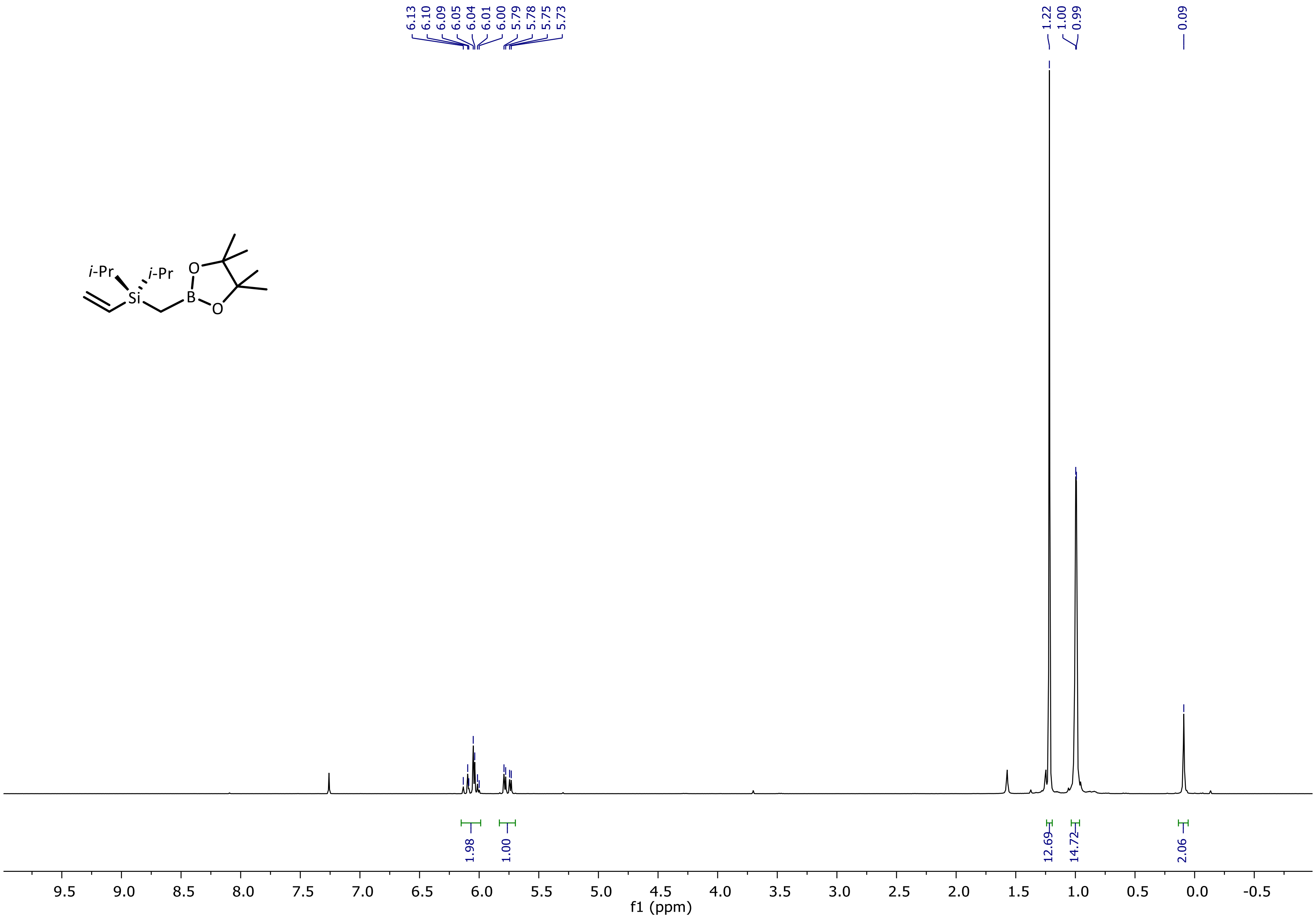

*^{13}C-NMR (101 MHz, CDCl$_3$) for compound **1h***

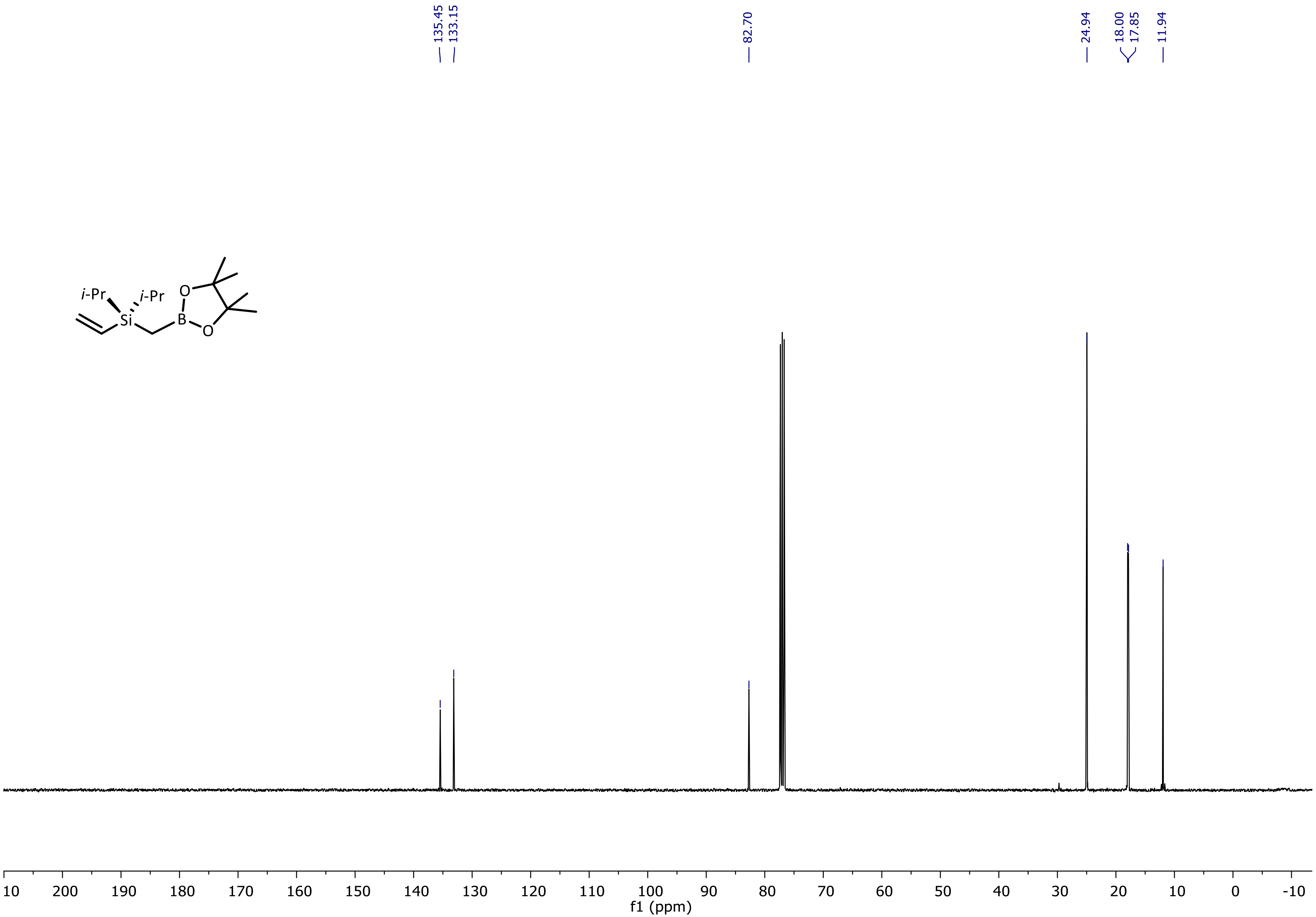

^1H-NMR (400 MHz, $CDCl_3$) for compound **1i**

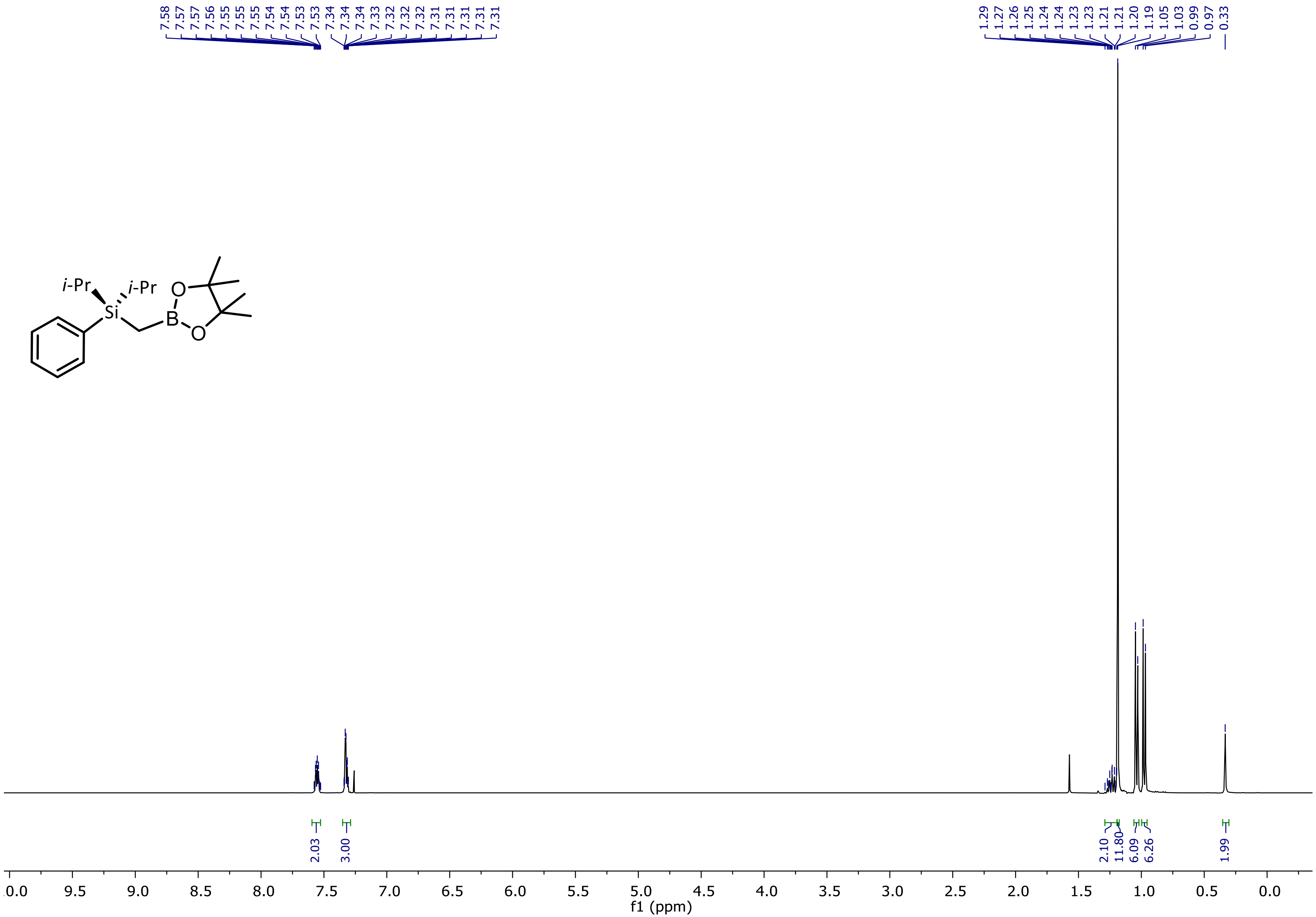

*^{13}C-NMR (101 MHz, $CDCl_3$) for compound **1i***

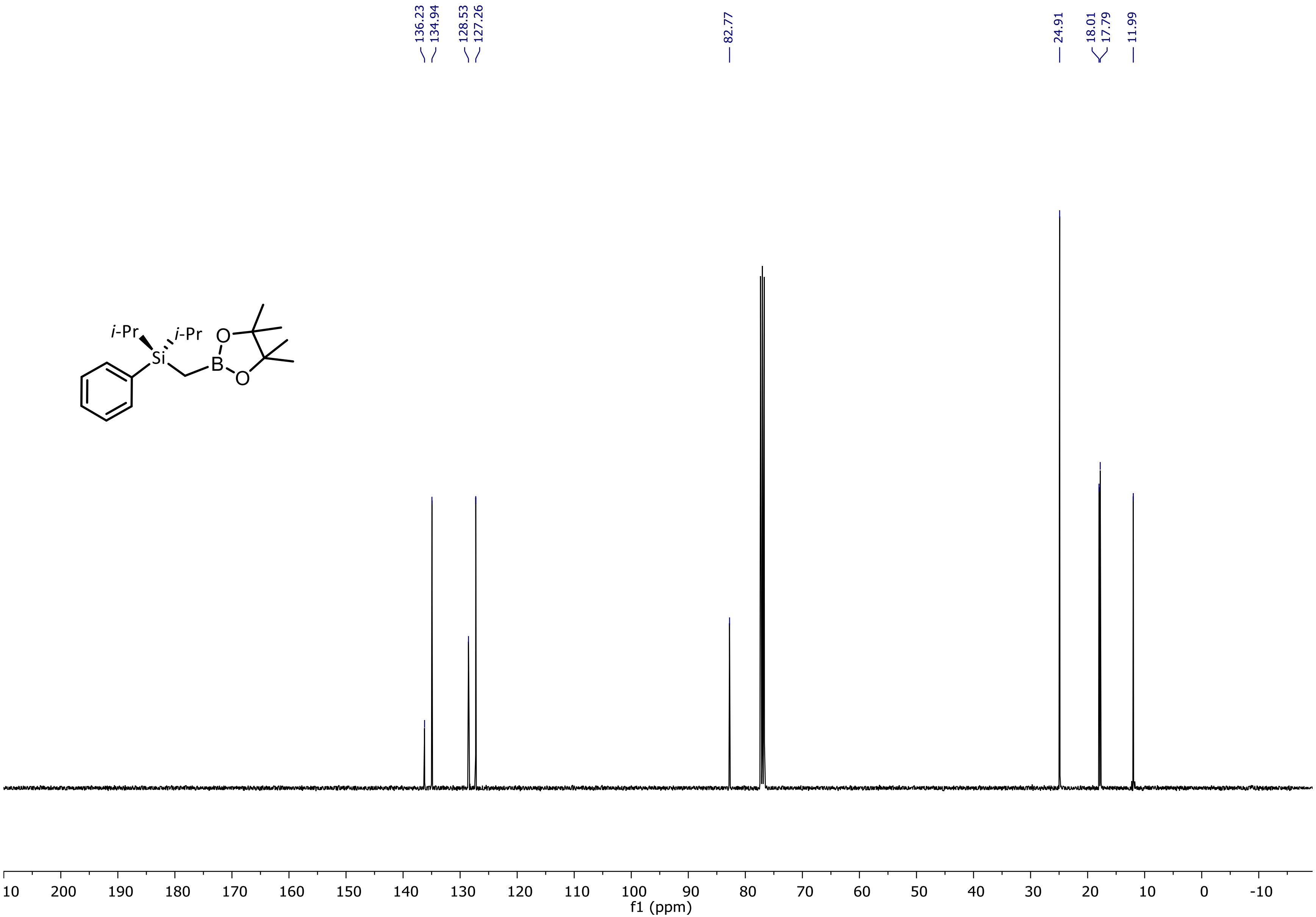

^{1}H-NMR (400 MHz, $CDCl_3$) for compound **1j**

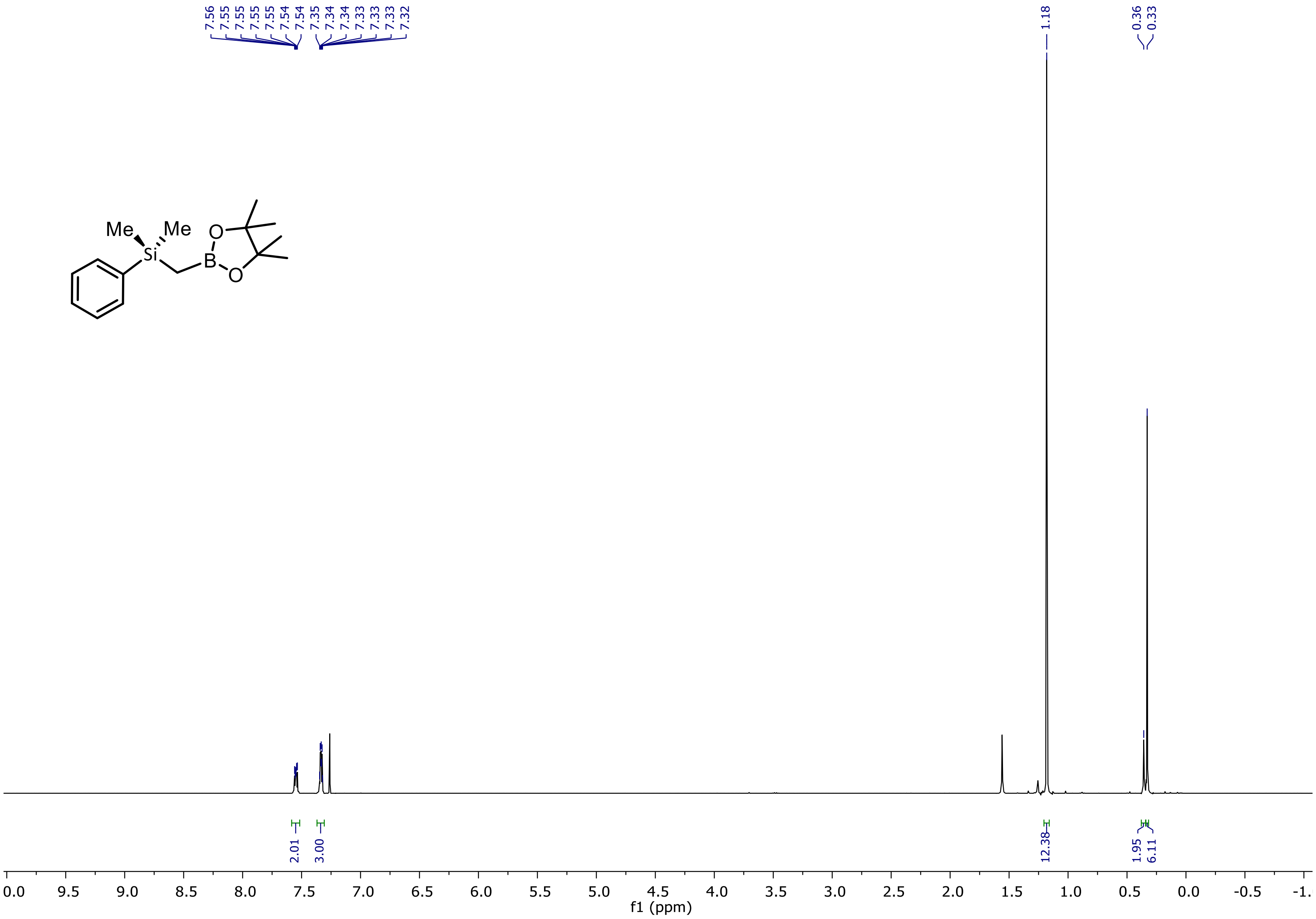

^{13}C-NMR (101 MHz, $CDCl_3$) for compound **1j**

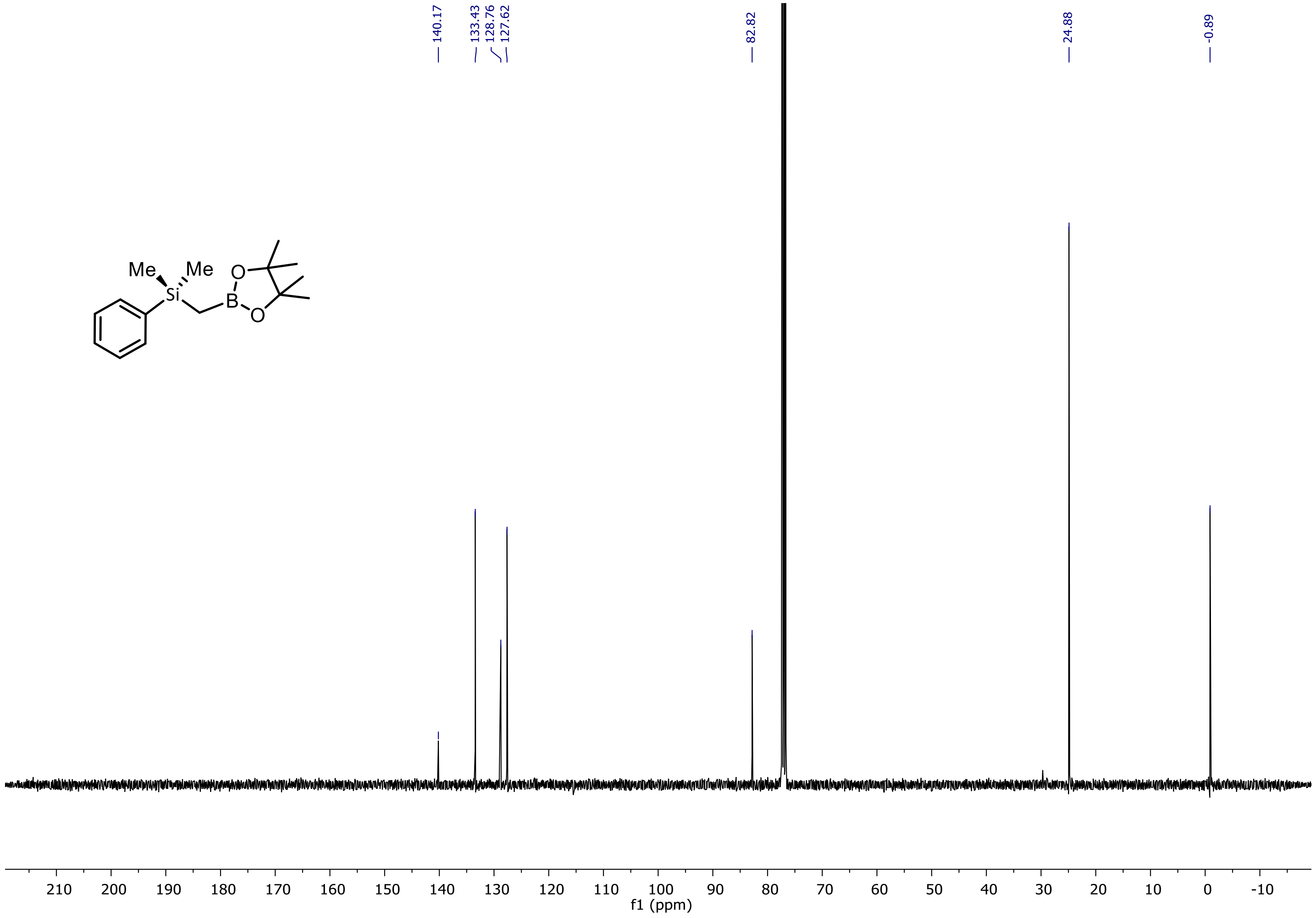

^{1}H-NMR (400 MHz, $CDCl_3$) for compound ***1k***

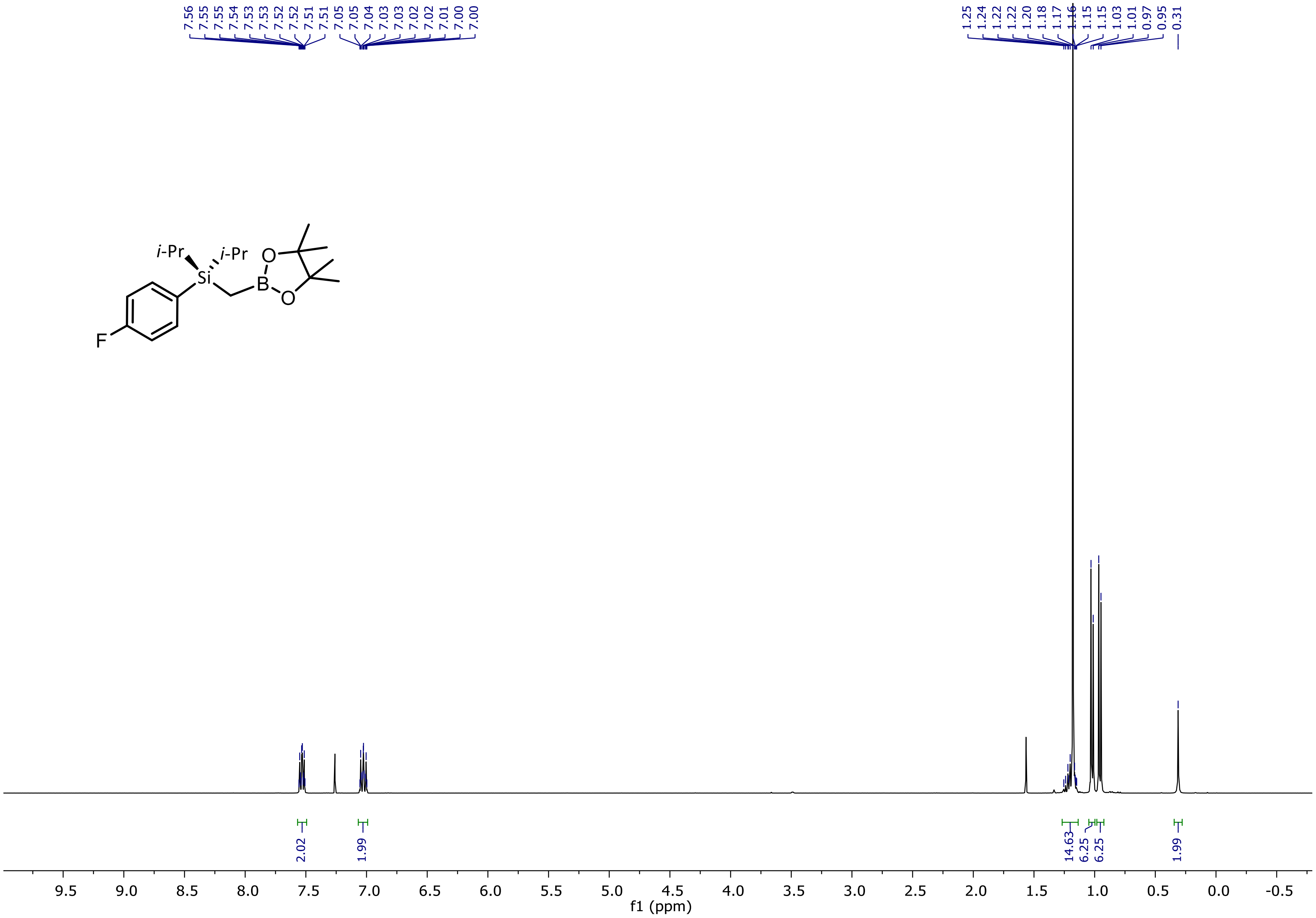

^{13}C-NMR (101 MHz, $CDCl_3$) for compound **1k**

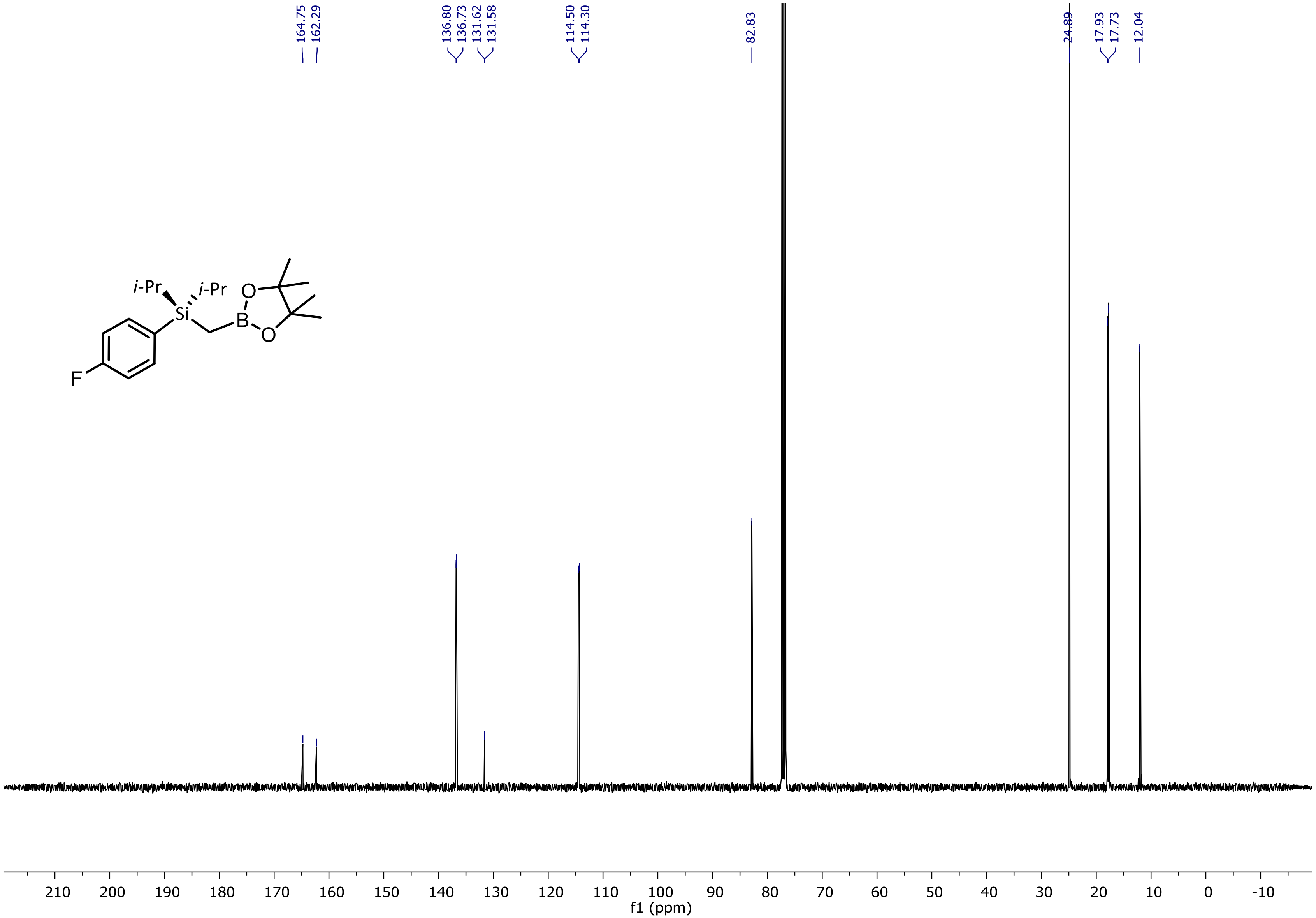

^{19}F-NMR (377 MHz, CDCl$_3$) for compound ***1k***

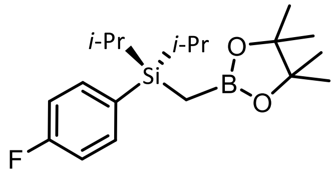

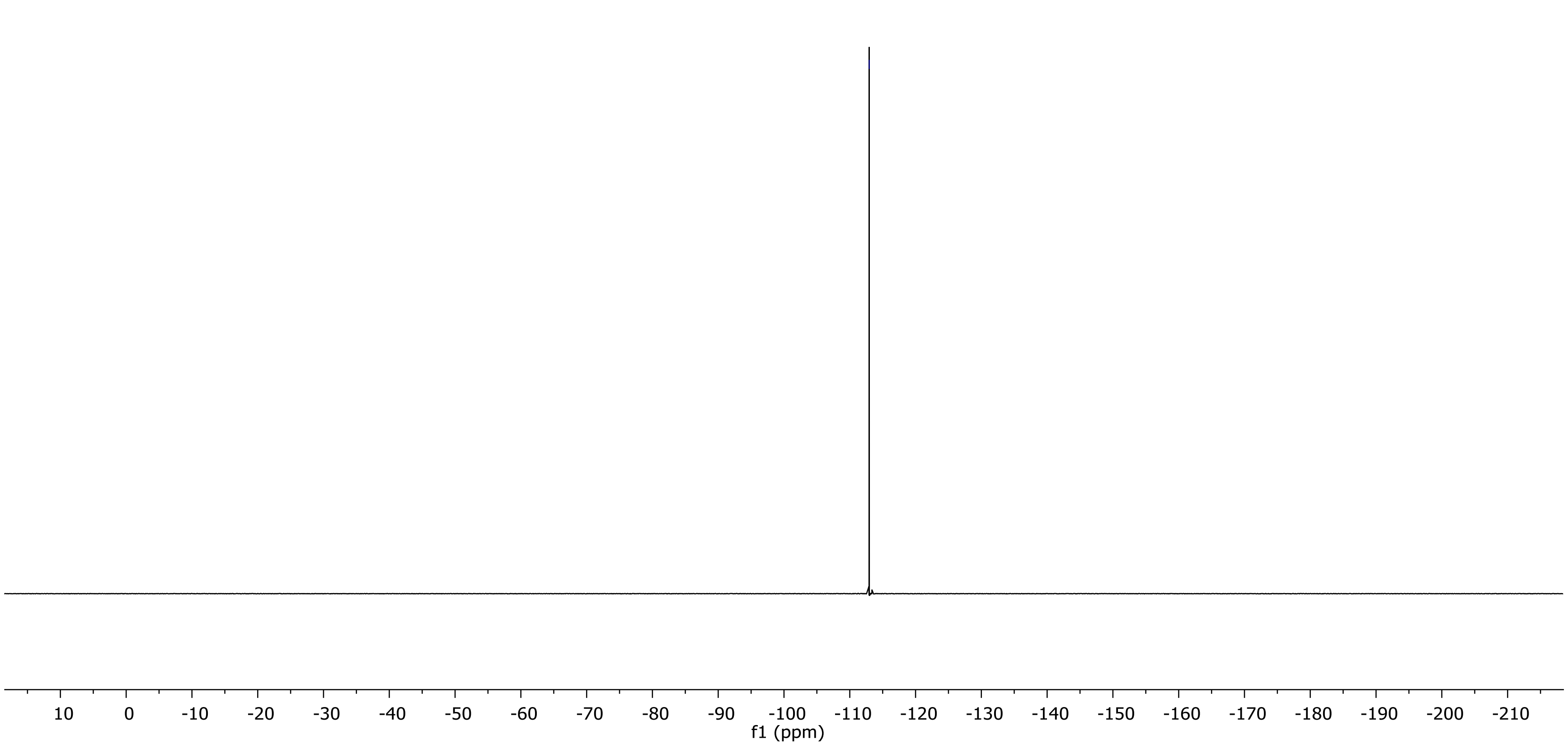

*^{1}H-NMR (400 MHz, $CDCl_3$) for compound **1l***

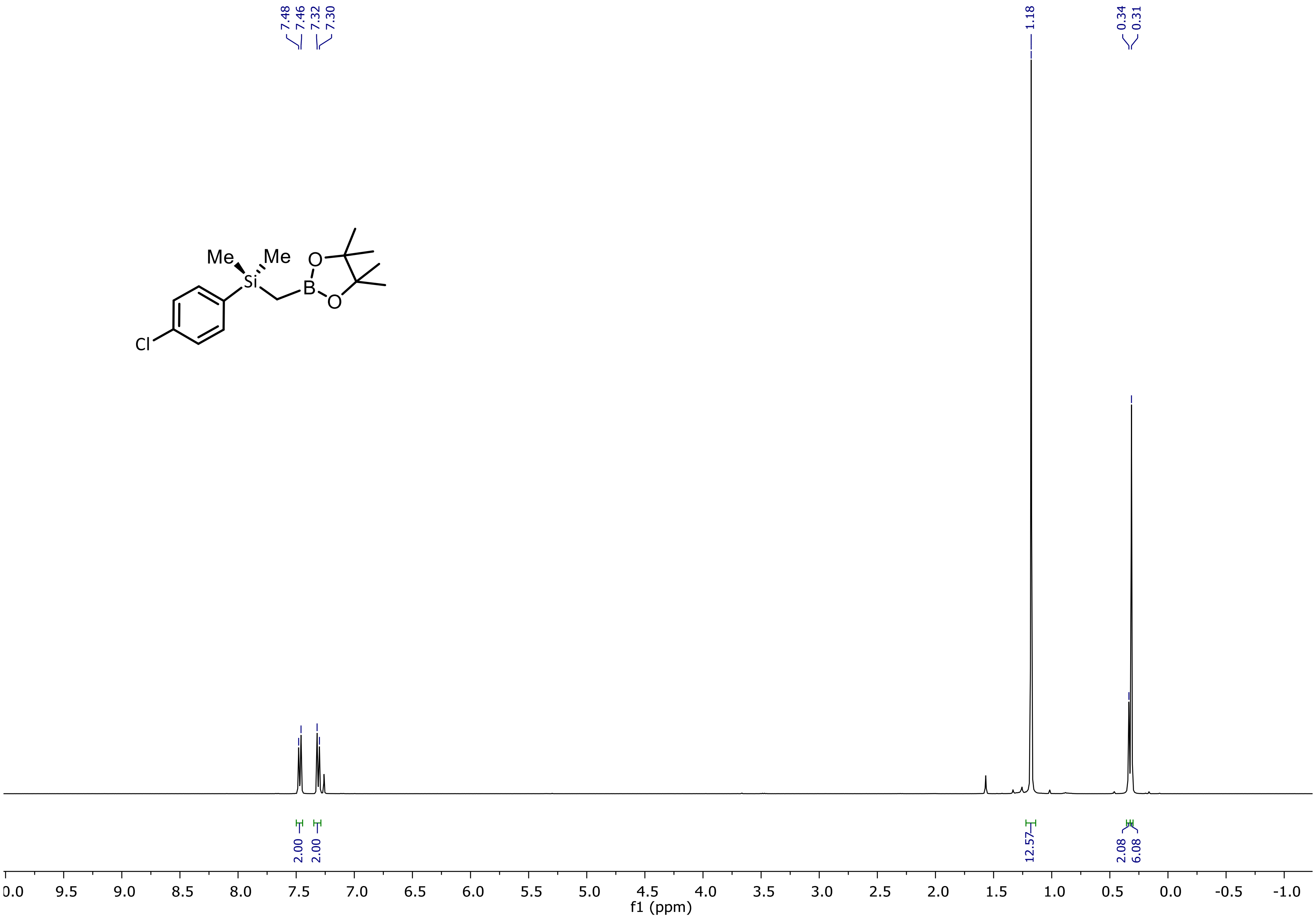

^{13}C-NMR (101 MHz, CDCl$_3$) for compound **1l**

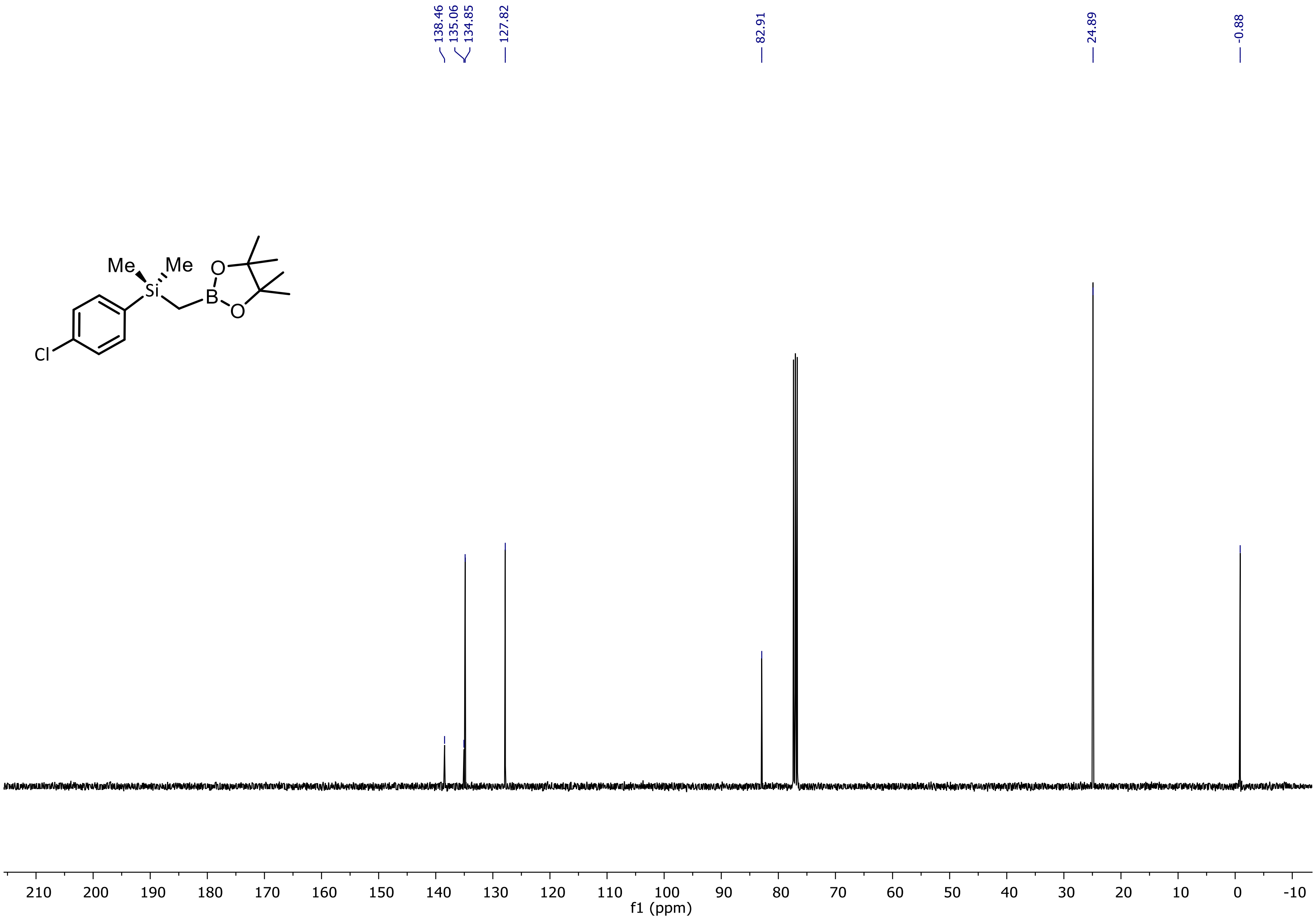

*^{1}H-NMR (400 MHz, $CDCl_3$) for compound **1m***

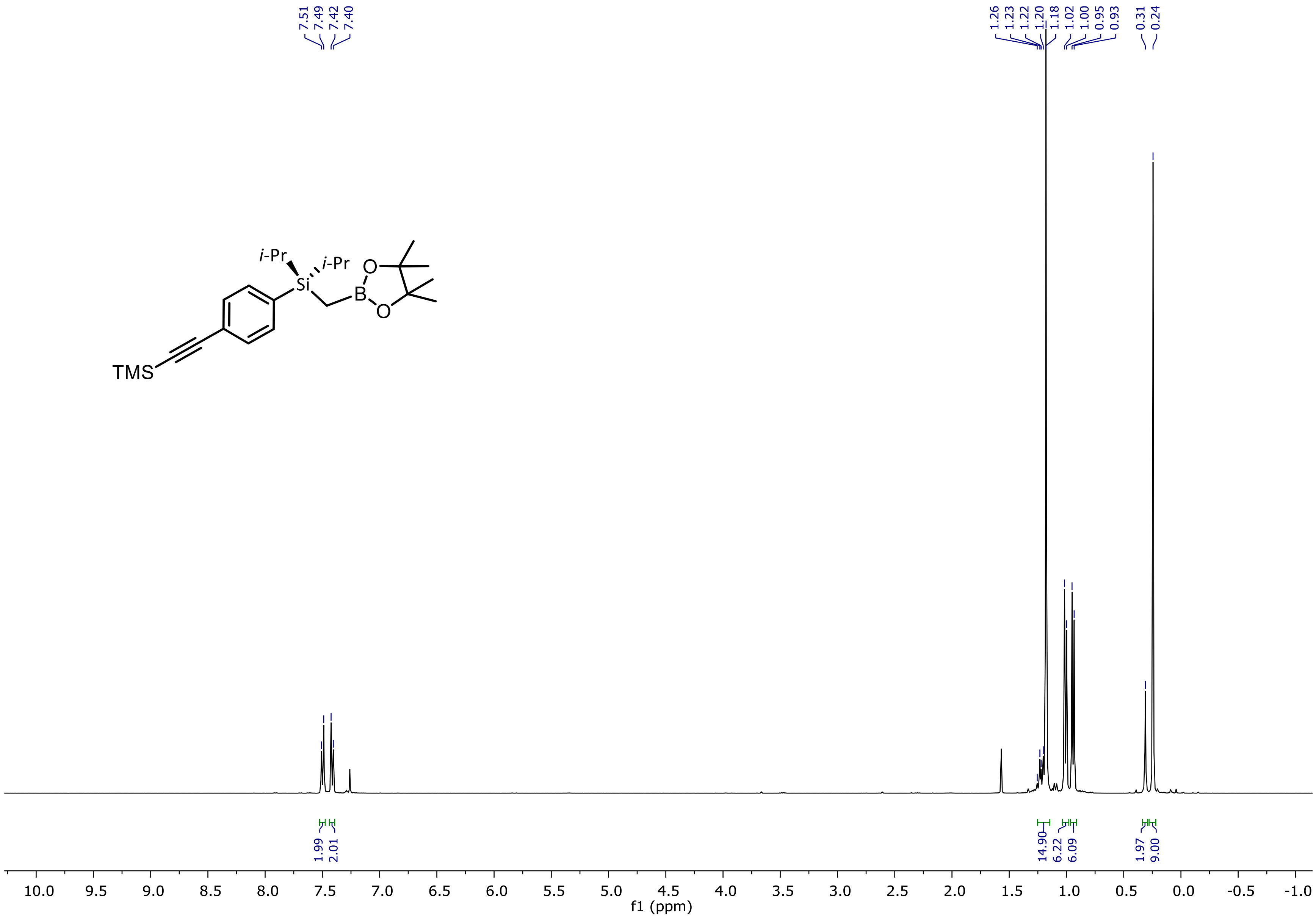

*^{13}C-NMR (101 MHz, CDCl$_3$) for compound **1m***

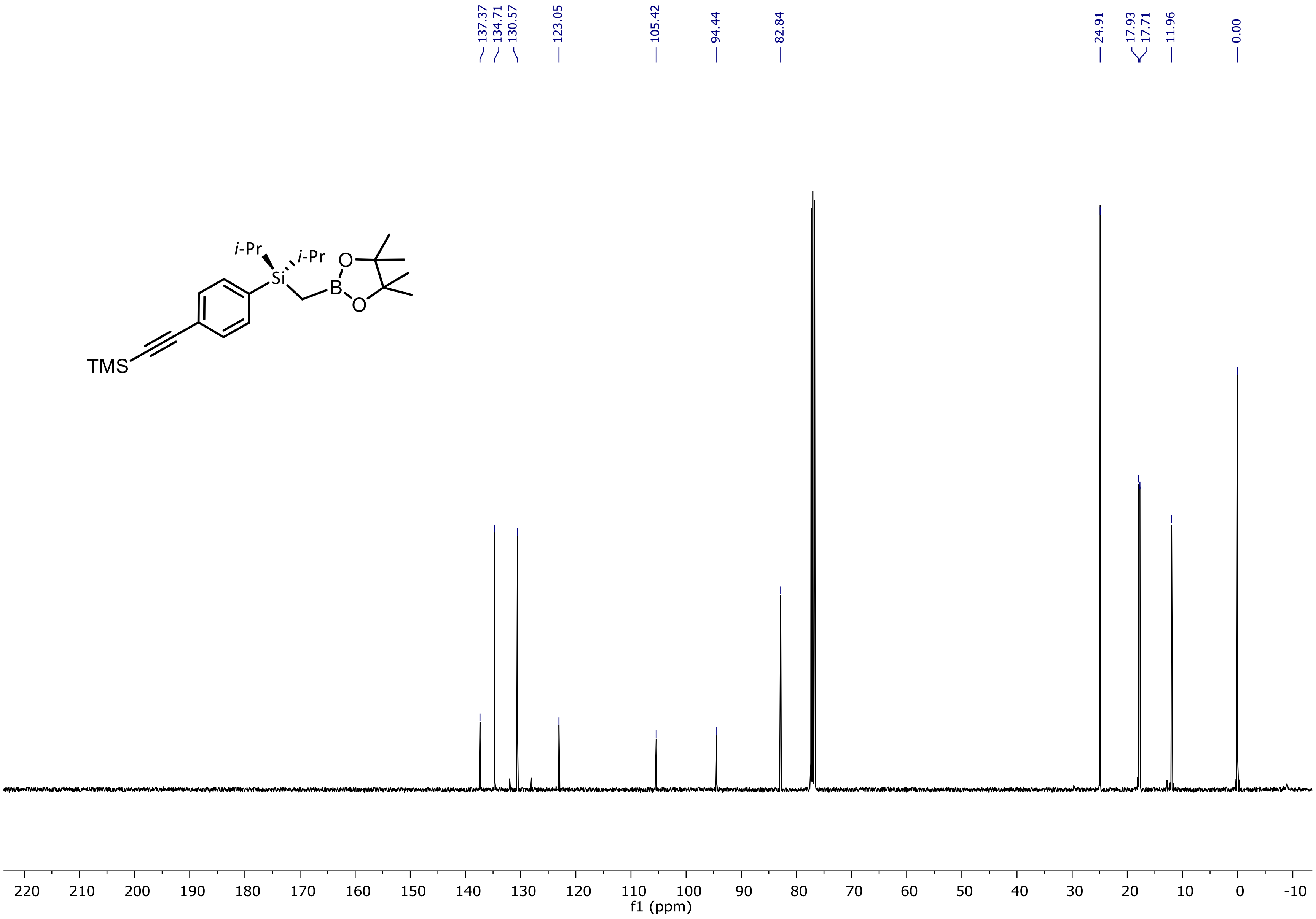

^{1}H-NMR (400 MHz, CDCl$_3$) for compound ***1n***

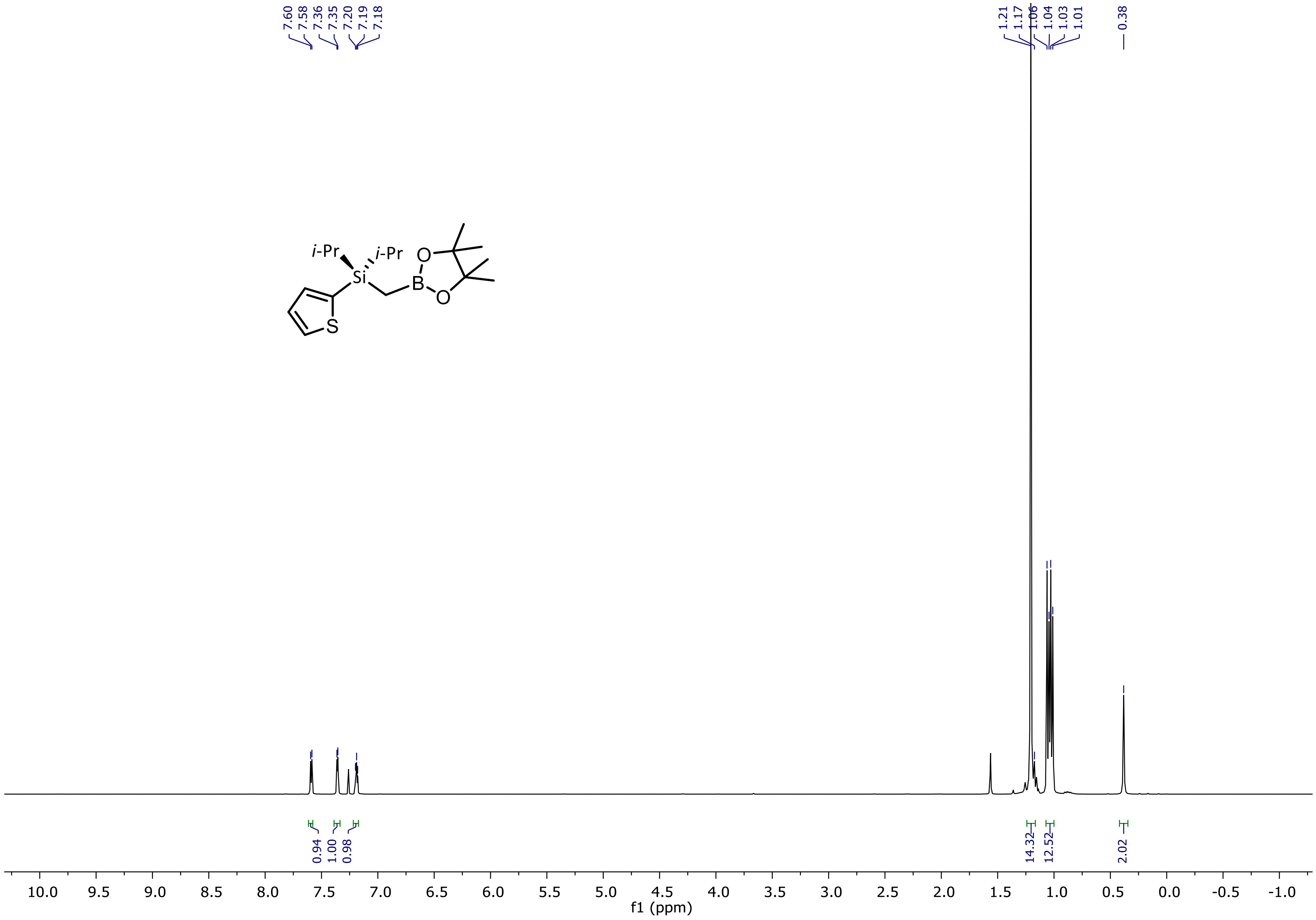

^{13}C-NMR (101 MHz, $CDCl_3$) for compound ***1n***

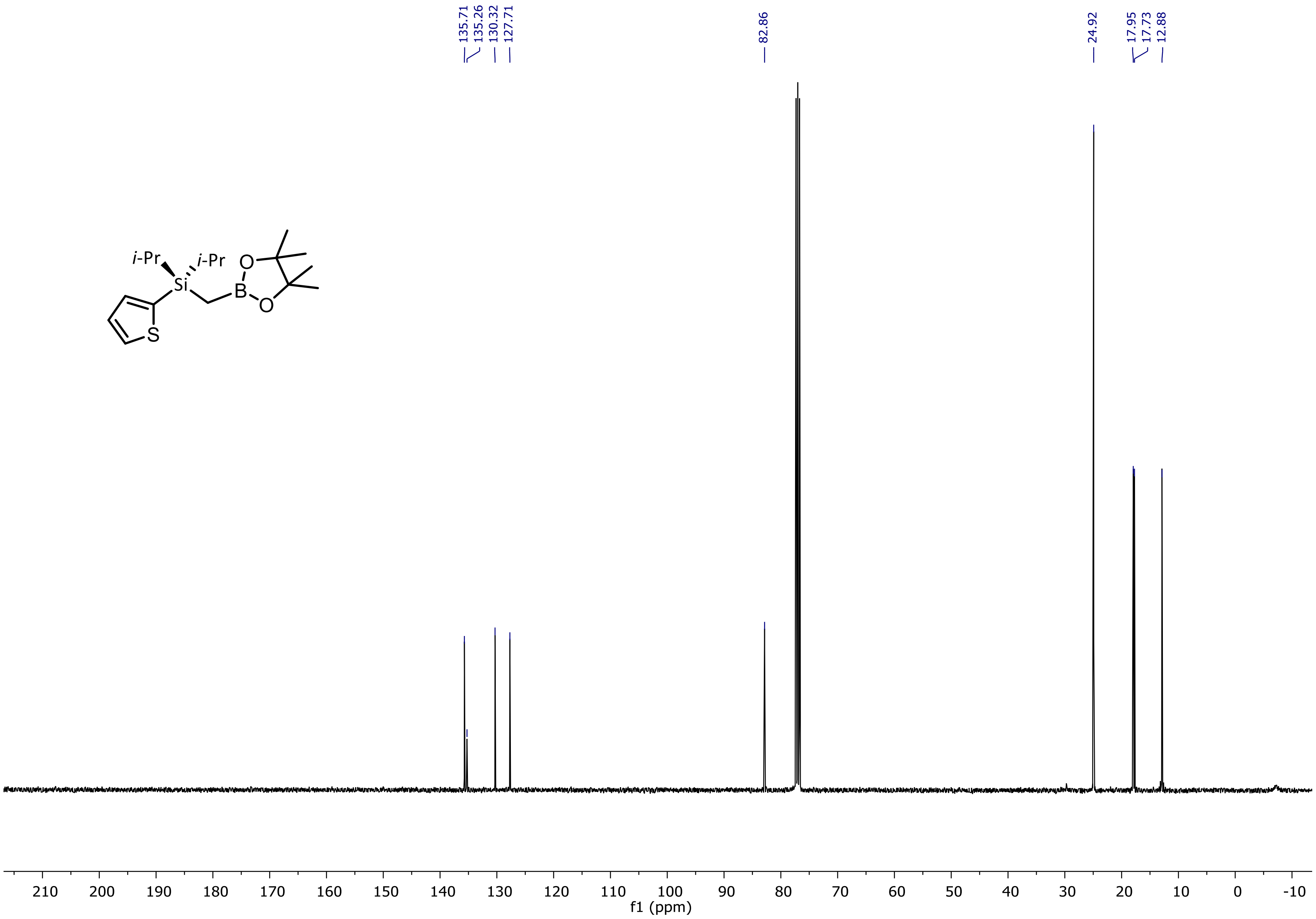

*^{1}H-NMR (400 MHz, $CDCl_3$) for compound **1o***

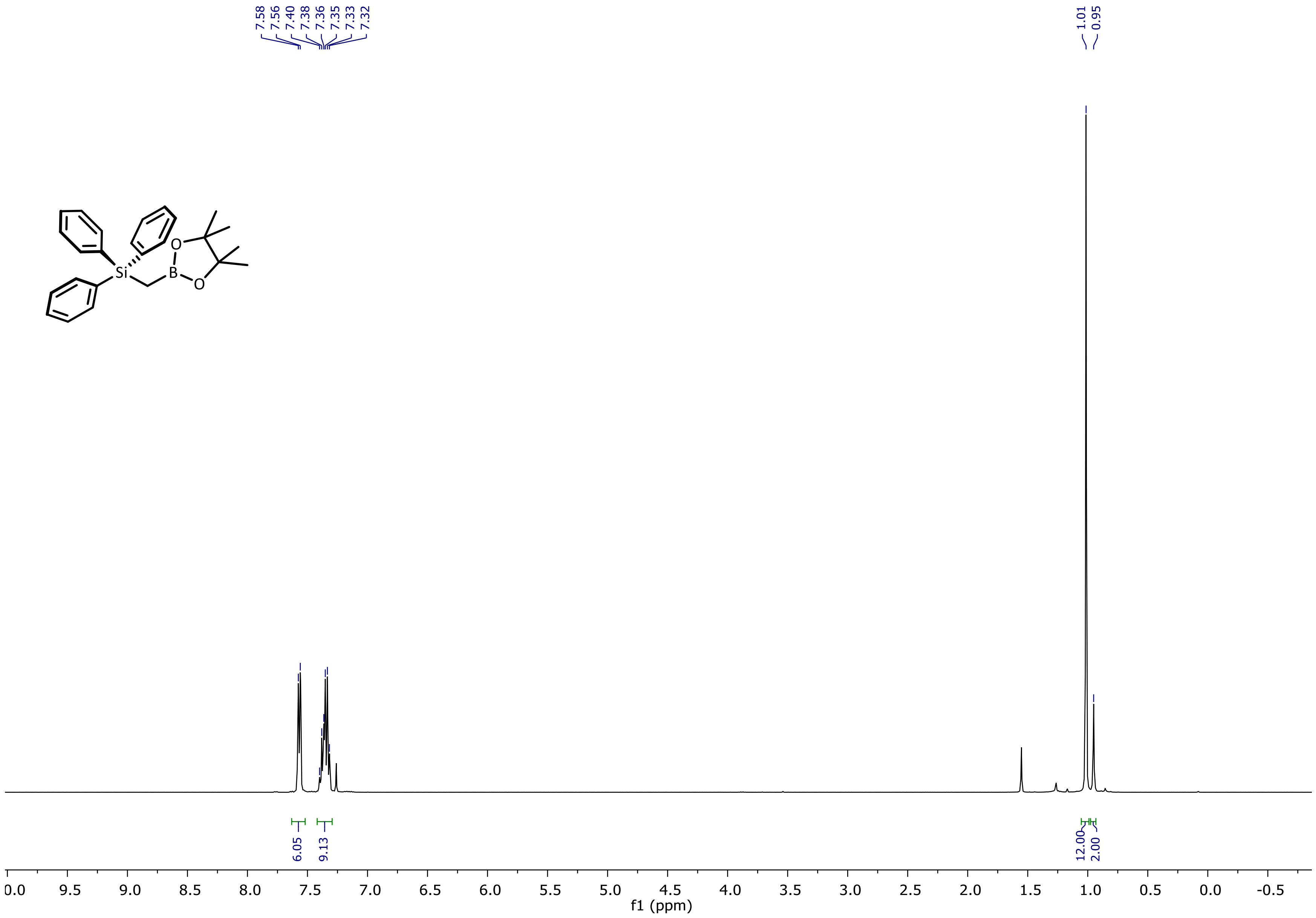

*^{13}C-NMR (101 MHz, CDCl$_3$) for compound **1o***

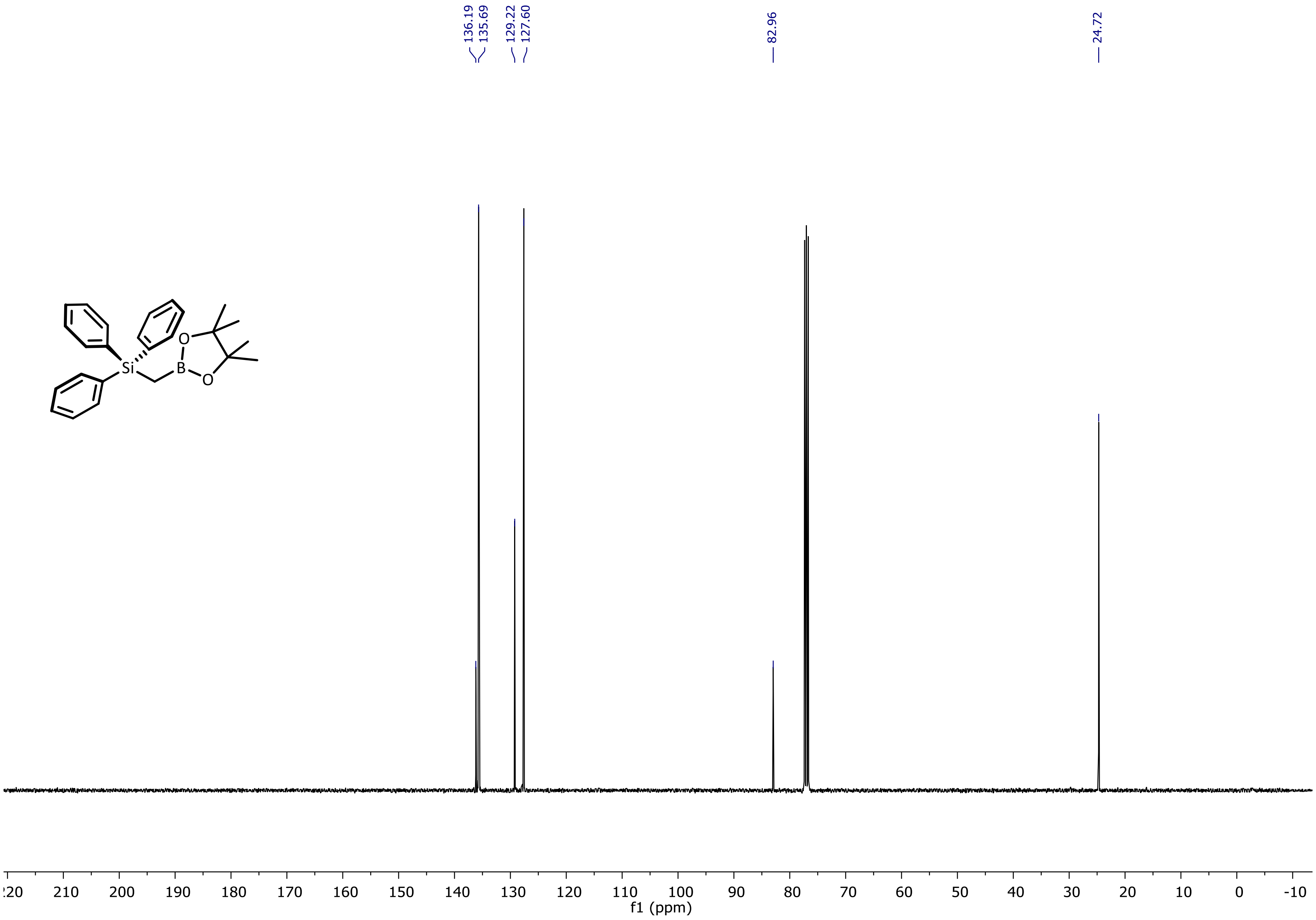

*^{1}H-NMR (400 MHz, $CDCl_3$) for compound **1p***

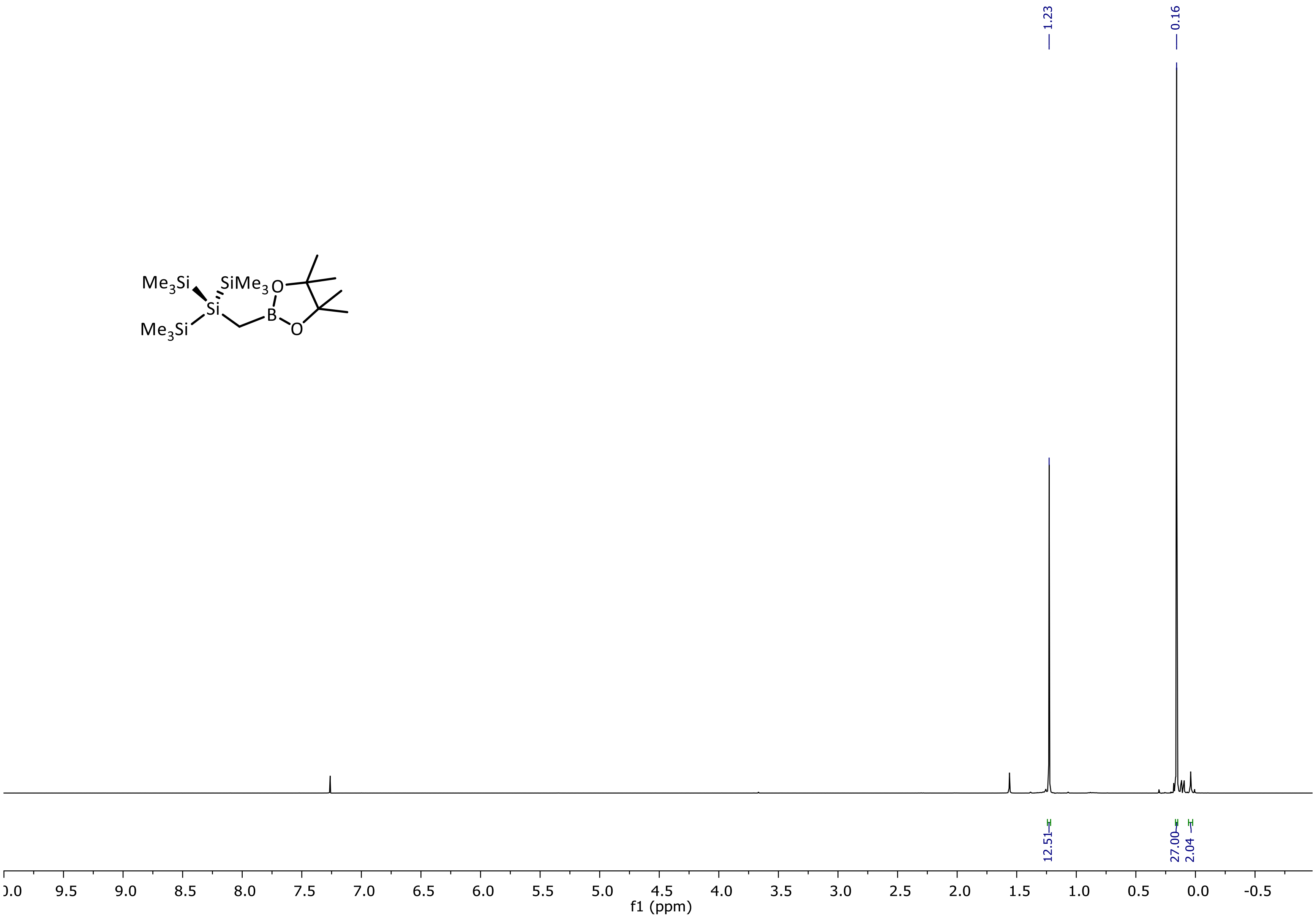

*^{13}C-NMR (101 MHz, CDCl₃) for compound **1p***

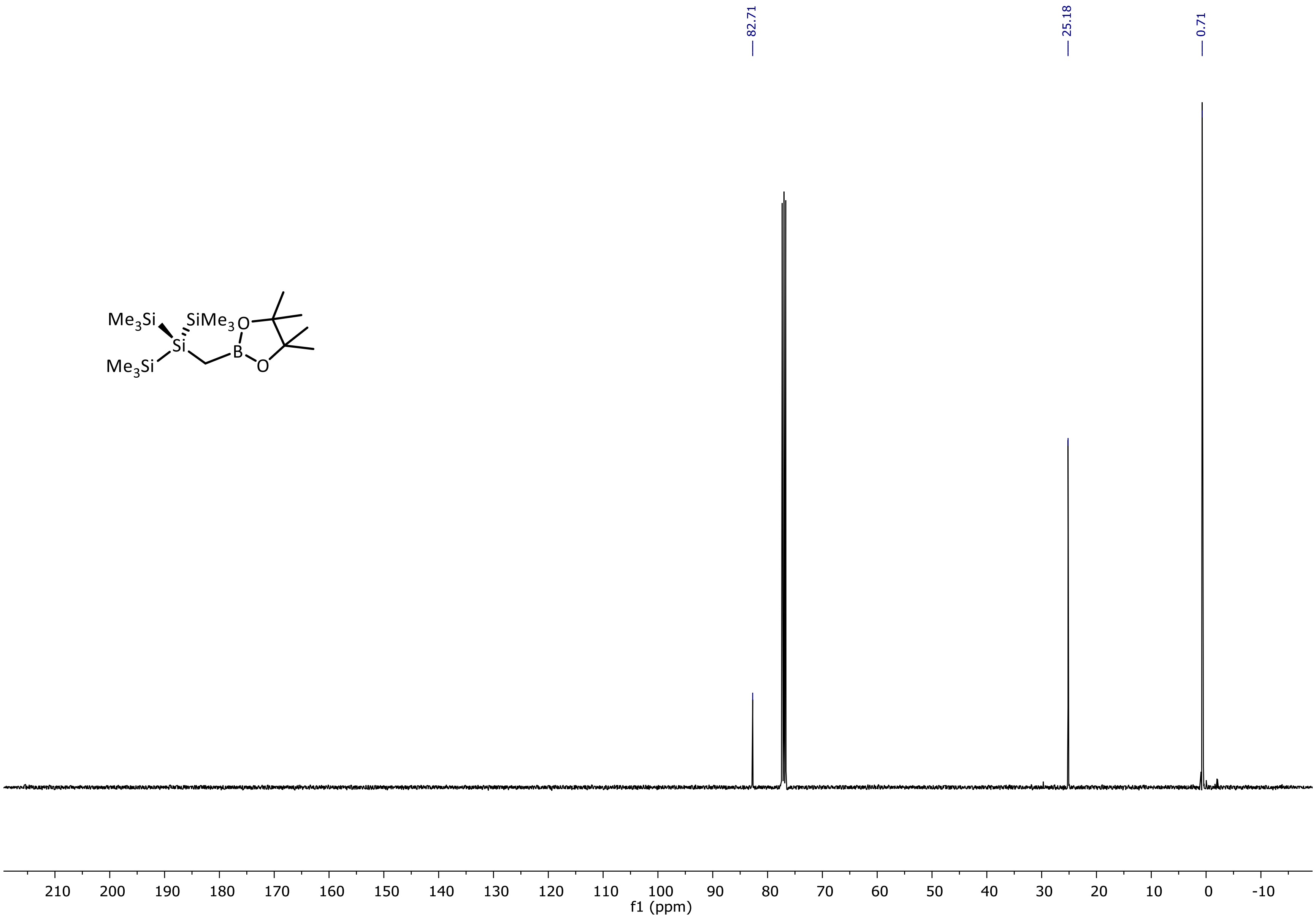

^{1}H-NMR (400 MHz, $CDCl_3$) for compound **1q**

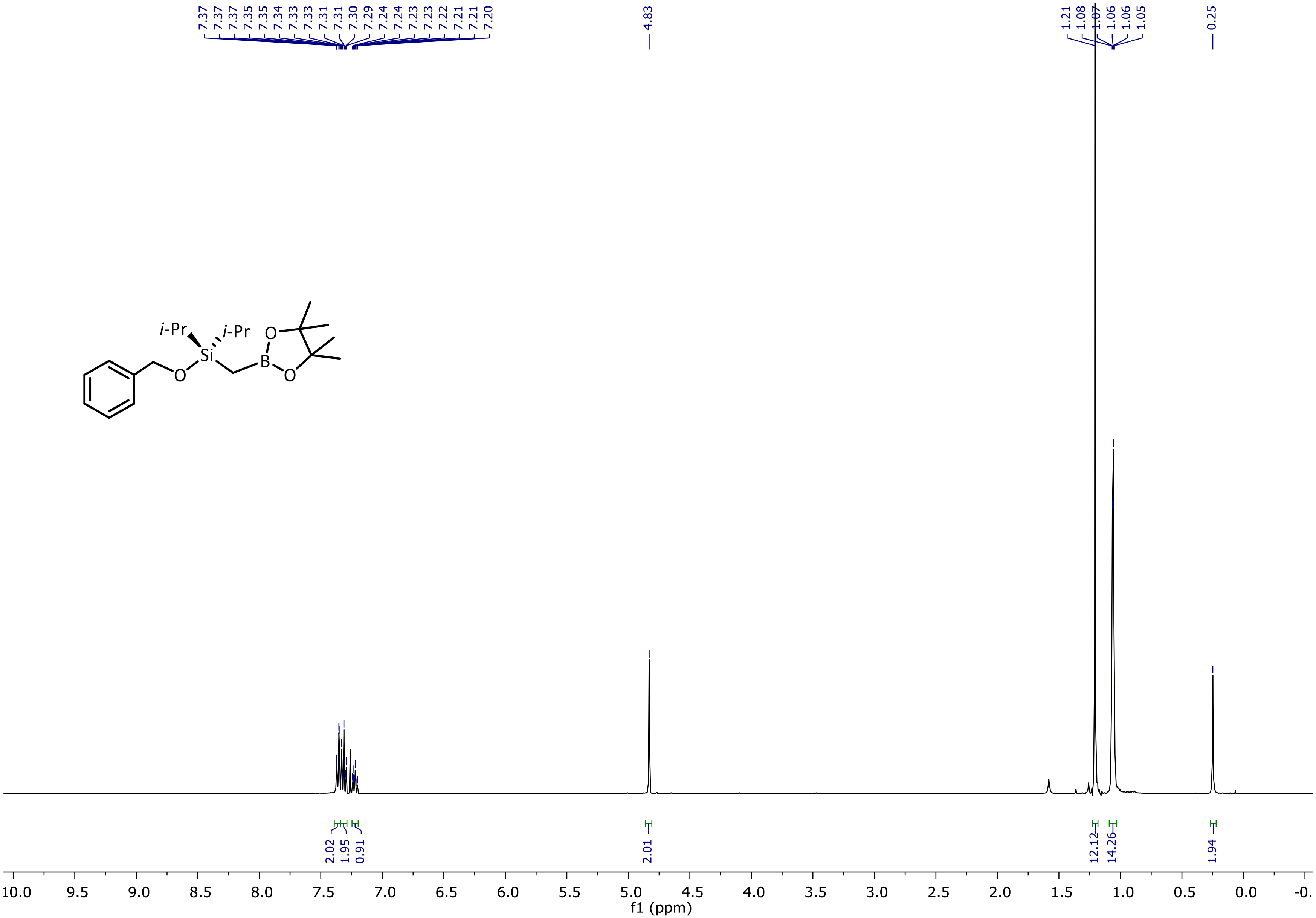

*^{13}C-NMR (101 MHz, CDCl$_3$) for compound **1q***

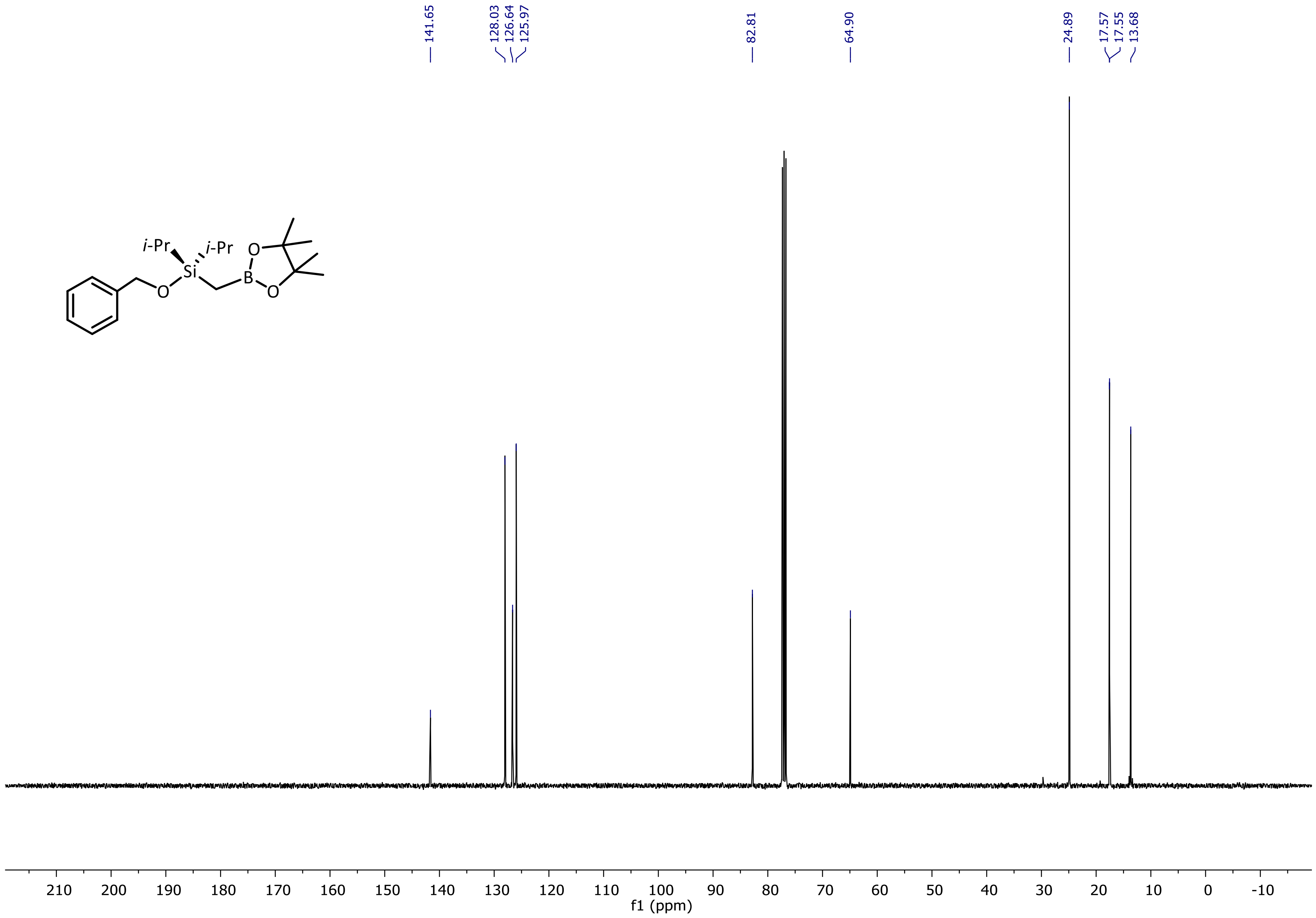

^{1}H-NMR (400 MHz, $CDCl_3$) for compound **1r**

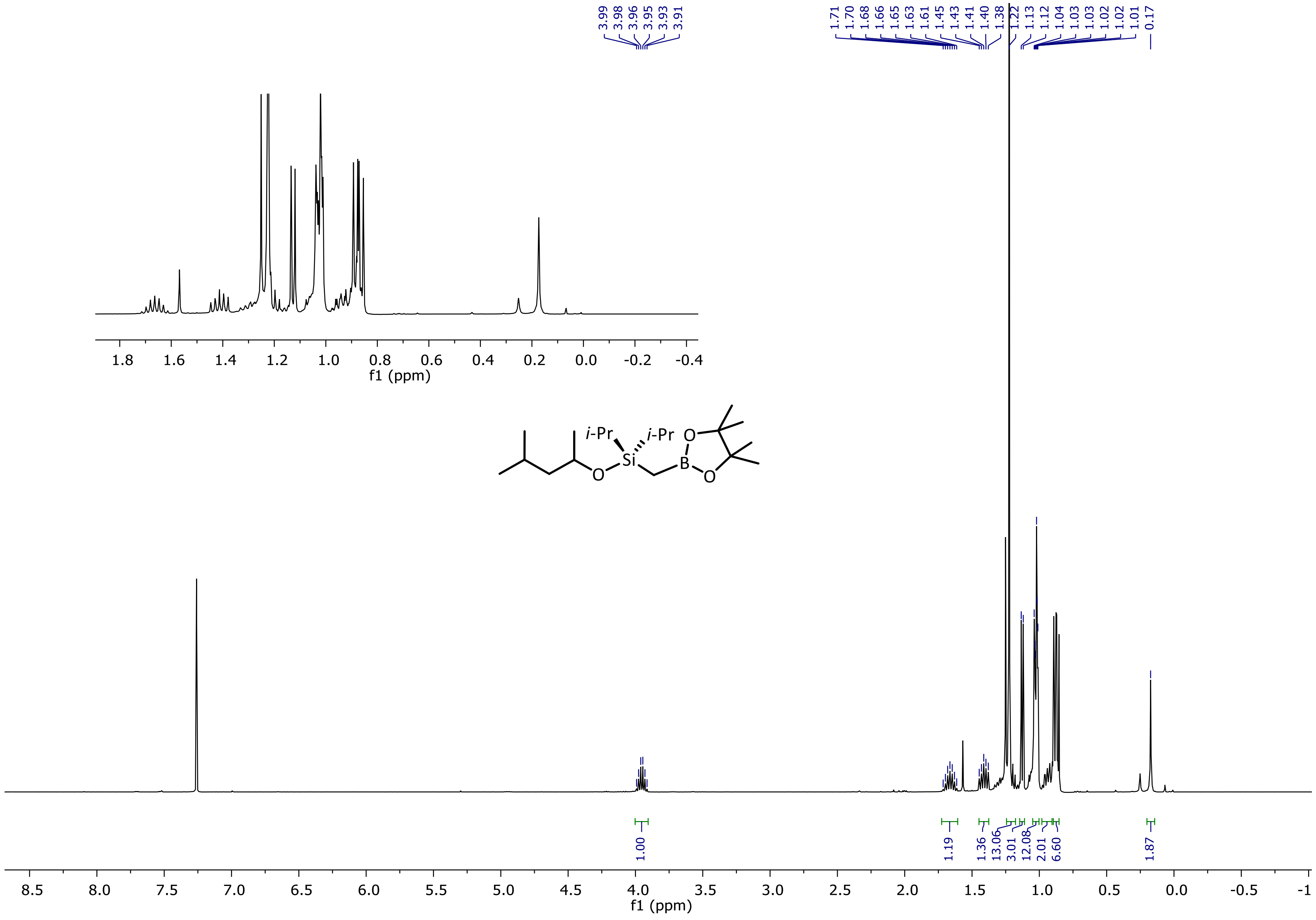

^{13}C-NMR (101 MHz, $CDCl_3$) for compound **1r**

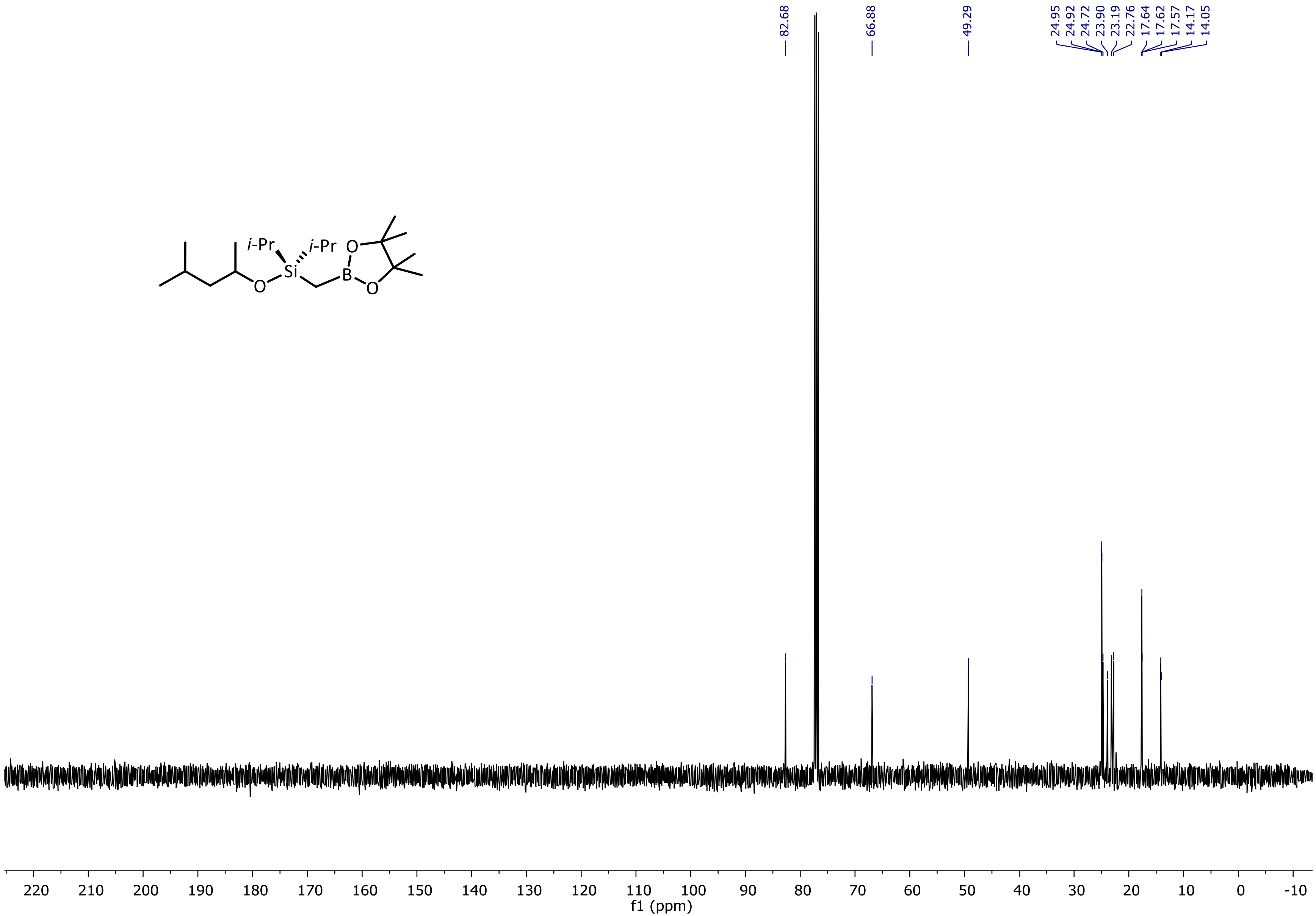

*HSQC-NMR (CDCl$_3$) for compound **1r***

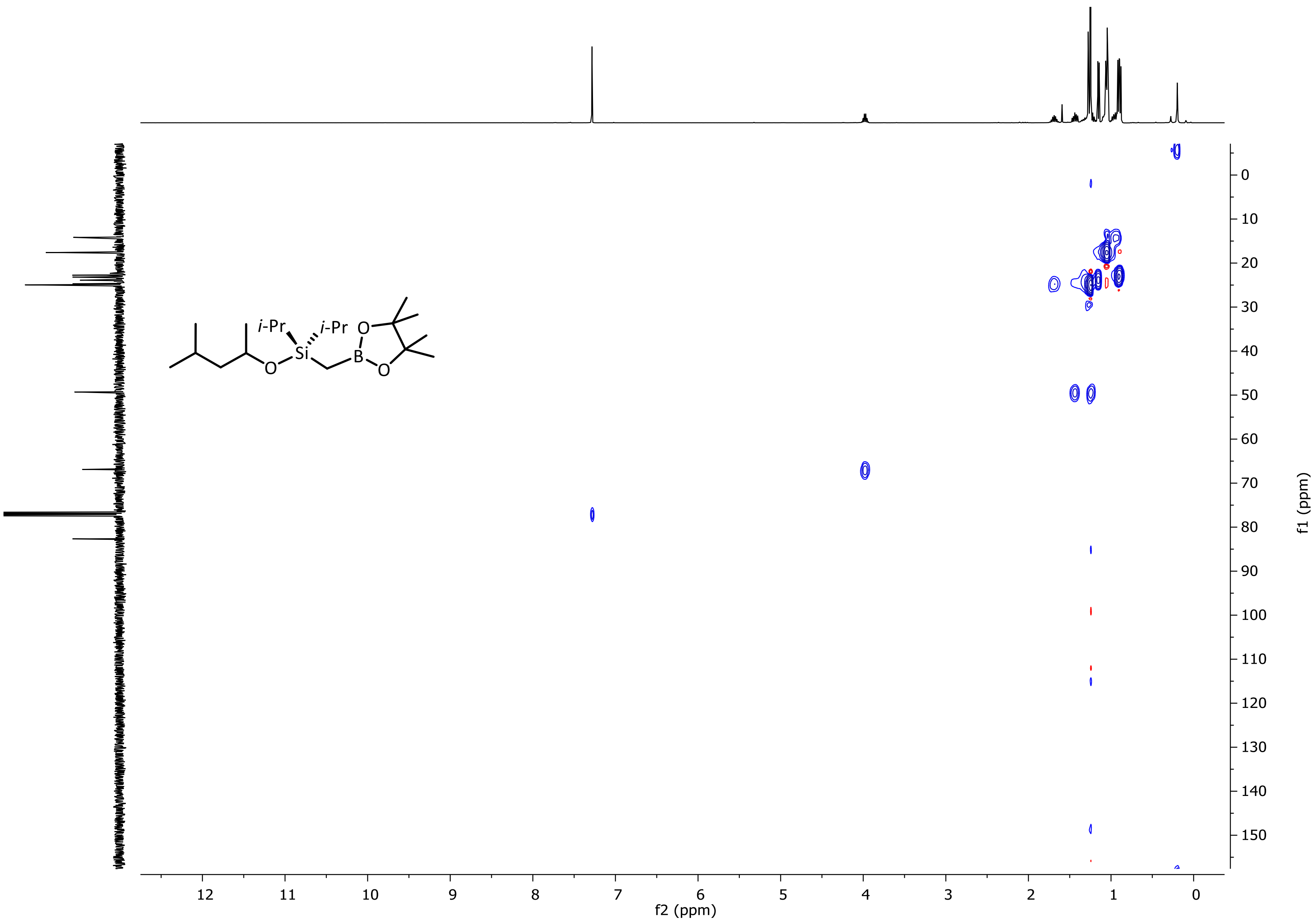

^{1}H-NMR (400 MHz, $CDCl_3$) for compound ***1s***

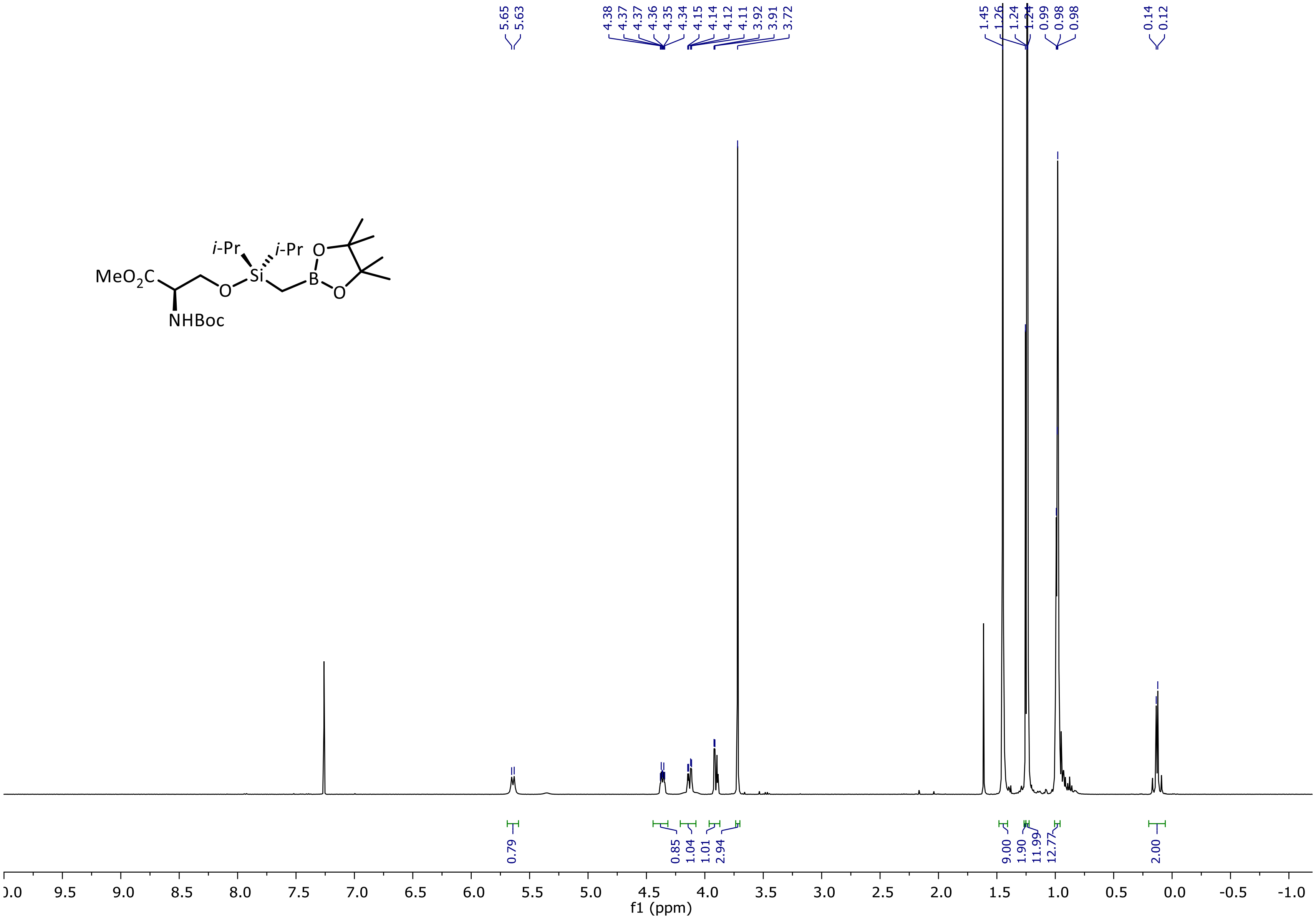

^{13}C-NMR (101 MHz, $CDCl_3$) for compound **1s**

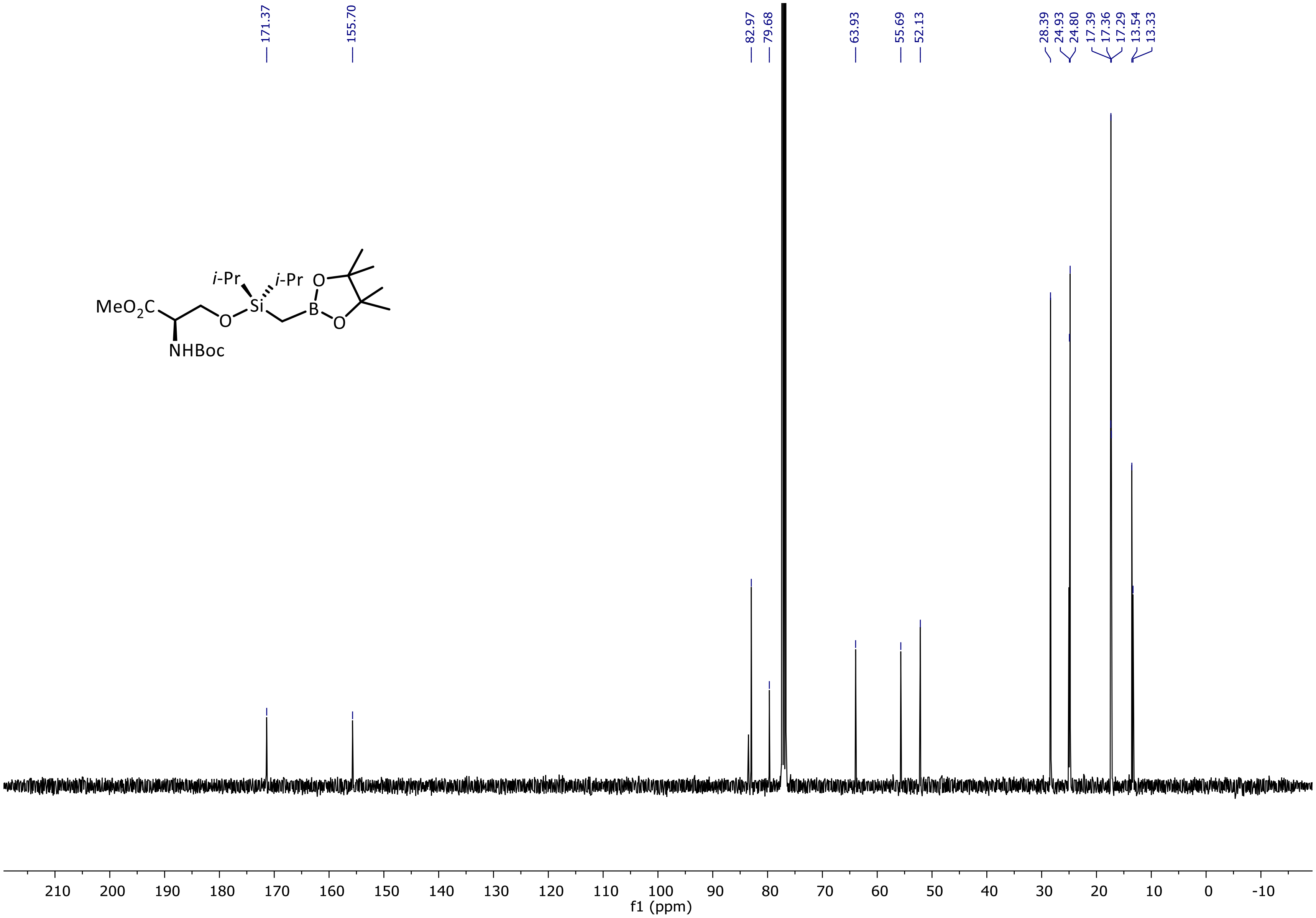

*^{1}H-NMR (400 MHz, $CDCl_3$) for compound **1t***

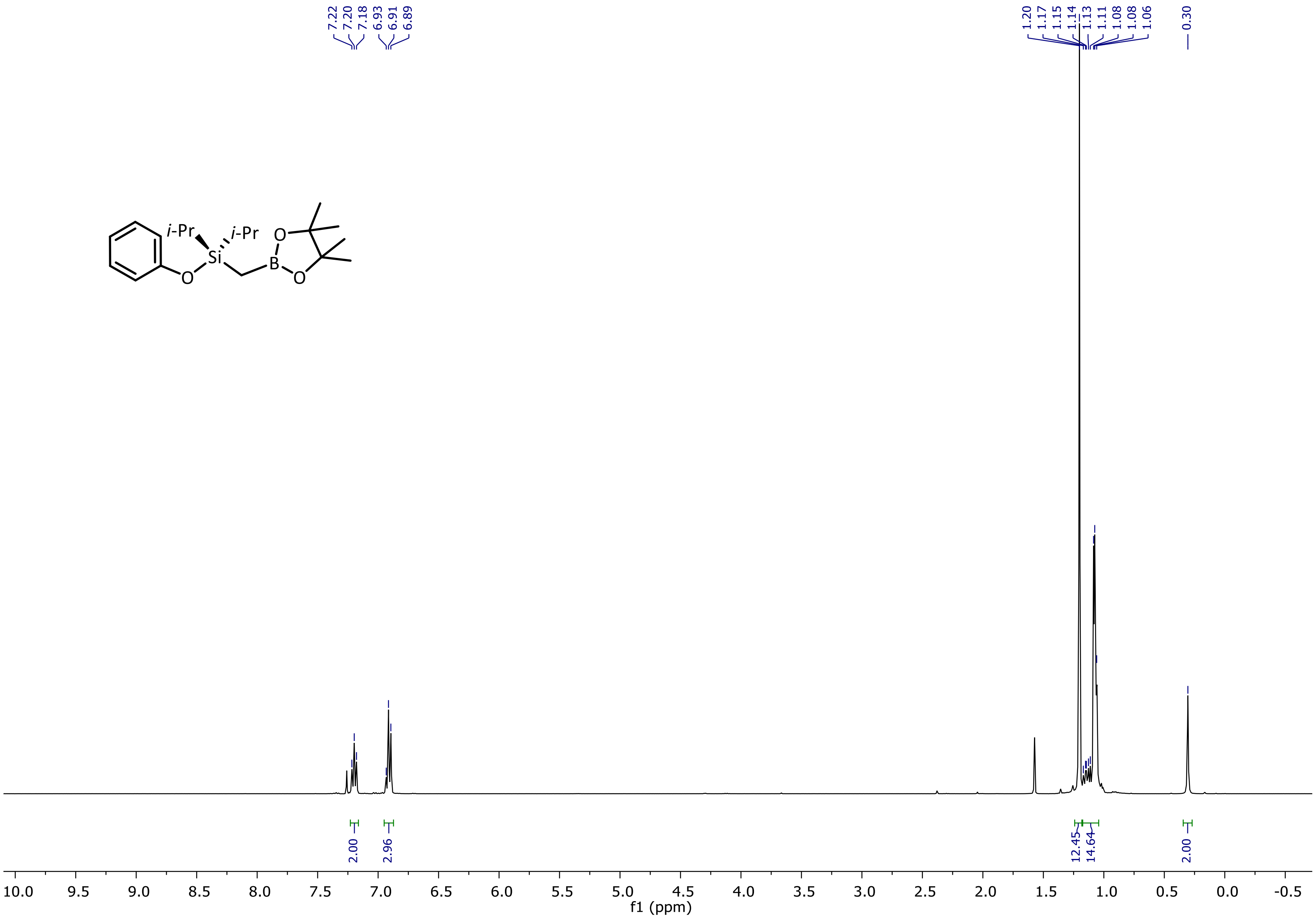

*^{13}C-NMR (101 MHz, CDCl$_3$) for compound **1t***

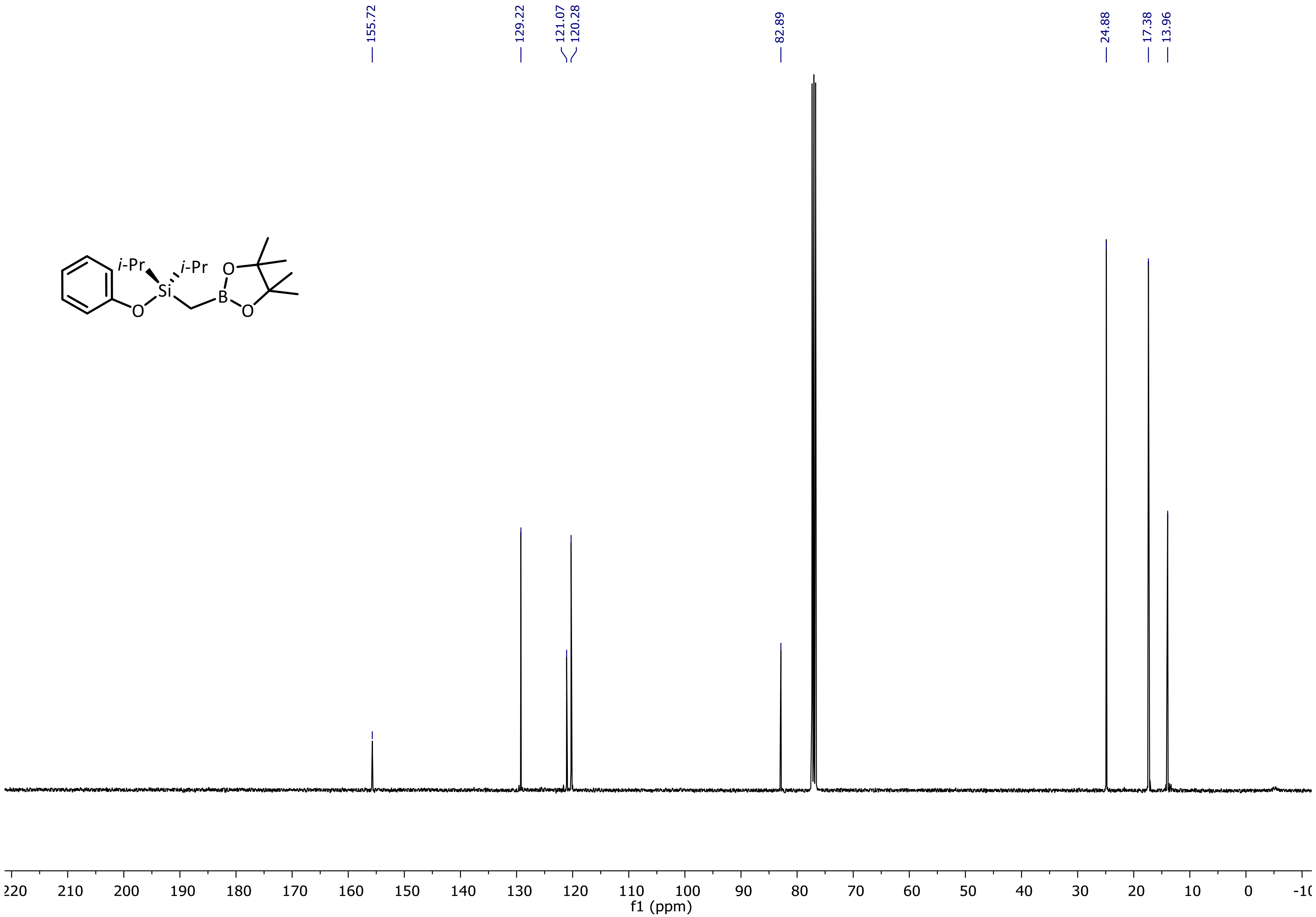

^{1}H-NMR (400 MHz, $CDCl_3$) for compound ***1u***

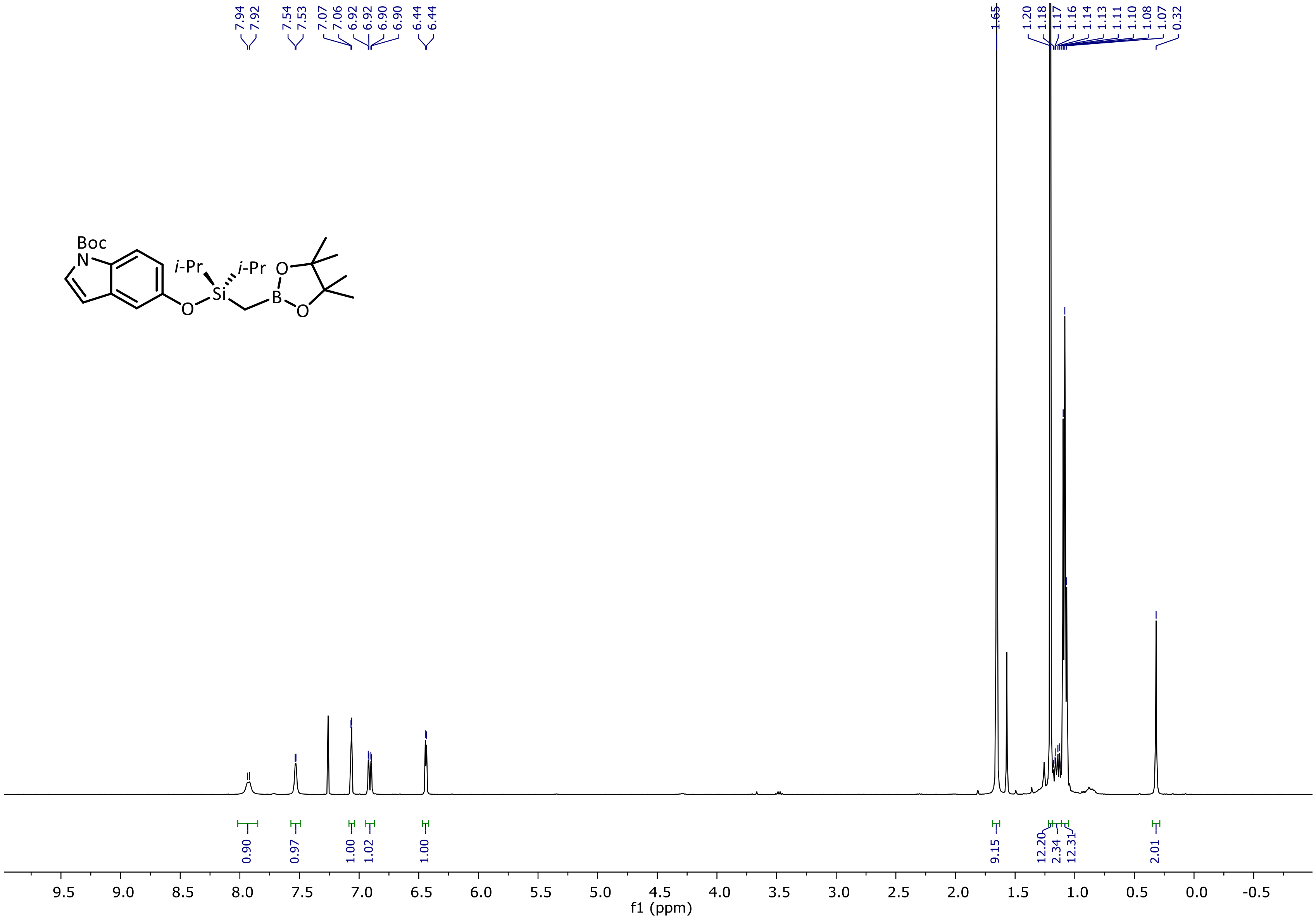

*^{13}C-NMR (101 MHz, CDCl₃) for compound **1u***

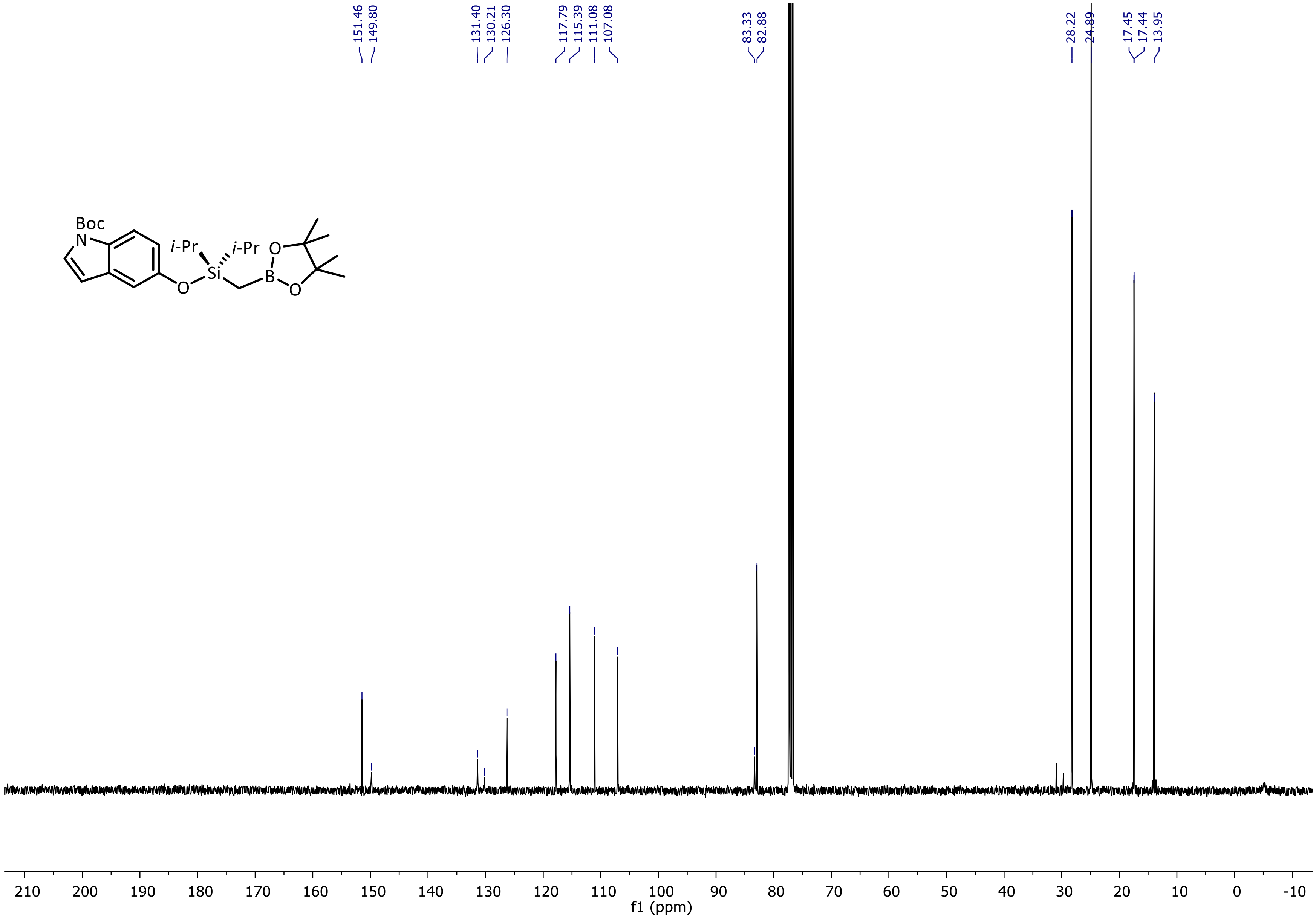

*^{1}H-NMR (400 MHz, $CDCl_3$) for compound **1v***

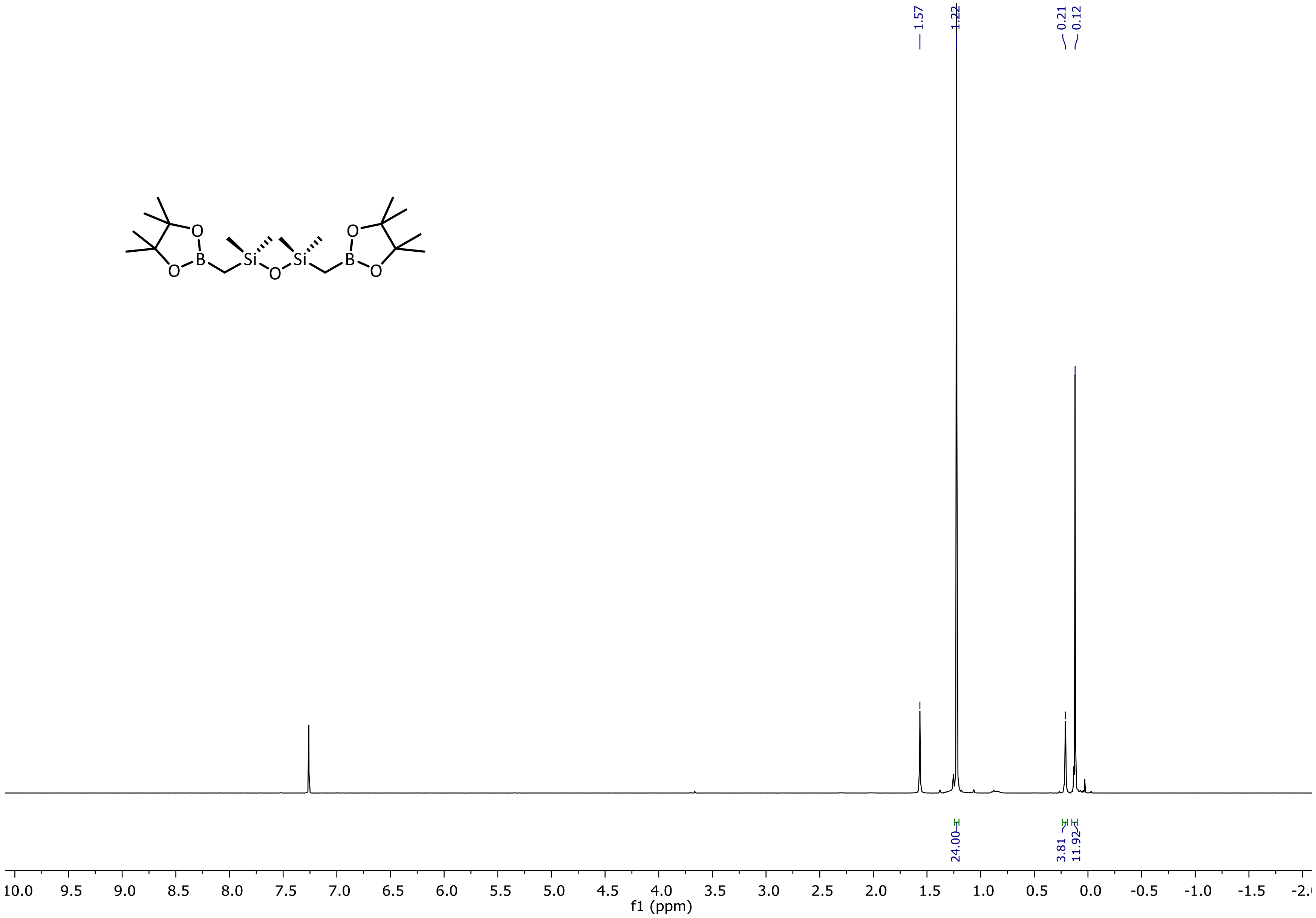

^{13}C-NMR (101 MHz, CDCl$_3$) for compound **1v**

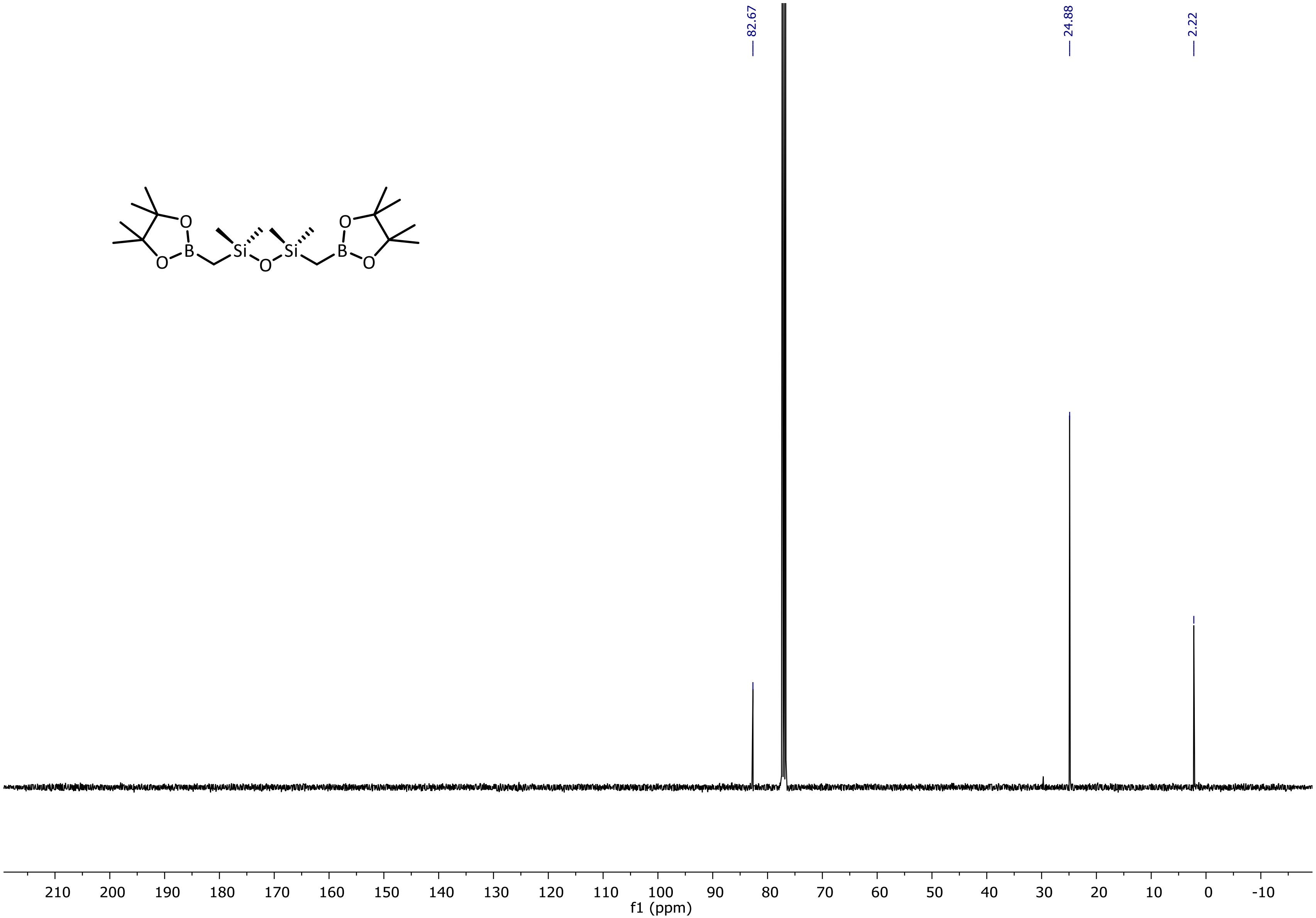

Supplement: Supplementary file 1 — ol3c00474_si_001.pdf [file ol3c00474_si_001.pdf]
